# Genome-wide identification of alternate bearing-associated microRNAs (miRNAs) in olive (*Olea europaea* L.)

**DOI:** 10.1186/1471-2229-13-10

**Published:** 2013-01-15

**Authors:** Huriye Yanik, Mine Turktas, Ekrem Dundar, Pilar Hernandez, Gabriel Dorado, Turgay Unver

**Affiliations:** 1 Faculty of Science, Department of Biology, Cankiri Karatekin University, 18100, Cankiri, Turkey; 2 Department of Biology, Balikesir University, Faculty of Art and Science, 10145, Balikesir, Turkey; 3 Instituto de Agricultura Sostenible (IAS-CSIC), Alameda del Obispo s/n, 14080, Córdoba, Spain; 4 Dep. Bioquímica y Biología Molecular, Campus Rabanales C6-1-E17, Campus de Excelencia Internacional Agroalimentario (ceiA3), Universidad de Córdoba, 14071, Córdoba, Spain

**Keywords:** **H**igh-throughput small RNA sequencing, MicroRNA, Olive, Periodicity

## Abstract

**Background:**

Alternate bearing is a widespread phenomenon among crop plants, defined as the tendency of certain fruit trees to produce a high-yield crop one year ("on-year"), followed by a low-yield or even no crop the following year **(**"off-year"). Several factors may affect the balance between such developmental phase-transition processes. Among them are the microRNA (miRNA), being gene-expression regulators that have been found to be involved as key determinants in several physiological processes.

**Results:**

Six olive (*Olea europaea* L. cv. Ayvalik variety) small RNA libraries were constructed from fruits (ripe and unripe) and leaves (”on year” and ”off year” leaves in July and in November, respectively) and sequenced by high-throughput Illumina sequencing. The RNA was retrotranscribed and sequenced using the high-throughput Illumina platform. Bioinformatics analyses of 93,526,915 reads identified 135 conserved miRNA, belonging to 22 miRNA families in the olive. In addition, 38 putative novel miRNAs were discovered in the datasets. Expression of olive tree miRNAs varied greatly among the six libraries, indicating the contribution of diverse miRNA in balancing between reproductive and vegetative phases. Predicted targets of miRNA were categorized into 108 process ontology groups with significance abundance. Among those, potential alternate bearing-associated processes were found, such as development, hormone-mediated signaling and organ morphogenesis. The KEGG analyses revealed that the miRNA-targeted genes are involved in seven main pathways, belonging to carbohydrate metabolism and hormone signal-transduction pathways.

**Conclusion:**

A comprehensive study on olive miRNA related to alternate bearing was performed. Regulation of miRNA under different developmental phases and tissues indicated that control of nutrition and hormone, together with flowering processes had a noteworthy impact on the olive tree alternate bearing. Our results also provide significant data on the miRNA-fruit development interaction and advance perspectives in the miRNA profile of the olive tree.

## Background

The olive tree (*Olea europaea* L.) belongs to the *Oleaceae* family and is an evergreen plant native to the Mediterranean Basin. It is one of the most economically important fruit crops in the world. Interestingly, the olive oil has been declared as a healthy medicine for cardiovascular protection (“qualified health claim”) by the internationally recognized Food and Drug Administration (FDA) of the United States of America (USA) <http://www.fda.gov/Food/LabelingNutrition/LabelClaims/QualifiedHealthClaims/default.htm>, due to its protective effect against cardiovascular diseases, being the third of such labels approved for a conventional food (after the hazelnut and omega-3 fatty acids) <http://www.fda.gov/Food/LabelingNutrition/LabelClaims/QualifiedHealthClaims/ucm072756.htm>.

In addition to its agricultural and dietary values, the olive tree is particularly known for its tendency to bear fruits in an uneven manner (alternate bearing, biennial bearing, uneven bearing or periodicity) [1,2].

This is a well-known pomology phenomenon among crop plants, defined as the tendency of fruit trees to produce a high-yield crop one year ("on-year"), followed by a low-yield or even no crop the following year ("off-year"). Thus, this phenomenon may severely affect the fruit yield of many trees [3,4]. The floral induction of the olive tree starts in July, with the floral buds being initiated in November. The differences between on- and off-year product yield varies between 5 to 30 t/ha representing a serious problem of the olive industry [4]. The harvest yield variation depends on genetic and physiological factors, as well as environmental conditions. To decrease the severity of the alternate bearing, a variety of agronomical practices are being applied [1]. It has been proposed that three main factors had influence the alternate bearing: flower-site, endogenous plant growth hormones, and carbohydrate storage [3]. The life cycle of flowering plants undergoes transitions from the juvenile to the adult stage of the vegetative phase, and then may enter the reproductive phase, which is under the tight control of a complex genetic network [5]. Thus, discovering the control mechanisms underlying these transitions represents a crucial step to understand the molecular bases of such processes. Thus, discovering control mechanisms of these transitions is crucial to understand the basis of this tendency.

The MicroRNA (miRNA) are non-coding small RNA (sRNA) found in diverse eukaryotes, negatively regulating specific target messenger RNA (mRNA) [6]. The plant miRNA range in size from 20 to 24 bases (b) [7]. They act as key regulators in several processes, such as development and stress responses [8,9]. So far, their involvement in developmental regulation and flowering processes has been studied in various plants [10-16]. Several miRNA discovery methods have been employed by researchers, such as computational prediction, quantitative Reverse-Transcription Polymerase Chain Reaction (qRT-PCR or qPCR), etc. [17-22]. However, they are not always effective, since prior sequence information is a requirement to use such techniques. Thus, the absence of olive tree miRNA in databases limits the applicability of such approaches to such speciess. Fortunately, the new high-throughput sRNA sequencing allows to identify miRNA de novo; without any previous sequence information. Low-abundance miRNA can also be detected by this method, while they are difficult to identify using traditional methodologies.

In this study, we aimed to identify miRNA regulated by alternate bearing in *O. europaea* L. A total of 93,526,915 raw reads were produced from six libraries. We detected 135 previously known miRNA belonging to 22 families, and 38 novel ones. The differential expression of the miRNA was evaluated on the basis of the developmental phase of the samples.

## Results

### Small RNA sequencing

**Figure 1:**
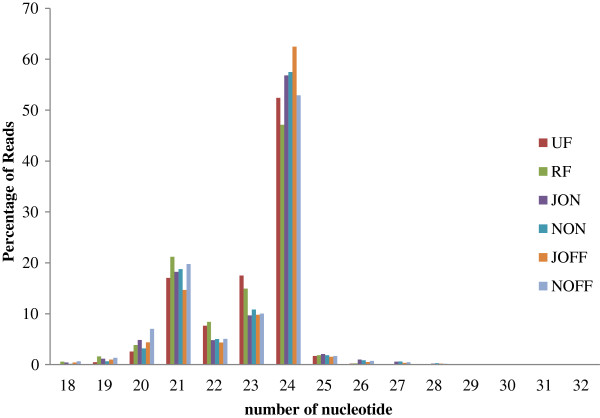
**Length distribution of sRNA.** The length distribution of high-quality sequences were obtained from six *O. europaea* libraries. The distribution of the total reads is shown as percentages. UF: unripe fruit; RF: ripe fruit; JON: “on-year” July leaf; NON: “on-year” adult leaf; JOFF: “off-year” July leaf; and NOFF: “off-year” adult leaf.

In order to determine responsive sRNAs for alternate bearing, six small RNA libraries were constructed from "on-year" and “off year” leaves in July (JON and JOFF, respectively), again in "on-year" and “off year” leaves in November (NON and NOFF, respectively) as well as with ripe (RF) and unripe (UF) fruits. A total of 93.526.915 raw reads were generated with the high-throughput Illumina HiSeq 2000 Sequencing System,, with about 15,587,819 reads from each library (Table 1). After processing of primary reads, 15,260,014, 13,817,321, 16,950,209, 15,153,468, 15,931,860 and 15,710,421 total clean reads were counted for the UF, RF, JON, NON, JOFF and NOFF libraries, respectively. The size distributions of the reads in the six datasets were quite similar. The length of the sRNA varied from 18 to 32 bases (b), being 24 b the most abundant read length or statistical mode (Figure 1).

**Table 1:** Raw and clean read statistics of small RNAs

	**UF**		**RF**		**NON**		**JON**		**NOFF**		**JOFF**	
	**Count**	**%**	**Count**	**%**	**Count**	**%**	**Count**	**%**	**Count**	**%**	**Count**	**%**
**total_reads**	15,364,727		13,895,311		15,310,134		17,043,189		15,849,260		16,064,294	
**high_quality**	15,340,544	100	13,868,830	100	15,288,291	100	17,018,648	100	15,825,515	100	16,041,414	100
**3’adapter_null**	4,407	0.03	4,141	0.03	3,703	0.02	3,941	0.02	4,334	0.03	3,937	0.02
**insert_null**	6,029	0.04	1,148	0.01	3,681	0.02	2,750	0.02	2,995	0.02	4,423	0.03
**5’adapter_contaminants**	46,316	0.30	12,104	0.09	93,196	0.61	18,866	0.11	4,0797	0.26	38,204	0.24
**smaller_than_18nt**	23,051	0.15	33,526	0.24	32,945	0.22	42,170	0.25	66,057	0.42	62,461	0.39
**polyA**	727	0.00	590	0.00	1,298	0.01	712	0.00	911	0.01	529	0.00
**clean_reads**	15,260,014	99.48	13,817,321	99.63	15,153,468	99.12	16,950,209	99.60	15,710,421	99.27	15,931,860	99.32

**Figure 2:**
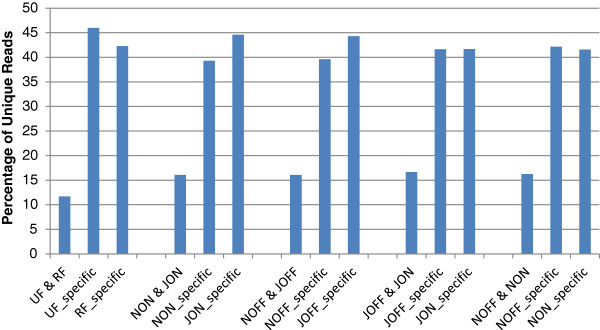
**Common and specific sRNA tags between libraries.** The histograms show the miRNA shared between the analyzed libraries (five comparisons). The shared and unique miRNA in each library are shown as percentages. See legend of Figure 1.

The common and specific unique tags were identified for the UF vs. RF, JON vs. NON, JOFF vs. NOFF, JOFF vs. JON and NOFF vs. NON libraries (Figure 2). About 12% of the sequences were shared between the fruit libraries (UF and RF), and about 16% for the others (NON and JOIN, NOFF and JOFF, JOFF and JON, and NOFF and NON). The UF library had a higher amount of specific sequences than the RF one, while the ratio was slightly lower for JON and JOFF compared to NON and NOFF, respectively. Almost equal amounts of specific sequences were counted for each JOFF, JON, NOFF and NON libraries (Figure 2).

**Figure 3:**
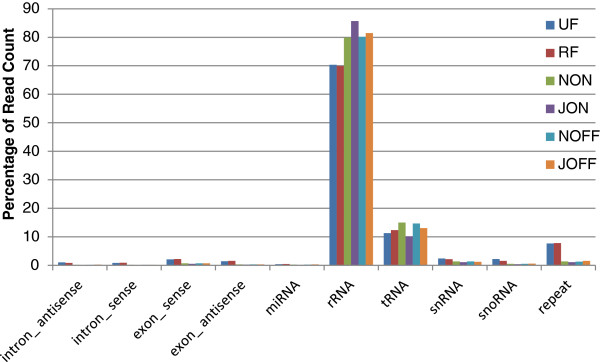
**Classification of unique reads into different sRNA groups.** The reads were categorized into 10 different classes of sRNA, showing their distributions as percentages.

The sRNA tags were grouped into unique sequences on the basis of their identity (7,933,475 for UF; 7,423,620 for RF; 5,479,750 for JON; 6,003,166 for NON; 5,535,758 for JOFF; and 6,001,443 for NOFF), and they were mapped to the *Populus trichocarpa* genome by SOAP2 [23]. A very low percentage of the sRNA sequences matched such genome (Table 2). For example, out of 7,933,475 unique sRNA in the UF library, only 26,242 reads (0.33%) were mapped that way. The reads were categorized into different classes of sRNA, such as ribosomal RNA (rRNA), transfer RNA (tRNA), small nuclear RNA (snRNA), small nucleolar RNA (snoRNA), etc., by aligning them with the *Populus trichocarpa* genome of the Rfam <http://www.sanger.ac.uk/resources/databases/rfam.html> and GenBank <http://www.ncbi.nlm.nih.gov/genbank> databases (Figure 3). Most of the sRNA of each library were annotated as rRNA, followed by tRNA. A few reads were derived from repeated sequences, while the majority of sRNA remained as unannotated. Compared to "on-year" derived libraries (JOFF and NOFF), "off-year" datasets (JON and NON) contained a slightly higher number of unique sRNA, with a similar pattern being observed between ripe- and unripe-fruit-derived libraries (Table 2). About 200 unique reads were considered as miRNA in two libraries constructed from fruits (UF and RF), while four leaf derived libraries (JON, NON, JOFF and NOFF) included about 300 miRNA. The libraries derived from the "off-year" leaves (NOFF and JOFF) had the highest number of miRNA (319 and 337, respectively) (Figure 1).

**Table 2:** Mapping statistics of the sRNA*

**Libraries**	**Unique sRNAs**	**Percentage (%)**	**Total sRNAs**	**Percentage (%)**
UF	26,242	0.33	850,744	5.57
RF	26,711	0.36	1,219,147	8.82
JON	26,735	0.45	1,876,682	11.07
NON	22,769	0.42	1,353,664	8.93
JOFF	28,383	0.47	1,412,100	8.86
NOFF	27,589	0.50	2,126,303	13.53

*See Table 1.

### Known miRNA and expression levels

**Figure 4:**
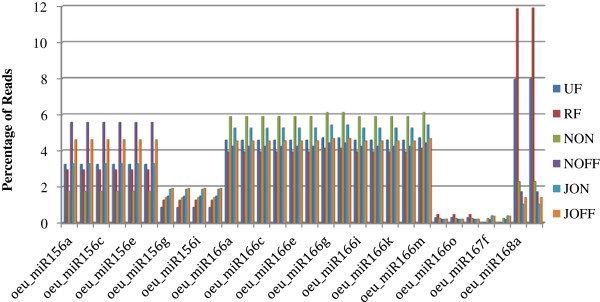
**The most abundantly expressed miRNA.** The percentages of the most abundant miRNAs among all the miRNAs were given.

Although different miRNA have been identified for several plant species, the sequence information of the olive miRNAs is missing in the miRBase <http://www.mirbase.org> and PMRD <http://bioinformatics.cau.edu.cn/PMRD> databases. Recently, RNA silencing-associated sRNAs in *O. europaea* L. were reported [24]. To identify conserved and new miRNAs from six olive tree libraries, sRNAs were subjected to a nucleotide-nucleotide BLAST (blastn) search <http://www.ncbi.nlm.nih.gov/Class/MLACourse/Modules/BLAST/nucleotide_blast.html> using the miRBase version 18.0, including 4,014 Viridiplantae miRNA belonging to 52 plant species. After filtering, the sequences were aligned with the *P. trichocarpa* genome. Due to conservation of miRNA families between closely related species [25,26], olive miRNAs were validated in poplar genome which was fully sequenced. Comparing sequence information, 136 conserved miRNAs were identified, belonging to 22 miRNA families (Additional file 1). The identified miRNA families were also found to be conserved in various plant species [20,21,25-27]. The abundance of the defined miRNAs was determined by their read frequencies in the libraries (Table 3). Some of the miRNA were represented in equivalent amounts in each library, but the read counts for most of the miRNA varied between datasets (Figure 4). As expected, some miRNA showed library-specific expression patterns. Additionally, some miRNA were found at very low amounts, albeit with unique expression profiles. For example, the miR319e, miR319f, miR319g and miR319h (Additional files 1 and 2) appeared only in the "off-year" leaf libraries (NOFF and JOFF).

**Table 3:** Read counts of known miRNA in each library

**miRNA_Name**	**UF**	**RF**	**NON**	**NOFF**	**JON**	**JOFF**
oeu_miR156a	90,292	39,031	124,269	643,286	339,746	311,706
oeu_miR156b	90,252	39,016	124,180	643,040	339,621	311,629
oeu_miR156c	90,292	39,031	124,269	643,286	339,746	311,706
oeu_miR156d	90,218	39,076	124,178	643,011	339,606	311,576
oeu_miR156e	90,218	39,076	124,178	643,011	339,606	311,576
oeu_miR156f	90,292	39,031	124,269	643,286	339,746	311,706
oeu_miR156g	24,167	16,890	100,442	172,856	193,886	130,260
oeu_miR156h	24,044	16,818	100,233	172,426	193,558	129,940
oeu_miR156i	24,167	16,890	100,442	172,856	193,886	130,260
oeu_miR156j	24,167	16,890	100,442	172,856	193,886	130,260
oeu_miR156k	78	31	135	691	337	306
oeu_miR159a	605	363	2,593	3,673	1,908	7,809
oeu_miR159b	605	363	2,591	3,673	1,909	7,808
oeu_miR159c	605	363	2,593	3,673	1,908	7,809
oeu_miR159d	1	0	0	2	2	0
oeu_miR160a	4	2	4	83	18	109
oeu_miR160b	2	2	1	69	11	98
oeu_miR160c	2	2	1	69	11	98
oeu_miR160d	4	2	4	83	18	109
oeu_miR160g	0	0	0	1	0	0
oeu_miR164a	63	28	1,577	2,050	3,133	4,902
oeu_miR164b	63	28	1,575	2,046	3,136	4,896
oeu_miR164c	63	28	1,575	2,046	3,131	4,895
oeu_miR164d	63	28	1,577	2,050	3,133	4,902
oeu_miR164e	64	28	1,602	2,066	3,166	4,955
oeu_miR164f	1	2	0	0	0	1
oeu_miR166a	127,248	52,310	419,523	491,358	542,522	306,999
oeu_miR166b	127,229	52,256	419,792	491,598	543,180	307,541
oeu_miR166c	127,212	52,264	419,434	491,303	542,458	306,940
oeu_miR166d	127,249	52,247	419,941	491,769	543,446	307,715
oeu_miR166e	127,229	52,256	419,792	491,598	543,180	307,541
oeu_miR166f	127,249	52,247	419,941	491,769	543,446	307,715
oeu_miR166g	130,974	55,055	435,594	510,783	560,419	317,070
oeu_miR166h	130,974	55,056	435,594	510,783	560,419	317,070
oeu_miR166i	127,266	52,249	419,710	491,618	542,907	307,162
oeu_miR166j	127,334	52,402	419,709	491,540	542,715	307,163
oeu_miR166k	127,334	52,402	419,709	491,540	542,715	307,163
oeu_miR166l	127,266	52,249	419,710	491,618	542,907	307,162
oeu_miR166m	130,970	55,036	435,737	510,951	560,657	317,213
oeu_miR166n	8,529	6,286	18,521	23,769	21,358	15,080
oeu_miR166o	8,529	6,285	18,521	23,769	21,358	15,080
oeu_miR166p	2	0	3	13	11	10
oeu_miR166q	8,538	6,292	18,512	23,768	21,362	15,078
oeu_miR167a	19	13	359	141	318	412
oeu_miR167b	7	7	349	133	302	404
oeu_miR167c	19	13	360	141	318	412
oeu_miR167d	7	7	349	133	302	404
oeu_miR167e	31	7	204	200	492	427
oeu_miR167f	57	40	18,541	22,195	41,904	26,123
oeu_miR167g	57	40	18,598	22,204	41,915	26,140
oeu_miR168a	22,1292	157,636	163,173	198,720	108,056	95,642
oeu_miR168b	22,1753	158,132	163,292	198,885	108,320	95,878
oeu_miR169a	4	7	2	5	3	13
oeu_miR169b	4	7	2	5	3	13
oeu_miR169c	4	7	2	5	3	13
oeu_miR169d	0	0	48	41	42	59
oeu_miR169e	0	0	48	41	42	59
oeu_miR169f	0	0	50	41	41	59
oeu_miR169g	0	0	48	41	42	59
oeu_miR169h	0	0	48	41	42	59
oeu_miR169i	0	0	0	0	1	1
oeu_miR169j	0	0	0	0	1	1
oeu_miR169k	0	0	0	0	1	1
oeu_miR169l	0	0	0	0	1	1
oeu_miR169m	0	0	0	0	1	1
oeu_miR169r	0	0	0	0	2	10
oeu_miR169s	0	0	48	41	42	59
oeu_miR169v	0	0	0	0	1	1
oeu_miR169w	0	0	0	0	1	1
oeu_miR171a	33	17	12	12	9	12
oeu_miR171b	33	17	12	12	9	12
oeu_miR171c	0	0	4	0	3	3
oeu_miR171d	0	0	4	0	3	3
oeu_miR171e	33	17	12	12	9	11
oeu_miR171f	33	17	12	12	9	11
oeu_miR171g	33	17	13	12	9	12
oeu_miR171h	33	17	13	12	9	12
oeu_miR171i	33	17	12	12	9	11
oeu_miR172a	359	201	5,070	3,060	3,372	2,545
oeu_miR172b	359	202	5,070	3,060	3,373	2,545
oeu_miR172c	359	201	5,070	3,060	3,372	2,545
oeu_miR172d	3	1	6	12	14	9
oeu_miR172e	3	1	6	12	14	9
oeu_miR172f	359	202	5,070	3,060	3,373	2,545
oeu_miR172g	0	0	3	2	1	4
oeu_miR172h	0	0	3	2	1	4
oeu_miR172i	11	6	121	67	89	66
oeu_miR319a	0	0	0	1	2	6
oeu_miR319b	0	0	0	1	2	5
oeu_miR319c	0	0	1	2	2	7
oeu_miR319d	0	0	1	2	2	7
oeu_miR319e	0	0	0	2	0	5
oeu_miR319f	0	0	1	3	0	8
oeu_miR319g	0	0	1	3	0	8
oeu_miR319h	0	0	0	2	0	5
oeu_miR390a	114	109	87	176	105	97
oeu_miR390b	113	108	87	176	105	97
oeu_miR390c	114	109	87	176	105	97
oeu_miR390d	113	108	87	176	105	97
oeu_miR393a	0	7	2	1	2	4
oeu_miR393b	0	7	2	1	2	4
oeu_miR393c	16	57	132	215	341	509
oeu_miR393d	16	57	132	215	341	509
oeu_miR394a-5p	0	0	0	9	5	4
oeu_miR394b-5p	0	0	0	9	5	5
oeu_miR395b	16	1	2	13	96	39
oeu_miR395c	16	1	2	13	96	39
oeu_miR395d	16	1	2	13	96	39
oeu_miR395e	16	1	2	13	96	40
oeu_miR395f	16	1	2	13	96	40
oeu_miR395g	16	1	2	13	96	39
oeu_miR395h	16	1	2	13	96	39
oeu_miR395i	16	1	2	13	96	39
oeu_miR395j	16	1	2	13	96	39
oeu_miR396a	36	36	1,367	786	1,051	832
oeu_miR396b	36	36	1,367	786	1,051	832
oeu_miR396c	39	73	296	171	276	278
oeu_miR396d	39	73	296	171	276	278
oeu_miR396e	39	74	296	172	276	278
oeu_miR396f	0	0	222	61	90	357
oeu_miR396g	0	0	269	95	116	525
oeu_miR397a	6	6	71	163	246	300
oeu_miR397b	0	0	0	1	0	2
oeu_miR398b	0	1	2	9	9	10
oeu_miR398c	0	1	2	9	9	10
oeu_miR399b	0	0	0	0	0	1
oeu_miR399c	0	0	0	0	0	1
oeu_miR399f	0	0	0	0	0	2
oeu_miR399g	0	0	0	0	0	1
oeu_miR399i	9	10	2	7	23	39
oeu_miR403a	121	105	945	968	1,360	1,130
oeu_miR403b	121	105	941	966	1,359	1,125
oeu_miR403c	121	105	941	966	1,357	1,124
oeu_miR408	1	2	5	60	70	97
oeu_miR530a	0	0	0	1	1	0

The analyses of the miRNA in the six libraries exhibited that the majority of the identified miRNAs were either absent or present at lower amount in fruit-derived libraries (UF and RF), in comparison to leaf libraries. Moreover, the comparison to the leaf libraries (NON, JON, NOFF and JOFF). Moreover, the comparison between the miRNA expression levels in the two fruit libraries demonstrated that the unripe fruit library (UF) expressed significantly higher amounts of miRNA than the ripe fruit one (RF). For instance, the expression of miR395 was 4-fold higher in the UF than in the RF (Table 3). The libraries constructed from leaves were also different from each other in relation to their miRNA expression profiles. The evaluation of the November leaf libraries revealed that the "off-year" library (NOFF) possessed higher miRNA expression than the "on-year" one (NON), while particular expression profiles were observed between July leaf libraries (JOFF and JON). Another library-specific pattern was found between two of the "on-year" libraries. More miRNA were counted for the July leaf library (JON) than for the November leaf one (NON) (Table 3 and Figure 4).

**Figure 5:**
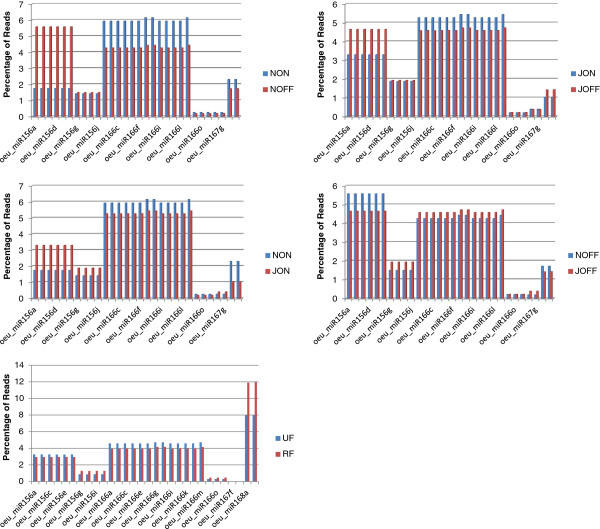
**The most differentially expressed miRNA.** The most differentially expressed known miRNAs were given as five comparisons of the libraries. The expressions were given as percentages.

Most sequences were found at low frequencies in the datasets, which may be due to the unsaturation of the libraries and the broad sRNA variation in the olive tree. However, four miRNA (miR156, miR166, miR167 and miR168) showed very high redundancies in each library. The most abundant miRNA are shown in the Figure 4. The expressions of the miR156, miR159 and miR166 were higher in the leaf libraries than the ones constructed from fruits. The miR159 was expressed at higher levels in the "off-year" NOFF and JOFF datasets than in the "on-year" NON and JON libraries, respectively. However, the expression of the miR156 was similar between the "off-year" NOFF and the "on-year" NON libraries. Showing slight variations, the miR166 was expressed evenly in each leaf library. A significant variation was observed for the miR168 expression between libraries (Table 3 and Figure 5).

### Putative novel miRNA in olive

**Figure 6:**
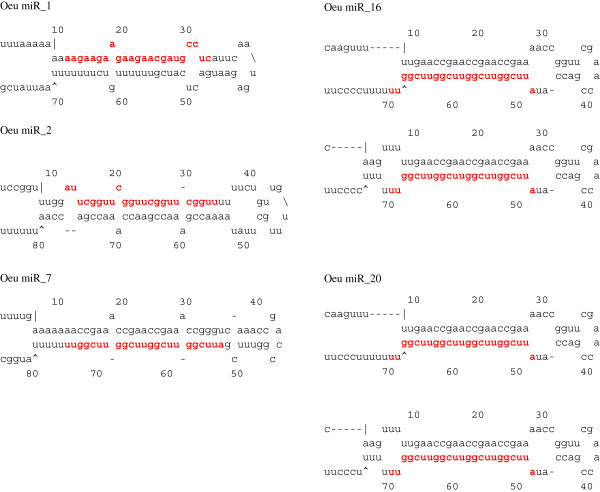
**The most differentially expressed miRNA.** The mature miRNA sequences are shown in red color.

The secondary structure and minimum free-energies were calculated to identify the putative novel miRNA in the olive tree dataset. Based on these predictions, 38 miRNAs were considered as novel (Table 4). Secondary structures of the putative novel miRNA are shown in the Figure 6 and with more detail in Additional file 3. A higher proportion of the sequences was identified at the 5’-end of hairpins than at the 3’-end.

**Table 4:** Identified putative novel miRNA and read counts of the miRNA in the six libraries

**Novel ID**	**Location**	**Strand (+/−)**	**Energy (kcal/mol)**	**Sequence of 5p**	**Sequence of 3p**	**UF**		**RF**		**NON**		**JON**		**NOFF**		**JOFF**	
						**(count)**		**(count)**		**(count)**		**(count)**		**(count)**		**(count)**	
						**5p**	**3p**	**5p**	**3p**	**5p**	**3p**	**5p**	**3p**	**5p**	**3p**	**5p**	**3p**
oeu_mir_1	scaffold_10:17226129:17226207	+	−27.20	AAGAAGAAGAAGAACGAUGCCUC	-	15	-	15	-	-	-	-	-	-	-	-	-
oeu_mir_2	scaffold_10:20223072:20223157	–	−36.30	-	AUUCGGUUCGGUUCGGUUCGGUU	100	117	-	33	32	50	-	45	74	71	53	58
oeu_mir_3	scaffold_14:15500470:15500726	–	−49.71	-	UGUCGACAUAGAAAUGAUUGGC	-	13	-	-	-	-	-	-	-	-	-	-
oeu_mir_4	scaffold_16:5755707:5756007	–	−69.50	-	UCUUUGGAUUGUUUAACAUGGUA	-	10	-	-	-	-	-	-	-	-	-	-
oeu_mir_5	scaffold_16:12916010:12916345	–	−42.40	-	UGAUGAUGAUGAUGACGACGACA	-	14	-	-	-	-	-	-	-	-	-	5
oeu_mir_6	scaffold_19:8213709:8214023	+	−50.83	UCUGAUACCAACUGAUGUGAACC	UCUGAUACCAACUGAUGUGAACC	5	5	-	-	-	-	-	-	-	-	-	-
oeu_mir_7	scaffold_19:15701744:15701856	+	−32.20	UCUGUGUACAAUAAGCCGAUGCU	-	59	-	6	-	-	-	-	-	-	-	-	-
oeu_mir_8	scaffold_1:34545837:34545992	+	−37.10	AGUAGAAGACGCUCUGGUGAG	-	10	-	-	-	-	-	-	-	7	-	-	-
oeu_mir_9	scaffold_1:387325:387591	–	−88.20	UGAUUGAGCCGCGCCAAUAUC	-	51	-	39	-	-	-	-	-	-	-	-	-
oeu_mir_10	scaffold_1:12533361:12533445	–	−25.40	UCGGUUCGGGUUCGGUUCGGUUC	-	16	-	-	-	-	-	-	-	9	-	13	-
oeu_mir_11	scaffold_2:18857579:18857663	+	−34.80	GGUCGGUUCGGUUCGGUUCGGUU	-	10	-	11	-	8	-	5	-	18	-	11	-
oeu_mir_12	scaffold_3:16233511:16233599	–	−31.52	AGAUGGAGAUGGAGAUGGAGAUG	-	7	-	-	-	-	-	-	-	-	-	-	-
oeu_mir_13	scaffold_4:16304187:16304406	+	−44.80	GCUGGAGUAGCUCAGUUGGUU	-	55	-	167	-	64	-	24	-	83	-	36	-
oeu_mir_14	scaffold_5:13476318:13476427	+	−40.21	-	GAGGGGGAGUGUUGGCGUGAG	-	22	-	-	-	-	-	-	-	-	-	-
oeu_mir_15	scaffold_7:6284292:6284592	–	−46.40	UGUUGUUGUUGUUGUCGUCGUCA	-	13	-	-	-	-	-	12	-	-	-	-	-
oeu_mir_16	scaffold_11:6066546:6066885	+	−81.90	-	UCCGUUGUAGUCUAGUUGGUU	-	-	-	132	-	-	-	-	-	-	-	-
oeu_mir_17	scaffold_12:2050403:2050549	–	−60.20	UCUUGCUCAAAUGAGUAUUCCA	-	-	-	12	5	86	36	-	-	12	12	-	-
oeu_mir_18	scaffold_1362:5405:5632	–	−48.92	-	GUUGUAGACAUGAAAGCGUAAGA	-	-	-	28	-	-	-	-	-	-	-	-
oeu_mir_19	scaffold_14:13968161:13968276	+	−32.40	-	GUUUGGUUCGGUUCGGUUCGGUU	-	-	-	28	-	14	-	18	-	25	-	16
oeu_mir_20	scaffold_19:13198720:13199053	+	−107.50	-	UGGUGGUGGUGGUGGUGGUGACA	-	-	-	12	-	-	-	-	-	-	-	-
oeu_mir_21	scaffold_1:30807164:30807488	-	−87.40	-	AAUAUUUUUGAUCUUUUGGAU	-	-	-	7	-	8	-	-	-	-	-	-
oeu_mir_22	scaffold_5:4584254:4584466	+	−39.20	-	UGUCUGGACCAGUUUACGUGC	-	-	-	12	-	-	-	-	-	11	-	-
oeu_mir_23	scaffold_136:423:565	+	−38.90	-	GUUCGGUUCGGUUCAGUUCGGUU	-	-	-	-	7	10	-	-	-	-	-	-
oeu_mir_24	scaffold_13:1053462:1053647	+	−46.84	GAAGCUAUGAGAUCUGAGGG	-	-	-	-	-	12	-	-	-	-	-	-	-
oeu_mir_25	scaffold_16:12933922:12934079	–	−56.90	UGGGGAAGACAGGCACAUGAA	-	-	-	-	-	11	-	22	-	11	-	16	-
oeu_mir_26	scaffold_18:1471946:1472262	–	−66.60	-	GGGGGAGGGGGAGAGAGAGAGAG	-	-	-	-	-	5	-	-	-	-	-	-
oeu_mir_27	scaffold_1:29385469:29385555	–	−37.30	UGGCGGUGGCGGUGGCGGUGGU	-	-	-	-	-	7	-	6	-	5	-	-	-
oeu_mir_28	scaffold_1:31083890:31083975	–	−35.80	-	GAUGGUGGGGUUGUGGGUGGC	-	-	-	-	-	26	-	-	-	22	-	-
oeu_mir_29	scaffold_1:34445830:34445942	–	−27.10	UUGACAGAAGAUGGAGAGCAC	-	-	-	-	-	17	-	54	-	32	-	34	-
oeu_mir_30	scaffold_204:28760:28841	–	−32.55	GUUCGGUUCGGUUCUGUUCGGUU	-	-	-	-	-	12	-	-	-	-	-	-	-
oeu_mir_31	scaffold_3:15576344:15576447	–	−51.20	UUCCACGGCUUUCUUGAACUUC	-	-	-	-	-	194	-	212	-	88		588	-
oeu_mir_32	scaffold_4:14314575:14314936	–	−48.55	-	UUUAAAGAGAUUGUUGUAAUU	-	-	-	-	-	22	-	8	-	-	-	-
oeu_mir_33	scaffold_16:2089550:2089717	+	−34.00	AAAUUGGUCCGGACACCCAUA	-	-	-	-	-	-	-	8	-	-	-	6	-
oeu_mir_34	scaffold_4:13969966:13970214	+	−57.51	UGGAGGUGGAGGUGGCGGUGG	-	-	-	-	-	-	-	20	-	-	-	-	-
oeu_mir_35	scaffold_1:42369615:42369709	–	−23.90	-	GUUCAGUUCGGUUCGGUUCGGUU	-	-	-	-	-	-	-	-	-	13	-	-
oeu_mir_36	scaffold_13:1053462:1053647	+	−46.84	GAAGCUAUGAGAUCUGAGGGC	-	-	-	-	-	-	-	-	-	-	-	10	-
oeu_mir_37	scaffold_16:11530564:11530665	–	−26.60	AGGGGAGGGAGAGAGAGAGAGAG	-	-	-	-	-	-	-	-	-	-	-	6	5
oeu_mir_38	scaffold_4:14079438:14079535	–	−39.30	UCUCGGUUCGGUUCGGUUCGGUU	-	-	-	-	-	-	-	-	-	-	-	13	-

**Figure 7:**
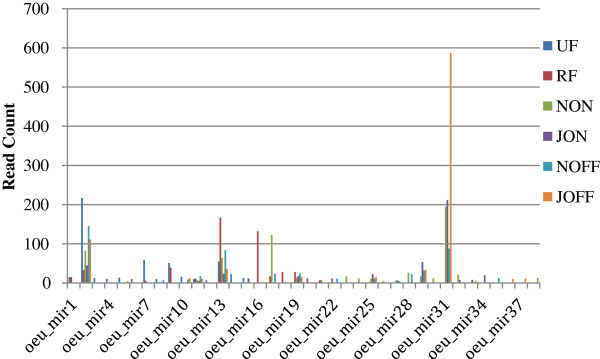
**The most differentially expressed putative novel miRNA.** The histograms show the most differentially expressed putative novel miRNA for the six libraries. The expressions were shown as read counts.

With an average of 15 read counts, the putative novel miRNA showed lower expression levels than the miRNA. Some of the putative novel miRNA showed particular expression profiles (Table 4). Thus, 10 were expressed only in libraries created from fruits, and missing in leaf ones. Some of them (miR3, miR4, miR6, miR12 and miR14) were detected only in the unripe fruit library (UF), while the miR16 and miR20 were found in the ripe fruit library (RF). Although the miR7 was counted in both fruit-derived libraries, its expression was three-fold higher in the UF than in the RF dataset. The miR1 and miR9 had similar expression levels in both libraries (Table 4 and Figure 7).

On the contrary, 16 putative novel miRNAs were specifically detected in leaf libraries, and they showed different expression patterns (Figure 7). Additionally the miRNA* sequences of the putative novel miRNAs were also identified (Additional file 2). Some of them were found to be associated with leaf developmental stages. The miR35 was detected just in the NOFF library, whereas the miRNA28 was present only in both of the November leaf libraries (NON and NOFF). The miRNA33 showed the opposite pattern, being expressed only in July leaf libraries (JON and JOFF). Others (miR23, miR24, miR26 and miR30) were found exclusively in the NON library. On the other hand, some of them (miR36, miR37 and miR38) were only expressed in the "off-year" July library (JOFF). Others (miR32 and miR34) were detected solely in the "on-year" July library (JON), whereas the miR31 was identified in both on- and "off-year" libraries. The rest three (miR25, miR27 and miR29) were expressed with no particular pattern among the leaf libraries.

### Target transcript analyses of olive miRNAs

To obtain a further insight view of the mechanisms underlying the alternate bearing in the olive tree, both the Gene Ontology (GO) <http://www.geneontology.org> and the Kyoto Encyclopedia of Genes and Genomes (KEGG) <http://www.genome.jp/kegg> pathway analyses were performed on the obtained datasets. Thus, different targets of miRNA were predicted as described by Allen et al. [28]. A total of 1,616 and 1,288 mRNA were predicted as targets of 135 known and 32 putative novel miRNA, respectively (see detailed information in Additional file 4). The gene ontology analyses of biological function and molecular processes of miRNA targets are summarized in Table 5. In most cases, single genes were targeted by several miRNA (eg., the gene POPTR_0003s18900.1|PACid:18216192 was targeted by both miR159 and miR319). Yet, in some cases, multiple targets were regulated by a single miRNA (eg., the miR167e targets 15 genes). Thus, a total of 280 groups were identified on the basis of their functional similarities. More than 80% of the targets showed binding function, and the following cluster contained genes with oxidoreductase activity. The most significantly abundant groups (p-value <0.05) are shown in detail in the Additional file 5.

**Table 5:** Summary of the gene-ontology analyses of the biological functions and molecular processes related to the miRNA target genes

**miRNA**	**Total targets**	**Individual targets**	**GO biological processes**	**GO molecular functions**
oeu_miR156 a/b/c/d/e/f/g/h/i/j/k	306	50	Biological regulation, metabolic process, cellular process, signal transduction, response to stimulus, response to stress, organismal development, transport, localization, reproductive developmental process and phyllome development.	Binding, oxidoreductase activity, kinase activity, transferase activity, catalytic activity, phosphatase regulator activity and enzyme regulator activity.
oeu_miR159 a/b/c/d	65	19	Organismal development, reproductive developmental process, biological regulation, developmental process, cellular response to hormone stimulus, metabolic process, biological regulation and transcription.	Binding, oxidoreductase activity, catalytic activity, hydrolase activity and ligase activity.
oeu_miR160 a/b/c/d/e/f/g	45	10	biological regulation, metabolic process, transcription, response to stimulus, organismal development and reproductive development.	Binding.
oeu_miR164 a/b/c/d/e/f	46	12	Biological regulation, metabolic process, transcription, response to stimulus, organismal development, reproductive developmental process, transport, localization and phyllome development.	Binding, oxidoreductase activity, kinase activity, transferase activity, catalytic activity and hydrolase activity.
oeu_miR166 a/b/c/d/e/f/g/h/i/j/k/l/m/n/o/p/q	159	16	Organismal development, reproductive developmental process, metabolic process, biological regulation.	Binding, transporter activity, oxidoreductase activity, protease activity, peptidase activity, catalytic activity and hydrolase activity.
oeu_miR167 a/b/c/d/e/f/g	83	25	Organismal development, biological regulation and metabolic process.	Binding, oxidoreductase activity, catalytic activity, kinase activity, transferase activity, phosphatase activity, hydrolase activity and unannotated.
oeu_miR168 a/b	14	7	Organismal development and biological regulation.	Binding.
oeu_miR169 a/b/c/d/e/f/g/h/i/j/k/l/m/r/s/v/w	84	18	Transport and localization.	Binding and transporter activity.
oeu_miR171 a/b/c/d/e/f/g/h/i	96	24	Organismal development, metabolic process and gene expression.	Binding, oxidoreductase activity, catalytic activity and transporter activity.
oeu_miR172 a/b/c/d/e/f/g/h/i	153	30	Organismal development, reproductive developmental process, metabolic process, transport, localization, biological regulation and transcription.	Binding, oxidoreductase activity, signal transducer activity, hydrolase, transporter activity and protein kinase activity.
oeu_miR319 a/b/c/d/e/f/g/h	128	22	Organismal development, reproductive developmental process and transcription.	Binding.
oeu_miR390 a/b/c/d	84	21	Biological regulation, metabolic process, organismal development and inorganic anion transport.	Binding, oxidoreductase activity, phosphotransferase activity and protein kinase activity.
oeu_miR393 a/b	12	12	Organismal development, reproductive developmental process, flower development, metabolic process, response to stimulus, response to stress, transport and localization.	Binding, phosphotransferase activity and protein kinase activity.
oeu_miR394 a/b	30	15	–	–
oeu_miR395 b/c/d/e/f/g/h/i/j	72	8	Metabolic process, response to chemical stimulus and ion transport.	Binding, catalytic activity, transporter activity and adenylyltransferase activity.
oeu_miR396 a/b/c/d/e/f	98	34	Biological regulation, negative regulation of molecular function, metabolic process, organismal development, reproductive developmental process and flower development.	Binding, catalytic activity, hydrolase activity and methyltransferase activity.
oeu_miR397 a/b	59	35	Organismal development, reproductive developmental process, lignin metabolic process and biological regulation. <−−missing something at the end??	Binding, oxidoreductase activity and signal transducer activity.
oeu_miR399 b/c/f/g/i	18	7	Transport and localization.	Transporter activity.
oeu_miR408	13	13	Lignin metabolic process, unannotated and biological regulation.	Binding, oxidoreductase activity and transferase activity.
oeu_miR530 a	39	39	–	–

The identified target genes are involved in a broad range of biological processes. A total of 341 GO terms from the process ontology were identified. Among them, 108 groups were found with significant abundance (p-value <0.05), the majority of which were associated with metabolic processes (>70%) followed by developmental processes (33%) and biological regulation (30%). The GO terms for all of the 108 groups are shown in the Additional file 6. Some targets were involved in many other general processes such as transcription, reproduction and anatomical structure maintenance. Approximately, 1% of the targets could not be annotated. About 19%, 12% and 6% of the targets were clustered in groups of organ development, hormone-mediated signaling and flower-development processes, respectively, which are thought to be associated with alternate bearing. For example, the target of miR166 is associated with flower development, and interestingly, it was more upregulated in the unripe fruit (UF) library than in the ripe fruit dataset (RF). Similarly, the phyllome development-responsible gene was targeted by the miR164, which exhibited a lower count in the fruit- than in the leaf-derived library. Additionally, the predicted targets of the putative novel miRNA were also analyzed to better understand their physiological roles. However, all of them were found to encode unidentified proteins. The GO terms for all of the 108 groups are shown in the Additional file 6.

A total of 130 KEGG pathways were enriched (see Additional file 7), of which seven main groups were detected at significantly high abundance level (p-value <0.05), as shown in Table 6. The majority of the targets are involved in the ascorbate and aldarate metabolism, as well as hormone-signal transduction pathways. A few percentage of targets corresponded to the biosynthesis of the brassinosteroids, which regulate the growth and development in plants.

**Table 6:** Pathway annotations of genes targeted by miRNAs

		**UF**		**RF**		**NON**		**JON**		**NOFF**		**YT**	
**Pathway**	**Pathway ID**	**p-value**	**q-value**	**p-value**	**q-value**	**p-value**	**q-value**	**p-value**	**q-value**	**p-value**	**q-value**	**p-value**	**q-value**
Ascorbate and aldarate metabolism	ko00053	1.46E-34	1.75E-32	8.73E-35	9.87E-33	4.65E-34	5.35E-32	9.92E-32	1.24E-29	5.72E-32	7.20E-30	1.38E-32	1.69E-30
Plant hormone signal transduction	ko04075	1.12E-06	6.71E-05	7.59E-07	4.29E-05	3.81E-07	2.19E-05	2.74E-07	1.71E-05	1.67E-07	1.05E-05	2.16E-06	1.33E-04
Brassinosteroid biosynthesis	ko00905	7.05E-06	2.82E-04	6.48E-06	2.44E-04	8.54E-06	3.27E-04	2.08E-05	8.65E-04	1.90E-05	7.96E-04	1.50E-05	6.13E-04

### Sequencing read validation and target transcript identification

**Figure 8:**
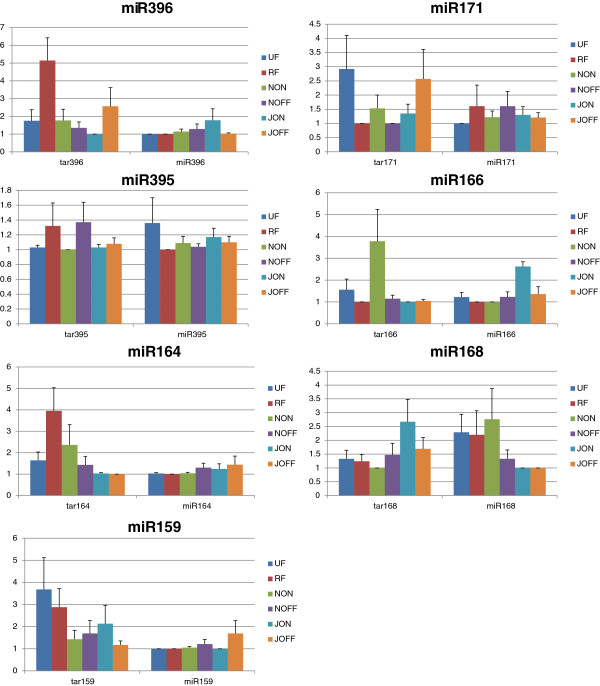
**qRT-PCR validation of selected miRNA and their target genes.** The histograms show the relative values for the quantified expressions of seven miRNA and their target genes in six olive tree samples. The analyses were performed as triplicates, and the error bars indicate the standard deviations.

In order to confirm the results of the Illumina sequencing and quantify the expression patterns of both the miRNA and their target transcripts, nine miRNA-target gene pairs were arbitrarily selected for further analyses by qRT-PCR. All six sample sets (UF, RF, JON, NON, JOFF and NOFF) were used for validation and expression measurement of conserved olive tree miRNA (miR156, miR159, miR164, miR166, miR168, miR171, miR172, miR395 and miR396) (see Additional file 8). The quantification of the identified olive tree miRNA and miRNA target transcript expression levels are comparatively showed in the Figure 8. Of the nine studied miRNA, only two (miR156 and miR172) were not detected via qRT-PCR. miR395 was detected as upregulated in the unripe fruit, as compared to the ripe fruit tissue, while the standart deviation of miR395 was not low enough to fully validate those expression differences. The qRT-PCR experiments also validated the deep-sequencing results of upregulation in the JON as compared to the JOFF for miR166 and formiR396. The latter had high standart deviation; however expression level of target showed a clear difference between JON and JOFF indicating relable expression difference between the samples. Downregulation in the JON as compared to the JOFF was also confirmed for miR159 and miR164 by qRT-PCR. Read counts of miR159, miR164, and miR166 revealed that there were a slight upregulation in the NON tissue as compared to the NOFF. Similarly, qRT-PCR data showed the minor expression differences. The obtained miR166, miR395 and miR396 expressions were shown as upregulated in the JON as compared to the NON tissue. On the other hand, the expression measurement of the JOFF versus the NOFF showed the upregulation in miR159, miR164. Congruent with that the miRNA-transcript target quantifications detected the expected correlations between the miRNA and the target gene expression levels. Expression of their target genes were downregulated in JOFF as comparered to NOFF (Table 3 and Figure 8). Additionally, the target transcript of the miR164 was measured as upregulated in RF compared to UF, validating the miRNA deep-sequencing results, while it was not verified with miRNA expression by qRT-PCR. The expression difference of miR396 between NON and JON correlated with expression of its target. Similarly, miR168 was detected as upregulated in the NON compared to the JON, and the target transcripts of these miRNA were measured as downregulated (Table 3, Figure 8).

## Discussion

The olive tree is known for its irregular crop production from year to year [2]. Although different plant species exhibit such phenomenon, the physiological processes involved are not universal, but rather species-specific [29]. However, it has become clear that the genetic networks triggering the developmental phase-transitions share some common factors among species. Thus, Ulger et al. [30] reported that some endogenous plant growth hormones like the abscisic acid (ABA), gibberellins like the gibberellic acid (GA_3_) and auxins like the indole-3-acetic acid (IAA) have important roles on the alternate bearing. Additionally, the stored carbohydrate amounts in the plants differ between the on- and off-years, and consequently, an association between carbohydrate mobilization and biennial bearing was proposed [31].

The olive tree has a natural tendency to produce high number of flowers and fruits. Yet, the developing seeds inside the fruits produce molecular messengers (like the gibberellins) that inhibit the floral induction, arresting the buds and directing them towards shoots or leaves (vegetative buds) instead of flowers (reproductive buds). Likewise, the growing of a large number of fruits may produce the depletion of the carbohydrate reserves in the tree. Therefore, different strategies can be used to reduce or even eliminate the alternate bearing in the olive tree: i) reduction of the number of fruits, by means of a proper pruning the year before the expected high production; ii) reduction of the density of the fruits at a very early stage of development (very small fruits); iii) early harvest of the developed fruits (large green fruits; even though the flowering inhibition has started at such stage, this strategy has some influence); and iv) prevention of the depletion of the carbohydrate reserves in the tree (eg, supplying a convenient irrigation to favor the plant nourishment and reserve accumulation). These facts indicate that the periodicity in bearing is indeed an adaptation mechanism of the plant to avoid excessive depletion of its reserves, to assure that part of them are devoted to a significant vegetative growth (leaf and shoot buds), and a defensive mechanism to cope with restrictive environmental conditions like drought and macronutrient/micronutrient deficiencies [32].

So far, several miRNA have been identified and characterized in plants, and their roles in diverse biological processes have been documented in many instances. They are involved in many physiological processes, like development and stress responses [9,16,33]. The study of miRNA with traditional methods may be complicated, but the recent high-throughput sequencing methodologies have become an excellent approach to discover miRNA in different organisms [34-36].

In this study, we sequenced and assessed the sRNA of six olive libraries constructed from two organs, each at different developmental stages, using the Illumina high-throughput system to identify a comprehensive set of alternate bearing-associated miRNAs in *O. europaea* L. With a total of 93,526,915 sequence reads, these libraries represent, to our knowledge, the deepest olive tree miRNA sampling to date. Thus, it is about 550 times higher than the one produced by Donaire et al. [36], which is the only olive tree miRNA report previously published to date. In that study, two libraries were constructed (juvenile and mature shoots), and 18 families of known and five putative miRNA were identified in *O. europaea* L. In addition to those, we have discovered four more conserved miRNAs within 22 families.

The high-abundance miRNA (except the miR156a, miR156b, miR156c and miR156d) were generally ubiquitously expressed in the six libraries, suggesting a correlation between the abundance of miRNA and their expression levels. Supporting our data, evolutionarily conserved miRNA have been found often among the most abundant miRNA by other studies [37]. The miR156 was previously identified in adult olive tree shoots [24]. However, in that study, the miR156 had a very low count. Moreover, the miR156 regulates the expression of the Squamosa Promoter-binding protein-Like (*spl*) gene, which plays a role in the juvenile-to-adult and annual phase transitions [12,13,24]. Shalom et al. [38] analyzed the expression of the miR156 in "on" and "off" citrus trees, and no significant differences were found between samples. They also analyzed the expression of the *spl* gene, and detected upregulation in "off" year trees, as compared to "on" year trees. Supporting that, we found that the expression of the miR156 (which is a negative regulator of the *spl* gene) in "on" year juvenile olive tree sample pool (JON) was higher than in the "on" year mature one (NON).

The expression level of several miRNA varied between the six analyzed libraries, indicating a differential functional role of the involved genes in the development-associated regulation. The miR395 that we have found was also detected in the previous study on the olive tree [24], which targets transport and response-to-stimulus genes, being four-fold upregulated in the unripe fruit library (UF) as compared to the ripe fruit one (RF). Its expression was also higher in July (JON-JOFF) than in November leaves (NON-NOFF). The nutritional control has been considered as one of the principal regulatory mechanisms for alternate bearing [29]. Yet, contradictory results have been reported on the involvement of the carbohydrate reserves on the reproductive development in the olive tree [30,31,39,40]. Recently, Bustan et al. [2] showed that the demand on carbohydrates by the developing fruits is significant; yet the carbohydrate deficiency was not an essential controller for the alternate bearing. In addition, deficient boron transport was proposed as an important factor in the alternate bearing in avocado [41]. Previously, it was reported that the miR395 might be involved in the salt-induced response pathways, such as the maintenance of the energy supply in maize [42]. In the view of the previous reports and the present study, we conclude that the transport of nutrients (including ions) is involved in the development and ripening, with a significant role of the miR395 on the alternate bearing in such species.

The count of known miRNA in the leaf-derived libraries was higher than that of fruit-originated ones. Moreover, a higher diversification of miRNA was also found in the leaf libraries. These results indicate that more miRNA are involved in the leaf than in the fruit physiology. This is not surprising, taking into account that the latter represents a specific organ, whereas the former is a more general-purpose organ. Supporting our data, the contribution of leaves to the reproductive growth was found to be two-fold [3].

The miR156 and miR166 are the two most abundant conserved miRNA in our analyses, being also found in large amounts in other studies [31]. The six libraries exhibited significant differential expression of the miR156 members. Their expression decreased approximately 2.5-fold in NON as compared to NOFF. The assessment of the “on-year” July (JON) and November (NON) leaf libraries showed that the miR156 expression was downregulated in the July (JON) leaf library, indicating the specificity of the miR156 for the November leaf stage. Previous studies also revealed that, besides the miR172, the miR156 also regulates the Squamosa Promoter-binding protein-Like (SPL) transcription factors, controlling the maintenance of the juvenile phase and the timing of the juvenile-to-adult phase transition in both *Arabidopsis thaliana* and olive tree [12,13,24]. The miR172 is considered one of the most ancient miRNA families in plants, being shown to function in regulating the transition between developmental stages [10,14]. In our study, the expression of the miR172 members was clearly higher in the leaf libraries than in the fruit datasets, supporting their activity in the maintenance of juvenile stage. The higher expression of two miR172 members (miR172d and miR172e) in NOFF than in NON also supports this conclusion. Additionally, a similar pattern was observed for the miR172g and miR172h expression of July leaf libraries (JOFF and JON). Moreover, the July leaves (JON) expressed significantly more miR172d and miR172e than the November ones (NON). Except for miR172d, miR172e, miR172g and miR172h, the rest were ubiquitously expressed in the leaf libraries. Therefore, we conclude that the miR172 plays not the main, but a significant role in the onset of the developmental phase-transition in the olive tree.

The hormonal control has been proposed as one of the major regulatory mechanisms of alternate bearing [28]. To give an example, members of the miR160 and miR319 (targeting the genes involved in the hormone-mediated signaling) were significantly regulated by bearing on the olive tree. The expression difference of the miR160 members between the on- and off-year varied from 4- to 6-fold, while its expression was slightly upregulated in juvenile leaves compared to mature leaves which was also detected in olive shoots [24]. Whereas the difference between on- and off-years was about 1.8-fold for the miR319. Besides, significant fold changes were observed between the fruit libraries. Similarly, the fruit libraries showed considerable expression differences of the miR393 (which targets genes responsible for hormone-mediated signaling), indicating an important contribution of the hormone-mediated response on the olive tree development processes.

The analyses of the miRNA also revealed that a number of targets are related to functions which may be associated to alternate bearing, as in the case for floral development and organ morphogenesis. The comparison of the expression of the miR168 and miR396 between the on- and “off-year” libraries revealed small but significant differences. The effect of the miR396 on the leaf development via the targeting growth-regulating factors had been previously shown [43]. Additionally, the miR166 and miR171 targeting genes involved in organ morphogenesis and developmental processes showed a fruit library-specific regulation. Thus, their expression was significantly upregulated in the unripe fruit (UF) library. It may be speculated that the suppression of these target genes may inhibit the maturation of fruits, albeit such hypothesis needs further investigation.

The KEGG analyses revealed that the genes targeted by the olive tree miRNA are largely involved in the carbohydrate metabolism and hormone signal-transduction pathways, followed by the brassinosteroid biosynthesis pathway. As discussed above, the nutrition and hormone control have been proposed as two principal factors involved in the alternate bearing. Besides, the brassinosteroids belong to the plant steroid hormone group that regulates the growth and development [44]. Thus, our data further support that the nutritional and hormone control play essential roles in the alternate bearing for the olive tree.

In this study, in addition to the 135 conserved miRNA, we have also identified 38 putative novel miRNA in the olive tree, which exhibit the diversity of the miRNA expression in *O. europaea* L. and show the occurrence of more miRNA than previously known. This genome-wide survey of six olive tree miRNA libraries has allowed to discover the miRNA expression profiles associated to alternate bearing, and consequently can contribute to elucidate the processes involved in this relevant physiological phenomenon. These results allow to conclude that not just a few, but many genes, may be involved in the alternate bearing in the olive tree. Thus, the differentially-expressed miRNA identified in this study will be a valuable source for further assessments of alternate bearing-associated genes in such and other species.

### Concluding remarks

A comprehensive study on the olive tree miRNA related to the alternate bearing was performed. The miRNA of six olive libraries constructed from fruits (ripe and unripe) and leaves ("on-year" and "off-year" in July and in November, respectively) were subjected to Illumina deep-sequencing. The bioinformatics analyses of 93,526,915 reads identified 135 conserved miRNA belonging to 22 families, with 38 putative novel miRNA for the olive tree datasets. The expression of the miRNA varied greatly between the six libraries, indicating the contribution of diverse miRNAs in the balancing between the reproductive and the vegetative developments. The predicted targets of the miRNAs were categorized into 108 process-ontology groups with significant abundance. Among those, some potential alternate bearing-associated processes were found, such as development, hormone-mediated signaling and organ morphogenesis. The KEGG analyses indicated that the nutritional and hormonal control play essential roles in the alternate bearing of the olive tree. The regulation of the miRNAs under different developmental stages indicated that the nutritional and hormonal controls, together with the ones for the flowering processes had noteworthy impacts on the alternate bearing of *O. europaea* L. Our results provide significant data on the miRNA-dependent developmental phase transition interaction and advance perspectives in the miRNA profiling of the olive tree that may be also useful for other species.

## Methods

### Sample collection

Olive (*Olea europaea* L. cv. Ayvalik) leaf samples from the Edremit Olive Seedling Growing Station were collected directly into liquid nitrogen from “on year” leaves and from “off year” leaves in July and from “on year” leaves and from “off year” leaves in November. Unripe and ripe fruits were also collected the same way in November and stored in an −80°C freezer until use. The total RNA extractions from the olive tree leaves and fruits were performed using the RNeasy Plant Mini Kit (Qiagen, Hilden, Germany) following the manufacturer’s instructions. The "on year" trees and the "off year" trees were approximately four meters apart from each other and they were not shading one another.

### Small RNA library construction and sequencing

Six sample sets were prepared: unripe fruit (UF), ripe fruit (RF), “on-year” November leaf (NON), “on-year” July leaf (JON), “off-year” November leaf (NOFF) and “off-year” July leaf (JOFF). Total RNA extractions from olive leaves and fruits were performed using RNeasy Plant Mini Kit (Qiagen, Germany) and TriPure (Roche, Germany) following manufacturer instructions for each library set, the RNA samples from leaves and fruits of the olive trees were used as a single RNA pool. The construction of the sRNA libraries, cluster generation and deep-sequencing were carried out by the Beijing Genomics Institute (BGI) (Hong Kong). Briefly, the isolated total RNA of each sample was fractionated on 15% denaturing polyacrylamide gel for size selection. The small RNAs (18 to 30 nt) were then ligated to a pair of Solexa adapters at the 5’- and 3’-ends using the T4 RNA ligase. The ligated products were selected by size-fractionation and purified from gel. Then, adapter-ligated fractions were amplified for 15-cycles with a pair of adapter-complementary primers to produce sequencing libraries. The purified PCR products were directly sequenced with the HiSeq 2000 Sequencing System (Illumina, San Diego, CA, USA) according to the manufacturer’s protocol.

### Bioinformatics analyses and putative novel miRNA prediction

The sequence tags from the HiSeq sequencing were processed by a data-cleaning pipeline to get rid of the low-quality and too-small tags, as well as the adapter sequences from the tags. The high-quality sRNA reads were searched using the National Center for Biotechnology Information (NCBI) GenBank and Rfam databases [45]. The clean tags were annotated into the appropriate non-coding RNA categories, such as rRNA, tRNA, snRNA or snoRNA, or either being considered as degradation fragments of mRNA (and thus, discarded).

In order to identify known miRNAs in the olive tree, the remaining unique small RNA sequences were subjected to BLASTn searches using the miRBase database version 18.0 [46]. The near perfectly matched (up to two mismatched) sequences were considered to be known miRNA [47]. Those which could not be annotated were used to predict putative novel miRNA by the software MicroRNA Discovery By Deep Sequencing (Mireap) <http://sourceforge.net/projects/mireap>, developed by the Beijing Genome Institute (BGI, China).

To identify putative novel miRNAs, the small RNA tags that matched the miRBase and Rfam databases were filtered and the remaining tags were aligned with the *Populus trichocarpa* genome using the SOAP 2.0 program [23], due to absence of a reference genome for the olive tree. Olive database a collection of *Olea europaea* L. EST reads is available online (http://www.oleadb.it/). However, the sequences in database have not been fully annotated. In this paper, we aimed to identify conserved and as well as new miRNAs. Since the whole genome sequence is missing, there is a possibility of misinterpreting the results especially for identification of novel miRNAs in case of using sequences olive database. Blast analysis for the sequences assigned as putative novel miRNA was performed against the sequences in olive database, and any hit was detected; whereas they were aligned against poplar genome. Since these miRNAs could not be mapped to genomic locus on olive chromosomes, we referred them as "putative novel miRNAs". And the olive miRNAs were validated in poplar genome which was fully sequenced. The secondary structures of the matched sequences were analyzed by the Mireap program. The structures that met previously described criteria [28] were considered as putative novel miRNA candidates. The secondary structures of the putative novel miRNA were folded again using Mfold 3.2 [48]. The match alignment score thresholds between the candidate olive tree and the known plant miRNA were set to 4.5 for conserved and to 5.0 for putative novel miRNA, and annotated defined by Meyers et al. [47].

The frequency of miRNA read counts was normalized as transcripts per million (TPM) and normalization of miRNA expression levels between six olive libraries was carried out based on the following formula [49]:

Normalization formula: ActualmiRNAcountTotalcountofcleanreads×106

The normalized read counts for each miRNA in six libraries were given in Table 3.

The data produced in this work have been deposited in NCBI’s Gene Expression Omnibus and are accessible through GEO Series accession number GSE42978.

### miRNA quantification by real time RT-PCR

A volume of one μl of total RNA (1000 ng) was used for the miRNA stem-loop reverse transcription reaction in a total volume of 10 μl, containing 0.5 μl of the 10 mM dNTP mix, one μl of the stem-loop reverse-transcription (RT) primer (1 μM) and 7.5 μl of nuclease-free water. The mix was incubated at 65°C for 5 min and then put on ice for 2 min. After that, 4 μl of first-strand buffer (5X), 2 μl of 0.1 M dithiothreitol (DTT), 0.1 of μl of RNAseOUT (40 units/μl) from Invitrogen (Carlsbad, CA, USA) and 0.25 μl of SuperScript III (200 U/μl) from the same manufacturer were added into each tube, and the RT reaction was performed as follows: 30 min at 16°C; and 60 RT cycles (30°C for 30 s, 42°C for 30 s and 50°C for 1 s). The RT reactions were terminated at 85°C for 5 min. During the cDNA synthesis for the microRNA quantification, control reaction tubes including all components without either the RT primer (no-RT) or the RNA template (no-RNA) were also prepared [19-21,50].

The real-time RT-PCR was carried out using a SYBR Green I Master mix from Roche on the LightCycler 480 II Real-Time PCR from Roche Applied Science. By using the previously synthesized 2 μl RT stem-looped cDNA products, the quantitative PCR reactions were performed in a reaction mix containing 10 μl of the 2X Master mix, 1 μl of the forward (10 pmol) and 1 μl of the reverse (10 pmol) primers, 0.3 μl (30 nM) of reference dye and 7.7 μl of nuclease-free water. Specifically-designed forward primers for each individual miRNA as well as the universal reverse-primer (5’-GTGCAGGGTCCGAGGT-3’) [32,48] were used for quantifications. The qRT-PCR conditions were setup as follows: 95°C for 15 min, followed by 40 cycles of 95°C for 5 s, 56°C for 10 s and 72°C for 30 s. All PCR products were denatured at 95°C and cooled to 65°C, and the fluorescence signals were accumulated consistently from 65°C to 95°C as the temperature increased at 0.2°C per second. The reactions were repeated at least three times for sound statistical analyses.

### Target prediction and annotation

The putative mature miRNA sequences were aligned with the Expressed Sequence Tags (EST) database to predict putative miRNA targets. The resulting alignments should match the criteria suggested by Allen at al [28] and Schwab et al. [51] to be considered genuine. In short, the criteria used were as follows: i) no more than four mismatches between the sRNA and the target (G-U bases count as 0.5 mismatches); ii) no more than two adjacent mismatches in the miRNA/target duplex; iii) no adjacent mismatches in in positions 2–12 of the miRNA/target duplex (5’ of the miRNA); iv) no mismatches in positions 10–11 of miRNA/target duplex; v) no more than 2.5 mismatches in positions 1–12 of the of the miRNA/target duplex (5’ of the miRNA); and vi) the Minimum Free Energy (MFE) of the miRNA/target duplex should be ≥75% of the MFE of the miRNA bound to its perfect complement.

### qRT-PCR validation of predicted target genes

To validate and detect the expression levels of the predicted miRNA target genes related to the olive miRNA, qRT-PCR were performed with nine miRNA-target gene pairs. The target transcripts of miR156, miR159, miR164, miR166, miR168, miR171, miR172, miR395 and miR396 (see Additional file 8) were obtained using the Plant Small RNA Target Analysis Server (psRNATarget) <http://plantgrn.noble.org/psRNATarget> (selecting the “User-submitted small RNA/user-submitted transcripts” tab) [52] and the BlastN algorithm <http://blast.ncbi.nlm.nih.gov/Blast.cgi?PROGRAM=blastn>. Specific PCR primers were designed using the online Primer3Plus software <http://www.bioinformatics.nl/cgi-bin/primer3plus/primer3plus.cgi>[53]. Specific PCR primers were designed using online Primer3Plus software [32]. Firstly, 1.5 μg of total RNA were retrotranscribed into cDNA using the Superscript III First-Strand Synthesis System from Invitrogen, according to the manufacturer’s instructions. In brief, the qPCR was performed in a 96-well plate instrument (LightCycler 480 Instrument II) and in 20 μl reactions that contained one μl of the cDNA, 10 nM of each specific forward and reverse primers, and FastStart SYBR Green I master mix. Each experiment was run in triplicate for each gene and the relative quantities of the target transcripts were calculated based on the *18s rRNA* (forward primer: 5’-GTGACGGGTGACGGAGAATT-3’; reverse primer: 5’-GACACTAATGCGCCCGGTAT-3’) as a normalizer housekeeping gene. The qRT-PCR conditions were as follows: preheating for 10 min at 95°C; and 40 cycles (95°C for 30 s, 58°C for 1 min and 72°C for 10 min). To filter the false-positive peaks, the melting curves of the real-time PCR results were analyzed for each run and the data of the fluorescence signals were obtained from 58°C to 95°C, as the temperature increased at 0.5°C per second [16,19,49].

### GO-enrichment and KEGG pathway analyses

In order to understand the miRNA target function, a GO enrichment analysis was performed on the predicted target-gene candidates. The BLASTx <http://blast.ncbi.nlm.nih.gov/Blast.cgi?PROGRAM=blastx> was carried out using the target sequences and the NCBI database to identify the potential miRNA target genes. The biological processes, cellular components and molecular functions of the miRNA target genes were obtained. The results revealed the functions significantly related with the predicted target gene candidates of the miRNA.

The KEGG pathway analyses were performed for the predicted miRNA target genes, to further investigate the understanding their biological functions. These putative miRNA target sequences were used as queries against the KEGG database.

## Competing interest

The authors declare no competing interests.

## Author’s contribution

HY contributed to miRNA sequencing, and qRT-PCR analyses. MT contributed to bioinformatics analyses, manuscript preparation and critical edition. ED collected plant samples, planned the experimental design, isolated the RNA samples and performed critical edition of the manuscript. PH contributed to miRNA sequencing, bioinformatics analyses and manuscript critical edition. GD contributed to miRNA sequencing, bioinformatics analyses and manuscript critical edition. TU conceived and organized the experiments, contributed to miRNA sequencing, bioinformatics analyses, and preparation and manuscript critical edition. All authors have read and approved the final manuscript.

## Author’s information

TU is group leader at the Cankiri Karatekin University GD is Tenured Full Professor at the Cordoba University (Spain). PH is Tenured Scientist at the Instituto de Agricultura Sostenible (IAS-CSIC) (Spain).

## Supplementary Material

Additional file 1 **Sequences of conserved miRNAs discovered in olive.** A total of 136 conserved miRNA, belonging to 22 miRNA families, were identified in the six *Olea europaea* L. libraries. Click here for file

Additional file 2 **Predicted secondary structures of the putative novel miRNA.** A total of 38 putative novel miRNA were detected in the six libraries. The mature miRNA sequences are shown in green color. Click here for file

Additional file 3 The list of miRNA* sequences from six sRNA libraries. Click here for file

Additional file 4 **Gene ontology terms of biological function and molecular processes of miRNA targets.** Members of 22 conserved miRNA families were involved in diverse biological functions and processes. Click here for file

Additional file 5 **The most abundant gene ontology terms from the biological function ontology.** Eight ontology terms were found with significant abundance (p <0.05). Click here for file

Additional file 6 **The most abundant gene ontology terms from the molecular process ontology.** A total of 108 ontology terms were found with significant abundance (p <0.05). Click here for file

Additional file 7 **Pathway annotation of the miRNA targets.** The miRNA targets were included in 130 KEGG pathways. Click here for file

Additional file 8 Primer sequences of selected miRNA and target genes for qRT–PCR validation experiments. Click here for file
