# Supplementary material for: Genome-wide identification of alternate bearing-associated microRNAs (miRNAs) in olive (Olea europaea L.)
Source: BMC Plant Biol. 2013 Jan 15;13:10. doi: 10.1186/1471-2229-13-10 (PMC3564680; doi:10.1186/1471-2229-13-10)
Supplement: Additional file 1 — Sequences of conserved miRNAs discovered in olive. A total of 136 conserved miRNA, belonging to 22 miRNA families, were identified in the six Olea europaea L. libraries. [file 1471-2229-13-10-S1.doc]

**Additional file 1: Sequences of conserved miRNA discovered in olive.** 136 conserved miRNAs which belong to 22 miRNA families were identified in six *O. europaea* L. libraries.

**miRNA Name Mature miRNA sequence (5’ –> 3’)**

oeu_miR156a ACUGACAGAAGAGAGUGAGCA

oeu_miR156b AGGUGACAGAAGAGAGUGAGCAC

oeu_miR156c ACUGACAGAAGAGAGUGAGCA

oeu_miR156d UUGACAGAAGAGAGUGAGCAC

oeu_miR156e UUGACAGAAGAGAGUGAGCAC

oeu_miR156f ACUGACAGAAGAGAGUGAGCA

oeu_miR156g GUUGUUGACAGAAGAUAGAGAGCA

oeu_miR156h UGUUGACAGAAGAUAGAGAGC

oeu_miR156i GUUGUUGACAGAAGAUAGAGAGCA

oeu_miR156j GUUGUUGACAGAAGAUAGAGAGCA

oeu_miR156k UGACAGAAGAGAGGGAGCAC

oeu_miR159a UUUGGAUUGAAGGGAGCUCUA

oeu_miR159b UUUGGAUUGAAGGGAGCUCUA

oeu_miR159c UUUGGAUUGAAGGGAGCUCUA

oeu_miR159d UUGGAUUGAAGGGAGCUC

oeu_miR160a AUGCCUGGCUCCCUGUAUGCCACA

oeu_miR160b UGCCUGGCUCCCUGUAUGCCA

oeu_miR160c UGCCUGGCUCCCUGUAUGCCA

oeu_miR160d AUGCCUGGCUCCCUGUAUGCCACA

oeu_miR160g UGCCUGGCUCCCUGGAUGCCA

oeu_miR164a UGGAGAAGCAGGGCACGUGCA

oeu_miR164b UGGAGAAGCAGGGCACGUGCA

oeu_miR164c UGGAGAAGCAGGGCACGUGCA

oeu_miR164d UGGAGAAGCAGGGCACGUGCA

oeu_miR164e UGGAGAAGCAGGGCACGUGCA

oeu_miR164f UGGAGAAGCAGGGCACAUG

oeu_miR166a UUUGAGGGGAAUGUUGUCUGG

oeu_miR166b UUUCGGACCAGGCUUCAUUCC

oeu_miR166c UUUGAGGGGAAUGUUGUCUGG

oeu_miR166d CUUCGGACCAGGCUUCAUUCC

oeu_miR166e UUUCGGACCAGGCUUCAUUCC

oeu_miR166f CUUCGGACCAGGCUUCAUUCC

oeu_miR166g AUCUCGGACCAGGCUUCAUUCCCC

oeu_miR166h CGAAUGAUCUCGGACCAGGCU

oeu_miR166i CCUCGGACCAGGCUUCAUUCC

oeu_miR166j UUUGAGGGGAAUGUUGUCUGG

oeu_miR166k UUUGAGGGGAAUGUUGUCUGG

oeu_miR166l CUCGGACCAGGCUUCAUUCC

oeu_miR166m CUCUCGGACCAGGCUUCAUUC

oeu_miR166n AUCUCGGACCAGGCUUCAUUC

oeu_miR166o AUCUCGGACCAGGCUUCAUUC

oeu_miR166p UCUCGGACCAGGCUCCAUUCC

oeu_miR166q GGAAUGUUGUCUGGCUCGAGG

oeu_miR167a UGAAGCUGCCAGCAUGAUCUAA

oeu_miR167b UGAAGCUGCCAGCAUGAUCUA

oeu_miR167c UGAAGCUGCCAGCAUGAUCUAA

oeu_miR167d UGAAGCUGCCAGCAUGAUCUA

oeu_miR167e UUGAAGCUGCCAGCAUGAUCUG

oeu_miR167f UGAAGCUGCCAGCAUGAUCUU

oeu_miR167g UGAAGCUGCCAGCAUGAUCUU

oeu_miR168a AUUCGCUUGGUGCAGGUCGGG

oeu_miR168b UAAUUCGCUUGGUGCAGGUCG

oeu_miR169a CAGCCAAGGAUGACUUGCCGA

oeu_miR169b CAGCCAAGGAUGACUUGCCGA

oeu_miR169c CAGCCAAGGAUGACUUGCCGA

oeu_miR169d CAGCCAAGGAUGACUUGCCGG

oeu_miR169e CAGCCAAGGAUGACUUGCCGG

oeu_miR169f CAGCCAAGGAUGACUUGCCGG

oeu_miR169g CAGCCAAGGAUGACUUGCCGG

oeu_miR169h CAGCCAAGGAUGACUUGCCGG

oeu_miR169i UAGCCAAGGAUGACUUGCC

oeu_miR169j UAGCCAAGGAUGACUUGCC

oeu_miR169k UAGCCAAGGAUGACUUGCC

oeu_miR169l UAGCCAAGGAUGACUUGCC

oeu_miR169m UAGCCAAGGAUGACUUGCC

oeu_miR169r UAGCCAAGGAUGACUUGCCUA

oeu_miR169s CAGCCAAGGAUGACUUGCCGG

oeu_miR169v UAGCCAAGGAUGACUUGCC

oeu_miR169w UAGCCAAGGAUGACUUGCC

oeu_miR171a UGAUUGAGCCGUGCCAAUAUC

oeu_miR171b UGAUUGAGCCGUGCCAAUAUC

oeu_miR171c AAGAAAGCGAUGUUGGUGAGGUU

oeu_miR171d AAGAAAGCGAUGUUGGUGAGGUU

oeu_miR171e UGAUUGAGCCGUGCCAAUAUC

oeu_miR171f UGAUUGAGCCGUGCCAAUAUC

oeu_miR171g UGAUUGAGCCGUGCCAAUAUC

oeu_miR171h UGAUUGAGCCGUGCCAAUAUC

oeu_miR171i UGAUUGAGCCGUGCCAAUAUC

oeu_miR172a GAGAAUCUUGAUGAUGCUGCAU

oeu_miR172b GAGAAUCUUGAUGAUGCUGCAU

oeu_miR172c GAGAAUCUUGAUGAUGCUGCAU

oeu_miR172d GAAUCUUGAUGAUGCUGCAU

oeu_miR172e GAAUCUUGAUGAUGCUGCAU

oeu_miR172f GAGAAUCUUGAUGAUGCUGCAU

oeu_miR172g GAAUCUUGAUGAUGCUGCA

oeu_miR172h GAAUCUUGAUGAUGCUGCA

oeu_miR172i AGAAUCCUGAUGAUGCUGCA

oeu_miR319a CUUGGACUGAAGGGAGCUCC

oeu_miR319b UUGGACUGAAGGGAGCUCC

oeu_miR319c UGUGCUUGGACUGAAGGGAGC

oeu_miR319d UGUGCUUGGACUGAAGGGAGC

oeu_miR319e UUGGACUGAAGGGAGCUCC

oeu_miR319f UGUGCUUGGACUGAAGGGAGC

oeu_miR319g UGUGCUUGGACUGAAGGGAGC

oeu_miR319h UUGGACUGAAGGGAGCUCC

oeu_miR390a AAGCUCAGGAGGGAUAGCGCC

oeu_miR390b AAGCUCAGGAGGGAUAGCGCC

oeu_miR390c AAGCUCAGGAGGGAUAGCGCC

oeu_miR390d AAGCUCAGGAGGGAUAGCGCC

oeu_miR393a UCCAAAGGGAUCGCAUUGAUC

oeu_miR393b UCCAAAGGGAUCGCAUUGAUC

oeu_miR393c UUCCAAAGGGAUCGCAUUGAUC

oeu_miR393d UUCCAAAGGGAUCGCAUUGAUC

oeu_miR394a-5p UUGGCAUUCUGUCCACCUCC

oeu_miR394b-5p UAUUGGCAUUCUGUCCACCUC

oeu_miR395b CUGAAGUGUUUGGGGGAACUC

oeu_miR395c CUGAAGUGUUUGGGGGAACUC

oeu_miR395d CUGAAGUGUUUGGGGGAACUC

oeu_miR395e CUGAAGUGUUUGGGGGAACUC

oeu_miR395f CUGAAGUGUUUGGGGGAACUC

oeu_miR395g CUGAAGUGUUUGGGGGAACUC

oeu_miR395h CUGAAGUGUUUGGGGGAACUC

oeu_miR395i CUGAAGUGUUUGGGGGAACUC

oeu_miR395j CUGAAGUGUUUGGGGGAACUC

oeu_miR396a UUCCACAGCUUUCUUGAACUG

oeu_miR396b UUCCACAGCUUUCUUGAACUG

oeu_miR396c UUCCACAGCUUUCUUGAACUU

oeu_miR396d UUCCACAGCUUUCUUGAACUU

oeu_miR396e UUCCACAGCUUUCUUGAACUU

oeu_miR396f UUUUCCACGGCUUUCUUGAAC

oeu_miR396g UUUUCCACGGCUUUCUUGAAC

oeu_miR397a UCAUUGAGUGCAGCGUUGAUG

oeu_miR397b AUUGAGUGCAGCGUUGAUGA

oeu_miR398b UGUGUUCUCAGGUCGCCCCUG

oeu_miR398c UGUGUUCUCAGGUCGCCCCUG

oeu_miR399b UGCCAAAGGAGAUUUGCCCGG

oeu_miR399c UGCCAAAGGAGAUUUGCCCGG

oeu_miR399f UUUGCCAAAGGAGAAUUGCCC

oeu_miR399g GUGCAAUUCUCCUUUGGCAGA

oeu_miR399i UGCCAAAGGAGAGUUGCCCUA

oeu_miR403a UUAGAUUCACGCACAAACUCG

oeu_miR403b UUAGAUUCACGCACAAACUCG

oeu_miR403c UUAGAUUCACGCACAAACUCG

oeu_miR408 UGCACUGCCUCUUCCCUGGC

oeu_miR530a UGCAUUUGCACCUGCACC
