# Supplementary material for: Genome-wide identification of alternate bearing-associated microRNAs (miRNAs) in olive (Olea europaea L.)
Source: BMC Plant Biol. 2013 Jan 15;13:10. doi: 10.1186/1471-2229-13-10 (PMC3564680; doi:10.1186/1471-2229-13-10)
Supplement: Additional file 3 — The list of miRNA* sequences from six sRNA libraries. [file 1471-2229-13-10-S3.doc]

**Additional file 2. Predicted secondary structures of the novel miRNA.** A total of 38 novel miRNA were detected in the six libraries. The mature miRNA sequences are shown in green color.

|  | **Structure** | **Initial ΔG kcal/mol** |
| --- | --- | --- |
| VK1-m0001 | 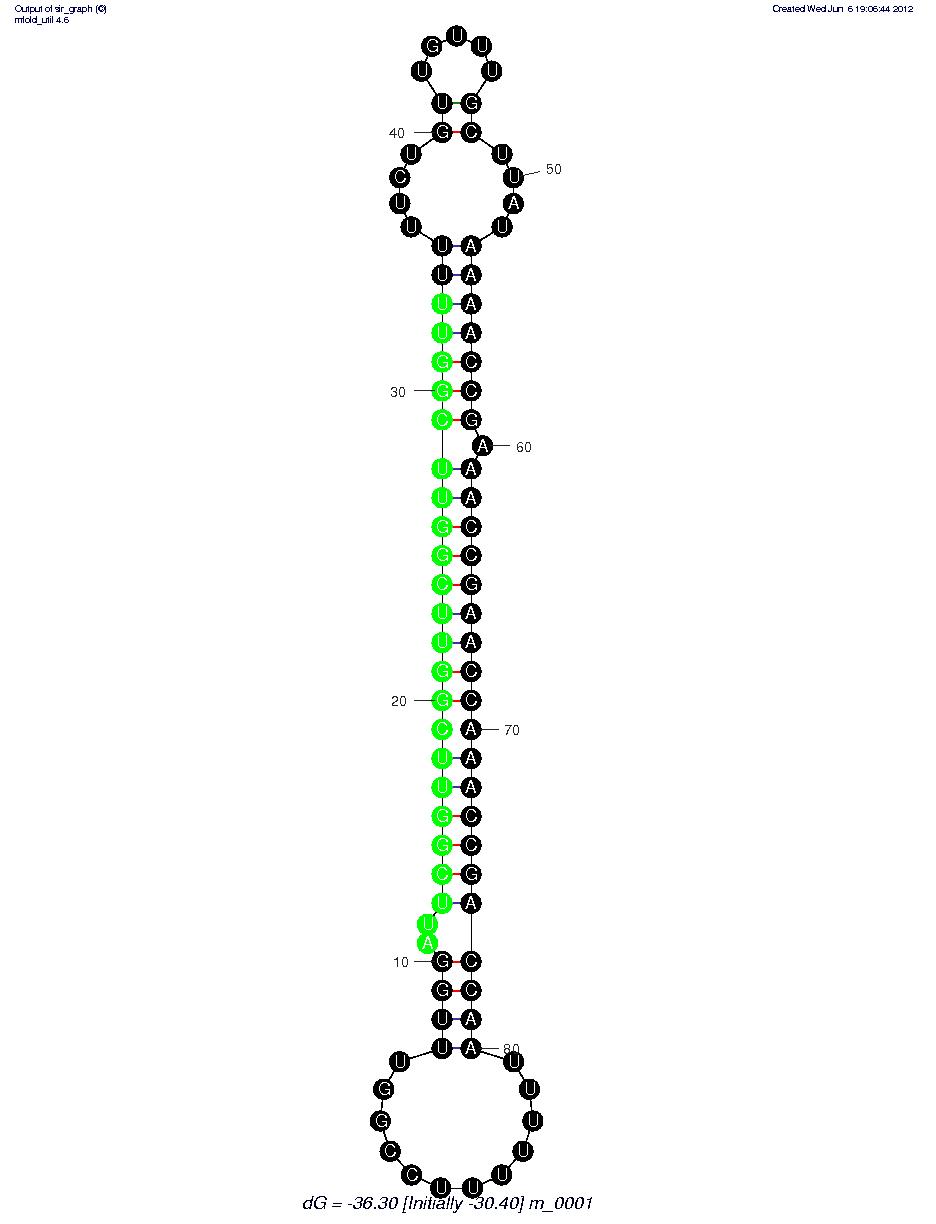 | -30.40 |
| VK1-m0002 | 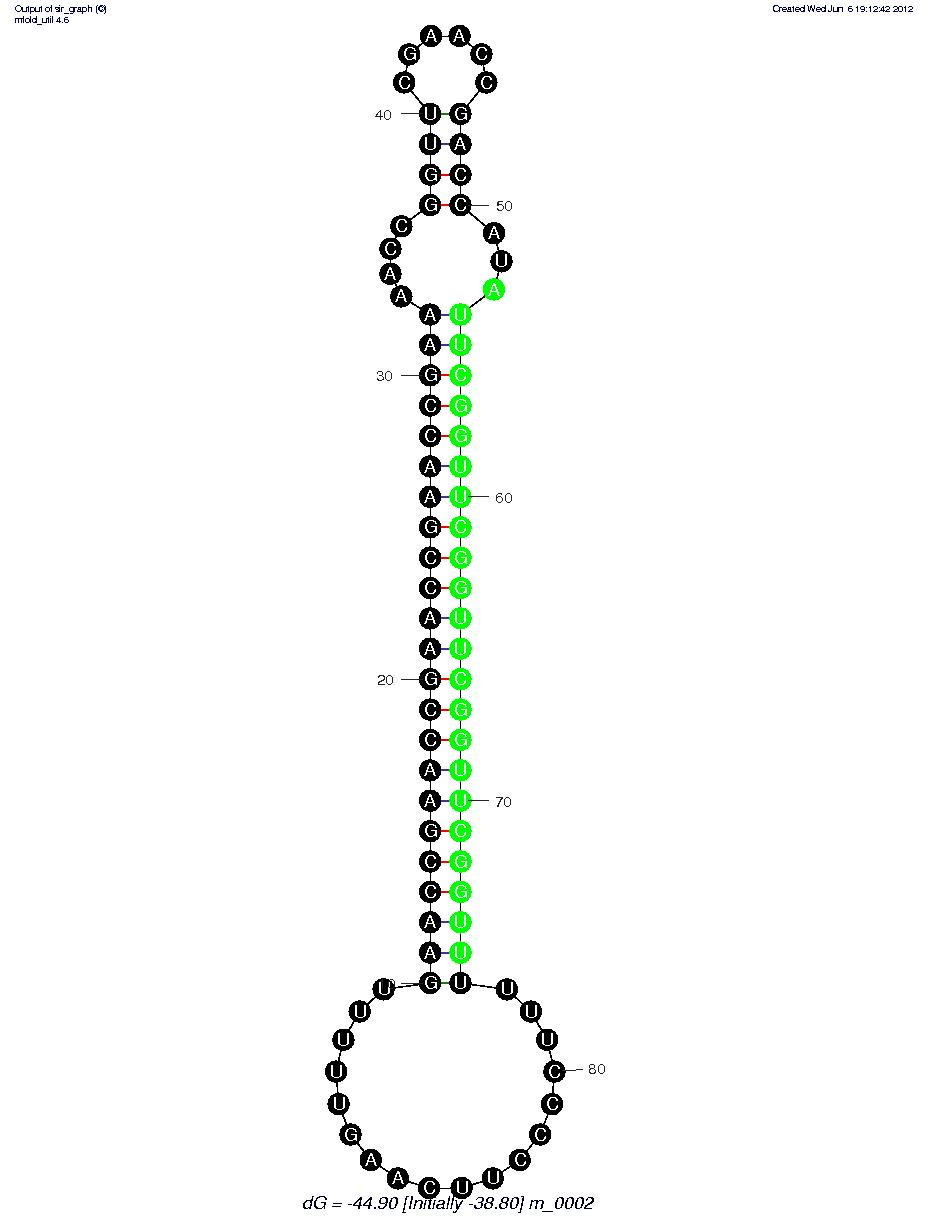 | -38.80 |
| VK1-m0003 | 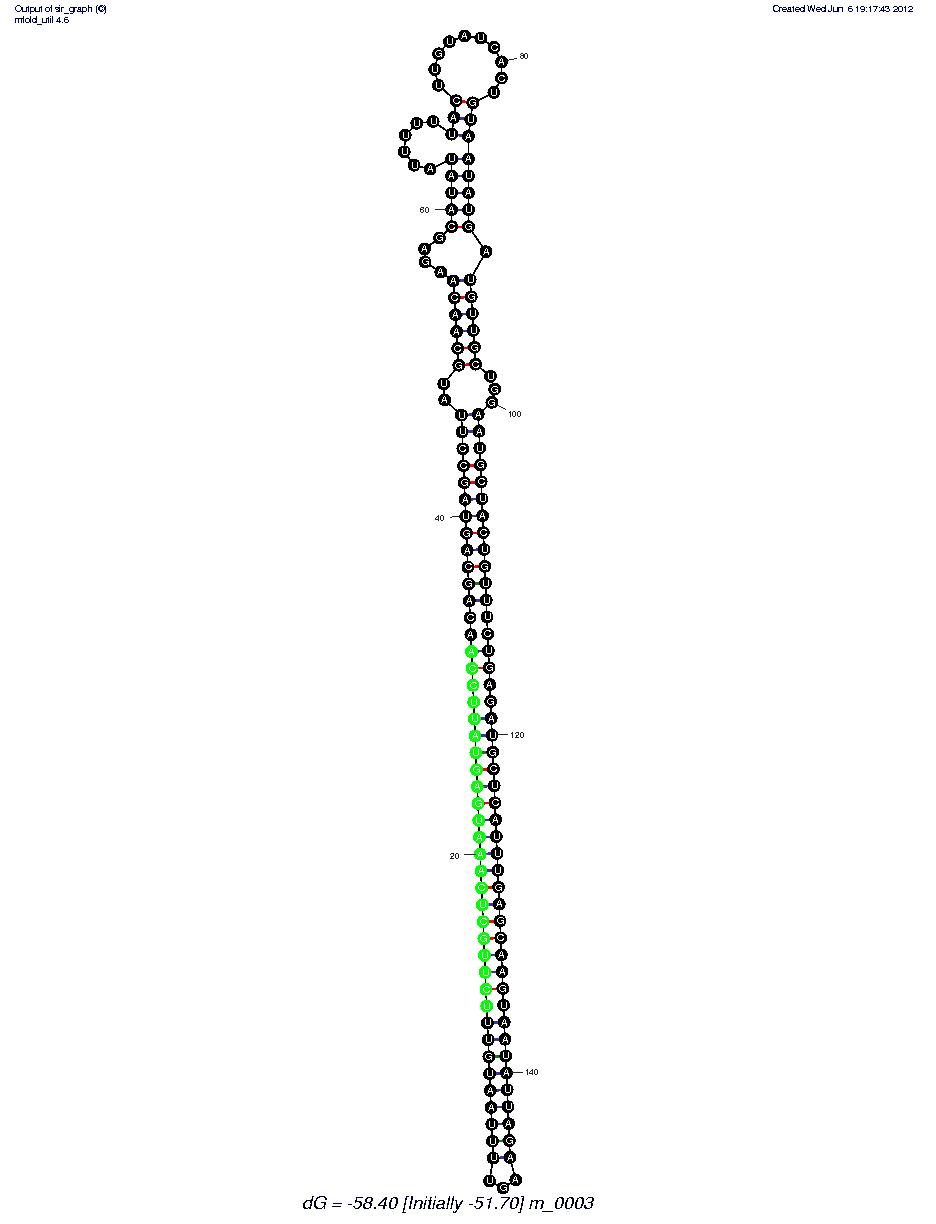 | -51.70 |
| VK1-m0004 | 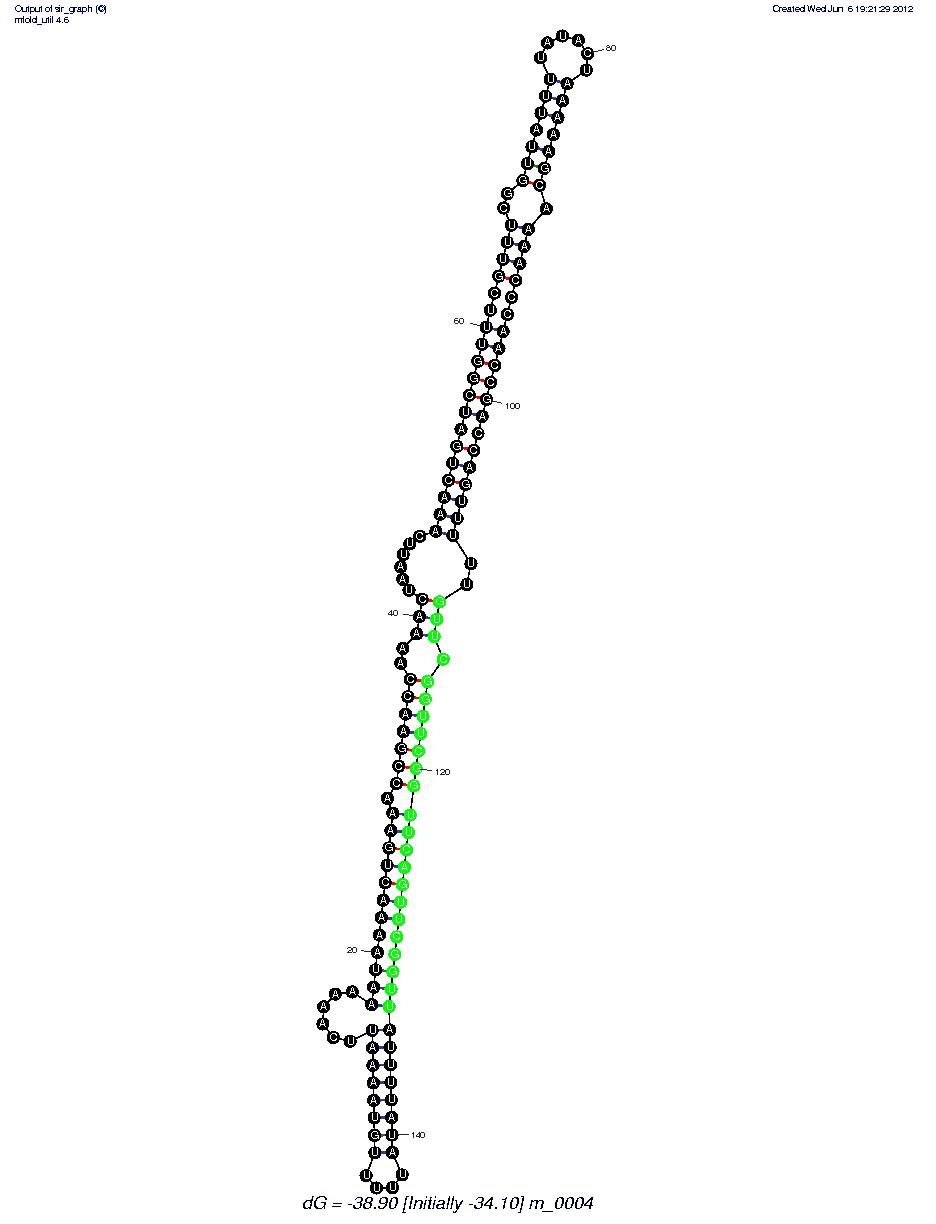 | -34.10 |
| VK1-m0005 | 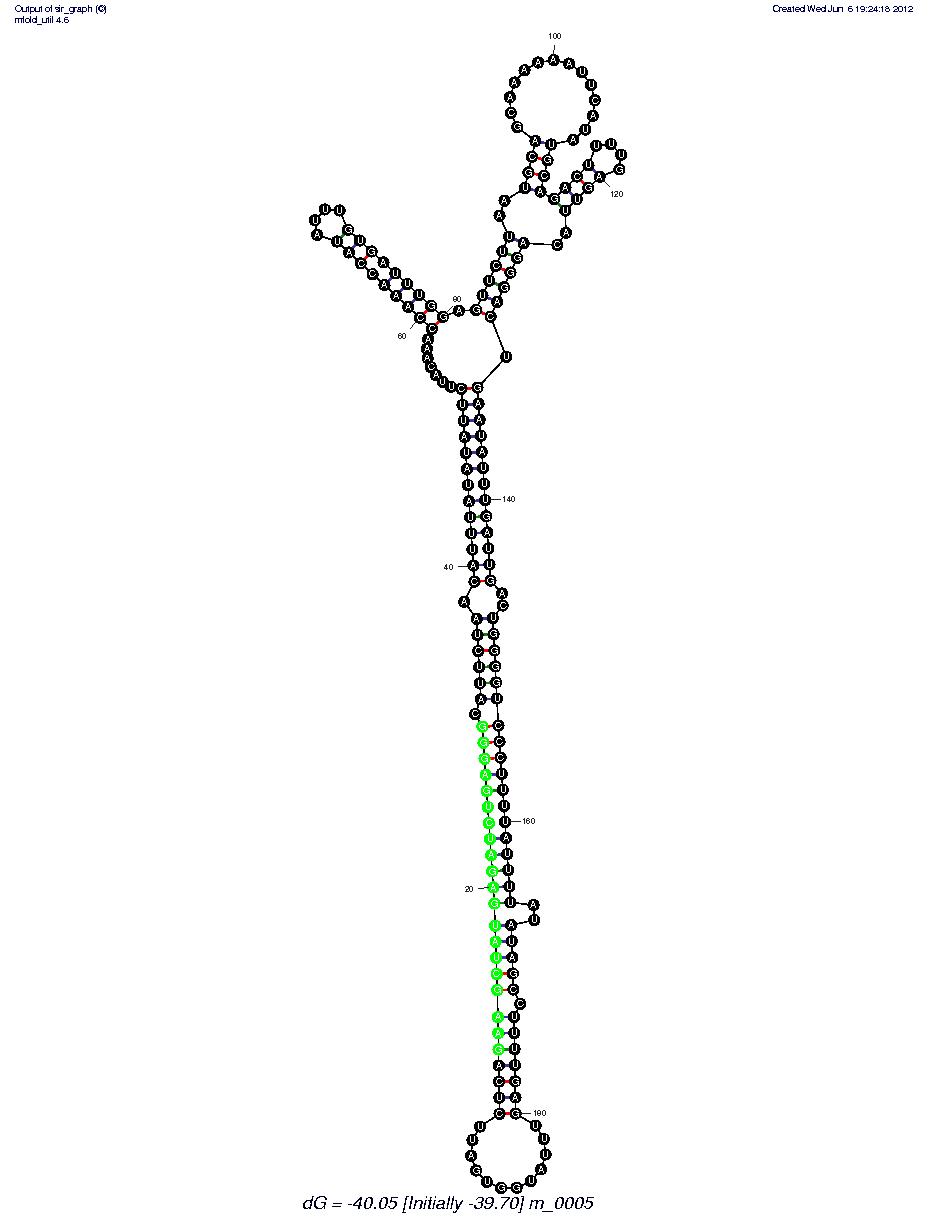 | -39.70 |
| VK1-m0006 | 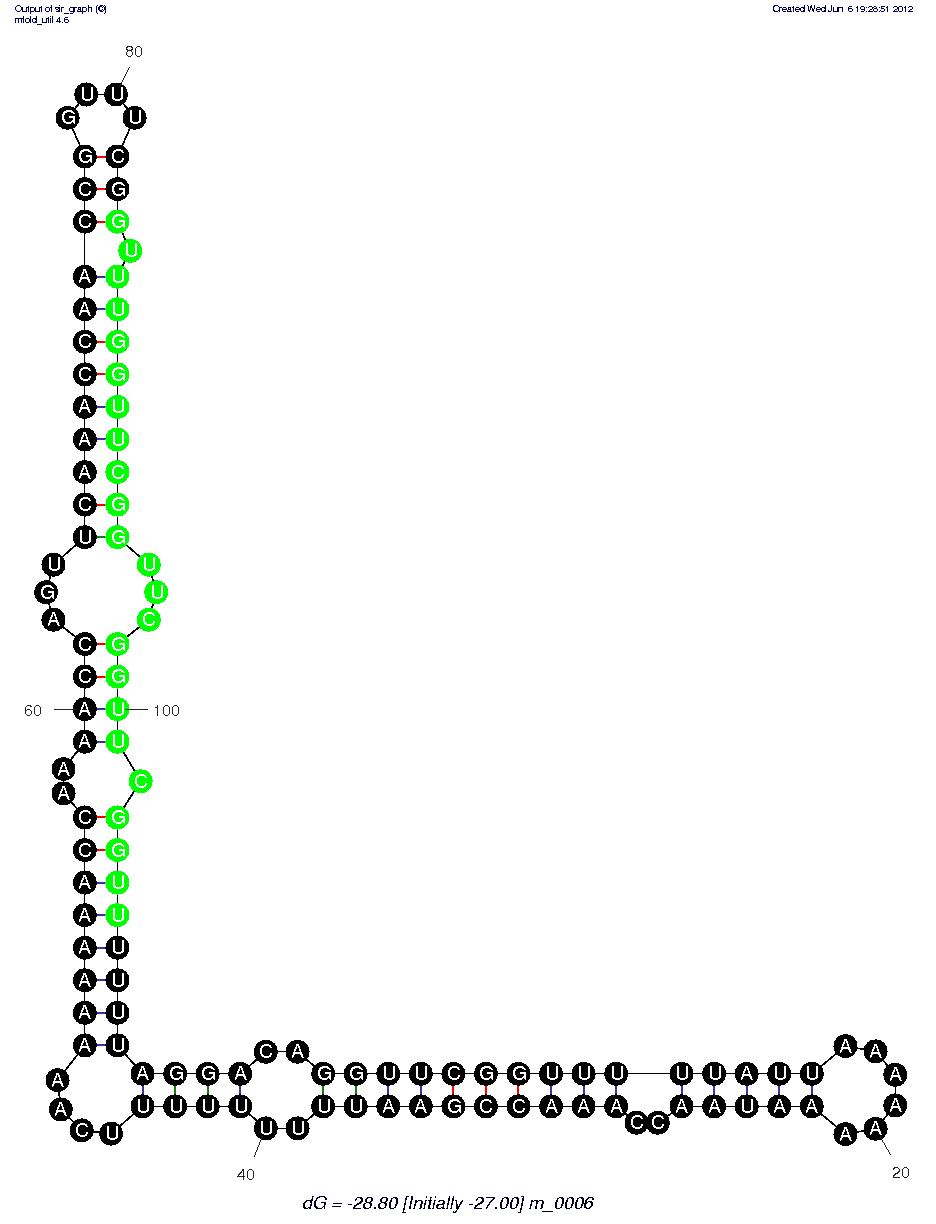 | -27.00 |
| VK1-m0007 | 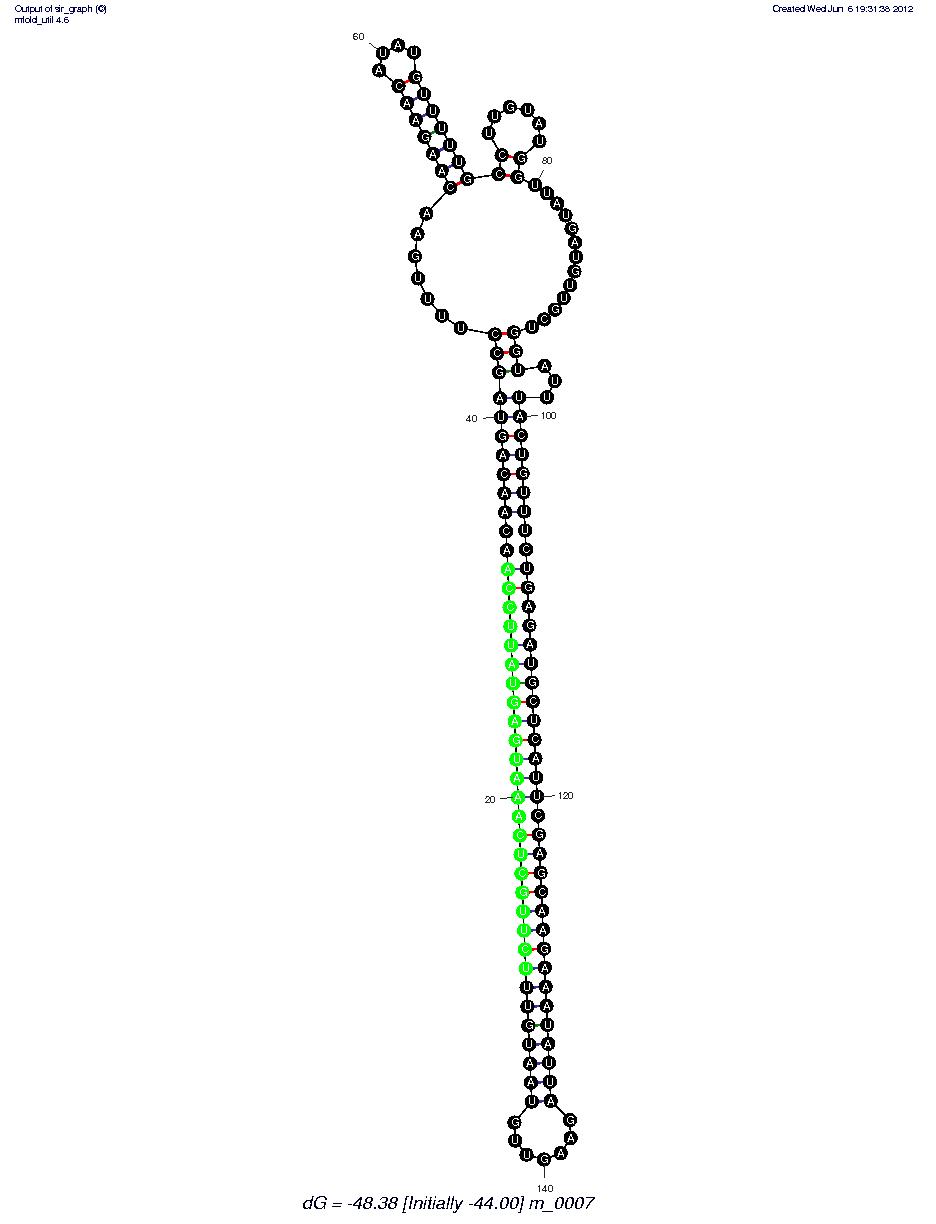 | -44.00 |
| VK1-m0008 | 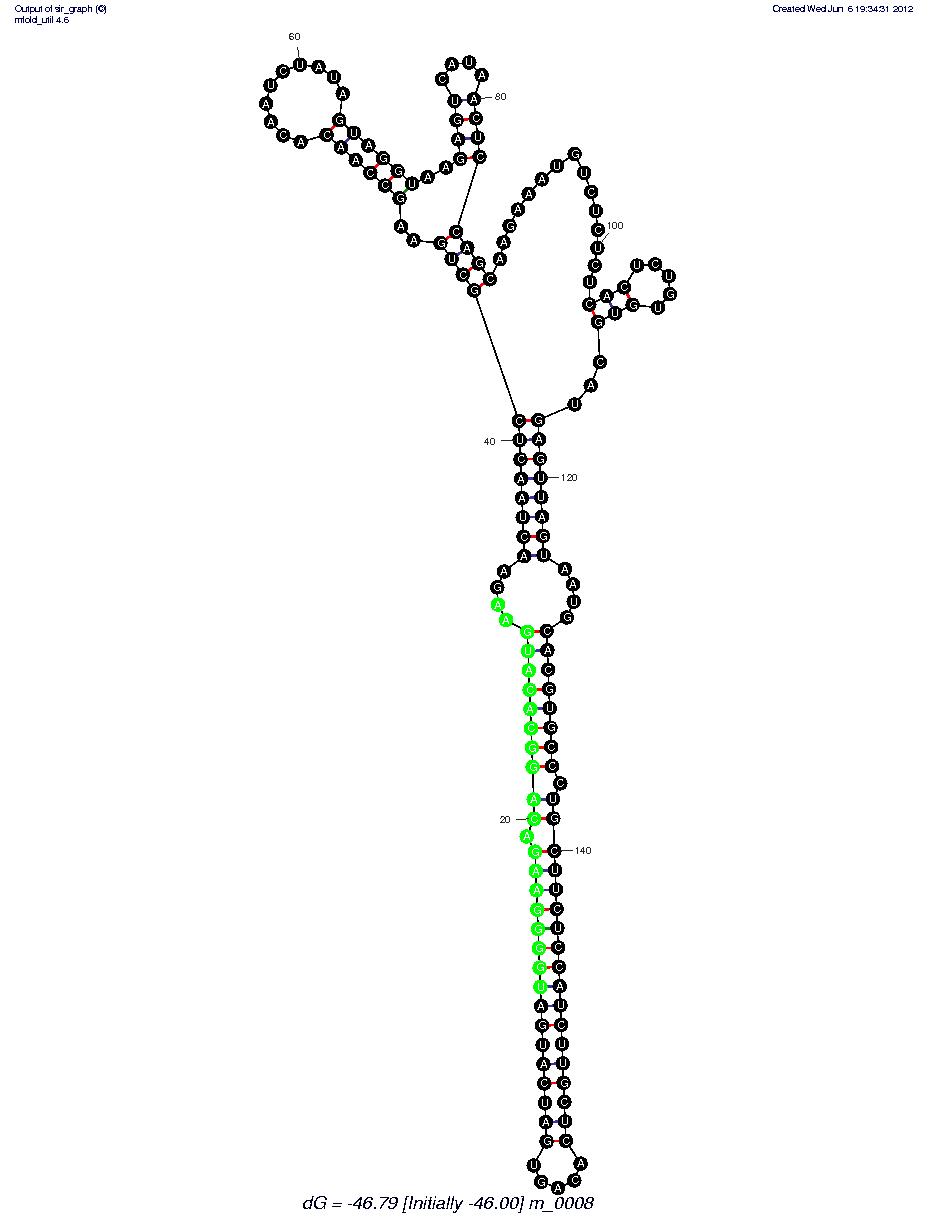 | -46.00 |
| VK1-m0009 | 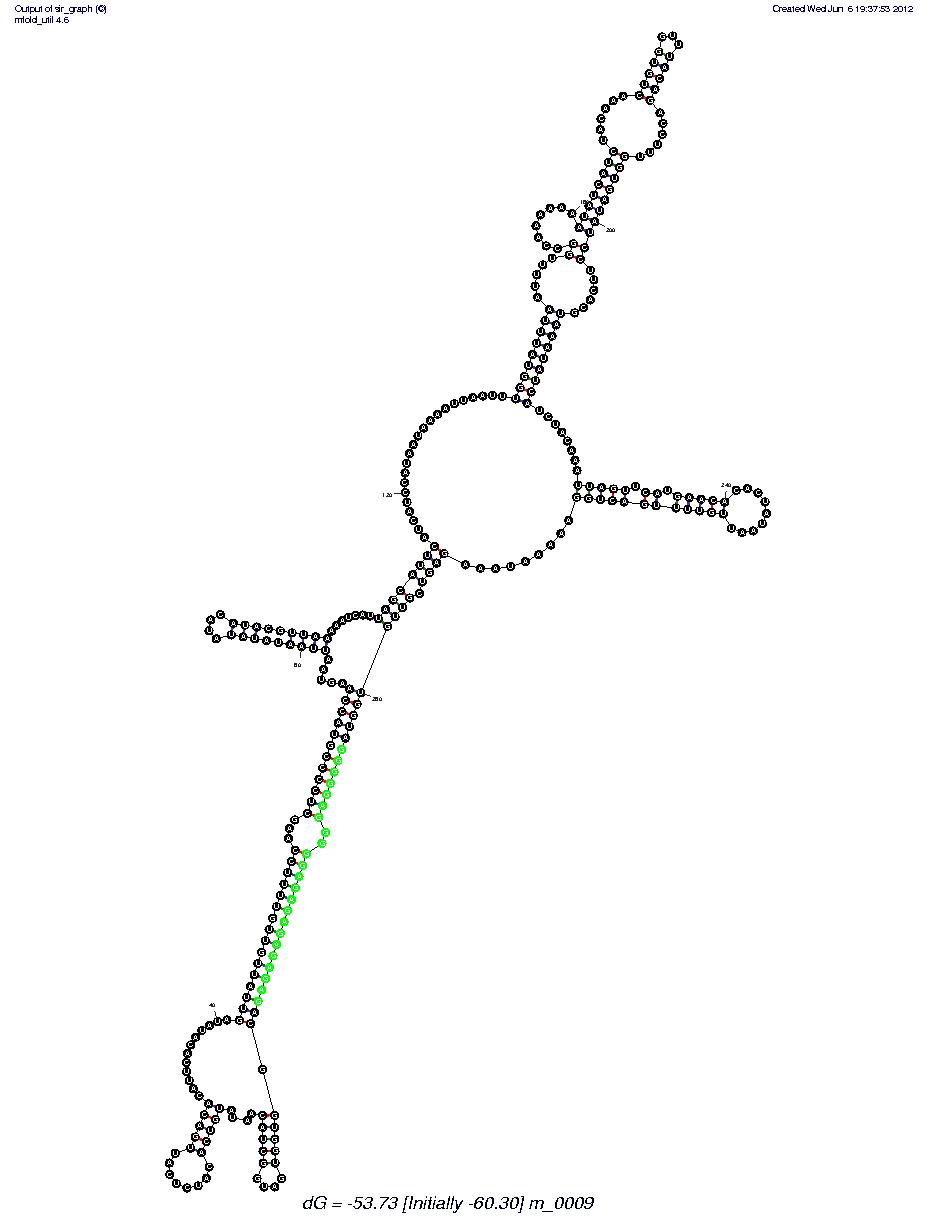 | -60.30 |
| VK1-m0010 | 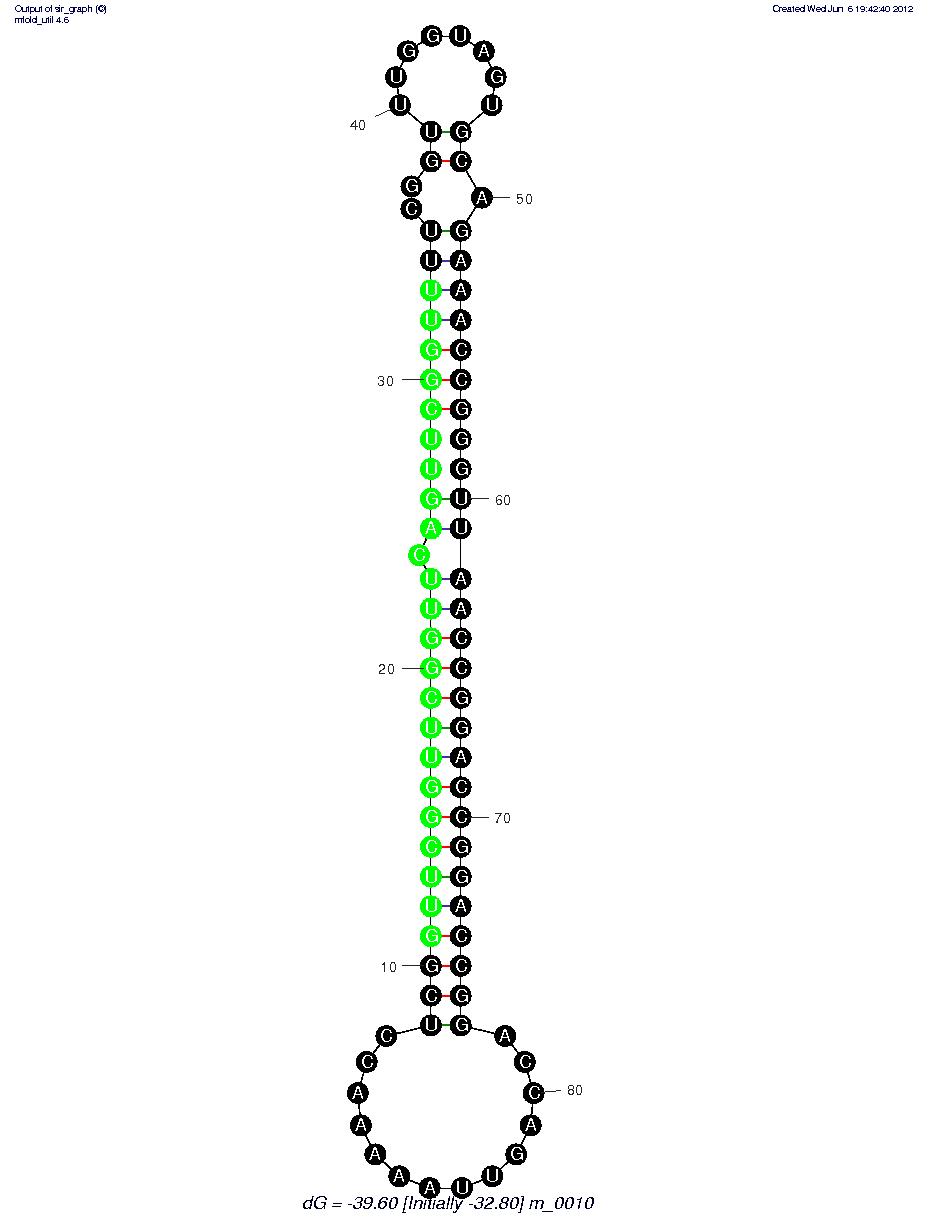 | -32.80 |
| VK1-m0011 | 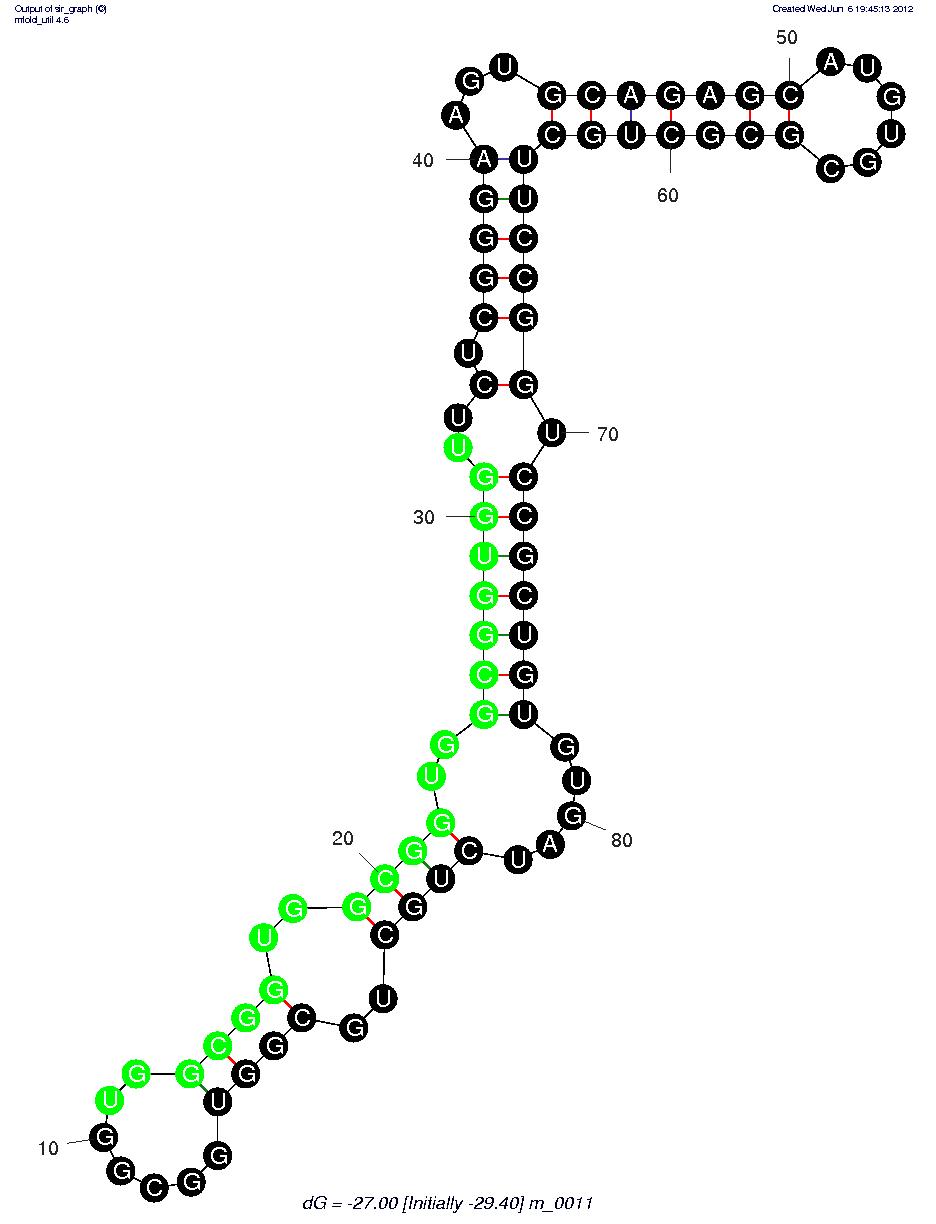 | -29.40 |
| VK1-m0012 | 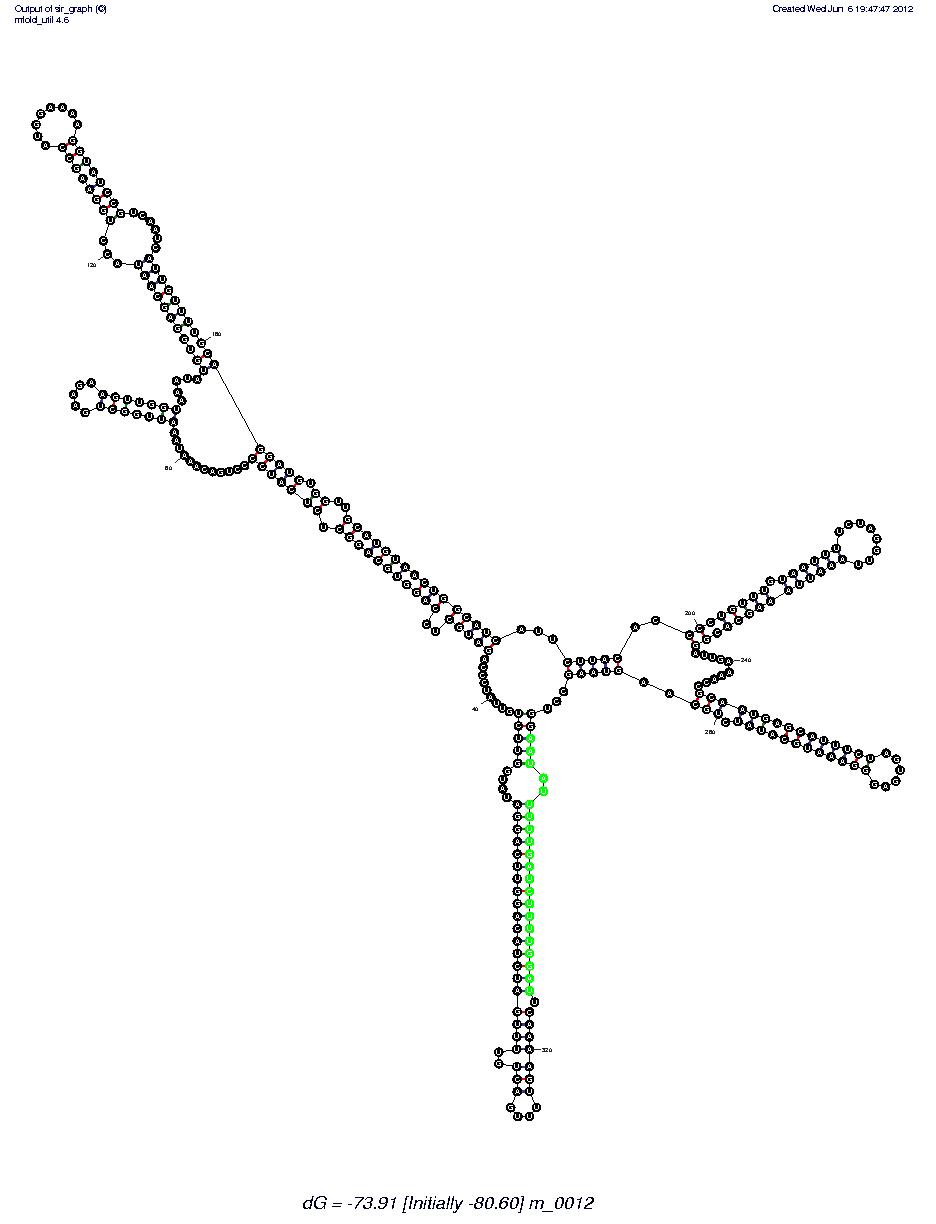 | -80.60 |
| VK1-m0013 | 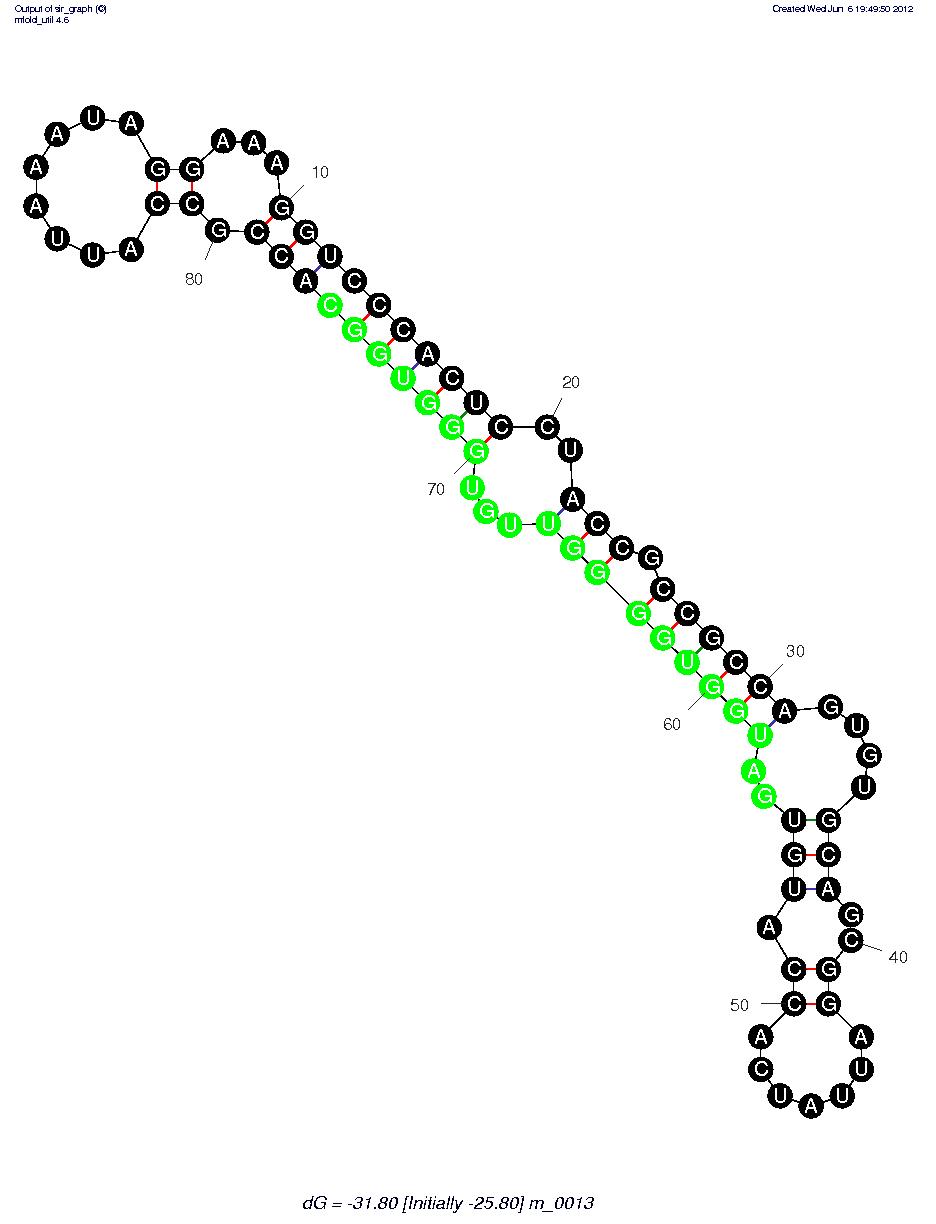 | -25.80 |
| VK1-m0014 | 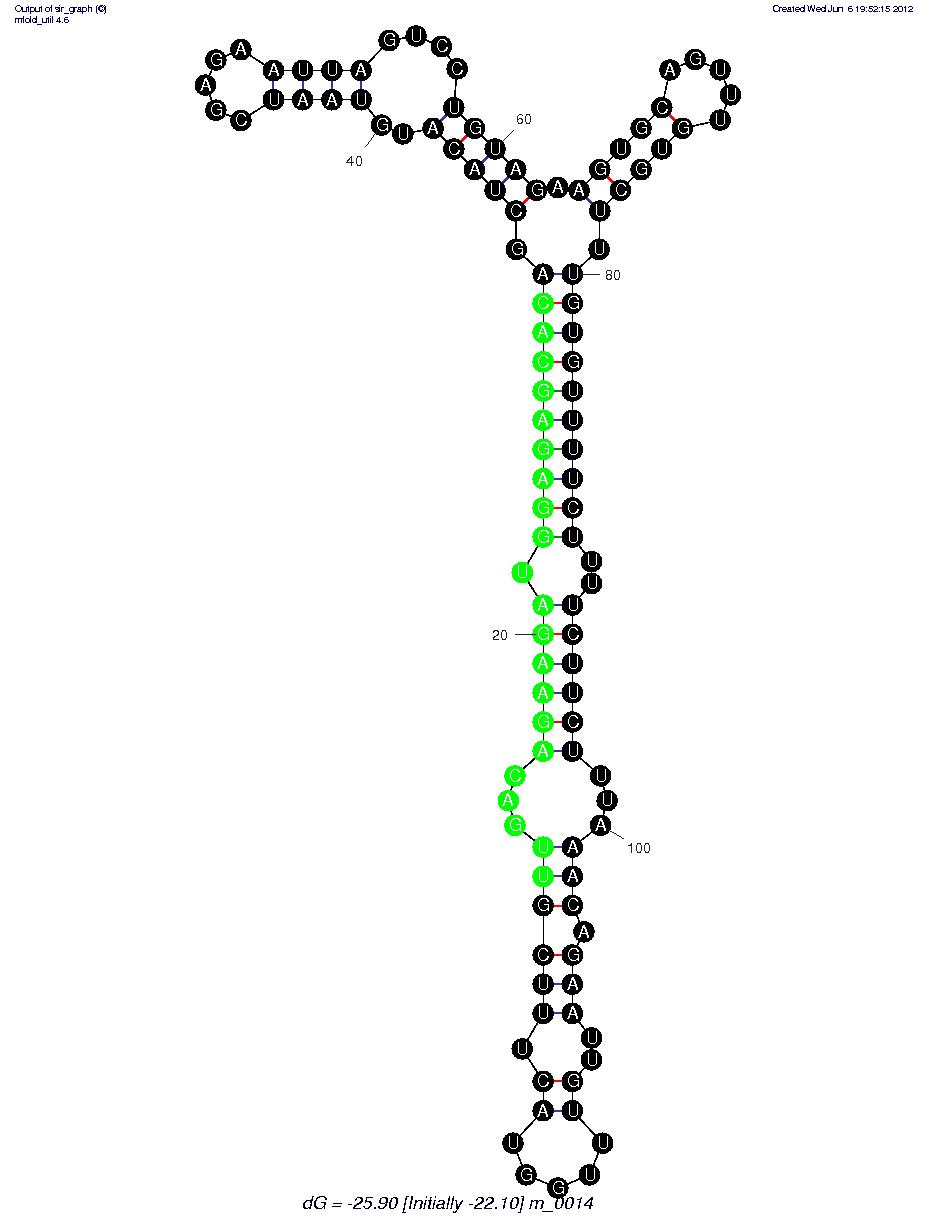 | -22.10 |
| VK1-m0015 | 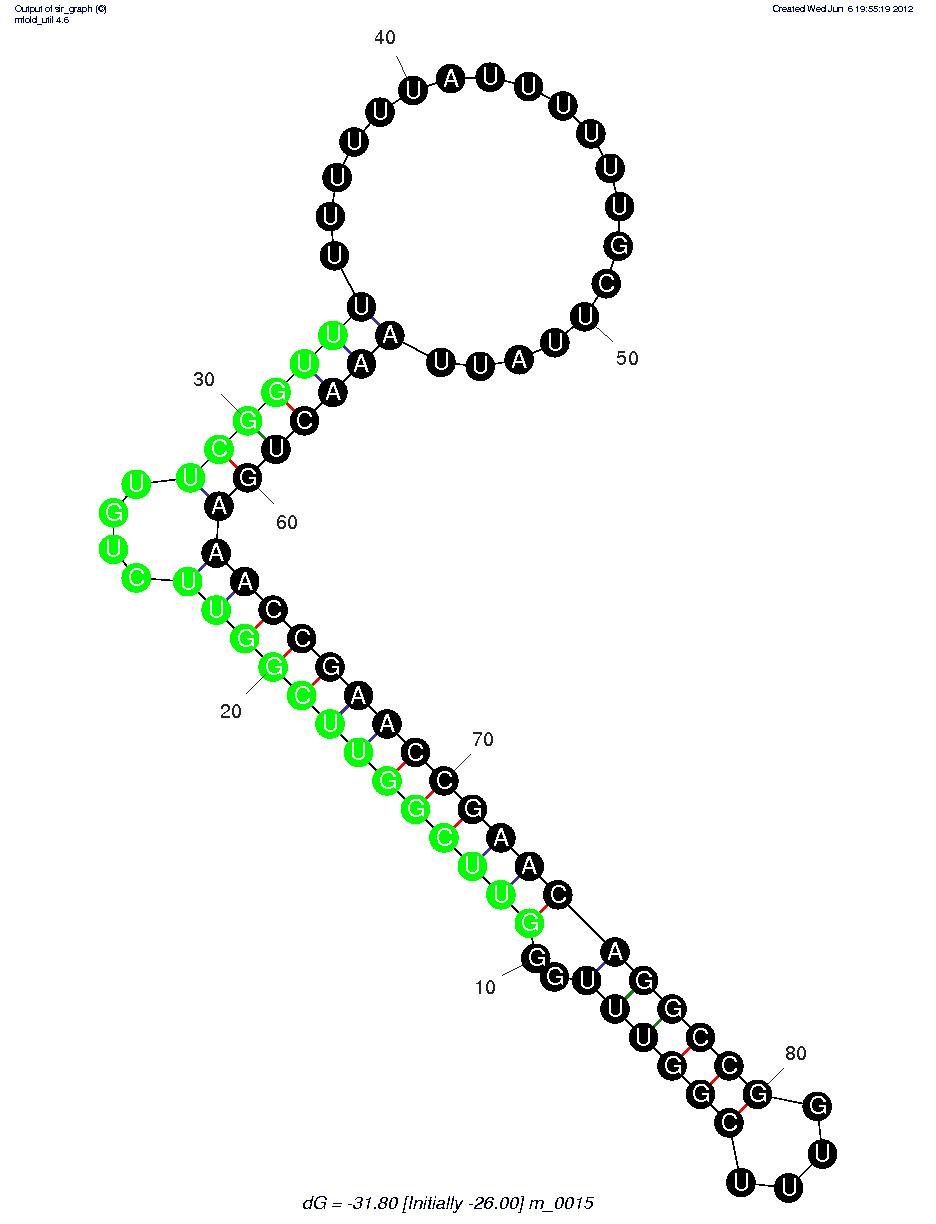 | -26.00 |
| VK1-m0016 | 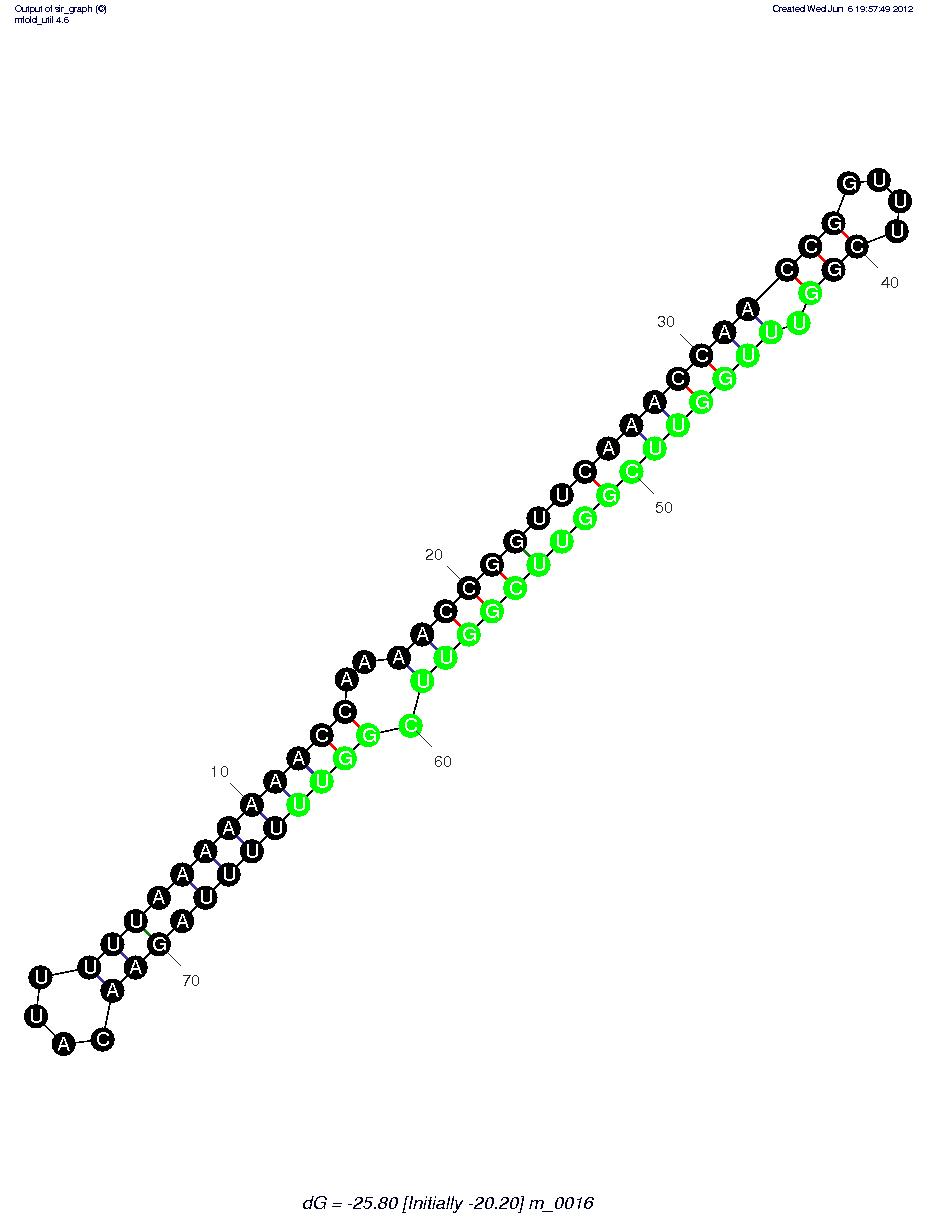 | -20.20 |
| VK1-m0017 | 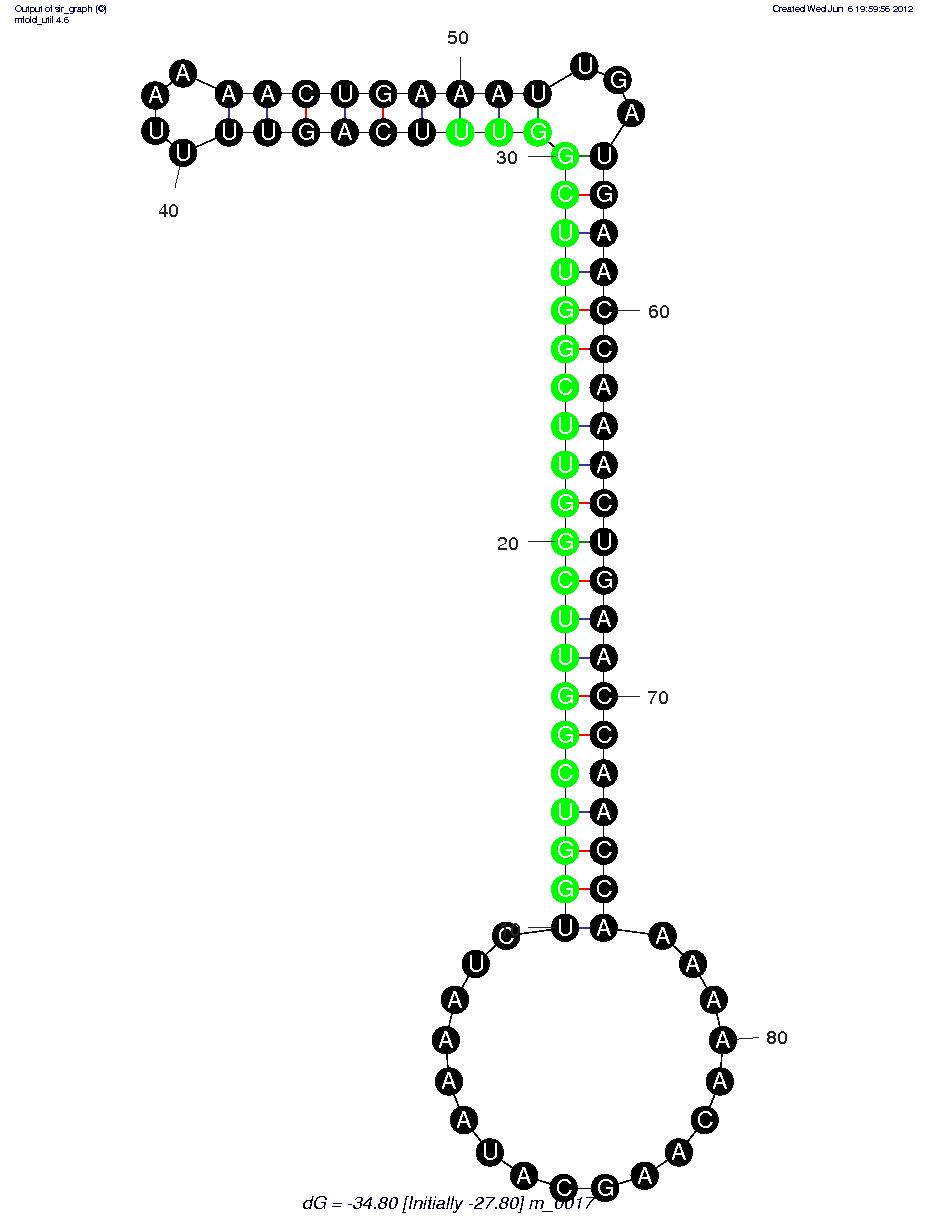 | -27.80 |
| VK1-m0018 | 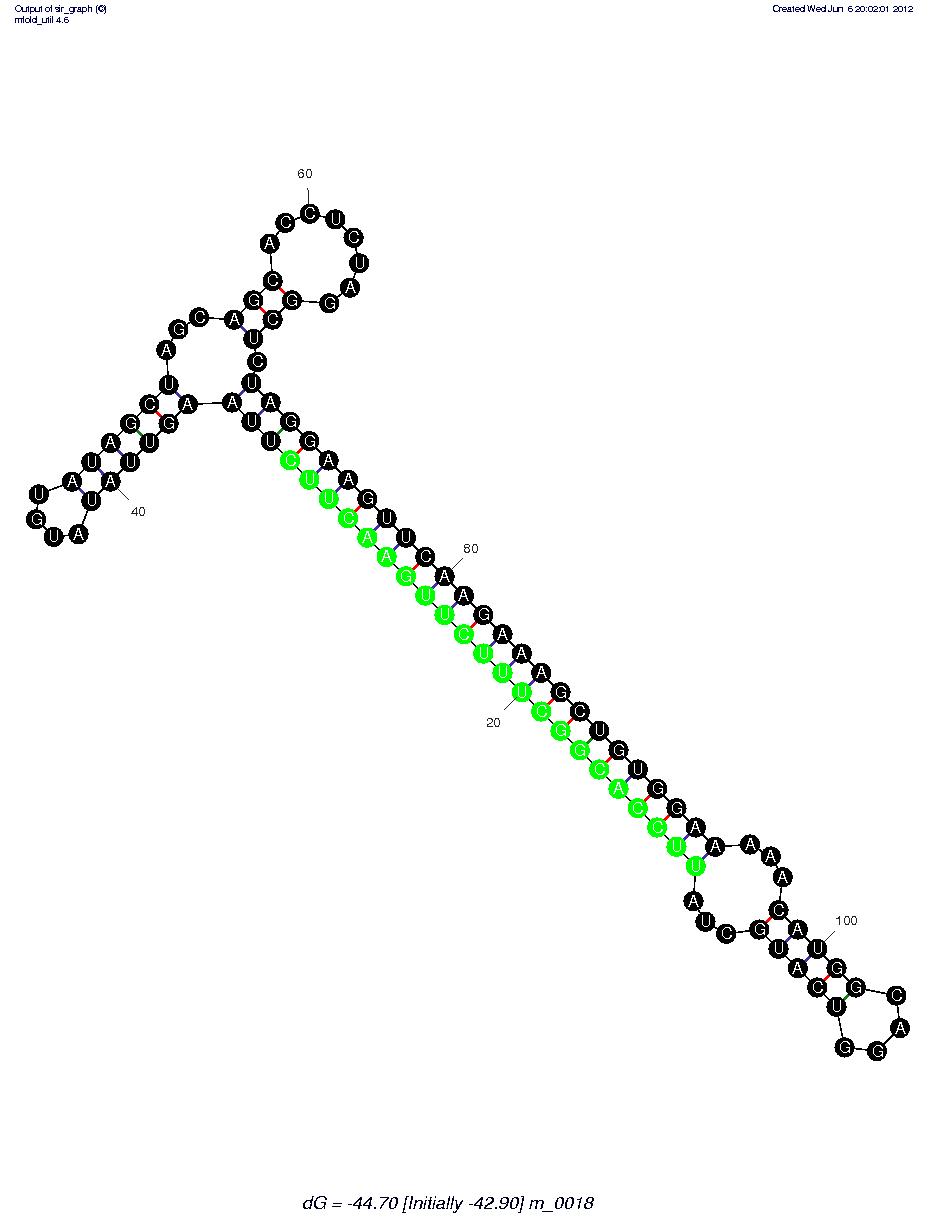 | -42.90 |
| VK1-m0019 | 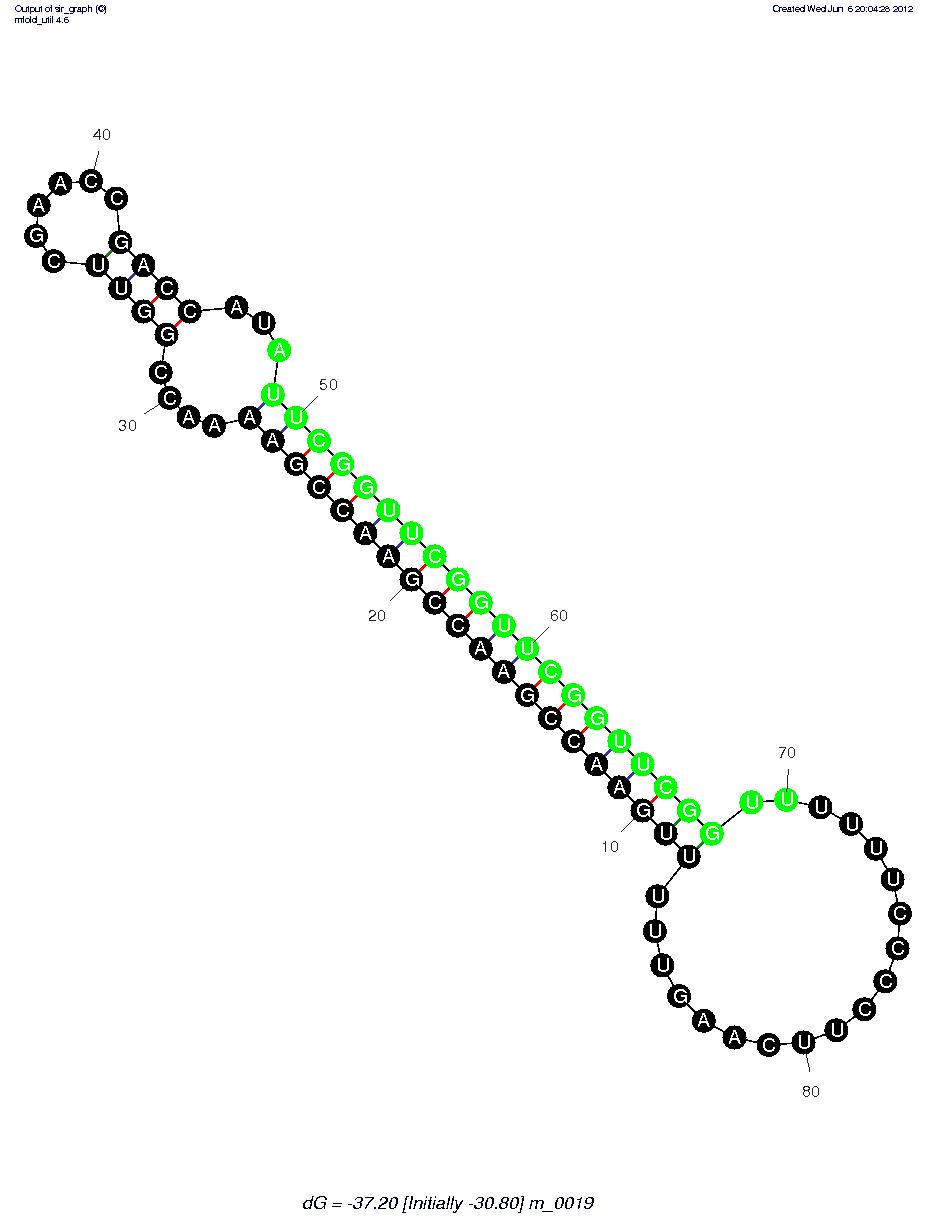 | -30.80 |
| VK1-m0020 | 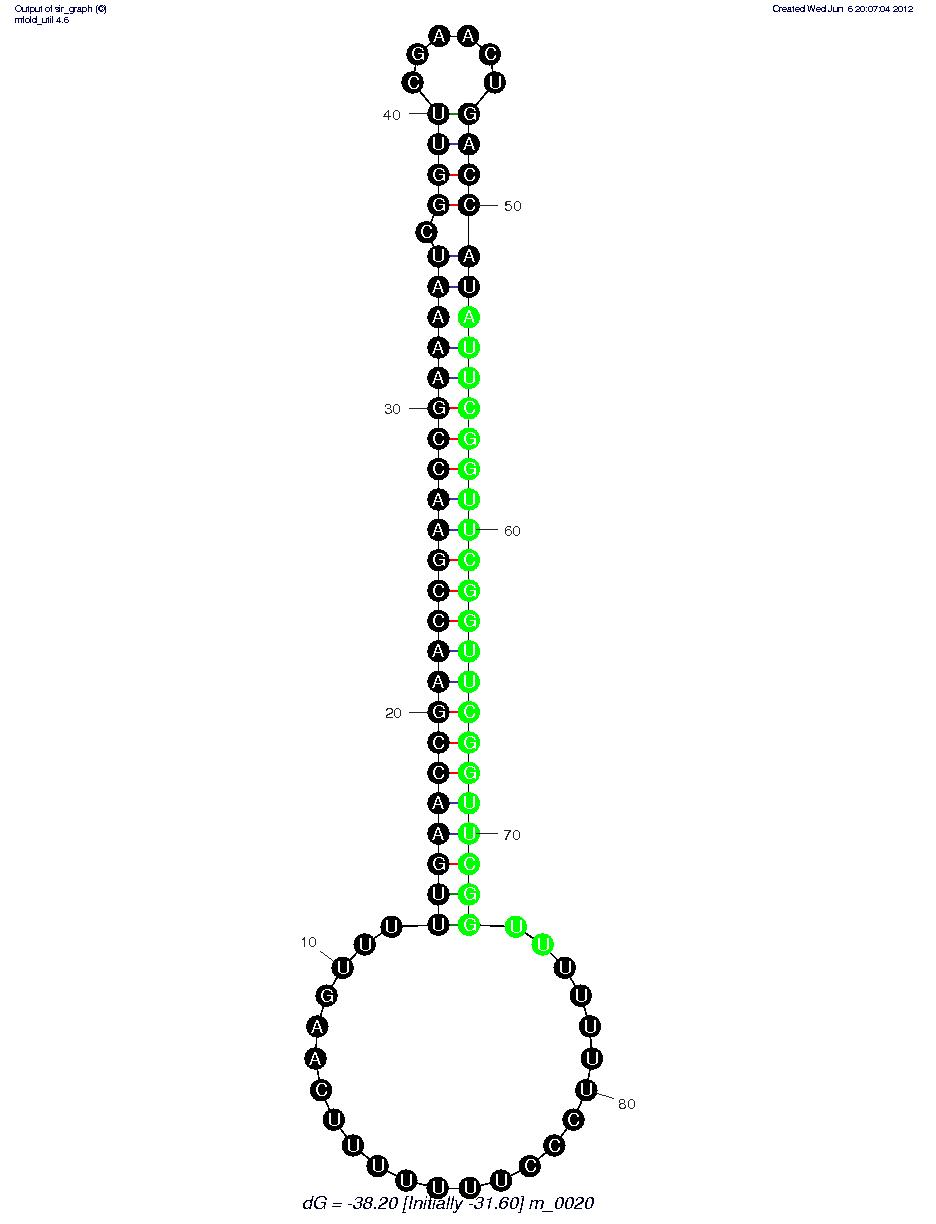 | -31.60 |
| VK1-m0021 | 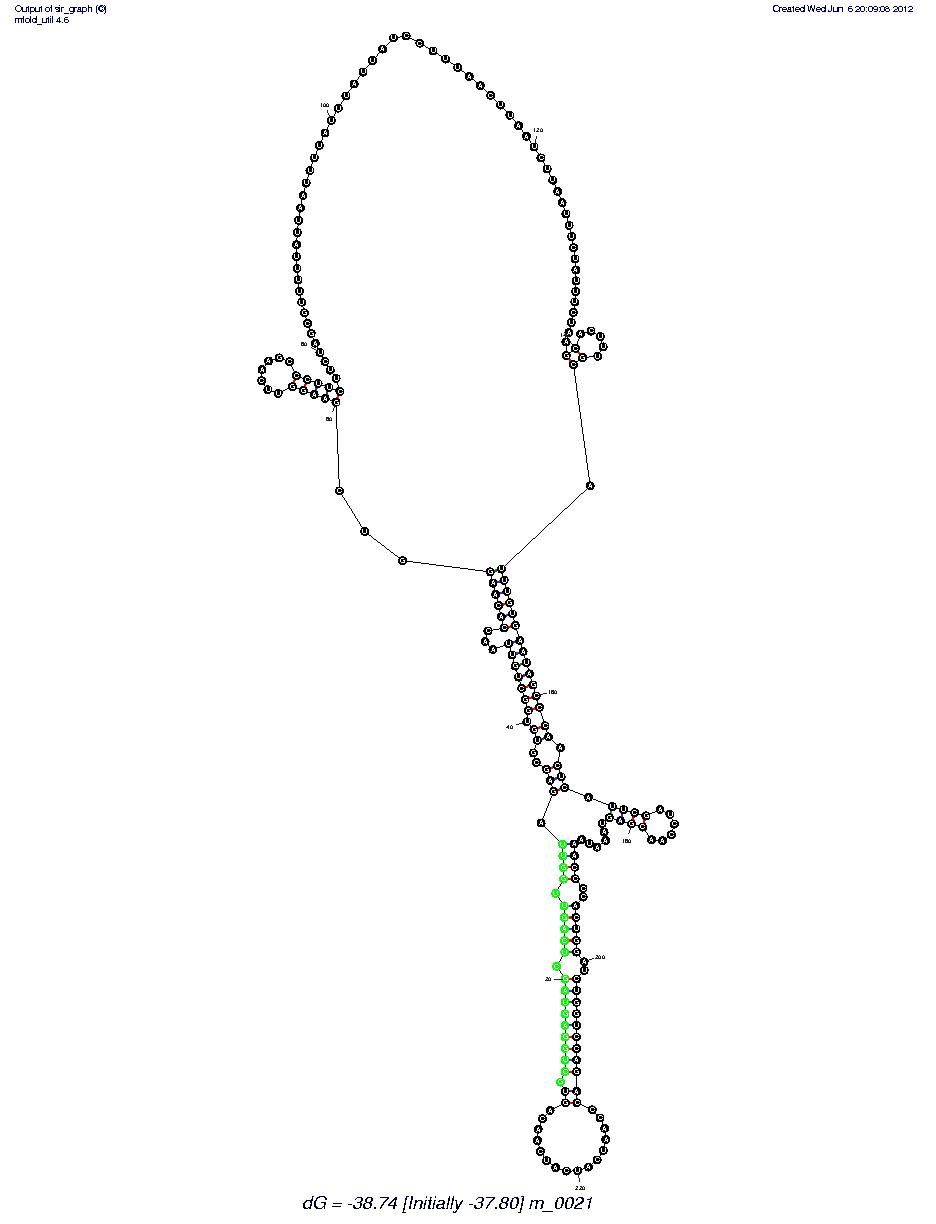 | -37.80 |
| VK1-m0022 | 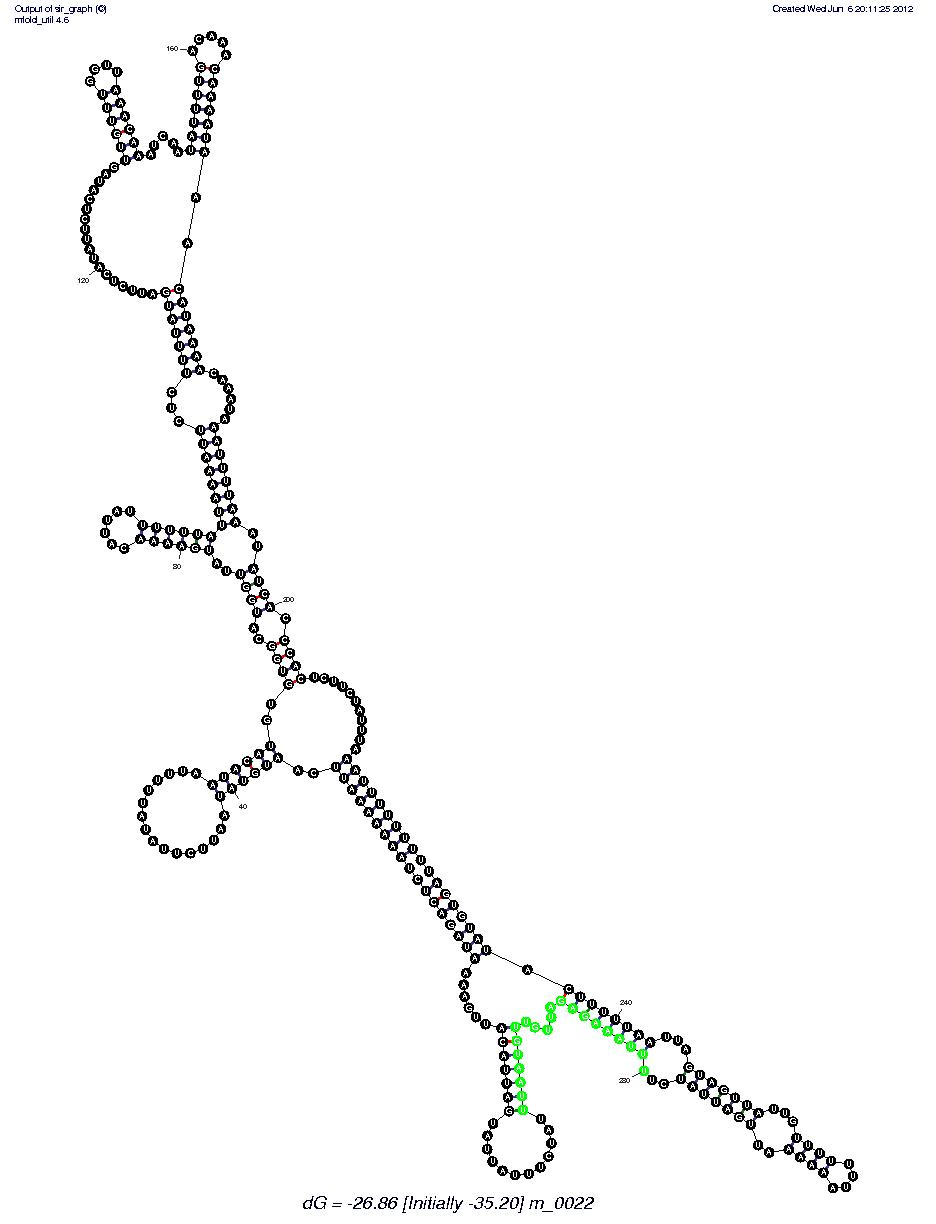 | -35.20 |
| VK1-m0023 | 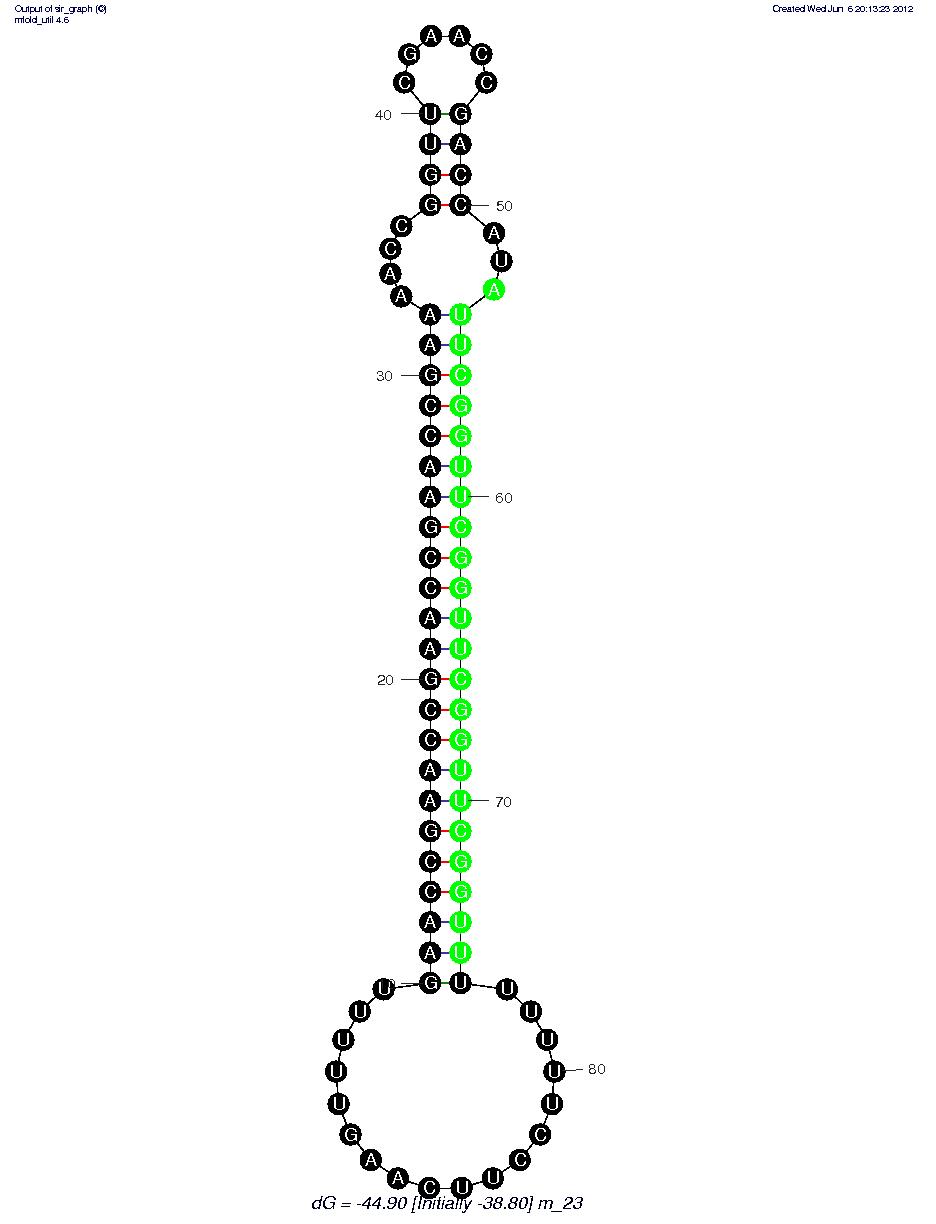 | -38.80 |
| VK1-m0024 | 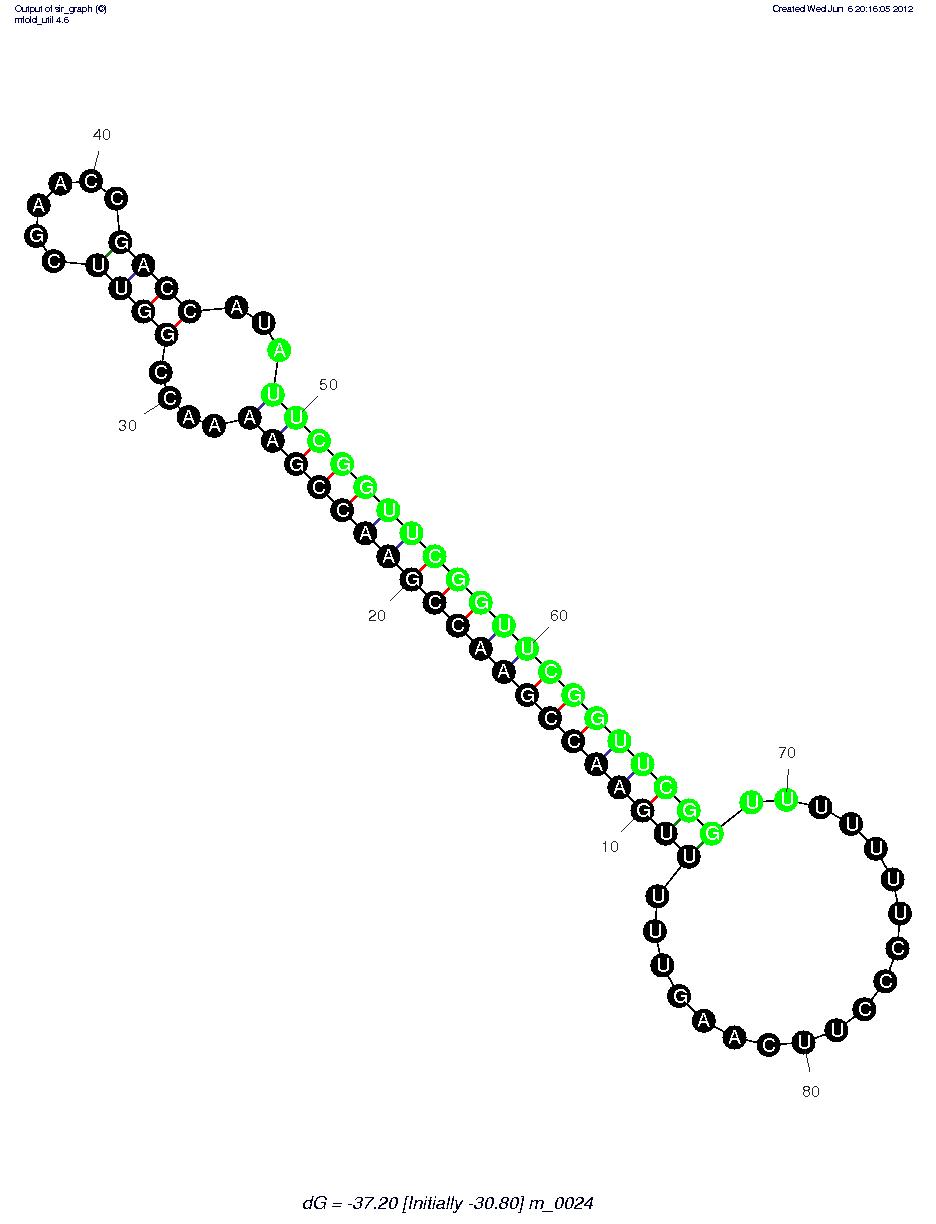 | -30.80 |
| VK1-m0025 | 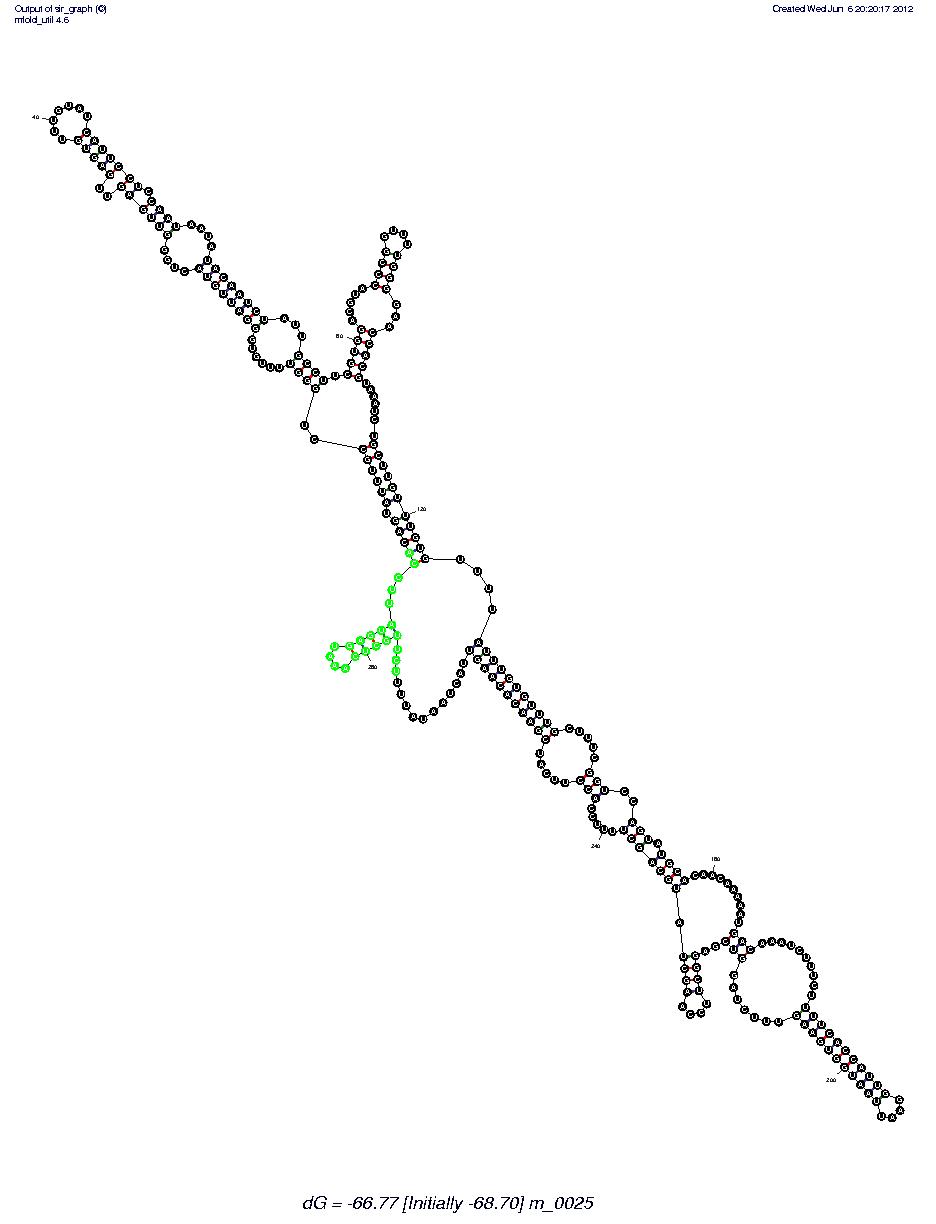 | -68.70 |
| VK1-m0026 | 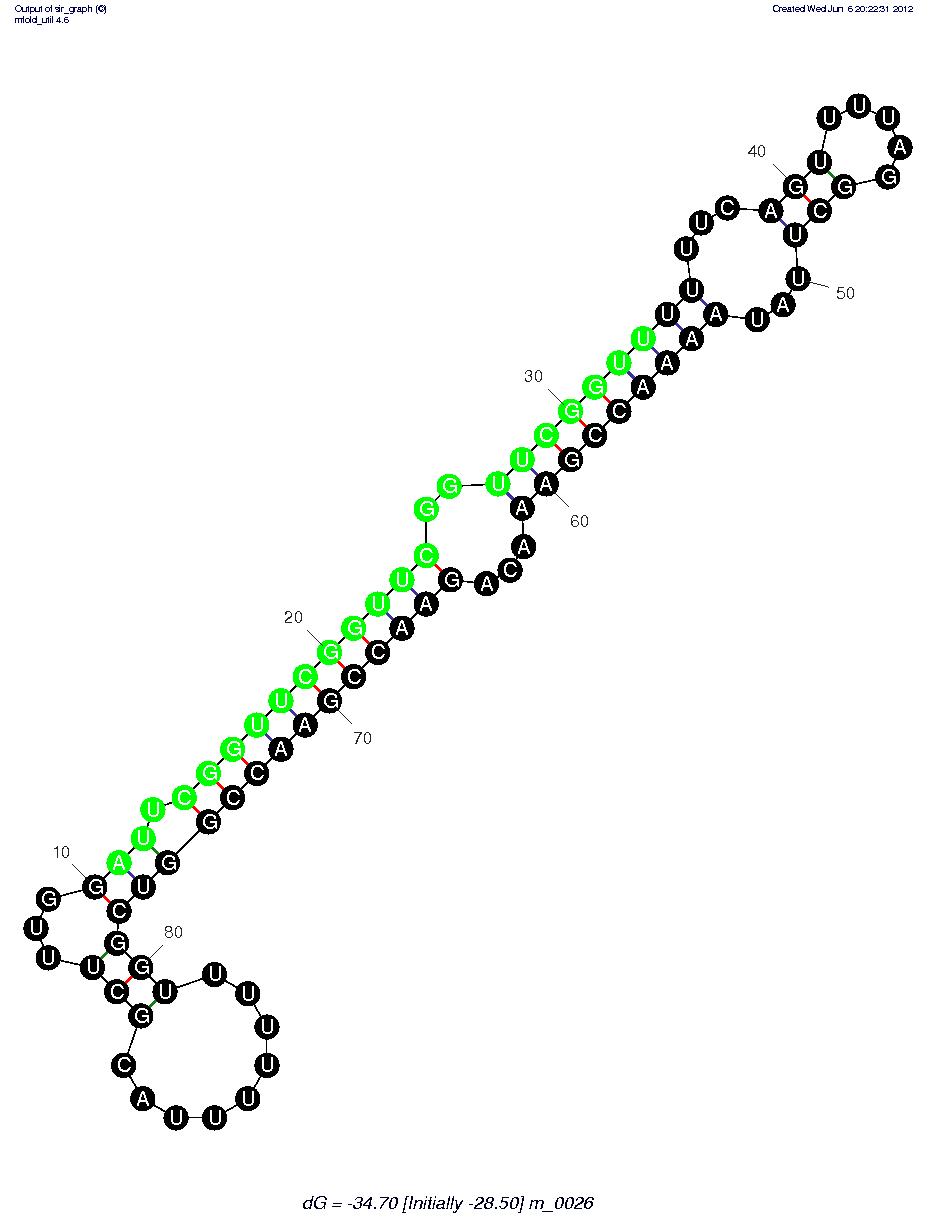 | -28.50 |
| VK1-m0027 | 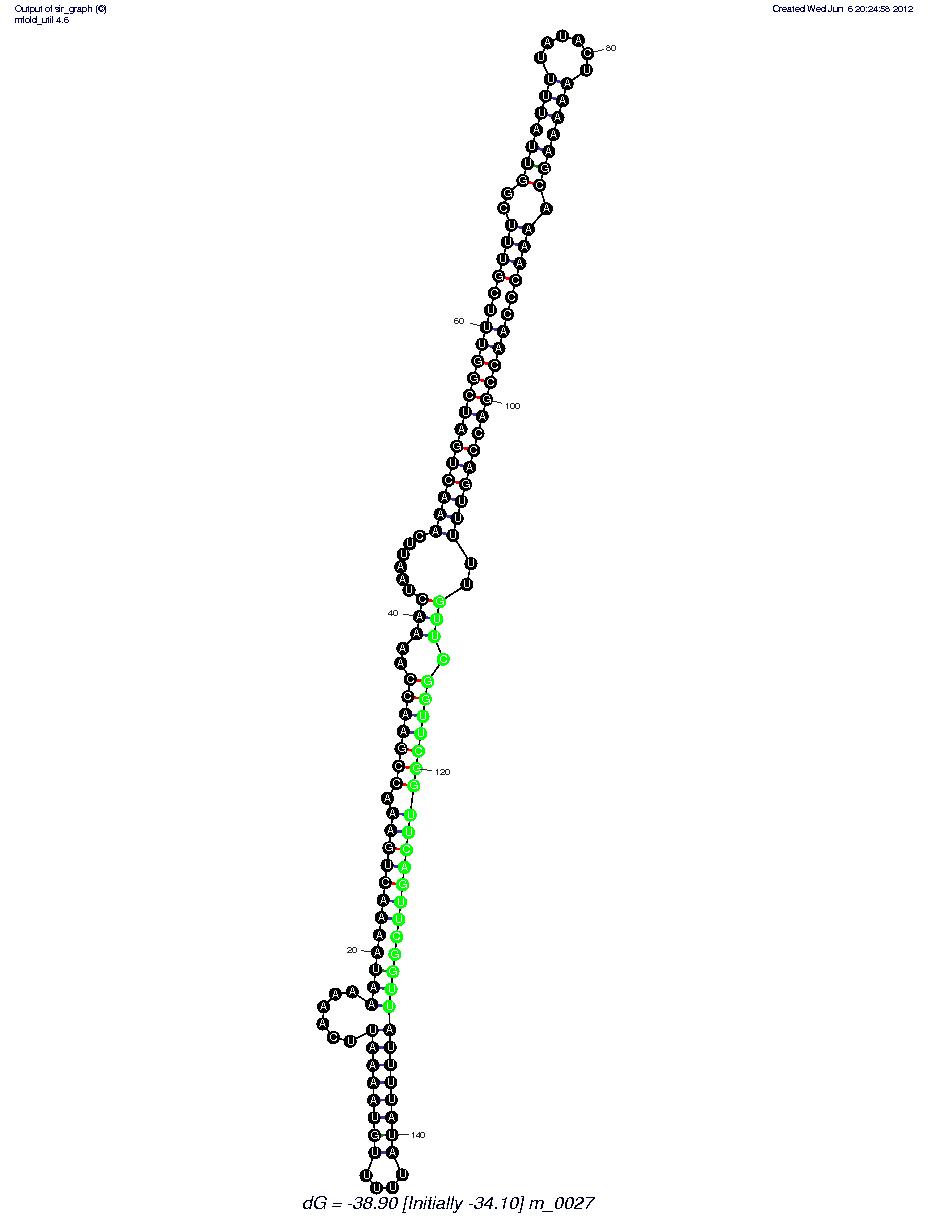 | -34.10 |
|  |  |  |
