# Supplementary material for: Genome-wide identification of alternate bearing-associated microRNAs (miRNAs) in olive (Olea europaea L.)
Source: BMC Plant Biol. 2013 Jan 15;13:10. doi: 10.1186/1471-2229-13-10 (PMC3564680; doi:10.1186/1471-2229-13-10)
Supplement: Additional file 4 — Gene ontology terms of biological function and molecular processes of miRNA targets. Members of 22 conserved miRNA families were involved in diverse biological functions and processes. [file 1471-2229-13-10-S4.doc]

**Additional file 3: Gene ontology terms of biological function and molecular processes of miRNA targets. Members of 22 conserved miRNA families were involved in diverse biological functions and processes.**

| **miRNA** | **GO biological Process** | **GO molecular function** |
| --- | --- | --- |
| **oeu-miR156a** | - | - |
| POPTR_0018s02070.1|PACid:18214742 | - | - |
| POPTR_0018s14680.1|PACid:18215570 | - | binding |
| POPTR_0018s14680.2|PACid:18215571 | - | binding |
| POPTR_0018s14680.3|PACid:18215572 | - | binding |
| POPTR_0003s16780.1|PACid:18216841 | - | binding |
| POPTR_0003s17120.1|PACid:18217293 | - | binding |
| POPTR_0014s16720.1|PACid:18222603 | metabolic process | catalytic activity, transferase activity |
| POPTR_0014s04170.1|PACid:18223645 | - | catalytic activity, transferase activity |
| POPTR_0014s05690.1|PACid:18223901 | - | binding |
| POPTR_0004s06450.1|PACid:18225055 | response to stimulus,response to stress | - |
| POPTR_0004s06450.2|PACid:18225056 | response to stimulus,response to stress | - |
| POPTR_0012s10260.1|PACid:18229814 | - | binding |
| POPTR_0011s05490.1|PACid:18232063 | - | - |
| POPTR_0015s07140.1|PACid:18232622 | - | binding |
| POPTR_0015s11100.1|PACid:18232812 | - | binding |
| POPTR_0001s13630.1|PACid:18234979 | - | - |
| POPTR_0001s08880.1|PACid:18237521 | metabolic process | binding, oxidoreductase activity |
| POPTR_0001s13890.1|PACid:18239288 | - | - |
| POPTR_0010s06830.1|PACid:18240565 | biological regulation, metabolic process, signal transduction | binding, kinase activity, transferase activity |
| POPTR_0010s16400.1|PACid:18242283 | - | binding |
| POPTR_0002s14330.1|PACid:18244437 | organismal development | binding |
| POPTR_0002s03480.1|PACid:18244598 | metabolic process | binding, transferase activity |
| POPTR_0002s03480.2|PACid:18244599 | metabolic process | binding, transferase activity |
| POPTR_0002s03480.2|PACid:18244600 | - | - |
| POPTR_0002s03480.2|PACid:18244601 | - | - |
|  |  |  |
| **oeu-miR156b** |  |  |
| POPTR_0018s02070.1|PACid:18214742 | - | - |
| POPTR_0018s14680.1|PACid:18215570 | - | binding |
| POPTR_0018s14680.2|PACid:18215571 | - | binding |
| POPTR_0018s14680.3|PACid:18215572 | - | binding |
| POPTR_0003s16780.1|PACid:18216841 | - | binding |
| POPTR_0003s17120.1|PACid:18217293 | - | binding |
| POPTR_0014s16720.1|PACid:18222603 | metabolic process | catalytic activity, transferase activity |
| POPTR_0014s04170.1|PACid:18223645 | - | catalytic activity, transferase activity |
| POPTR_0014s05690.1|PACid:18223901 | - | binding |
| POPTR_0004s06450.1|PACid:18225055 | response to stimulus, response to stress | - |
| POPTR_0004s06450.2|PACid:18225056 | response to stimulus, response to stress | - |
| POPTR_0012s10260.1|PACid:18229814 | - | binding |
| POPTR_0011s05490.1|PACid:18232063 | - | - |
| POPTR_0015s07140.1|PACid:18232622 | - | binding |
| POPTR_0015s11100.1|PACid:18232812 | - | binding |
| POPTR_0001s13630.1|PACid:18234979 | - | - |
| POPTR_0001s08880.1|PACid:18237521 | metabolic process | binding, oxidoreductase activity |
| POPTR_0001s13890.1|PACid:18239288 | - | - |
| POPTR_0010s06830.1|PACid:18240565 | biological regulation, metabolic process, signal transduction | binding, kinase activity, transferase activity |
| POPTR_0010s16400.1|PACid:18242283 | - | binding |
| POPTR_0002s14330.1|PACid:18244437 | organismal development | binding |
| POPTR_0002s03480.1|PACid:18244598 | metabolic process | binding, transferase activity |
| POPTR_0002s03480.2|PACid:18244599 | metabolic process | binding, transferase activity |
| POPTR_0008s09750.1|PACid:18247435 | - | - |
| POPTR_0016s04890.1|PACid:18249881 | organismal development, reproductive developmental process, pyhllome development | - |
|  |  |  |
| **oeu-miR156c** |  |  |
| POPTR_0018s02070.1|PACid:18214742 | - | - |
| POPTR_0018s14680.1|PACid:18215570 | - | binding |
| POPTR_0018s14680.2|PACid:18215571 | - | binding |
| POPTR_0018s14680.3|PACid:18215572 | - | binding |
| POPTR_0003s16780.1|PACid:18216841 | - | binding |
| POPTR_0003s17120.1|PACid:18217293 | - | binding |
| POPTR_0014s16720.1|PACid:18222603 | metabolic process | catalytic activity, transferase activity |
| POPTR_0014s04170.1|PACid:18223645 | - | catalytic activity, transferase activity |
| POPTR_0014s05690.1|PACid:18223901 | - | binding |
| POPTR_0004s06450.1|PACid:18225055 | response to stimulus, response to stress | - |
| POPTR_0004s06450.2|PACid:18225056 | response to stimulus, response to stress | - |
| POPTR_0012s10260.1|PACid:18229814 | - | binding |
| POPTR_0011s05490.1|PACid:18232063 | - | - |
| POPTR_0015s07140.1|PACid:18232622 | - | binding |
| POPTR_0015s11100.1|PACid:18232812 | - | binding |
| POPTR_0001s13630.1|PACid:18234979 | - | - |
| POPTR_0001s08880.1|PACid:18237521 | metabolic process | binding, oxidoreductase activity |
| POPTR_0001s13890.1|PACid:18239288 | - | - |
| POPTR_0010s06830.1|PACid:18240565 | biological regulation, metabolic process, signal transduction | binding, kinase activity, transferase activity |
| POPTR_0010s16400.1|PACid:18242283 |  | binding |
| POPTR_0002s14330.1|PACid:18244437 | organismal development | binding |
| POPTR_0002s03480.1|PACid:18244598 | metabolic process | binding, transferase activity |
| POPTR_0002s03480.2|PACid:18244599 | metabolic process | binding, transferase activity |
| POPTR_0008s09750.1|PACid:18247435 | - | - |
| POPTR_0016s04890.1|PACid:18249881 | organismal development, reproductive developmental process, pyhllome development | - |
|  |  |  |
| **oeu-miR156d** |  |  |
| POPTR_0018s02070.1|PACid:18214742 | - | - |
| POPTR_0018s14680.1|PACid:18215570 | - | binding |
| POPTR_0018s14680.2|PACid:18215571 | - | binding |
| POPTR_0018s14680.3|PACid:18215572 | - | binding |
| POPTR_0003s16780.1|PACid:18216841 | - | binding |
| POPTR_0003s17120.1|PACid:18217293 | - | binding |
| POPTR_0014s16720.1|PACid:18222603 | metabolic process | catalytic activity, transferase activity |
| POPTR_0014s04170.1|PACid:18223645 | - | catalytic activity, transferase activity |
| POPTR_0014s05690.1|PACid:18223901 | - | binding |
| POPTR_0004s06450.1|PACid:18225055 | response to stimulus, response to stress | - |
| POPTR_0004s06450.2|PACid:18225056 | response to stimulus, response to stress | - |
| POPTR_0012s10260.1|PACid:18229814 | - | binding |
| POPTR_0011s05490.1|PACid:18232063 | - | - |
| POPTR_0015s07140.1|PACid:18232622 | - | binding |
| POPTR_0015s11100.1|PACid:18232812 | - | binding |
| POPTR_0001s13630.1|PACid:18234979 | - | - |
| POPTR_0001s08880.1|PACid:18237521 | metabolic process | binding, oxidoreductase activity |
| POPTR_0001s13890.1|PACid:18239288 | - | - |
| POPTR_0010s06830.1|PACid:18240565 | biological regulation, metabolic process, signal transduction | binding, kinase activity, transferase activity |
| POPTR_0010s16400.1|PACid:18242283 | - | binding |
| POPTR_0002s14330.1|PACid:18244437 | organismal development | binding |
| POPTR_0002s03480.1|PACid:18244598 | metabolic process | binding, transferase activity |
| POPTR_0002s03480.2|PACid:18244599 | metabolic process | binding, transferase activity |
| POPTR_0008s09750.1|PACid:18247435 | - | - |
| POPTR_0016s04890.1|PACid:18249881 | organismal development, reproductive developmental process, pyhllome development | - |
|  |  |  |
| **oeu-miR156e** |  |  |
| POPTR_0018s02070.1|PACid:18214742 | - | - |
| POPTR_0018s14680.1|PACid:18215570 | - | binding |
| POPTR_0018s14680.2|PACid:18215571 | - | binding |
| POPTR_0018s14680.3|PACid:18215572 | - | binding |
| POPTR_0003s16780.1|PACid:18216841 | - | binding |
| POPTR_0003s17120.1|PACid:18217293 | - | binding |
| POPTR_0014s16720.1|PACid:18222603 | metabolic process | catalytic activity, transferase activity |
| POPTR_0014s04170.1|PACid:18223645 | - | catalytic activity, transferase activity |
| POPTR_0014s05690.1|PACid:18223901 | - | binding |
| POPTR_0004s06450.1|PACid:18225055 | response to stimulus, response to stress | - |
| POPTR_0004s06450.2|PACid:18225056 | response to stimulus, response to stress | - |
| POPTR_0012s10260.1|PACid:18229814 | - | binding |
| POPTR_0011s05490.1|PACid:18232063 | - | - |
| POPTR_0015s07140.1|PACid:18232622 | - | binding |
| POPTR_0015s11100.1|PACid:18232812 | - | binding |
| POPTR_0001s13630.1|PACid:18234979 | - | - |
| POPTR_0001s08880.1|PACid:18237521 | metabolic process | binding, oxidoreductase activity |
| POPTR_0001s13890.1|PACid:18239288 | - | - |
| POPTR_0010s06830.1|PACid:18240565 | biological regulation, metabolic process, signal transduction | binding, kinase activity, transferase activity |
| POPTR_0010s16400.1|PACid:18242283 | - | binding |
| POPTR_0002s14330.1|PACid:18244437 | organismal development | binding |
| POPTR_0002s03480.1|PACid:18244598 | metabolic process | binding, transferase activity |
| POPTR_0002s03480.2|PACid:18244599 | metabolic process | binding, transferase activity |
| POPTR_0008s09750.1|PACid:18247435 | - | - |
| POPTR_0016s04890.1|PACid:18249881 | organismal development, reproductive developmental process, pyhllome development | - |
|  |  |  |
| **oeu-miR156f** |  |  |
| POPTR_0018s02070.1|PACid:18214742 | - | - |
| POPTR_0018s14680.1|PACid:18215570 | - | binding |
| POPTR_0018s14680.2|PACid:18215571 | - | binding |
| POPTR_0018s14680.3|PACid:18215572 | - | binding |
| POPTR_0003s16780.1|PACid:18216841 | - | binding |
| POPTR_0003s17120.1|PACid:18217293 | - | binding |
| POPTR_0014s16720.1|PACid:18222603 | metabolic process | catalytic activity, transferase activity |
| POPTR_0014s04170.1|PACid:18223645 | - | catalytic activity, transferase activity |
| POPTR_0014s05690.1|PACid:18223901 | - | binding |
| POPTR_0004s06450.1|PACid:18225055 | response to stimulus, response to stress | - |
| POPTR_0004s06450.2|PACid:18225056 | response to stimulus, response to stress | - |
| POPTR_0012s10260.1|PACid:18229814 | - | binding |
| POPTR_0011s05490.1|PACid:18232063 | - | - |
| POPTR_0015s07140.1|PACid:18232622 | - | binding |
| POPTR_0015s11100.1|PACid:18232812 | - | binding |
| POPTR_0001s13630.1|PACid:18234979 | - | - |
| POPTR_0001s08880.1|PACid:18237521 | metabolic process | binding, oxidoreductase activity |
| POPTR_0001s13890.1|PACid:18239288 | - | - |
| POPTR_0010s06830.1|PACid:18240565 | biological regulation, metabolic process, signal transduction | binding, kinase activity, transferase activity |
| POPTR_0010s16400.1|PACid:18242283 | - | binding |
| POPTR_0002s14330.1|PACid:18244437 | organismal development | binding |
| POPTR_0002s03480.1|PACid:18244598 | metabolic process | binding, transferase activity |
| POPTR_0002s03480.2|PACid:18244599 | metabolic process | binding, transferase activity |
| POPTR_0008s09750.1|PACid:18247435 | - | - |
| POPTR_0016s04890.1|PACid:18249881 | organismal development, reproductive developmental process, pyhllome development | - |
|  |  |  |
| **oeu-miR156g** |  |  |
| POPTR_0017s00560.1|PACid:18209377 | cellular process, response to stimulus, response to stress | binding |
| POPTR_0017s04680.1|PACid:18209935 | cellular process, response to stimulus, response to stress | binding |
| POPTR_0017s00570.1|PACid:18210277 | cellular process, response to stimulus, response to stress | binding |
| POPTR_0073s00200.1|PACid:18210829 | metabolic process | binding, oxidoreductase activity, kinase activity, transferase activity |
| POPTR_0018s05050.1|PACid:18214988 | metabolic process | phosphatase regulator activity, enzyme regulator activity |
| POPTR_0018s05050.2|PACid:18214989 | metabolic process | phosphatase regulator activity, enzyme regulator activity |
| POPTR_0018s05050.3|PACid:18214990 | metabolic process | phosphatase regulator activity, enzyme regulator activity |
| POPTR_0018s14680.1|PACid:18215570 | - | binding |
| POPTR_0018s14680.2|PACid:18215571 | - | binding |
| POPTR_0018s14680.3|PACid:18215572 | - | binding |
| POPTR_0003s16780.1|PACid:18216841 | - | binding |
| POPTR_0003s17120.1|PACid:18217293 | - | binding |
| POPTR_0019s02760.1|PACid:18218568 | metabolic process | binding, kinase activity, transferase activity |
| POPTR_0019s02700.1|PACid:18218953 | metabolic process | binding, kinase activity, transferase activity |
| POPTR_0019s01910.1|PACid:18219603 | metabolic process | binding, oxidoreductase activity, kinase activity, transferase activity |
| POPTR_0019s02740.1|PACid:18219644 | metabolic process | - |
| POPTR_0013s08490.1|PACid:18220802 | - | binding, kinase activity, transferase activity |
| POPTR_0013s13970.1|PACid:18221077 | organismal development | - |
| POPTR_0053s00220.1|PACid:18222175 | - | binding, kinase activity, transferase activity |
| POPTR_0014s05690.1|PACid:18223901 | - | binding |
| POPTR_0012s08950.1|PACid:18229278 | transport, localization | - |
| POPTR_0012s10260.1|PACid:18229814 | - | binding |
| POPTR_0011s05490.1|PACid:18232063 | - | - |
| POPTR_0015s07140.1|PACid:18232622 | - | binding |
| POPTR_0015s11100.1|PACid:18232812 | - | binding |
| POPTR_0001s13630.1|PACid:18234979 | - | - |
| POPTR_0001s13890.1|PACid:18239288 | - | - |
| POPTR_0679s00210.1|PACid:18239430 | metabolic process | catalytic activity, hydrolase activity |
| POPTR_0010s16400.1|PACid:18242283 | - | binding |
| POPTR_0002s14330.1|PACid:18244437 | organismal development | binding |
| POPTR_0008s09750.1|PACid:18247435 | - | binding |
| POPTR_0016s04890.1|PACid:18249881 | organismal development, reproductive developmental process, pyhllome development | binding |
|  |  |  |
| **oeu-miR156h** |  |  |
| POPTR_0017s00560.1|PACid:18209377 | cellular process, response to stimulus, response to stress | binding |
| POPTR_0017s04680.1|PACid:18209935 | cellular process, response to stimulus, response to stress | binding |
| POPTR_0017s00570.1|PACid:18210277 | cellular process, response to stimulus, response to stress | binding |
| POPTR_0073s00200.1|PACid:18210829 | metabolic process | binding, oxidoreductase activity, kinase activity, transferase activity |
| POPTR_0018s05050.1|PACid:18214988 | metabolic process | phosphatase regulator activity, enzyme regulator activity |
| POPTR_0018s05050.2|PACid:18214989 | metabolic process | phosphatase regulator activity, enzyme regulator activity |
| POPTR_0018s05050.3|PACid:18214990 | metabolic process | phosphatase regulator activity, enzyme regulator activity |
| POPTR_0018s14680.1|PACid:18215570 | - | binding |
| POPTR_0018s14680.2|PACid:18215571 | - | binding |
| POPTR_0018s14680.3|PACid:18215572 | - | binding |
| POPTR_0003s16780.1|PACid:18216841 | - | binding |
| POPTR_0003s17120.1|PACid:18217293 | - | binding |
| POPTR_0019s02760.1|PACid:18218568 | metabolic process | binding, kinase activity, transferase activity |
| POPTR_0019s02700.1|PACid:18218953 | metabolic process | binding, kinase activity, transferase activity |
| POPTR_0019s01910.1|PACid:18219603 | metabolic process | binding, oxidoreductase activity, kinase activity, transferase activity |
| POPTR_0019s02740.1|PACid:18219644 | metabolic process |  |
| POPTR_0013s08490.1|PACid:18220802 |  | binding, kinase activity, transferase activity |
| POPTR_0013s13970.1|PACid:18221077 | organismal development, reproductive developmental process, pyhllome development | - |
| POPTR_0053s00220.1|PACid:18222175 | - | binding, kinase activity, transferase activity |
| POPTR_0014s05690.1|PACid:18223901 | - | binding |
| POPTR_0012s08950.1|PACid:18229278 | transport, localization | - |
| POPTR_0012s10260.1|PACid:18229814 | - | binding |
| POPTR_0011s05490.1|PACid:18232063 | - | - |
| POPTR_0015s07140.1|PACid:18232622 | - | binding |
| POPTR_0015s11100.1|PACid:18232812 | - | binding |
| POPTR_0001s13630.1|PACid:18234979 | - | - |
| POPTR_0001s13890.1|PACid:18239288 | - | - |
| POPTR_0679s00210.1|PACid:18239430 | metabolic process | catalytic activity, hydrolase activity |
| POPTR_0010s16400.1|PACid:18242283 | - | binding |
| POPTR_0002s14330.1|PACid:18244437 | organismal development | binding |
| POPTR_0008s09750.1|PACid:18247435 | - | binding |
| POPTR_0016s04890.1|PACid:18249881 | organismal development, reproductive developmental process, pyhllome development | binding |
|  |  |  |
| **oeu-miR156i** |  |  |
| POPTR_0017s00560.1|PACid:18209377 | cellular process, response to stimulus, response to stress | binding |
| POPTR_0017s04680.1|PACid:18209935 | cellular process, response to stimulus, response to stress | binding |
| POPTR_0017s00570.1|PACid:18210277 | cellular process, response to stimulus, response to stress | binding |
| POPTR_0073s00200.1|PACid:18210829 | metabolic process | binding, oxidoreductase activity, kinase activity, transferase activity |
| POPTR_0018s05050.1|PACid:18214988 | metabolic process | phosphatase regulator activity, enzyme regulator activity |
| POPTR_0018s05050.2|PACid:18214989 | metabolic process | phosphatase regulator activity, enzyme regulator activity |
| POPTR_0018s05050.3|PACid:18214990 | metabolic process | phosphatase regulator activity, enzyme regulator activity |
| POPTR_0018s14680.1|PACid:18215570 | - | binding |
| POPTR_0018s14680.2|PACid:18215571 | - | binding |
| POPTR_0018s14680.3|PACid:18215572 | - | binding |
| POPTR_0003s16780.1|PACid:18216841 | - | binding |
| POPTR_0003s17120.1|PACid:18217293 | - | binding |
| POPTR_0019s02760.1|PACid:18218568 | metabolic process | binding, kinase activity, transferase activity |
| POPTR_0019s02700.1|PACid:18218953 | metabolic process | binding, kinase activity, transferase activity |
| POPTR_0019s01910.1|PACid:18219603 | metabolic process | binding, oxidoreductase activity, kinase activity, transferase activity |
| POPTR_0019s02740.1|PACid:18219644 | metabolic process | - |
| POPTR_0013s08490.1|PACid:18220802 | - | binding, kinase activity, transferase activity |
| POPTR_0013s13970.1|PACid:18221077 | organismal development | - |
| POPTR_0053s00220.1|PACid:18222175 | - | binding, kinase activity, transferase activity |
| POPTR_0014s05690.1|PACid:18223901 | - | binding |
| POPTR_0012s08950.1|PACid:18229278 | transport, localization | - |
| POPTR_0012s10260.1|PACid:18229814 | - | binding |
| POPTR_0011s05490.1|PACid:18232063 | - | - |
| POPTR_0015s07140.1|PACid:18232622 | - | binding |
| POPTR_0015s11100.1|PACid:18232812 | - | binding |
| POPTR_0001s13630.1|PACid:18234979 | - | - |
| POPTR_0001s13890.1|PACid:18239288 | - | - |
| POPTR_0679s00210.1|PACid:18239430 | metabolic process | catalytic activity, hydrolase activity |
| POPTR_0010s16400.1|PACid:18242283 | - | binding |
| POPTR_0002s14330.1|PACid:18244437 | organismal development | binding |
| POPTR_0008s09750.1|PACid:18247435 | - | binding |
| POPTR_0016s04890.1|PACid:18249881 | organismal development, reproductive developmental process, pyhllome development | binding |
|  |  |  |
| **oeu-miR156j** |  |  |
| POPTR_0017s00560.1|PACid:18209377 | cellular process, response to stimulus, response to stress | binding |
| POPTR_0017s04680.1|PACid:18209935 | cellular process, response to stimulus, response to stress | binding |
| POPTR_0017s00570.1|PACid:18210277 | cellular process, response to stimulus, response to stress | binding |
| POPTR_0073s00200.1|PACid:18210829 | metabolic process | binding, oxidoreductase activity, kinase activity, transferase activity |
| POPTR_0018s05050.1|PACid:18214988 | metabolic process | phosphatase regulator activity, enzyme regulator activity |
| POPTR_0018s05050.2|PACid:18214989 | metabolic process | phosphatase regulator activity, enzyme regulator activity |
| POPTR_0018s05050.3|PACid:18214990 | metabolic process | phosphatase regulator activity, enzyme regulator activity |
| POPTR_0018s14680.1|PACid:18215570 | - | binding |
| POPTR_0018s14680.2|PACid:18215571 | - | binding |
| POPTR_0018s14680.3|PACid:18215572 | - | binding |
| POPTR_0003s16780.1|PACid:18216841 | - | binding |
| POPTR_0003s17120.1|PACid:18217293 | - | binding |
| POPTR_0019s02760.1|PACid:18218568 | metabolic process | binding, kinase activity, transferase activity |
| POPTR_0019s02700.1|PACid:18218953 | metabolic process | binding, kinase activity, transferase activity |
| POPTR_0019s01910.1|PACid:18219603 | metabolic process | binding, oxidoreductase activity, kinase activity, transferase activity |
| POPTR_0019s02740.1|PACid:18219644 | metabolic process | - |
| POPTR_0013s08490.1|PACid:18220802 | - | binding, kinase activity, transferase activity |
| POPTR_0013s13970.1|PACid:18221077 | organismal development | - |
| POPTR_0053s00220.1|PACid:18222175 | - | binding, kinase activity, transferase activity |
| POPTR_0014s05690.1|PACid:18223901 | - | binding |
| POPTR_0012s08950.1|PACid:18229278 | transport, localization | - |
| POPTR_0012s10260.1|PACid:18229814 | - | binding |
| POPTR_0011s05490.1|PACid:18232063 | - | - |
| POPTR_0015s07140.1|PACid:18232622 | - | binding |
| POPTR_0015s11100.1|PACid:18232812 | - | binding |
| POPTR_0001s13630.1|PACid:18234979 | - | - |
| POPTR_0001s13890.1|PACid:18239288 | - | - |
| POPTR_0679s00210.1|PACid:18239430 | metabolic process | catalytic activity, hydrolase activity |
| POPTR_0010s16400.1|PACid:18242283 | - | binding |
| POPTR_0002s14330.1|PACid:18244437 | organismal development | binding |
| POPTR_0008s09750.1|PACid:18247435 | - | binding |
| POPTR_0016s04890.1|PACid:18249881 | organismal development, reproductive developmental process, pyhllome development | binding |
|  |  |  |
| **oeu-miR156k** |  |  |
| POPTR_0006s27380.1|PACid:18212236 | - | - |
| POPTR_0018s02070.1|PACid:18214742 | - | - |
| POPTR_0018s14680.1|PACid:18215570 | - | binding |
| POPTR_0018s14680.2|PACid:18215571 | - | binding |
| POPTR_0018s14680.3|PACid:18215572 | - | binding |
| POPTR_0003s16780.1|PACid:18216841 | - | binding |
| POPTR_0003s17120.1|PACid:18217293 | - | binding |
| POPTR_0014s16720.1|PACid:18222603 | metabolic process | catalytic activity, transferase activity |
| POPTR_0014s04170.1|PACid:18223645 | - | transferase activit |
| POPTR_0014s05690.1|PACid:18223901 | - | binding |
| POPTR_0004s05340.1|PACid:18225277 | metabolic process | oxidoreductase activity, catalytic activity |
| POPTR_0009s01920.1|PACid:18227164 | biological regulation, cellular process, response to stimulus, response to stress | oxidoreductase activity, catalytic activity, transferase activity |
| POPTR_0012s06820.1|PACid:18228982 | - | - |
| POPTR_0012s10260.1|PACid:18229814 | - | binding |
| POPTR_0011s05490.1|PACid:18232063 | - | - |
| POPTR_0015s07140.1|PACid:18232622 | - | binding |
| POPTR_0015s11100.1|PACid:18232812 | - | binding |
| POPTR_0001s13630.1|PACid:18234979 | - | - |
| POPTR_0001s13890.1|PACid:18239288 | - | - |
| POPTR_0010s06830.1|PACid:18240565 | biological regulation, cellular process, response to stimulus, response to stress | binding, kinase activity, transferase activity |
| POPTR_0010s16400.1|PACid:18242283 | - | binding, nucleic acid binding |
| POPTR_0007s08110.1|PACid:18242673 | - | - |
| POPTR_0002s14330.1|PACid:18244437 | organismal development | binding |
| POPTR_0002s03340.1|PACid:18244597 | biological regulation, metabolic process, cellular response to hormone stimulus | binding, kinase activity, transferase activity |
| POPTR_0002s14150.1|PACid:18245712 | metabolic process | binding |
| POPTR_0008s09750.1|PACid:18247435 | - | - |
| POPTR_0008s16230.1|PACid:18248112 | - | - |
| POPTR_0016s04890.1|PACid:18249881 | organismal development | binding |
|  |  |  |
| **oeu-miR159a** |  |  |
| POPTR_0006s04650.1|PACid:18211314 | organismal development | binding, oxidoreductase activity, catalytic activity |
| POPTR_0006s04410.1|PACid:18211750 | biological regulation, cellular process | binding, hydrolase activity |
| POPTR_0003s18900.1|PACid:18216192 | reproductive developmental process, biological regulation, developmental process, cellular response to hormone stimulus, metabolic process | binding |
| POPTR_0003s18900.2|PACid:18216193 | reproductive developmental process, biological regulation, developmental process, cellular response to hormone stimulus, metabolic process | binding |
| POPTR_0019s11890.1|PACid:18218610 | - |  |
| POPTR_0014s05420.1|PACid:18223082 | reproductive developmental process,developmental process | binding |
| POPTR_0014s05370.1|PACid:18224032 | reproductive developmental process,developmental process | binding |
| POPTR_0009s02380.1|PACid:18227868 | - | binding |
| POPTR_0011s12790.1|PACid:18232088 | - | - |
| POPTR_0001s07330.1|PACid:18234458 | reproductive developmental process, biological regulation, developmental process, cellular response to hormone stimulus, metabolic process | binding |
| POPTR_0001s07330.2|PACid:18234459 | reproductive developmental process, biological regulation, developmental process, cellular response to hormone stimulus, metabolic process | binding |
| POPTR_0001s41950.1|PACid:18236778 | - | - |
| POPTR_0001s23170.1|PACid:18238390 | - | binding |
| POPTR_0001s38460.1|PACid:18238723 | - | - |
| POPTR_0010s16520.1|PACid:18239702 | metabolic process | ligase activity, catalytic activity |
| POPTR_0016s04340.1|PACid:18250432 | organismal development, biological regulation, metabolic process, cellular hormone metabolic process | binding, oxidoreductase activity, catalytic activity |
| POPTR_0016s14550.1|PACid:18250464 | metabolic process | binding, hydrolase activity |
|  |  |  |
| **oeu-miR159b** |  |  |
| POPTR_0006s04650.1|PACid:18211314 | organismal development, biological regulation, metabolic process, cellular hormone metabolic process | binding, oxidoreductase activity, catalytic activity |
| POPTR_0006s04410.1|PACid:18211750 | biological regulation, transcription | binding, hydrolase activity |
| POPTR_0003s18900.1|PACid:18216192 | reproductive developmental process, biological regulation, developmental process, cellular response to hormone stimulus, metabolic process | binding |
| POPTR_0003s18900.2|PACid:18216193 | reproductive developmental process, biological regulation, developmental process, cellular response to hormone stimulus, metabolic process | binding |
| POPTR_0019s11890.1|PACid:18218610 | - | - |
| POPTR_0014s05420.1|PACid:18223082 | reproductive developmental process,developmental process | binding |
| POPTR_0014s05370.1|PACid:18224032 | reproductive developmental process,developmental process | binding |
| POPTR_0009s02380.1|PACid:18227868 | - | binding |
| POPTR_0011s12790.1|PACid:18232088 | - | - |
| POPTR_0001s07330.1|PACid:18234458 | reproductive developmental process, biological regulation, developmental process, cellular response to hormone stimulus, metabolic process | binding |
| POPTR_0001s07330.2|PACid:18234459 | reproductive developmental process, biological regulation, developmental process, cellular response to hormone stimulus, metabolic process | binding |
| POPTR_0001s41950.1|PACid:18236778 | - | - |
| POPTR_0001s23170.1|PACid:18238390 | - | binding |
| POPTR_0001s38460.1|PACid:18238723 | - |  |
| POPTR_0010s16520.1|PACid:18239702 | metabolic process | ligase activity, catalytic activity |
| POPTR_0016s04340.1|PACid:18250432 | organismal development, biological regulation, metabolic process, cellular hormone metabolic process | binding, oxidoreductase activity, catalytic activity |
| POPTR_0016s14550.1|PACid:18250464 | metabolic process | binding, hydrolase activity |
|  |  |  |
| **oeu-miR159c** |  |  |
| POPTR_0006s04650.1|PACid:18211314 | organismal development, biological regulation, metabolic process, cellular hormone metabolic process | binding, oxidoreductase activity, catalytic activity |
| POPTR_0006s04410.1|PACid:18211750 | biological regulation, transcription | binding, hydrolase activity |
| POPTR_0003s18900.1|PACid:18216192 | reproductive developmental process, biological regulation, developmental process, cellular response to hormone stimulus, metabolic process | binding |
| POPTR_0003s18900.2|PACid:18216193 | reproductive developmental process, biological regulation, developmental process, cellular response to hormone stimulus, metabolic process | binding |
| POPTR_0019s11890.1|PACid:18218610 | - |  |
| POPTR_0014s05420.1|PACid:18223082 | reproductive developmental process,developmental process | binding |
| POPTR_0014s05370.1|PACid:18224032 | reproductive developmental process,developmental process | binding |
| POPTR_0009s02380.1|PACid:18227868 | - | binding |
| POPTR_0011s12790.1|PACid:18232088 | - | - |
| POPTR_0001s07330.1|PACid:18234458 | reproductive developmental process, biological regulation, developmental process, cellular response to hormone stimulus, metabolic process | binding |
| POPTR_0001s07330.2|PACid:18234459 | reproductive developmental process, biological regulation, developmental process, cellular response to hormone stimulus, metabolic process | binding |
| POPTR_0001s41950.1|PACid:18236778 | - | - |
| POPTR_0001s23170.1|PACid:18238390 | - | binding |
| POPTR_0001s38460.1|PACid:18238723 | - |  |
| POPTR_0010s16520.1|PACid:18239702 | metabolic process | ligase activity, catalytic activity |
| POPTR_0016s04340.1|PACid:18250432 | organismal development, biological regulation, metabolic process, cellular hormone metabolic process | binding, oxidoreductase activity, catalytic activity |
| POPTR_0016s14550.1|PACid:18250464 | metabolic process | binding, hydrolase activity |
|  |  |  |
| **oeu-miR159d** |  |  |
| POPTR_0003s18900.1|PACid:18216192 | reproductive developmental process, biological regulation, developmental process, cellular response to hormone stimulus, metabolic process | binding |
| POPTR_0003s18900.2|PACid:18216193 | reproductive developmental process, biological regulation, developmental process, cellular response to hormone stimulus, metabolic process | binding |
| POPTR_0014s05420.1|PACid:18223082 | reproductive developmental process,developmental process | binding |
| POPTR_0014s01760.1|PACid:18223665 | - | binding |
| POPTR_0014s05370.1|PACid:18224032 | reproductive developmental process,developmental process | binding |
| POPTR_0009s02380.1|PACid:18227868 | - | binding |
| POPTR_0011s12790.1|PACid:18232088 | - | - |
| POPTR_0001s07330.1|PACid:18234458 | reproductive developmental process, biological regulation, developmental process, cellular response to hormone stimulus, metabolic process | binding |
| POPTR_0001s07330.2|PACid:18234459 | reproductive developmental process, biological regulation, developmental process, cellular response to hormone stimulus, metabolic process | binding |
| POPTR_0001s41950.1|PACid:18236778 | - | binding |
| POPTR_0001s23170.1|PACid:18238390 | - | binding |
| POPTR_0001s38460.1|PACid:18238723 | - | binding |
| POPTR_0002s12010.1|PACid:18244970 | - | binding |
| POPTR_0008s16360.1|PACid:18249414 | - | - |
|  |  |  |
| **oeu-miR160a** |  |  |
| POPTR_0005s16320.1|PACid:18208076 | organismal development | binding |
| POPTR_0006s12930.1|PACid:18212166 | biological regulation, metabolic process, transcription, response to stimulus | binding |
| POPTR_0004s22130.1|PACid:18224673 | biological regulation, metabolic process, transcription, response to stimulus, development | binding |
| POPTR_0009s02020.1|PACid:18227573 | biological regulation, metabolic process, transcription, response to stimulus, organismal development, reproductive development | binding |
| POPTR_0009s16620.1|PACid:18228574 | - | - |
| POPTR_0010s23000.1|PACid:18240058 | biological regulation, metabolic process, cellular response to hormone stimulus, metabolic process | binding |
| POPTR_0002s09050.1|PACid:18245253 | organismal development | binding |
| POPTR_0008s03890.1|PACid:18247557 | biological regulation, metabolic process, transcription, response to stimulus, development | binding |
| POPTR_0016s09150.1|PACid:18249918 | biological regulation, metabolic process, transcription, response to stimulus, development | binding |
|  |  |  |
| **oeu-miR160b** |  |  |
| POPTR_0005s16320.1|PACid:18208076 | organismal development | binding |
| POPTR_0006s12930.1|PACid:18212166 | biological regulation, metabolic process, transcription, response to stimulus | binding |
| POPTR_0004s22130.1|PACid:18224673 | biological regulation, metabolic process, transcription, response to stimulus, development | binding |
| POPTR_0009s02020.1|PACid:18227573 | biological regulation, metabolic process, transcription, response to stimulus, organismal development, reproductive development | binding |
| POPTR_0009s16620.1|PACid:18228574 | - | - |
| POPTR_0010s23000.1|PACid:18240058 | biological regulation, metabolic process, cellular response to hormone stimulus, metabolic process | binding |
| POPTR_0002s09050.1|PACid:18245253 | organismal development | binding |
| POPTR_0008s03890.1|PACid:18247557 | biological regulation, metabolic process, transcription, response to stimulus, development | binding |
| POPTR_0016s09150.1|PACid:18249918 | biological regulation, metabolic process, transcription, response to stimulus, development | binding |
|  |  |  |
| **oeu-miR160c** |  |  |
| POPTR_0005s16320.1|PACid:18208076 | organismal development | binding |
| POPTR_0006s12930.1|PACid:18212166 | biological regulation, metabolic process, transcription, response to stimulus | binding |
| POPTR_0004s22130.1|PACid:18224673 | biological regulation, metabolic process, transcription, response to stimulus, development | binding |
| POPTR_0009s02020.1|PACid:18227573 | biological regulation, metabolic process, transcription, response to stimulus, organismal development, reproductive development | binding |
| POPTR_0009s16620.1|PACid:18228574 | - | - |
| POPTR_0010s23000.1|PACid:18240058 | biological regulation, metabolic process, cellular response to hormone stimulus, metabolic process | binding |
| POPTR_0002s09050.1|PACid:18245253 | organismal development | binding |
| POPTR_0008s03890.1|PACid:18247557 | biological regulation, metabolic process, transcription, response to stimulus, development | binding |
| POPTR_0016s09150.1|PACid:18249918 | biological regulation, metabolic process, transcription, response to stimulus, development | binding |
|  |  |  |
| **oeu-miR160d** |  |  |
| POPTR_0005s16320.1|PACid:18208076 | organismal development | binding |
| POPTR_0006s12930.1|PACid:18212166 | biological regulation, metabolic process, transcription, response to stimulus | binding |
| POPTR_0004s22130.1|PACid:18224673 | biological regulation, metabolic process, transcription, response to stimulus, development | binding |
| POPTR_0009s02020.1|PACid:18227573 | biological regulation, metabolic process, transcription, response to stimulus, organismal development, reproductive development | binding |
| POPTR_0009s16620.1|PACid:18228574 | - | - |
| POPTR_0010s23000.1|PACid:18240058 | biological regulation, metabolic process, cellular response to hormone stimulus, metabolic process | binding |
| POPTR_0002s09050.1|PACid:18245253 | organismal development | binding |
| POPTR_0008s03890.1|PACid:18247557 | biological regulation, metabolic process, transcription, response to stimulus, development | binding |
| POPTR_0016s09150.1|PACid:18249918 | biological regulation, metabolic process, transcription, response to stimulus, development | binding |
|  |  |  |
| **oeu-miR160g** |  |  |
| POPTR_0005s16320.1|PACid:18208076 | organismal development | binding |
| POPTR_0006s12930.1|PACid:18212166 | biological regulation, metabolic process, transcription, response to stimulus | binding |
| POPTR_0014s12220.1|PACid:18224067 | biological regulation, metabolic process, transcription, response to stimulus, development | - |
| POPTR_0004s22130.1|PACid:18224673 | biological regulation, metabolic process, transcription, response to stimulus, organismal development, reproductive development | binding |
| POPTR_0009s02020.1|PACid:18227573 | - | binding |
| POPTR_0010s23000.1|PACid:18240058 | biological regulation, metabolic process, cellular response to hormone stimulus, metabolic process | binding |
| POPTR_0002s09050.1|PACid:18245253 | organismal development | binding |
| POPTR_0008s03890.1|PACid:18247557 | biological regulation, metabolic process, transcription, response to stimulus, development | binding |
| POPTR_0016s09150.1|PACid:18249918 | biological regulation, metabolic process, transcription, response to stimulus, development | binding |
|  |  |  |
| **oeu-miR164a** |  |  |
| POPTR_0005s10100.1|PACid:18206764 | organismal development, reproductive developmental process, biological regulation, response to stimulus | binding |
| POPTR_0122s00210.1|PACid:18222158 | biological regulation, metabolic process, cellular response to hormone stimulus, metabolic process | binding, oxidoreductase activity,kinase activity, transferase activity |
| POPTR_0012s01610.1|PACid:18229362 | organismal process | binding |
| POPTR_0011s11600.1|PACid:18232285 | organismal development, reproductive developmental process | binding |
| POPTR_0015s02170.1|PACid:18233200 | organismal process | binding |
| POPTR_0001s40680.1|PACid:18237776 | organismal development, reproductive developmental process, biological regulation, response to stimulus | binding |
| POPTR_0007s08420.1|PACid:18242696 | organismal development, reproductive developmental process, biological regulation, response to stimulus | binding |
|  |  |  |
| **oeu-miR164b** |  |  |
| POPTR_0005s10100.1|PACid:18206764 | organismal development, reproductive developmental process, biological regulation, response to stimulus | binding |
| POPTR_0122s00210.1|PACid:18222158 | biological regulation, metabolic process, cellular response to hormone stimulus, metabolic process | binding, oxidoreductase activity,kinase activity, transferase activity |
| POPTR_0012s01610.1|PACid:18229362 | organismal process | binding |
| POPTR_0011s11600.1|PACid:18232285 | organismal development, reproductive developmental process | binding |
| POPTR_0015s02170.1|PACid:18233200 | organismal process | binding |
| POPTR_0001s40680.1|PACid:18237776 | organismal development, reproductive developmental process, biological regulation, response to stimulus | binding |
| POPTR_0007s08420.1|PACid:18242696 | organismal development, reproductive developmental process, biological regulation, response to stimulus | binding |
|  |  |  |
| **oeu-miR164c** |  |  |
| POPTR_0005s10100.1|PACid:18206764 | organismal development, reproductive developmental process, biological regulation, response to stimulus | binding |
| POPTR_0122s00210.1|PACid:18222158 | biological regulation, metabolic process, cellular response to hormone stimulus, metabolic process | binding, oxidoreductase activity,kinase activity, transferase activity |
| POPTR_0012s01610.1|PACid:18229362 | organismal process | binding |
| POPTR_0011s11600.1|PACid:18232285 | organismal development, reproductive developmental process | binding |
| POPTR_0015s02170.1|PACid:18233200 | organismal process | binding |
| POPTR_0001s40680.1|PACid:18237776 | organismal development, reproductive developmental process, biological regulation, response to stimulus | binding |
| POPTR_0007s08420.1|PACid:18242696 | organismal development, reproductive developmental process, biological regulation, response to stimulus | binding |
|  |  |  |
| **oeu-miR164d** |  |  |
| POPTR_0005s10100.1|PACid:18206764 | organismal development, reproductive developmental process, biological regulation, response to stimulus | binding |
| POPTR_0122s00210.1|PACid:18222158 | biological regulation, metabolic process, cellular response to hormone stimulus, metabolic process | binding, oxidoreductase activity,kinase activity, transferase activity |
| POPTR_0012s01610.1|PACid:18229362 | organismal process | binding |
| POPTR_0011s11600.1|PACid:18232285 | organismal development, reproductive developmental process | binding |
| POPTR_0015s02170.1|PACid:18233200 | organismal process | binding |
| POPTR_0001s40680.1|PACid:18237776 | organismal development, reproductive developmental process, biological regulation, response to stimulus | binding |
| POPTR_0007s08420.1|PACid:18242696 | organismal development, reproductive developmental process, biological regulation, response to stimulus | binding |
|  |  |  |
| **oeu-miR164e** |  |  |
| POPTR_0005s10100.1|PACid:18206764 | organismal development, reproductive developmental process, biological regulation, response to stimulus | binding |
| POPTR_0122s00210.1|PACid:18222158 | biological regulation, metabolic process, cellular response to hormone stimulus, metabolic process | binding, oxidoreductase activity,kinase activity, transferase activity |
| POPTR_0012s01610.1|PACid:18229362 | organismal process | binding |
| POPTR_0011s11600.1|PACid:18232285 | organismal development, reproductive developmental process | binding |
| POPTR_0015s02170.1|PACid:18233200 | organismal process | binding |
| POPTR_0001s40680.1|PACid:18237776 | organismal development, reproductive developmental process, biological regulation, response to stimulus | binding |
| POPTR_0007s08420.1|PACid:18242696 | organismal development, reproductive developmental process, biological regulation, response to stimulus | binding |
|  |  |  |
| **oeu-miR164f** |  |  |
| POPTR_1855s00205.1|PACid:18205975 | transport, localization | transporter activity |
| POPTR_0005s10100.1|PACid:18206764 | organismal development, reproductive developmental process, biological regulation, response to stimulus | binding |
| POPTR_0018s14860.1|PACid:18215691 | transport, localization | transporter activity |
| POPTR_0003s05520.1|PACid:18218066 | - |  |
| POPTR_0014s12360.1|PACid:18222282 | - | catalytic activity, hydrolase activity |
| POPTR_0012s01610.1|PACid:18229362 | organismal process | binding |
| POPTR_0011s11600.1|PACid:18232285 | organismal development, reproductive developmental process, pyhllome development | binding |
| POPTR_0015s02170.1|PACid:18233200 | multicellular organismal process | binding |
| POPTR_0001s40680.1|PACid:18237776 | organismal development, reproductive developmental process, pyhllome development | binding |
| POPTR_0007s08420.1|PACid:18242696 | organismal development, reproductive developmental process, biological regulation, response to stimulus | binding |
| POPTR_0002s07110.1|PACid:18246651 | - | binding |
|  |  |  |
| **oeu-miR166a** |  |  |
| POPTR_0006s25390.1|PACid:18212504 | organismal development, reproductive developmental process | binding |
| POPTR_0018s08110.1|PACid:18215654 | organismal development, reproductive developmental process | binding |
| POPTR_0003s04860.1|PACid:18216482 | organismal development, reproductive developmental process | binding |
| POPTR_0004s22090.1|PACid:18226209 | organismal development, reproductive developmental process | binding |
| POPTR_0009s01990.1|PACid:18228471 | organismal development, reproductive developmental process | binding |
| POPTR_0009s01990.2|PACid:18228472 | - | binding |
| POPTR_0001s18930.1|PACid:18234341 | - | binding |
| POPTR_0001s38120.1|PACid:18238509 | - | binding |
| POPTR_0016s06620.1|PACid:18251001 | metabolic process | binding, transporter activity |
|  |  |  |
| **oeu-miR166b** |  |  |
| POPTR_0006s25390.1|PACid:18212504 | organismal development, reproductive developmental process | binding |
| POPTR_0018s08110.1|PACid:18215654 | organismal development, reproductive developmental process | binding |
| POPTR_0003s04860.1|PACid:18216482 | organismal development, reproductive developmental process | binding |
| POPTR_0004s22090.1|PACid:18226209 | organismal development, reproductive developmental process | binding |
| POPTR_0009s01990.1|PACid:18228471 | organismal development, reproductive developmental process | binding |
| POPTR_0009s01990.2|PACid:18228472 | - | binding |
| POPTR_0001s18930.1|PACid:18234341 | - | binding |
| POPTR_0001s38120.1|PACid:18238509 | - | binding |
| POPTR_0016s06620.1|PACid:18251001 | metabolic process | binding, transporter activity |
|  |  |  |
| **oeu-miR166c** |  |  |
| POPTR_0006s25390.1|PACid:18212504 | organismal development, reproductive developmental process | binding |
| POPTR_0018s08110.1|PACid:18215654 | organismal development, reproductive developmental process | binding |
| POPTR_0003s04860.1|PACid:18216482 | organismal development, reproductive developmental process | binding |
| POPTR_0004s22090.1|PACid:18226209 | organismal development, reproductive developmental process | binding |
| POPTR_0009s01990.1|PACid:18228471 | organismal development, reproductive developmental process | binding |
| POPTR_0009s01990.2|PACid:18228472 | - | binding |
| POPTR_0001s18930.1|PACid:18234341 | - | binding |
| POPTR_0001s38120.1|PACid:18238509 | - | binding |
| POPTR_0016s06620.1|PACid:18251001 | metabolic process | binding, transporter activity |
|  |  |  |
| **oeu-miR166d** |  |  |
| POPTR_0006s25390.1|PACid:18212504 | organismal development, reproductive developmental process | binding |
| POPTR_0018s08110.1|PACid:18215654 | organismal development, reproductive developmental process | binding |
| POPTR_0003s04860.1|PACid:18216482 | organismal development, reproductive developmental process | binding |
| POPTR_0004s22090.1|PACid:18226209 | organismal development, reproductive developmental process | binding |
| POPTR_0009s01990.1|PACid:18228471 | organismal development, reproductive developmental process | binding |
| POPTR_0009s01990.2|PACid:18228472 | - | binding |
| POPTR_0001s18930.1|PACid:18234341 | - | binding |
| POPTR_0001s38120.1|PACid:18238509 | - | binding |
| POPTR_0016s06620.1|PACid:18251001 | metabolic process | binding, transporter activity |
|  |  |  |
| **oeu-miR166e** |  |  |
| POPTR_0006s25390.1|PACid:18212504 | organismal development, reproductive developmental process | binding |
| POPTR_0018s08110.1|PACid:18215654 | organismal development, reproductive developmental process | binding |
| POPTR_0003s04860.1|PACid:18216482 | organismal development, reproductive developmental process | binding |
| POPTR_0004s22090.1|PACid:18226209 | organismal development, reproductive developmental process | binding |
| POPTR_0009s01990.1|PACid:18228471 | organismal development, reproductive developmental process | binding |
| POPTR_0009s01990.2|PACid:18228472 | - | binding |
| POPTR_0001s18930.1|PACid:18234341 | - | binding |
| POPTR_0001s38120.1|PACid:18238509 | - | binding |
| POPTR_0016s06620.1|PACid:18251001 | metabolic process | binding, transporter activity |
|  |  |  |
| **oeu-miR166f** |  |  |
| POPTR_0006s25390.1|PACid:18212504 | organismal development, reproductive developmental process | binding |
| POPTR_0018s08110.1|PACid:18215654 | organismal development, reproductive developmental process | binding |
| POPTR_0003s04860.1|PACid:18216482 | organismal development, reproductive developmental process | binding |
| POPTR_0004s22090.1|PACid:18226209 | organismal development, reproductive developmental process | binding |
| POPTR_0009s01990.1|PACid:18228471 | organismal development, reproductive developmental process | binding |
| POPTR_0009s01990.2|PACid:18228472 | - | binding |
| POPTR_0001s18930.1|PACid:18234341 | - | binding |
| POPTR_0001s38120.1|PACid:18238509 | - | binding |
| POPTR_0016s06620.1|PACid:18251001 | metabolic process | binding, transporter activity |
|  |  |  |
| **oeu-miR166g** |  |  |
| POPTR_0006s25390.1|PACid:18212504 | organismal development, reproductive developmental process | binding |
| POPTR_0018s08110.1|PACid:18215654 | organismal development, reproductive developmental process | binding |
| POPTR_0003s04860.1|PACid:18216482 | organismal development, reproductive developmental process | binding |
| POPTR_0004s22090.1|PACid:18226209 | organismal development, reproductive developmental process | binding |
| POPTR_0009s01990.1|PACid:18228471 | organismal development, reproductive developmental process | binding |
| POPTR_0009s01990.2|PACid:18228472 | - | binding |
| POPTR_0001s18930.1|PACid:18234341 | - | binding |
| POPTR_0001s38120.1|PACid:18238509 | - | binding |
| POPTR_0016s06620.1|PACid:18251001 | metabolic process | binding, transporter activity |
|  |  |  |
| **oeu-miR166h** |  |  |
| POPTR_0006s25390.1|PACid:18212504 | organismal development, reproductive developmental process | binding |
| POPTR_0018s08110.1|PACid:18215654 | organismal development, reproductive developmental process | binding |
| POPTR_0003s04860.1|PACid:18216482 | organismal development, reproductive developmental process | binding |
| POPTR_0004s22090.1|PACid:18226209 | organismal development, reproductive developmental process | binding |
| POPTR_0009s01990.1|PACid:18228471 | organismal development, reproductive developmental process | binding |
| POPTR_0009s01990.2|PACid:18228472 | - | binding |
| POPTR_0001s18930.1|PACid:18234341 | - | binding |
| POPTR_0001s38120.1|PACid:18238509 | - | binding |
| POPTR_0016s06620.1|PACid:18251001 | metabolic process | binding, transporter activity |
|  |  |  |
| **oeu-miR166i** |  |  |
| POPTR_0006s25390.1|PACid:18212504 | organismal development, reproductive developmental process | binding |
| POPTR_0018s08110.1|PACid:18215654 | organismal development, reproductive developmental process | binding |
| POPTR_0003s04860.1|PACid:18216482 | organismal development, reproductive developmental process | binding |
| POPTR_0004s22090.1|PACid:18226209 | organismal development, reproductive developmental process | binding |
| POPTR_0009s01990.1|PACid:18228471 | organismal development, reproductive developmental process | binding |
| POPTR_0009s01990.2|PACid:18228472 | - | binding |
| POPTR_0001s18930.1|PACid:18234341 | - | binding |
| POPTR_0001s38120.1|PACid:18238509 | - | binding |
| POPTR_0016s06620.1|PACid:18251001 | metabolic process | binding, transporter activity |
|  |  |  |
| **oeu-miR166j** |  |  |
| POPTR_0006s25390.1|PACid:18212504 | organismal development, reproductive developmental process | binding |
| POPTR_0018s08110.1|PACid:18215654 | organismal development, reproductive developmental process | binding |
| POPTR_0003s04860.1|PACid:18216482 | organismal development, reproductive developmental process | binding |
| POPTR_0004s22090.1|PACid:18226209 | organismal development, reproductive developmental process | binding |
| POPTR_0009s01990.1|PACid:18228471 | organismal development, reproductive developmental process | binding |
| POPTR_0009s01990.2|PACid:18228472 | - | binding |
| POPTR_0001s18930.1|PACid:18234341 | - | binding |
| POPTR_0001s38120.1|PACid:18238509 | - | binding |
| POPTR_0016s06620.1|PACid:18251001 | metabolic process | binding, transporter activity |
|  |  |  |
| **oeu-miR166k** |  |  |
| POPTR_0006s25390.1|PACid:18212504 | organismal development, reproductive developmental process | binding |
| POPTR_0018s08110.1|PACid:18215654 | organismal development, reproductive developmental process | binding |
| POPTR_0003s04860.1|PACid:18216482 | organismal development, reproductive developmental process | binding |
| POPTR_0004s22090.1|PACid:18226209 | organismal development, reproductive developmental process | binding |
| POPTR_0009s01990.1|PACid:18228471 | organismal development, reproductive developmental process | binding |
| POPTR_0009s01990.2|PACid:18228472 | - | binding |
| POPTR_0001s18930.1|PACid:18234341 | - | binding |
| POPTR_0001s38120.1|PACid:18238509 | - | binding |
| POPTR_0016s06620.1|PACid:18251001 | metabolic process | binding, transporter activity |
|  |  |  |
| **oeu-miR166l** |  |  |
| POPTR_0006s25390.1|PACid:18212504 | organismal development, reproductive developmental process | binding |
| POPTR_0018s08110.1|PACid:18215654 | organismal development, reproductive developmental process | binding |
| POPTR_0003s04860.1|PACid:18216482 | organismal development, reproductive developmental process | binding |
| POPTR_0004s22090.1|PACid:18226209 | organismal development, reproductive developmental process | binding |
| POPTR_0009s01990.1|PACid:18228471 | organismal development, reproductive developmental process | binding |
| POPTR_0009s01990.2|PACid:18228472 | - | binding |
| POPTR_0001s18930.1|PACid:18234341 | - | binding |
| POPTR_0001s38120.1|PACid:18238509 | - | binding |
| POPTR_0016s06620.1|PACid:18251001 | metabolic process | binding, transporter activity |
|  |  |  |
| **oeu-miR166m** |  |  |
| POPTR_0006s25390.1|PACid:18212504 | organismal development, reproductive developmental process | binding |
| POPTR_0018s08110.1|PACid:18215654 | organismal development, reproductive developmental process | binding |
| POPTR_0003s04860.1|PACid:18216482 | organismal development, reproductive developmental process | binding |
| POPTR_0004s22090.1|PACid:18226209 | organismal development, reproductive developmental process | binding |
| POPTR_0009s01990.1|PACid:18228471 | organismal development, reproductive developmental process | binding |
| POPTR_0009s01990.2|PACid:18228472 | - | binding |
| POPTR_0001s18930.1|PACid:18234341 | - | binding |
| POPTR_0001s38120.1|PACid:18238509 | - | binding |
| POPTR_0016s06620.1|PACid:18251001 | metabolic process | binding, transporter activity |
|  |  |  |
| **oeu-miR166n** |  |  |
| POPTR_0006s25390.1|PACid:18212504 | organismal development, reproductive developmental process | binding |
| POPTR_0018s08110.1|PACid:18215654 | organismal development, reproductive developmental process | binding |
| POPTR_0003s04860.1|PACid:18216482 | organismal development, reproductive developmental process | binding |
| POPTR_0003s02840.1|PACid:18217566 | metabolic process | binding, protease activity |
| POPTR_0004s22090.1|PACid:18226209 | organismal development, reproductive developmental process, biological regulation, response to stimulus | binding |
| POPTR_0009s06380.1|PACid:18227080 | metabolic process | binding, oxidoreductase activity |
| POPTR_0009s01990.1|PACid:18228471 | organismal development, reproductive developmental process | binding |
| POPTR_0009s01990.2|PACid:18228472 | - | binding |
| POPTR_0001s20410.1|PACid:18234226 | metabolic process | binding, protease activity |
| POPTR_0001s18930.1|PACid:18234341 | - | binding |
| POPTR_0001s27120.1|PACid:18237533 | metabolic process | binding, oxidoreductase activity |
| POPTR_0001s38120.1|PACid:18238509 | - | binding |
|  |  |  |
| **oeu-miR166o** |  |  |
| POPTR_0006s25390.1|PACid:18212504 | organismal development, reproductive developmental process | binding |
| POPTR_0018s08110.1|PACid:18215654 | organismal development, reproductive developmental process | binding |
| POPTR_0003s04860.1|PACid:18216482 | organismal development, reproductive developmental process | binding |
| POPTR_0003s02840.1|PACid:18217566 | metabolic process | binding, protease activity |
| POPTR_0004s22090.1|PACid:18226209 | organismal development, reproductive developmental process | binding |
| POPTR_0009s06380.1|PACid:18227080 | metabolic process | binding, oxidoreductase activity |
| POPTR_0009s01990.1|PACid:18228471 | organismal development, reproductive developmental process | binding |
| POPTR_0009s01990.2|PACid:18228472 | - | binding |
| POPTR_0001s20410.1|PACid:18234226 | metabolic process | binding, protease activity |
| POPTR_0001s18930.1|PACid:18234341 | - | binding |
| POPTR_0001s27120.1|PACid:18237533 | metabolic process | binding, oxidoreductase activity |
| POPTR_0001s38120.1|PACid:18238509 | - | binding |
|  |  |  |
| **oeu-miR166p** |  |  |
| POPTR_0003s04860.1|PACid:18216482 | organismal development, reproductive developmental process | binding |
| POPTR_0004s22090.1|PACid:18226209 | organismal development, reproductive developmental process | binding |
| POPTR_0001s18930.1|PACid:18234341 | - | binding |
| POPTR_0007s04520.1|PACid:18243185 | biological regulation, metabolic process | binding, peptidase activity, catalytic activity, hydrolase activity |
| POPTR_0007s04510.1|PACid:18243374 | biological regulation, metabolic process | binding, peptidase activity, catalytic activity, hydrolase activity |
| POPTR_0007s04510.2|PACid:18243375 | biological regulation, metabolic process | binding, peptidase activity, catalytic activity, hydrolase activity |
|  |  |  |
| **oeu-miR166q** |  |  |
| POPTR_0006s25390.1|PACid:18212504 | organismal development, reproductive developmental process | binding |
| POPTR_0018s08110.1|PACid:18215654 | organismal development, reproductive developmental process | binding |
| POPTR_0003s04860.1|PACid:18216482 | organismal development, reproductive developmental process | binding |
| POPTR_0003s02840.1|PACid:18217566 | metabolic process | binding, protease activity |
| POPTR_0004s22090.1|PACid:18226209 | organismal development, reproductive developmental process | binding |
| POPTR_0009s06380.1|PACid:18227080 | metabolic process | binding, oxidoreductase activity |
| POPTR_0009s01990.1|PACid:18228471 | organismal development, reproductive developmental process | binding |
| POPTR_0009s01990.2|PACid:18228472 | - | binding |
| POPTR_0001s20410.1|PACid:18234226 | metabolic process | binding, protease activity |
| POPTR_0001s18930.1|PACid:18234341 | - | binding |
| POPTR_0001s27120.1|PACid:18237533 | metabolic process | binding, oxidoreductase activity |
| POPTR_0001s38120.1|PACid:18238509 | - | binding |
|  |  |  |
| **oeu-miR167a** |  |  |
| POPTR_0005s22930.1|PACid:18207021 | organismal development, biological regulation | binding |
| POPTR_0017s01870.1|PACid:18210635 | organismal development, biological regulation | binding |
| POPTR_0014s10960.1|PACid:18222770 | metabolic process | - |
| POPTR_0004s07640.1|PACid:18226423 | metabolic process | binding |
| POPTR_0009s00910.1|PACid:18228404 | - | - |
| POPTR_0012s09730.1|PACid:18229392 | - | - |
| POPTR_0011s09450.1|PACid:18230518 | organismal development, biological regulation | binding |
| POPTR_0001s36900.1|PACid:18235329 | organismal development, biological regulation | binding |
| POPTR_0002s05590.1|PACid:18244831 | organismal development, biological regulation | binding |
| POPTR_0008s07550.1|PACid:18249047 | - | - |
|  |  |  |
| **oeu-miR167b** |  |  |
| POPTR_0005s22930.1|PACid:18207021 | organismal development, biological regulation | binding |
| POPTR_0017s01870.1|PACid:18210635 | organismal development, biological regulation | binding |
| POPTR_0014s10960.1|PACid:18222770 | metabolic process | - |
| POPTR_0004s07640.1|PACid:18226423 | organismal development, biological regulation | binding |
| POPTR_0009s00910.1|PACid:18228404 | - | - |
| POPTR_0012s09730.1|PACid:18229392 | - | - |
| POPTR_0011s09450.1|PACid:18230518 | organismal development, biological regulation | binding |
| POPTR_0001s36900.1|PACid:18235329 | organismal development, biological regulation | binding |
| POPTR_0002s05590.1|PACid:18244831 | organismal development, biological regulation | binding |
| POPTR_0008s07550.1|PACid:18249047 | - | - |
|  |  |  |
| **oeu-miR167c** |  |  |
| POPTR_0005s22930.1|PACid:18207021 | organismal development, biological regulation | binding |
| POPTR_0017s01870.1|PACid:18210635 | organismal development, biological regulation | binding |
| POPTR_0014s10960.1|PACid:18222770 | metabolic process | - |
| POPTR_0004s07640.1|PACid:18226423 | organismal development, biological regulation | binding |
| POPTR_0009s00910.1|PACid:18228404 | - | - |
| POPTR_0012s09730.1|PACid:18229392 | - | - |
| POPTR_0011s09450.1|PACid:18230518 | organismal development, biological regulation | binding |
| POPTR_0001s36900.1|PACid:18235329 | organismal development, biological regulation | binding |
| POPTR_0002s05590.1|PACid:18244831 | organismal development, biological regulation | binding |
| POPTR_0008s07550.1|PACid:18249047 | - | - |
|  |  |  |
| **oeu-miR167d** |  |  |
| POPTR_0005s22930.1|PACid:18207021 | organismal development, biological regulation | binding |
| POPTR_0017s01870.1|PACid:18210635 | organismal development, biological regulation | binding |
| POPTR_0014s10960.1|PACid:18222770 | metabolic process | - |
| POPTR_0004s07640.1|PACid:18226423 | organismal development, biological regulation | binding |
| POPTR_0009s00910.1|PACid:18228404 | - | - |
| POPTR_0012s09730.1|PACid:18229392 | - | - |
| POPTR_0011s09450.1|PACid:18230518 | organismal development, biological regulation | binding |
| POPTR_0001s36900.1|PACid:18235329 | organismal development, biological regulation | binding |
| POPTR_0002s05590.1|PACid:18244831 | organismal development, biological regulation | binding |
| POPTR_0008s07550.1|PACid:18249047 | - | - |
|  |  |  |
| **oeu-miR167e** |  |  |
| POPTR_0005s22930.1|PACid:18207021 | organismal development, biological regulation | binding |
| POPTR_0017s01870.1|PACid:18210635 | organismal development, biological regulation | binding |
| POPTR_0013s14710.1|PACid:18221644 | metabolic process | binding, kinase activity, transferase activity |
| POPTR_0004s07640.1|PACid:18226423 | organismal development, biological regulation | binding |
| POPTR_0011s09450.1|PACid:18230518 | organismal development, biological regulation | binding |
| POPTR_0001s36900.1|PACid:18235329 | organismal development, biological regulation | binding |
| POPTR_0010s19710.1|PACid:18241232 | organismal development, biological regulation | binding, oxidoreductase activity, catalytic activity |
| POPTR_0010s19710.2|PACid:18241233 | organismal development, biological regulation | binding, oxidoreductase activity, catalytic activity |
| POPTR_0010s19710.3|PACid:18241234 | organismal development, biological regulation | binding, oxidoreductase activity, catalytic activity |
| POPTR_0010s19710.4|PACid:18241235 | organismal development, biological regulation | binding, oxidoreductase activity, catalytic activity |
| POPTR_0010s19710.5|PACid:18241236 | organismal development, biological regulation | binding, oxidoreductase activity, catalytic activity |
| POPTR_0002s05590.1|PACid:18244831 | organismal development, biological regulation | binding |
| POPTR_0008s10630.1|PACid:18248727 | - | - |
| POPTR_0008s07550.1|PACid:18249047 | - | - |
| POPTR_0016s09910.1|PACid:18250342 | metabolic process | - |
|  |  |  |
| **oeu-miR167f** |  |  |
| POPTR_0005s22930.1|PACid:18207021 | organismal development, biological regulation | binding |
| POPTR_0005s27310.1|PACid:18208000 | - | - |
| POPTR_0017s01870.1|PACid:18210635 | organismal development, biological regulation | binding |
| POPTR_0003s09330.1|PACid:18217548 | metabolic process | phosphatase activity, hydrolase activity |
| POPTR_0004s18730.1|PACid:18225776 | metabolic process | binding, oxidoreductase activity |
| POPTR_0004s07640.1|PACid:18226423 | organismal development, biological regulation | binding |
| POPTR_0012s14630.1|PACid:18229939 | unannotated | catalytic activity, hydrolase activity |
| POPTR_0011s09450.1|PACid:18230518 | organismal development, biological regulation | binding |
| POPTR_0001s36900.1|PACid:18235329 | organismal development, biological regulation | binding |
| POPTR_0010s05990.1|PACid:18241768 | metabolic process | binding, oxidoreductase activity |
| POPTR_0002s01060.1|PACid:18244823 | - | - |
| POPTR_0002s05590.1|PACid:18244831 | organismal development, biological regulation | binding |
| POPTR_0008s18370.1|PACid:18248580 | metabolic process | binding, oxidoreductase activity |
| POPTR_0008s07550.1|PACid:18249047 | - | - |
|  |  |  |
| **oeu-miR167g** |  |  |
| POPTR_0005s22930.1|PACid:18207021 | organismal development, biological regulation | binding |
| POPTR_0005s27310.1|PACid:18208000 | - | - |
| POPTR_0017s01870.1|PACid:18210635 | organismal development, biological regulation | binding |
| POPTR_0003s09330.1|PACid:18217548 | metabolic process | phosphatase activity, hydrolase activity, hydrolase activity |
| POPTR_0004s18730.1|PACid:18225776 | metabolic process | binding |
| POPTR_0004s07640.1|PACid:18226423 | organismal development, biological regulation | binding |
| POPTR_0012s14630.1|PACid:18229939 | unannotated | catalytic activity, hydrolase activity, hydrolase activity |
| POPTR_0011s09450.1|PACid:18230518 | organismal development, biological regulation | binding |
| POPTR_0001s36900.1|PACid:18235329 | organismal development, biological regulation | binding |
| POPTR_0010s05990.1|PACid:18241768 | metabolic process | binding, oxidoreductase activity |
| POPTR_0002s01060.1|PACid:18244823 | - | - |
| POPTR_0002s05590.1|PACid:18244831 | organismal development, biological regulation | binding |
| POPTR_0008s18370.1|PACid:18248580 | metabolic process | binding, oxidoreductase activity |
| POPTR_0008s07550.1|PACid:18249047 | - | - |
|  |  |  |
| **oeu-miR168a** |  |  |
| POPTR_0006s02840.1|PACid:18213128 | organismal development, biological regulation | binding |
| POPTR_0003s03740.1|PACid:18216759 | - | - |
| POPTR_0003s15310.2|PACid:18217618 | - | - |
| POPTR_1788s00200.1|PACid:18227034 | - | - |
| POPTR_0012s03410.1|PACid:18229530 | organismal development, biological regulation | binding |
| POPTR_0015s05550.1|PACid:18233722 | organismal development, biological regulation | binding |
| POPTR_0010s16510.1|PACid:18240954 | - | - |
|  |  |  |
| **oeu-miR168b** |  |  |
| POPTR_0006s02840.1|PACid:18213128 | metabolic process | binding |
| POPTR_0003s03740.1|PACid:18216759 | - | - |
| POPTR_0003s15310.2|PACid:18217618 | - | - |
| POPTR_1788s00200.1|PACid:18227034 | - | - |
| POPTR_0012s03410.1|PACid:18229530 | organismal development, biological regulation | binding |
| POPTR_0015s05550.1|PACid:18233722 | organismal development, biological regulation | binding |
| POPTR_0010s16510.1|PACid:18240954 | - | - |
|  |  |  |
| **oeu-miR169a** |  |  |
| POPTR_0017s07680.1|PACid:18209595 | - | binding |
| POPTR_0007s10230.1|PACid:18243277 | - | - |
| POPTR_0002s12590.1|PACid:18244421 | - | binding |
| POPTR_0002s12590.2|PACid:18244422 | - | binding |
| POPTR_0002s13590.1|PACid:18244756 | transport, localization | binding, transporter activity |
|  |  |  |
| **oeu-miR169b** |  |  |
| POPTR_0017s07680.1|PACid:18209595 | - | binding |
| POPTR_0007s10230.1|PACid:18243277 | - | - |
| POPTR_0002s12590.1|PACid:18244421 | - | binding |
| POPTR_0002s12590.2|PACid:18244422 | - | binding |
| POPTR_0002s13590.1|PACid:18244756 | transport, localization | binding, transporter activity |
|  |  |  |
| **oeu-miR169c** |  |  |
| POPTR_0017s07680.1|PACid:18209595 | - | binding |
| POPTR_0007s10230.1|PACid:18243277 | - | - |
| POPTR_0002s12590.1|PACid:18244421 | - | binding |
| POPTR_0002s12590.2|PACid:18244422 | - | binding |
| POPTR_0002s13590.1|PACid:18244756 | transport, localization | binding, transporter activity |
|  |  |  |
| **oeu-miR169d** |  |  |
| POPTR_0017s11180.1|PACid:18209986 | - | - |
| POPTR_0009s06540.1|PACid:18227630 | - | - |
|  |  |  |
| **oeu-miR169e** |  |  |
| POPTR_0017s11180.1|PACid:18209986 | - | - |
| POPTR_0009s06540.1|PACid:18227630 | - | - |
|  |  |  |
| **oeu-miR169f** |  |  |
| POPTR_0017s11180.1|PACid:18209986 | - | - |
| POPTR_0009s06540.1|PACid:18227630 | - | - |
|  |  |  |
| **oeu-miR169g** |  |  |
| POPTR_0017s11180.1|PACid:18209986 | - | - |
| POPTR_0009s06540.1|PACid:18227630 | - | - |
|  |  |  |
| **oeu-miR169h** |  |  |
| POPTR_0017s11180.1|PACid:18209986 | - | - |
| POPTR_0009s06540.1|PACid:18227630 | - | - |
|  |  |  |
| **oeu-miR169i** |  |  |
| POPTR_0017s11180.1|PACid:18209986 | - | - |
| POPTR_0475s00210.1|PACid:18220173 | - | - |
| POPTR_0475s00210.2|PACid:18220174 | - | - |
| POPTR_0009s06540.1|PACid:18227630 | - | - |
| POPTR_0010s22300.1|PACid:18241447 | - | - |
| POPTR_0010s24370.1|PACid:18241829 | - | - |
| POPTR_0002s18480.1|PACid:18244367 | - | - |
| POPTR_0008s02300.1|PACid:18249071 | - | - |
|  |  |  |
| **oeu-miR169j** |  | - |
| POPTR_0017s11180.1|PACid:18209986 | - | - |
| POPTR_0475s00210.1|PACid:18220173 | - | - |
| POPTR_0475s00210.2|PACid:18220174 | - | - |
| POPTR_0009s06540.1|PACid:18227630 | - | - |
| POPTR_0010s22300.1|PACid:18241447 | - | - |
| POPTR_0010s24370.1|PACid:18241829 | - | - |
| POPTR_0002s18480.1|PACid:18244367 | - | - |
| POPTR_0008s02300.1|PACid:18249071 | - | - |
|  |  |  |
| **oeu-miR169k** |  |  |
| POPTR_0017s11180.1|PACid:18209986 | - | - |
| POPTR_0475s00210.1|PACid:18220173 | - | - |
| POPTR_0475s00210.2|PACid:18220174 | - | - |
| POPTR_0009s06540.1|PACid:18227630 | - | - |
| POPTR_0010s22300.1|PACid:18241447 | - | - |
| POPTR_0010s24370.1|PACid:18241829 | - | - |
| POPTR_0002s18480.1|PACid:18244367 | - | - |
| POPTR_0008s02300.1|PACid:18249071 | - | - |
|  |  |  |
| **oeu-miR169l** |  |  |
| POPTR_0017s11180.1|PACid:18209986 | - | - |
| POPTR_0475s00210.1|PACid:18220173 | - | - |
| POPTR_0475s00210.2|PACid:18220174 | - | - |
| POPTR_0009s06540.1|PACid:18227630 | - | - |
| POPTR_0010s22300.1|PACid:18241447 | - | - |
| POPTR_0010s24370.1|PACid:18241829 | - | - |
| POPTR_0002s18480.1|PACid:18244367 | - | - |
| POPTR_0008s02300.1|PACid:18249071 | - | - |
|  |  |  |
| **oeu-miR169m** |  |  |
| POPTR_0017s11180.1|PACid:18209986 | - | - |
| POPTR_0475s00210.1|PACid:18220173 | - | - |
| POPTR_0475s00210.2|PACid:18220174 | - | - |
| POPTR_0009s06540.1|PACid:18227630 | - | - |
| POPTR_0010s22300.1|PACid:18241447 | - | - |
| POPTR_0010s24370.1|PACid:18241829 | - | - |
| POPTR_0002s18480.1|PACid:18244367 | - | - |
| POPTR_0008s02300.1|PACid:18249071 | - | - |
|  |  |  |
| **oeu-miR169r** |  |  |
| POPTR_0017s11180.1|PACid:18209986 | - | - |
| POPTR_0018s00900.1|PACid:18214285 | - | - |
| POPTR_0475s00210.1|PACid:18220173 | - | - |
| POPTR_0475s00210.2|PACid:18220174 | - | - |
| POPTR_0014s10520.1|PACid:18222319 | - | - |
| POPTR_0009s06540.1|PACid:18227630 | - | - |
| POPTR_0010s24370.1|PACid:18241829 | - | - |
| POPTR_0002s18480.1|PACid:18244367 | - | - |
| POPTR_0002s13590.1|PACid:18244756 | transport, localization | binding, transporter activity |
| POPTR_0008s02300.1|PACid:18249071 |  |  |
|  |  |  |
| **oeu-miR169s** |  |  |
| POPTR_0005s09380.1|PACid:18208583 | - | - |
| POPTR_0013s13440.1|PACid:18220664 | - | - |
| POPTR_0013s13440.2|PACid:18220665 | - | - |
|  |  |  |
| **oeu-miR169v** |  |  |
| POPTR_0018s00900.1|PACid:18214285 | - | - |
| POPTR_0002s18480.1|PACid:18244367 | - | - |
| POPTR_0002s13590.1|PACid:18244756 | transport, localization | binding, transporter activity |
|  |  |  |
| **oeu-miR169w** |  |  |
| POPTR_0018s00900.1|PACid:18214285 | - | - |
| POPTR_0002s18480.1|PACid:18244367 | - | - |
| POPTR_0002s13590.1|PACid:18244756 | transport, localization | binding, transporter activity |
|  |  |  |
| **oeu_miR171a** |  |  |
| POPTR_0003s11020.1|PACid:18217540 | organismal development | binding |
| POPTR_0014s05940.1|PACid:18224085 | organismal development | binding |
| POPTR_0014s05910.1|PACid:18224320 | organismal development | binding |
| POPTR_0001s00480.1|PACid:18235073 | organismal development | binding |
| POPTR_0002s14500.1|PACid:18244306 | organismal development | binding |
| POPTR_0002s14550.1|PACid:18244914 | organismal development | binding |
| POPTR_0008s12930.1|PACid:18249102 | metabolic process | oxidoreductase activity, catalytic activity |
|  |  |  |
| **oeu_miR171b** |  |  |
| POPTR_0003s11020.1|PACid:18217540 | organismal development | binding |
| POPTR_0014s05940.1|PACid:18224085 | organismal development | binding |
| POPTR_0014s05910.1|PACid:18224320 | organismal development | binding |
| POPTR_0001s00480.1|PACid:18235073 | organismal development | binding |
| POPTR_0002s14500.1|PACid:18244306 | organismal development | binding |
| POPTR_0002s14550.1|PACid:18244914 | organismal development | binding |
| POPTR_0008s12930.1|PACid:18249102 | metabolic process | oxidoreductase activity, catalytic activity |
|  |  |  |
| **oeu_miR171c** |  |  |
| POPTR_0005s12730.1|PACid:18208347 | - | - |
| POPTR_0003s11020.1|PACid:18217540 | organismal development | binding |
| POPTR_0014s05940.1|PACid:18224085 | organismal development | binding |
| POPTR_0014s05910.1|PACid:18224320 | organismal development | binding |
| POPTR_0011s02780.1|PACid:18230883 | - | - |
| POPTR_0001s00480.1|PACid:18235073 | organismal development | binding |
| POPTR_0007s12480.1|PACid:18243214 | - | - |
| POPTR_0002s14500.1|PACid:18244306 | organismal development | binding |
| POPTR_0002s14550.1|PACid:18244914 | organismal development | binding |
|  |  |  |
| **oeu_miR171d** |  |  |
| POPTR_0005s12730.1|PACid:18208347 | - | - |
| POPTR_0003s11020.1|PACid:18217540 | organismal development | binding |
| POPTR_0014s05940.1|PACid:18224085 | organismal development | binding |
| POPTR_0014s05910.1|PACid:18224320 | organismal development | binding |
| POPTR_0011s02780.1|PACid:18230883 | - | - |
| POPTR_0001s00480.1|PACid:18235073 | organismal development | binding |
| POPTR_0007s12480.1|PACid:18243214 | - | - |
| POPTR_0002s14500.1|PACid:18244306 | organismal development | binding |
| POPTR_0002s14550.1|PACid:18244914 | organismal development | binding |
|  |  |  |
| **oeu_miR171e** |  |  |
| POPTR_0005s12730.1|PACid:18208347 | - | - |
| POPTR_0003s11020.1|PACid:18217540 | organismal development | binding |
| POPTR_0014s05940.1|PACid:18224085 | organismal development | binding |
| POPTR_0014s05910.1|PACid:18224320 | organismal development | binding |
| POPTR_0001s00480.1|PACid:18235073 | organismal development | binding |
| POPTR_0007s12480.1|PACid:18243214 | - | - |
| POPTR_0002s14500.1|PACid:18244306 | organismal development | binding |
| POPTR_0002s14550.1|PACid:18244914 | organismal development | binding |
|  |  |  |
| **oeu_miR171f** |  |  |
| POPTR_0005s12730.1|PACid:18208347 | - | - |
| POPTR_0003s11020.1|PACid:18217540 | organismal development | binding |
| POPTR_0014s05940.1|PACid:18224085 | organismal development | binding |
| POPTR_0014s05910.1|PACid:18224320 | organismal development | binding |
| POPTR_0001s00480.1|PACid:18235073 | organismal development | binding |
| POPTR_0007s12480.1|PACid:18243214 | - | - |
| POPTR_0002s14500.1|PACid:18244306 | organismal development | binding |
| POPTR_0002s14550.1|PACid:18244914 | organismal development | binding |
|  |  |  |
| **oeu_miR171g** |  |  |
| POPTR_0005s12730.1|PACid:18208347 | - | - |
| POPTR_0003s11020.1|PACid:18217540 | organismal development | binding |
| POPTR_0014s05940.1|PACid:18224085 | organismal development | binding |
| POPTR_0014s05910.1|PACid:18224320 | organismal development | binding |
| POPTR_0001s00480.1|PACid:18235073 | organismal development | binding |
| POPTR_0007s12480.1|PACid:18243214 | - | - |
| POPTR_0002s14500.1|PACid:18244306 | organismal development | binding |
| POPTR_0002s14550.1|PACid:18244914 | organismal development | binding |
|  |  |  |
| **oeu_miR171h** |  |  |
| POPTR_0005s12730.1|PACid:18208347 | - | - |
| POPTR_0003s11020.1|PACid:18217540 | organismal development | binding |
| POPTR_0014s05940.1|PACid:18224085 | organismal development | binding |
| POPTR_0014s05910.1|PACid:18224320 | organismal development | binding |
| POPTR_0001s00480.1|PACid:18235073 | organismal development | binding |
| POPTR_0007s12480.1|PACid:18243214 | - | - |
| POPTR_0002s14500.1|PACid:18244306 | organismal development | binding |
| POPTR_0002s14550.1|PACid:18244914 | organismal development | binding |
|  |  |  |
| **oeu_miR171i** |  |  |
| POPTR_0005s12730.1|PACid:18208347 | - | - |
| POPTR_0003s11020.1|PACid:18217540 | organismal development | binding |
| POPTR_0014s05940.1|PACid:18224085 | organismal development | binding |
| POPTR_0014s05910.1|PACid:18224320 | organismal development | binding |
| POPTR_0001s00480.1|PACid:18235073 | organismal development | binding |
| POPTR_0007s12480.1|PACid:18243214 | - | - |
| POPTR_0002s14500.1|PACid:18244306 | organismal development | binding |
| POPTR_0002s14550.1|PACid:18244914 | organismal development | binding |
|  |  |  |
| **oeu_miR171l** |  |  |
| POPTR_0003s00850.1|PACid:18216075 | - | - |
| POPTR_0013s02090.1|PACid:18221203 | - | binding |
| POPTR_0015s03690.1|PACid:18233689 | metabolic process, gene expression | - |
| POPTR_0001s28200.1|PACid:18239264 | unannotated | binding |
| POPTR_0001s28200.2|PACid:18239265 | unannotated | binding |
| POPTR_0007s07100.1|PACid:18244015 | metabolic process | binding |
| POPTR_0002s03010.1|PACid:18246505 | metabolic process | transporter activity |
| POPTR_0008s12780.1|PACid:18248713 | metabolic process | transporter activity |
|  |  |  |
| **oeu_miR171m** |  |  |
| POPTR_0003s00850.1|PACid:18216075 | - | - |
| POPTR_0013s02090.1|PACid:18221203 | - | binding |
| POPTR_0015s03690.1|PACid:18233689 | metabolic process, gene expression | - |
| POPTR_0001s28200.1|PACid:18239264 | unannotated | binding |
| POPTR_0001s28200.2|PACid:18239265 | unannotated | binding |
| POPTR_0007s07100.1|PACid:18244015 | metabolic process | binding |
| POPTR_0002s03010.1|PACid:18246505 | metabolic process | transporter activity |
| POPTR_0008s12780.1|PACid:18248713 | metabolic process | transporter activity |
|  |  |  |
| **oeu_miR171n** |  |  |
| POPTR_0003s00850.1|PACid:18216075 | - | - |
| POPTR_0013s02090.1|PACid:18221203 | - | binding |
| POPTR_0015s03690.1|PACid:18233689 | metabolic process, gene expression | - |
| POPTR_0001s28200.1|PACid:18239264 | unannotated | binding |
| POPTR_0001s28200.2|PACid:18239265 | unannotated | binding |
| POPTR_0007s07100.1|PACid:18244015 | metabolic process | binding |
| POPTR_0002s03010.1|PACid:18246505 | metabolic process | transporter activity |
| POPTR_0008s12780.1|PACid:18248713 | metabolic process | transporter activity |
| POPTR_0001s39360.1|PACid:18239100 | biological regulation, metabolic process | oxidoreductase activity, binding, phosphotransferase activity, protein kinase activity |
| POPTR_0010s07550.1|PACid:18240913 |  |  |
| POPTR_0010s22320.1|PACid:18241770 | transcription, metabolic process | binding |
| POPTR_0007s10780.1|PACid:18243618 | organismal development, reproductive developmental process | binding, oxidoreductase activity |
| POPTR_0007s05600.1|PACid:18243665 |  |  |
| POPTR_0008s04490.1|PACid:18249709 | transcription, metabolic process | binding |
| POPTR_0016s08530.1|PACid:18250437 | organismal development, reproductive developmental process | binding |
|  |  |  |
| **oeu_miR172a** |  |  |
| POPTR_0005s18400.1|PACid:18208379 | organismal development, reproductive developmental process | binding, oxidoreductase activity |
| POPTR_0006s13460.1|PACid:18211020 | organismal development | binding |
| POPTR_0014s18780.1|PACid:18222675 | metabolic process | binding |
| POPTR_0009s01450.1|PACid:18228789 | - | - |
| POPTR_0012s12900.1|PACid:18229436 | metabolic process | binding, transferase activity, catalytic activity |
| POPTR_0015s11150.1|PACid:18233095 | - | - |
| POPTR_0001s39320.1|PACid:18235362 | metabolic process | binding, phosphotransferase activity, protein kinase activity |
| POPTR_0001s43900.1|PACid:18237219 | metabolic process | binding, protein kinase activity |
| POPTR_0001s03650.1|PACid:18238002 | biological regulation, homeostatic process, cellular process | binding, phosphotransferase activity, protein kinase activity |
| POPTR_0001s42330.1|PACid:18238175 | metabolic process | binding, phosphotransferase activity, protein kinase activity |
| POPTR_0001s42330.2|PACid:18238176 | metabolic process | binding, phosphotransferase activity, protein kinase activity |
| POPTR_0001s42340.1|PACid:18238610 | metabolic process | binding, phosphotransferase activity, protein kinase activity |
| POPTR_0001s39360.1|PACid:18239100 | biological regulation, homeostatic process, cellular process | binding, oxidoreductase activity, phosphotransferase activity,protein kinase activity |
| POPTR_0010s24450.1|PACid:18241005 | - | - |
| POPTR_0010s22320.1|PACid:18241770 | transcription, metabolic process, RNA biosynthetic process | binding |
| POPTR_0007s10780.1|PACid:18243618 | organismal development, reproductive developmental process | binding, oxidoreductase activity |
| POPTR_0008s04490.1|PACid:18249709 | transcription, metabolic process, RNA biosynthetic process | binding |
| POPTR_0016s07380.1|PACid:18249910 | - | - |
| POPTR_0016s08530.1|PACid:18250437 | organismal development, reproductive developmental process | binding |
|  |  |  |
| **oeu_miR172b** |  |  |
| POPTR_0005s18400.1|PACid:18208379 | organismal development, reproductive developmental process | binding, oxidoreductase activity |
| POPTR_0006s13460.1|PACid:18211020 | organismal development | binding |
| POPTR_0014s18780.1|PACid:18222675 | metabolic process | binding |
| POPTR_0009s01450.1|PACid:18228789 | - | - |
| POPTR_0012s12900.1|PACid:18229436 | metabolic process | binding, transferase activity, catalytic activity |
| POPTR_0015s11150.1|PACid:18233095 | - | - |
| POPTR_0001s39320.1|PACid:18235362 | metabolic process | binding, phosphotransferase activity, protein kinase activity |
| POPTR_0001s43900.1|PACid:18237219 | metabolic process | binding, protein kinase activity |
| POPTR_0001s03650.1|PACid:18238002 | biological regulation, homeostatic process, cellular process | transporter activity |
| POPTR_0001s42330.1|PACid:18238175 | metabolic process | binding, phosphotransferase activity, protein kinase activity |
| POPTR_0001s42330.2|PACid:18238176 | metabolic process | binding, phosphotransferase activity, protein kinase activity |
| POPTR_0001s42340.1|PACid:18238610 | metabolic process | binding, phosphotransferase activity, protein kinase activity |
| POPTR_0001s39360.1|PACid:18239100 | biological regulation, homeostatic process, cellular process | binding, oxidoreductase activity, phosphotransferase activity,protein kinase activity |
| POPTR_0010s24450.1|PACid:18241005 | - | - |
| POPTR_0010s22320.1|PACid:18241770 | transcription, metabolic process, RNA biosynthetic process | binding |
| POPTR_0007s10780.1|PACid:18243618 | organismal development, reproductive developmental process | binding, oxidoreductase activity |
| POPTR_0008s04490.1|PACid:18249709 | transcription, metabolic process, RNA biosynthetic process | binding |
| POPTR_0016s07380.1|PACid:18249910 | - | - |
| POPTR_0016s08530.1|PACid:18250437 | organismal development, reproductive developmental process | binding |
|  |  |  |
| **oeu_miR172c** |  |  |
| POPTR_0005s18400.1|PACid:18208379 | organismal development, reproductive developmental process | binding, oxidoreductase activity |
| POPTR_0006s13460.1|PACid:18211020 | organismal development | binding |
| POPTR_0014s18780.1|PACid:18222675 | metabolic process | binding |
| POPTR_0009s01450.1|PACid:18228789 | - | - |
| POPTR_0012s12900.1|PACid:18229436 | metabolic process | binding, transferase activity, catalytic activity |
| POPTR_0015s11150.1|PACid:18233095 | - | - |
| POPTR_0001s39320.1|PACid:18235362 | metabolic process | binding, phosphotransferase activity, protein kinase activity |
| POPTR_0001s43900.1|PACid:18237219 | metabolic process | binding, protein kinase activity |
| POPTR_0001s03650.1|PACid:18238002 | biological regulation, homeostatic process, cellular process | transporter activity |
| POPTR_0001s42330.1|PACid:18238175 | metabolic process | binding, phosphotransferase activity, protein kinase activity |
| POPTR_0001s42330.2|PACid:18238176 | metabolic process | binding, phosphotransferase activity, protein kinase activity |
| POPTR_0001s42340.1|PACid:18238610 | metabolic process | binding, phosphotransferase activity, protein kinase activity |
| POPTR_0001s39360.1|PACid:18239100 | biological regulation, homeostatic process, cellular process | binding, oxidoreductase activity, phosphotransferase activity,protein kinase activity |
| POPTR_0010s24450.1|PACid:18241005 | - | - |
| POPTR_0010s22320.1|PACid:18241770 | transcription, metabolic process, RNA biosynthetic process | binding |
| POPTR_0007s10780.1|PACid:18243618 | organismal development, reproductive developmental process | binding, oxidoreductase activity |
| POPTR_0008s04490.1|PACid:18249709 | transcription, metabolic process, RNA biosynthetic process | binding |
| POPTR_0016s07380.1|PACid:18249910 | - | - |
| POPTR_0016s08530.1|PACid:18250437 | organismal development, reproductive developmental process | binding |
|  |  |  |
| **oeu_miR172d** |  |  |
| POPTR_0005s18400.1|PACid:18208379 | organismal development, reproductive developmental process | binding, oxidoreductase activity |
| POPTR_0006s13460.1|PACid:18211020 | organismal development | binding |
| POPTR_0004s05190.1|PACid:18225557 |  |  |
| POPTR_0009s01450.1|PACid:18228789 | - | - |
| POPTR_0001s03470.1|PACid:18236316 | metabolic process | binding |
| POPTR_0001s45870.1|PACid:18236492 | - | - |
| POPTR_0001s03650.1|PACid:18238002 | biological regulation | transporter activity |
| POPTR_0010s22320.1|PACid:18241770 | transcription, metabolic process, RNA biosynthetic process | binding |
| POPTR_0007s10780.1|PACid:18243618 | organismal development, reproductive developmental process | binding, oxidoreductase activity |
| POPTR_0008s04490.1|PACid:18249709 | transcription, metabolic process, RNA biosynthetic process | binding |
| POPTR_0016s07380.1|PACid:18249910 | - | - |
| POPTR_0016s08530.1|PACid:18250437 | organismal development, reproductive developmental process | binding |
|  |  |  |
| **oeu_miR172e** |  |  |
| POPTR_0005s18400.1|PACid:18208379 | organismal development, reproductive developmental process | binding, oxidoreductase activity |
| POPTR_0006s13460.1|PACid:18211020 | organismal development | binding |
| POPTR_0004s05190.1|PACid:18225557 |  |  |
| POPTR_0009s01450.1|PACid:18228789 | - | - |
| POPTR_0001s03470.1|PACid:18236316 | metabolic process | binding |
| POPTR_0001s45870.1|PACid:18236492 | - | - |
| POPTR_0001s03650.1|PACid:18238002 | biological regulation | transporter activity |
| POPTR_0010s22320.1|PACid:18241770 | transcription, metabolic process, RNA biosynthetic process | binding |
| POPTR_0007s10780.1|PACid:18243618 | organismal development, reproductive developmental process | binding, oxidoreductase activity |
| POPTR_0008s04490.1|PACid:18249709 | transcription, metabolic process, RNA biosynthetic process | binding |
| POPTR_0016s07380.1|PACid:18249910 | - | - |
| POPTR_0016s08530.1|PACid:18250437 | organismal development, reproductive developmental process | binding |
|  |  |  |
| **oeu_miR172f** |  |  |
| POPTR_0005s18400.1|PACid:18208379 | organismal development, reproductive developmental process | binding, oxidoreductase activity |
| POPTR_0006s13460.1|PACid:18211020 | organismal development | binding |
| POPTR_0014s18780.1|PACid:18222675 | metabolic process | binding |
| POPTR_0009s01450.1|PACid:18228789 | - | - |
| POPTR_0012s12900.1|PACid:18229436 | metabolic process | binding, transferase activity, catalytic activity |
| POPTR_0015s11150.1|PACid:18233095 | - | - |
| POPTR_0001s39320.1|PACid:18235362 | metabolic process | binding, phosphotransferase activity, protein kinase activity |
| POPTR_0001s43900.1|PACid:18237219 | metabolic process | binding, protein kinase activity |
| POPTR_0001s03650.1|PACid:18238002 | biological regulation, homeostatic process, cellular process | transporter activity |
| POPTR_0001s42330.1|PACid:18238175 | metabolic process | binding, phosphotransferase activity, protein kinase activity |
| POPTR_0001s42330.2|PACid:18238176 | metabolic process | binding, phosphotransferase activity, protein kinase activity |
| POPTR_0001s42340.1|PACid:18238610 | metabolic process | binding, phosphotransferase activity, protein kinase activity |
| POPTR_0001s39360.1|PACid:18239100 | biological regulation, homeostatic process, cellular process | binding, oxidoreductase activity, phosphotransferase activity,protein kinase activity |
| POPTR_0010s24450.1|PACid:18241005 | - | - |
| POPTR_0010s22320.1|PACid:18241770 | transcription, metabolic process, RNA biosynthetic process | binding |
| POPTR_0007s10780.1|PACid:18243618 | organismal development, reproductive developmental process | binding, oxidoreductase activity |
| POPTR_0008s04490.1|PACid:18249709 | transcription, metabolic process, RNA biosynthetic process | binding |
| POPTR_0016s07380.1|PACid:18249910 | - | - |
| POPTR_0016s08530.1|PACid:18250437 | organismal development, reproductive developmental process | binding |
|  |  |  |
| **oeu_miR172g** |  |  |
| POPTR_0005s18400.1|PACid:18208379 | organismal development, reproductive developmental process | binding, oxidoreductase activity |
| POPTR_0006s13460.1|PACid:18211020 | organismal development | binding |
| POPTR_0004s05190.1|PACid:18225557 | transport, localization | signal transducer activity, transporter activity |
| POPTR_0009s01450.1|PACid:18228789 | - | - |
| POPTR_0011s13300.1|PACid:18230882 | - | - |
| POPTR_0011s13290.1|PACid:18231041 | - | - |
| POPTR_0015s11150.1|PACid:18233095 | - | - |
| POPTR_0001s42300.1|PACid:18234792 | - | - |
| POPTR_0001s39320.1|PACid:18235362 | metabolic process | binding, phosphotransferase activity, protein kinase activity |
| POPTR_0001s43900.1|PACid:18237219 | metabolic process | binding, protein kinase activity |
| POPTR_0001s42330.1|PACid:18238175 | metabolic process | binding, phosphotransferase activity, protein kinase activity |
| POPTR_0001s42330.2|PACid:18238176 | metabolic process | binding, phosphotransferase activity, protein kinase activity |
| POPTR_0001s42340.1|PACid:18238610 | metabolic process | binding, phosphotransferase activity, protein kinase activity |
| POPTR_0001s39360.1|PACid:18239100 | biological regulation, metabolic process | oxidoreductase activity, binding, phosphotransferase activity, protein kinase activity |
| POPTR_0010s07550.1|PACid:18240913 |  |  |
| POPTR_0010s22320.1|PACid:18241770 | transcription, metabolic process | binding |
| POPTR_0007s10780.1|PACid:18243618 | organismal development, reproductive developmental process | binding, oxidoreductase activity |
| POPTR_0007s05600.1|PACid:18243665 |  |  |
| POPTR_0008s04490.1|PACid:18249709 | transcription, metabolic process | binding |
| POPTR_0016s08530.1|PACid:18250437 | organismal development, reproductive developmental process | binding |
|  |  |  |
| **oeu_miR172h** |  |  |
| POPTR_0005s18400.1|PACid:18208379 | organismal development, reproductive developmental process | binding, oxidoreductase activity |
| POPTR_0006s13460.1|PACid:18211020 | organismal development | binding |
| POPTR_0004s05190.1|PACid:18225557 | transport, localization | signal transducer activity, transporter activity |
| POPTR_0009s01450.1|PACid:18228789 | - | - |
| POPTR_0011s13300.1|PACid:18230882 | - | - |
| POPTR_0011s13290.1|PACid:18231041 | - | - |
| POPTR_0015s11150.1|PACid:18233095 | - | - |
| POPTR_0001s42300.1|PACid:18234792 | - | - |
| POPTR_0001s39320.1|PACid:18235362 | metabolic process | binding, phosphotransferase activity, protein kinase activity |
| POPTR_0001s43900.1|PACid:18237219 | metabolic process | binding, protein kinase activity |
| POPTR_0001s42330.1|PACid:18238175 | metabolic process | binding, phosphotransferase activity, protein kinase activity |
| POPTR_0001s42330.2|PACid:18238176 | metabolic process | binding, phosphotransferase activity, protein kinase activity |
| POPTR_0001s42340.1|PACid:18238610 | metabolic process | binding, phosphotransferase activity, protein kinase activity |
| POPTR_0001s39360.1|PACid:18239100 | biological regulation, metabolic process | oxidoreductase activity, binding, phosphotransferase activity, protein kinase activity |
| POPTR_0010s07550.1|PACid:18240913 |  |  |
| POPTR_0010s22320.1|PACid:18241770 | transcription, metabolic process | binding |
| POPTR_0007s10780.1|PACid:18243618 | organismal development, reproductive developmental process | binding, oxidoreductase activity |
| POPTR_0007s05600.1|PACid:18243665 |  |  |
| POPTR_0008s04490.1|PACid:18249709 | transcription, metabolic process | binding |
| POPTR_0016s08530.1|PACid:18250437 | organismal development, reproductive developmental process | binding |
|  |  |  |
| **oeu_miR172i** |  |  |
| POPTR_0005s18400.1|PACid:18208379 | organismal development, reproductive developmental process | binding, oxidoreductase activity |
| POPTR_0006s13460.1|PACid:18211020 | organismal development | binding |
| POPTR_0006s19000.1|PACid:18213183 | metabolic process | carbohydrate phosphatase activity,transporter activity |
| POPTR_0013s02500.1|PACid:18221731 | metabolic process | hydrolase activity |
| POPTR_0004s05190.1|PACid:18225557 | transport, localization | signal transducer activity, transmembrane transporter activity |
| POPTR_0009s01450.1|PACid:18228789 | - | - |
| POPTR_0010s14710.1|PACid:18239858 | metabolic process | hydrolase activity |
| POPTR_0010s24450.1|PACid:18241005 | - | - |
| POPTR_0010s22320.1|PACid:18241770 | transcription, metabolic process | binding |
| POPTR_0007s10780.1|PACid:18243618 | organismal development, reproductive developmental process | binding, oxidoreductase activity |
| POPTR_0008s04490.1|PACid:18249709 | transcription, metabolic process | binding |
| POPTR_0016s07380.1|PACid:18249910 | - | - |
| POPTR_0016s08530.1|PACid:18250437 | organismal development, reproductive developmental process | binding |
|  |  |  |
| **oeu_miR319a** |  |  |
| POPTR_0003s18900.1|PACid:18216192 | organismal development, reproductive developmental process, transcription, hormone-mediated signalling | binding |
| POPTR_0003s18900.2|PACid:18216193 | organismal development, reproductive developmental process, transcription, hormone-mediated signalling | binding |
| POPTR_0003s10410.1|PACid:18217916 | - | - |
| POPTR_0013s14200.1|PACid:18220958 | - | - |
| POPTR_0004s06440.1|PACid:18225754 | - | - |
| POPTR_0009s02380.1|PACid:18227868 | - | binding |
| POPTR_0012s11080.1|PACid:18230002 | - | - |
| POPTR_0011s02350.1|PACid:18231968 | - | - |
| POPTR_0001s07330.1|PACid:18234458 | organismal development, reproductive developmental process, transcription, hormone-mediated signalling | binding |
| POPTR_0001s07330.2|PACid:18234459 | organismal development, reproductive developmental process, transcription, hormone-mediated signalling | binding |
| POPTR_0001s23170.1|PACid:18238390 | - | binding |
| POPTR_0001s38460.1|PACid:18238723 | - | - |
|  |  |  |
| **oeu_miR319b** |  |  |
| POPTR_0003s18900.1|PACid:18216192 | organismal development, reproductive developmental process, transcription, hormone-mediated signalling | binding |
| POPTR_0003s18900.2|PACid:18216193 | organismal development, reproductive developmental process, transcription, hormone-mediated signalling | binding |
| POPTR_0003s10410.1|PACid:18217916 | - | - |
| POPTR_0013s14200.1|PACid:18220958 | - | - |
| POPTR_0004s06440.1|PACid:18225754 | - | - |
| POPTR_0009s02380.1|PACid:18227868 | - | binding |
| POPTR_0012s11080.1|PACid:18230002 | - | - |
| POPTR_0011s02350.1|PACid:18231968 | - | - |
| POPTR_0001s07330.1|PACid:18234458 | organismal development, reproductive developmental process, transcription, hormone-mediated signalling | binding |
| POPTR_0001s07330.2|PACid:18234459 | organismal development, reproductive developmental process, transcription, hormone-mediated signalling | binding |
| POPTR_0001s23170.1|PACid:18238390 | - | binding |
| POPTR_0001s38460.1|PACid:18238723 | - | - |
|  |  |  |
| **oeu_miR319c** |  |  |
| POPTR_0003s18900.1|PACid:18216192 | organismal development, reproductive developmental process, transcription, hormone-mediated signalling | binding |
| POPTR_0003s18900.2|PACid:18216193 | organismal development, reproductive developmental process, transcription, hormone-mediated signalling | binding |
| POPTR_0003s10410.1|PACid:18217916 | - | - |
| POPTR_0013s14200.1|PACid:18220958 | - | - |
| POPTR_0004s06440.1|PACid:18225754 | - | - |
| POPTR_0009s02380.1|PACid:18227868 | - | binding |
| POPTR_0012s11080.1|PACid:18230002 | - | - |
| POPTR_0011s02350.1|PACid:18231968 | - | - |
| POPTR_0001s07330.1|PACid:18234458 | organismal development, reproductive developmental process, transcription, hormone-mediated signalling | binding |
| POPTR_0001s07330.2|PACid:18234459 | organismal development, reproductive developmental process, transcription, hormone-mediated signalling | binding |
| POPTR_0001s23170.1|PACid:18238390 | - | binding |
| POPTR_0001s38460.1|PACid:18238723 | - | - |
|  |  |  |
| **oeu_miR319d** |  |  |
| POPTR_0003s18900.1|PACid:18216192 | organismal development, reproductive developmental process, transcription, hormone-mediated signalling | binding |
| POPTR_0003s18900.2|PACid:18216193 | organismal development, reproductive developmental process, transcription, hormone-mediated signalling | binding |
| POPTR_0003s10410.1|PACid:18217916 | - | - |
| POPTR_0013s14200.1|PACid:18220958 | - | - |
| POPTR_0004s06440.1|PACid:18225754 | - | - |
| POPTR_0009s02380.1|PACid:18227868 | - | binding |
| POPTR_0012s11080.1|PACid:18230002 | - | - |
| POPTR_0011s02350.1|PACid:18231968 | - | - |
| POPTR_0001s07330.1|PACid:18234458 | organismal development, reproductive developmental process, transcription, hormone-mediated signalling | binding |
| POPTR_0001s07330.2|PACid:18234459 | organismal development, reproductive developmental process, transcription, hormone-mediated signalling | binding |
| POPTR_0001s23170.1|PACid:18238390 | - | binding |
| POPTR_0001s38460.1|PACid:18238723 | - | - |
|  |  |  |
| **oeu_miR319e** |  |  |
| POPTR_0003s18900.1|PACid:18216192 | organismal development, reproductive developmental process, transcription | binding |
| POPTR_0003s18900.2|PACid:18216193 | organismal development, reproductive developmental process, transcription | binding |
| POPTR_0019s12110.1|PACid:18218990 | - | - |
| POPTR_0013s12370.1|PACid:18220855 | - | - |
| POPTR_0014s01760.1|PACid:18223665 |  | binding |
| POPTR_0004s16670.1|PACid:18224931 | - | - |
| POPTR_0004s06440.1|PACid:18225754 | - | - |
| POPTR_0009s02380.1|PACid:18227868 |  | binding |
| POPTR_0012s11080.1|PACid:18230002 | - | - |
| POPTR_0011s09890.1|PACid:18230603 | - | - |
| POPTR_0011s02350.1|PACid:18231968 | - | - |
| POPTR_0011s12790.1|PACid:18232088 | - | - |
| POPTR_0001s07330.1|PACid:18234458 | organismal development, reproductive developmental process, transcription, hormone-mediated signalling | binding |
| POPTR_0001s07330.2|PACid:18234459 | organismal development, reproductive developmental process, transcription, hormone-mediated signalling | binding |
| POPTR_0001s41950.1|PACid:18236778 | - | - |
| POPTR_0001s23170.1|PACid:18238390 | - | binding |
| POPTR_0001s38460.1|PACid:18238723 | - | - |
| POPTR_0001s38480.1|PACid:18239062 | - | - |
| POPTR_0007s01150.1|PACid:18243762 | - | - |
| POPTR_0002s12010.1|PACid:18244970 |  | binding |
|  |  |  |
| **oeu_miR319f** |  |  |
| POPTR_0003s18900.1|PACid:18216192 | organismal development, reproductive developmental process, transcription, hormone-mediated signalling | binding |
| POPTR_0003s18900.2|PACid:18216193 | organismal development, reproductive developmental process, transcription, hormone-mediated signalling | binding |
| POPTR_0019s12110.1|PACid:18218990 | - | - |
| POPTR_0013s12370.1|PACid:18220855 | - | - |
| POPTR_0014s01760.1|PACid:18223665 |  | binding |
| POPTR_0004s16670.1|PACid:18224931 | - | - |
| POPTR_0004s06440.1|PACid:18225754 | - | - |
| POPTR_0009s02380.1|PACid:18227868 |  | binding |
| POPTR_0012s11080.1|PACid:18230002 | - | - |
| POPTR_0011s09890.1|PACid:18230603 | - | - |
| POPTR_0011s02350.1|PACid:18231968 | - | - |
| POPTR_0011s12790.1|PACid:18232088 | - | - |
| POPTR_0001s07330.1|PACid:18234458 | organismal development, reproductive developmental process, transcription, hormone-mediated signalling | binding |
| POPTR_0001s07330.2|PACid:18234459 | organismal development, reproductive developmental process, transcription, hormone-mediated signalling | binding |
| POPTR_0001s41950.1|PACid:18236778 | - | - |
| POPTR_0001s23170.1|PACid:18238390 | - | binding |
| POPTR_0001s38460.1|PACid:18238723 | - | - |
| POPTR_0001s38480.1|PACid:18239062 | - | - |
| POPTR_0007s01150.1|PACid:18243762 | - | - |
| POPTR_0002s12010.1|PACid:18244970 |  | binding |
|  |  |  |
| **oeu_miR319g** |  |  |
| POPTR_0003s18900.1|PACid:18216192 | organismal development, reproductive developmental process, transcription, hormone-mediated signalling | binding |
| POPTR_0003s18900.2|PACid:18216193 | organismal development, reproductive developmental process, transcription, hormone-mediated signalling | binding |
| POPTR_0019s12110.1|PACid:18218990 | - | - |
| POPTR_0013s12370.1|PACid:18220855 | - | - |
| POPTR_0014s01760.1|PACid:18223665 |  | binding |
| POPTR_0004s16670.1|PACid:18224931 | - | - |
| POPTR_0004s06440.1|PACid:18225754 | - | - |
| POPTR_0009s02380.1|PACid:18227868 |  | binding |
| POPTR_0012s11080.1|PACid:18230002 | - | - |
| POPTR_0011s09890.1|PACid:18230603 | - | - |
| POPTR_0011s02350.1|PACid:18231968 | - | - |
| POPTR_0011s12790.1|PACid:18232088 | - | - |
| POPTR_0001s07330.1|PACid:18234458 | organismal development, reproductive developmental process, transcription, hormone-mediated signalling | binding |
| POPTR_0001s07330.2|PACid:18234459 | organismal development, reproductive developmental process, transcription, hormone-mediated signalling | binding |
| POPTR_0001s41950.1|PACid:18236778 | - | - |
| POPTR_0001s23170.1|PACid:18238390 | - | binding |
| POPTR_0001s38460.1|PACid:18238723 | - | - |
| POPTR_0001s38480.1|PACid:18239062 | - | - |
| POPTR_0007s01150.1|PACid:18243762 | - | - |
| POPTR_0002s12010.1|PACid:18244970 |  | binding |
|  |  |  |
| **oeu_miR319h** |  |  |
| POPTR_0003s18900.1|PACid:18216192 | organismal development, reproductive developmental process, transcription, hormone-mediated signalling | binding |
| POPTR_0003s18900.2|PACid:18216193 | organismal development, reproductive developmental process, transcription, hormone-mediated signalling | binding |
| POPTR_0019s12110.1|PACid:18218990 | - | - |
| POPTR_0013s12370.1|PACid:18220855 | - | - |
| POPTR_0014s01760.1|PACid:18223665 |  | binding |
| POPTR_0004s16670.1|PACid:18224931 | - | - |
| POPTR_0004s06440.1|PACid:18225754 | - | - |
| POPTR_0009s02380.1|PACid:18227868 |  | binding |
| POPTR_0012s11080.1|PACid:18230002 | - | - |
| POPTR_0011s09890.1|PACid:18230603 | - | - |
| POPTR_0011s02350.1|PACid:18231968 | - | - |
| POPTR_0011s12790.1|PACid:18232088 | - | - |
| POPTR_0001s07330.1|PACid:18234458 | organismal development, reproductive developmental process, transcription, hormone-mediated signalling | binding |
| POPTR_0001s07330.2|PACid:18234459 | organismal development, reproductive developmental process, transcription, hormone-mediated signalling | binding |
| POPTR_0001s41950.1|PACid:18236778 | - | - |
| POPTR_0001s23170.1|PACid:18238390 | - | binding |
| POPTR_0001s38460.1|PACid:18238723 | - | - |
| POPTR_0001s38480.1|PACid:18239062 | - | - |
| POPTR_0007s01150.1|PACid:18243762 | - | - |
| POPTR_0002s12010.1|PACid:18244970 |  | binding |
|  |  |  |
| **oeu_miR390a** |  |  |
| POPTR_0006s06000.1|PACid:18211727 | - | - |
| POPTR_0006s05020.1|PACid:18211815 | metabolic process | binding |
| POPTR_0006s19320.1|PACid:18211940 | metabolic process | binding, oxidoreductase activity |
| POPTR_0013s06050.1|PACid:18220914 | metabolic process | binding, oxidoreductase activity |
| POPTR_0009s14680.1|PACid:18228812 | biological regulation | binding |
| POPTR_0012s02990.1|PACid:18229249 | metabolic process | phosphotransferase activity, protein kinase activity |
| POPTR_0012s09070.1|PACid:18229721 | organismal development | oxidoreductase activity, kinase activity |
| POPTR_0001s12420.1|PACid:18236162 | organismal development | binding, oxidoreductase activity |
| POPTR_0001s12290.1|PACid:18237071 | organismal development | binding, oxidoreductase activity |
| POPTR_0010s08270.1|PACid:18239925 | inorganic anion transport | transporter activity |
| POPTR_0010s11150.1|PACid:18240166 | organismal development | binding, oxidoreductase activity |
| POPTR_0010s13130.1|PACid:18240509 | - | catalytic activity |
| POPTR_0010s08240.1|PACid:18241942 | inorganic anion transport | transporter activity |
| POPTR_0002s25300.1|PACid:18244397 | biological regulation, metabolic process | binding |
| POPTR_0002s25990.1|PACid:18245651 | metabolic process | binding, oxidoreductase activity |
| POPTR_0002s25980.1|PACid:18245740 | metabolic process | binding, oxidoreductase activity |
| POPTR_0002s26010.1|PACid:18246090 | metabolic process | binding, oxidoreductase activity |
| POPTR_0002s26010.2|PACid:18246091 | metabolic process | phosphotransferase activity, protein kinase activity |
| POPTR_0008s09590.1|PACid:18247915 | organismal development | phosphotransferase activity, protein kinase activity |
| POPTR_0008s12270.1|PACid:18248507 | - | catalytic activity, phosphatase activity |
| POPTR_0016s05570.1|PACid:18250426 | metabolic process | binding, oxidoreductase activity |
|  |  |  |
| **oeu_miR390b** |  |  |
| POPTR_0006s06000.1|PACid:18211727 | - | - |
| POPTR_0006s05020.1|PACid:18211815 | metabolic process | binding |
| POPTR_0006s19320.1|PACid:18211940 | metabolic process | binding, oxidoreductase activity |
| POPTR_0013s06050.1|PACid:18220914 | metabolic process | binding, oxidoreductase activity |
| POPTR_0009s14680.1|PACid:18228812 | biological regulation | binding |
| POPTR_0012s02990.1|PACid:18229249 | metabolic process | phosphotransferase activity, protein kinase activity |
| POPTR_0012s09070.1|PACid:18229721 | organismal development | oxidoreductase activity, kinase activity |
| POPTR_0001s12420.1|PACid:18236162 | organismal development | binding, oxidoreductase activity |
| POPTR_0001s12290.1|PACid:18237071 | organismal development | binding, oxidoreductase activity |
| POPTR_0010s08270.1|PACid:18239925 | inorganic anion transport | transporter activity |
| POPTR_0010s11150.1|PACid:18240166 | organismal development | binding, oxidoreductase activity |
| POPTR_0010s13130.1|PACid:18240509 | - | catalytic activity |
| POPTR_0010s08240.1|PACid:18241942 | inorganic anion transport | transporter activity |
| POPTR_0002s25300.1|PACid:18244397 | biological regulation, metabolic process | binding |
| POPTR_0002s25990.1|PACid:18245651 | metabolic process | binding, oxidoreductase activity |
| POPTR_0002s25980.1|PACid:18245740 | metabolic process | binding, oxidoreductase activity |
| POPTR_0002s26010.1|PACid:18246090 | metabolic process | binding, oxidoreductase activity |
| POPTR_0002s26010.2|PACid:18246091 | metabolic process | phosphotransferase activity, protein kinase activity |
| POPTR_0008s09590.1|PACid:18247915 | organismal development | phosphotransferase activity, protein kinase activity |
| POPTR_0008s12270.1|PACid:18248507 | - | catalytic activity, phosphatase activity |
| POPTR_0016s05570.1|PACid:18250426 | metabolic process | binding, oxidoreductase activity |
|  |  |  |
| **oeu_miR390c** |  |  |
| POPTR_0006s06000.1|PACid:18211727 | - | - |
| POPTR_0006s05020.1|PACid:18211815 | metabolic process | binding |
| POPTR_0006s19320.1|PACid:18211940 | metabolic process | binding, oxidoreductase activity |
| POPTR_0013s06050.1|PACid:18220914 | metabolic process | binding, oxidoreductase activity |
| POPTR_0009s14680.1|PACid:18228812 | biological regulation | binding |
| POPTR_0012s02990.1|PACid:18229249 | metabolic process | phosphotransferase activity, protein kinase activity |
| POPTR_0012s09070.1|PACid:18229721 | organismal development | oxidoreductase activity, kinase activity |
| POPTR_0001s12420.1|PACid:18236162 | organismal development | binding, oxidoreductase activity |
| POPTR_0001s12290.1|PACid:18237071 | organismal development | binding, oxidoreductase activity |
| POPTR_0010s08270.1|PACid:18239925 | inorganic anion transport | transporter activity |
| POPTR_0010s11150.1|PACid:18240166 | organismal development | binding, oxidoreductase activity |
| POPTR_0010s13130.1|PACid:18240509 | - | catalytic activity |
| POPTR_0010s08240.1|PACid:18241942 | inorganic anion transport | transporter activity |
| POPTR_0002s25300.1|PACid:18244397 | biological regulation, metabolic process | binding |
| POPTR_0002s25990.1|PACid:18245651 | metabolic process | binding, oxidoreductase activity |
| POPTR_0002s25980.1|PACid:18245740 | metabolic process | binding, oxidoreductase activity |
| POPTR_0002s26010.1|PACid:18246090 | metabolic process | binding, oxidoreductase activity |
| POPTR_0002s26010.2|PACid:18246091 | metabolic process | phosphotransferase activity, protein kinase activity |
| POPTR_0008s09590.1|PACid:18247915 | organismal development | phosphotransferase activity, protein kinase activity |
| POPTR_0008s12270.1|PACid:18248507 | - | catalytic activity, phosphatase activity |
| POPTR_0016s05570.1|PACid:18250426 | metabolic process | binding, oxidoreductase activity |
|  |  |  |
| **oeu_miR390d** |  |  |
| POPTR_0006s06000.1|PACid:18211727 | - | - |
| POPTR_0006s05020.1|PACid:18211815 | metabolic process | binding |
| POPTR_0006s19320.1|PACid:18211940 | metabolic process | binding, oxidoreductase activity |
| POPTR_0013s06050.1|PACid:18220914 | metabolic process | binding, oxidoreductase activity |
| POPTR_0009s14680.1|PACid:18228812 | biological regulation | binding |
| POPTR_0012s02990.1|PACid:18229249 | metabolic process | phosphotransferase activity, protein kinase activity |
| POPTR_0012s09070.1|PACid:18229721 | organismal development | oxidoreductase activity, kinase activity |
| POPTR_0001s12420.1|PACid:18236162 | organismal development | binding, oxidoreductase activity |
| POPTR_0001s12290.1|PACid:18237071 | organismal development | binding, oxidoreductase activity |
| POPTR_0010s08270.1|PACid:18239925 | inorganic anion transport | transporter activity |
| POPTR_0010s11150.1|PACid:18240166 | organismal development | binding, oxidoreductase activity |
| POPTR_0010s13130.1|PACid:18240509 | - | catalytic activity |
| POPTR_0010s08240.1|PACid:18241942 | inorganic anion transport | transporter activity |
| POPTR_0002s25300.1|PACid:18244397 | biological regulation, metabolic process | binding |
| POPTR_0002s25990.1|PACid:18245651 | metabolic process | binding, oxidoreductase activity |
| POPTR_0002s25980.1|PACid:18245740 | metabolic process | binding, oxidoreductase activity |
| POPTR_0002s26010.1|PACid:18246090 | metabolic process | binding, oxidoreductase activity |
| POPTR_0002s26010.2|PACid:18246091 | metabolic process | phosphotransferase activity, protein kinase activity |
| POPTR_0008s09590.1|PACid:18247915 | organismal development | phosphotransferase activity, protein kinase activity |
| POPTR_0008s12270.1|PACid:18248507 | - | catalytic activity, phosphatase activity |
| POPTR_0016s05570.1|PACid:18250426 | metabolic process | binding, oxidoreductase activity |
|  |  |  |
| **oeu_miR393a** |  |  |
| POPTR_0020s00280.1|PACid:18209105 | organismal development, reproductive developmental process, flower development | binding |
| POPTR_0017s08860.1|PACid:18210568 | organismal development, reproductive developmental process, flower development | binding |
| POPTR_0014s14650.1|PACid:18222417 | - | - |
| POPTR_0014s12930.1|PACid:18223294 | organismal development, reproductive developmental process, flower development | binding |
| POPTR_0015s08720.1|PACid:18233569 | response to stimulus, response to stress | binding |
| POPTR_0015s08710.1|PACid:18233889 | response to stimulus, response to stress | binding |
| POPTR_0001s42050.1|PACid:18234317 | metabolic process | binding, phosphotransferase activity, protein kinase activity |
| POPTR_0001s33030.1|PACid:18235568 | organismal development, reproductive developmental process, flower development | binding |
| POPTR_0001s39590.1|PACid:18238497 | transport, localization | binding |
| POPTR_0001s39590.2|PACid:18238498 | transport, localization | binding, phosphotransferase activity, protein kinase activity |
| POPTR_0010s21640.1|PACid:18240477 | - | - |
| POPTR_0002s23650.1|PACid:18245608 | - | - |
|  |  |  |
| **oeu_miR393b** |  |  |
| POPTR_0020s00280.1|PACid:18209105 | organismal development, reproductive developmental process, flower development | binding |
| POPTR_0017s08860.1|PACid:18210568 | organismal development, reproductive developmental process, flower development | binding |
| POPTR_0014s14650.1|PACid:18222417 | - | - |
| POPTR_0014s12930.1|PACid:18223294 | organismal development, reproductive developmental process, flower development | binding |
| POPTR_0015s08720.1|PACid:18233569 | response to stimulus, response to stress | binding |
| POPTR_0015s08710.1|PACid:18233889 | response to stimulus, response to stress | binding |
| POPTR_0001s42050.1|PACid:18234317 | metabolic process | binding, phosphotransferase activity, protein kinase activity |
| POPTR_0001s33030.1|PACid:18235568 | organismal development, reproductive developmental process, flower development | binding |
| POPTR_0001s39590.1|PACid:18238497 | transport, localization | binding |
| POPTR_0001s39590.2|PACid:18238498 | transport, localization | binding, phosphotransferase activity, protein kinase activity |
| POPTR_0010s21640.1|PACid:18240477 | - | - |
| POPTR_0002s23650.1|PACid:18245608 | - | - |
|  |  |  |
| **oeu_miR393c** |  |  |
| POPTR_0020s00280.1|PACid:18209105 | organismal development, reproductive developmental process, flower development | binding |
| POPTR_0017s08860.1|PACid:18210568 | organismal development, reproductive developmental process, flower development | binding |
| POPTR_0014s14650.1|PACid:18222417 | - | - |
| POPTR_0014s12930.1|PACid:18223294 | organismal development, reproductive developmental process, flower development | binding |
| POPTR_0015s08720.1|PACid:18233569 | response to stimulus, response to stress | binding |
| POPTR_0015s08710.1|PACid:18233889 | response to stimulus, response to stress | binding |
| POPTR_0001s42050.1|PACid:18234317 | metabolic process | binding, phosphotransferase activity, protein kinase activity |
| POPTR_0001s33030.1|PACid:18235568 | organismal development, reproductive developmental process, flower development | binding |
| POPTR_0001s39590.1|PACid:18238497 | transport, localization | binding |
| POPTR_0001s39590.2|PACid:18238498 | transport, localization | binding, phosphotransferase activity, protein kinase activity |
| POPTR_0010s21640.1|PACid:18240477 | - | - |
| POPTR_0002s23650.1|PACid:18245608 | - | - |
|  |  |  |
| **oeu_miR393d** |  |  |
| POPTR_0020s00280.1|PACid:18209105 | organismal development, reproductive developmental process, flower development | binding |
| POPTR_0017s08860.1|PACid:18210568 | organismal development, reproductive developmental process, flower development | binding |
| POPTR_0014s14650.1|PACid:18222417 | - | - |
| POPTR_0014s12930.1|PACid:18223294 | organismal development, reproductive developmental process, flower development | binding |
| POPTR_0015s08720.1|PACid:18233569 | response to stimulus, response to stress | binding |
| POPTR_0015s08710.1|PACid:18233889 | response to stimulus, response to stress | binding |
| POPTR_0001s42050.1|PACid:18234317 | metabolic process | binding, phosphotransferase activity, protein kinase activity |
| POPTR_0001s33030.1|PACid:18235568 | organismal development, reproductive developmental process, flower development | binding |
| POPTR_0001s39590.1|PACid:18238497 | transport, localization | binding |
| POPTR_0001s39590.2|PACid:18238498 | transport, localization | binding, transporter activity |
| POPTR_0010s21640.1|PACid:18240477 | - | - |
| POPTR_0002s23650.1|PACid:18245608 | - | - |
|  |  |  |
| **oeu_miR394a-5p** |  |  |
| POPTR_0005s06690.1|PACid:18208042 | - | - |
| POPTR_0018s10370.1|PACid:18214902 | - | - |
| POPTR_0003s16980.1|PACid:18217514 | - | - |
| POPTR_0003s16980.2|PACid:18217515 | - | - |
| POPTR_0003s16980.3|PACid:18217516 | - | - |
| POPTR_0003s05310.1|PACid:18218313 | - | - |
| POPTR_0014s12500.1|PACid:18223361 | - | - |
| POPTR_0014s10610.1|PACid:18223893 | - | - |
| POPTR_0012s08190.1|PACid:18229292 | - | - |
| POPTR_0001s13770.1|PACid:18234508 | - | - |
| POPTR_0010s13240.1|PACid:18240773 | - | - |
| POPTR_0002s20700.1|PACid:18244406 | - | - |
| POPTR_0002s07760.1|PACid:18244476 | - | - |
| POPTR_0002s18590.1|PACid:18245686 | - | - |
| POPTR_0002s18590.2|PACid:18245687 | - | - |
|  |  |  |
| **oeu_miR394b-5p** |  |  |
| POPTR_0005s06690.1|PACid:18208042 | - | - |
| POPTR_0018s10370.1|PACid:18214902 | - | - |
| POPTR_0003s16980.1|PACid:18217514 | - | - |
| POPTR_0003s16980.2|PACid:18217515 | - | - |
| POPTR_0003s16980.3|PACid:18217516 | - | - |
| POPTR_0003s05310.1|PACid:18218313 | - | - |
| POPTR_0014s12500.1|PACid:18223361 | - | - |
| POPTR_0014s10610.1|PACid:18223893 | - | - |
| POPTR_0012s08190.1|PACid:18229292 | - | - |
| POPTR_0001s13770.1|PACid:18234508 | - | - |
| POPTR_0010s13240.1|PACid:18240773 | - | - |
| POPTR_0002s20700.1|PACid:18244406 | - | - |
| POPTR_0002s07760.1|PACid:18244476 | - | - |
| POPTR_0002s18590.1|PACid:18245686 | - | - |
| POPTR_0002s18590.2|PACid:18245687 | - | - |
|  |  |  |
| **oeu_miR395b** |  |  |
| POPTR_0005s23530.1|PACid:18206966 | inorganic anion transport | transporter activity |
| POPTR_0009s03390.1|PACid:18228182 | - | - |
| POPTR_0009s03390.2|PACid:18228183 | - | - |
| POPTR_0009s01240.1|PACid:18228577 | metabolic process | binding |
| POPTR_0001s24390.1|PACid:18237160 | - | - |
| POPTR_0010s09140.1|PACid:18239985 | metabolic process, response to chemical stimulus | adenylyltransferase activity |
| POPTR_0008s13590.1|PACid:18249173 | metabolic process | catalytic activity |
| POPTR_0008s15880.1|PACid:18249729 | metabolic process, response to chemical stimulus | adenylyltransferase activity |
|  |  |  |
| **oeu_miR395c** |  |  |
| POPTR_0005s23530.1|PACid:18206966 | inorganic anion transport | transporter activity |
| POPTR_0009s03390.1|PACid:18228182 | - | - |
| POPTR_0009s03390.2|PACid:18228183 | - | - |
| POPTR_0009s01240.1|PACid:18228577 | primary metabolic process | binding |
| POPTR_0001s24390.1|PACid:18237160 | - | - |
| POPTR_0010s09140.1|PACid:18239985 | metabolic process, response to chemical stimulus | adenylyltransferase activity |
| POPTR_0008s13590.1|PACid:18249173 | metabolic process | catalytic activity |
| POPTR_0008s15880.1|PACid:18249729 | metabolic process, response to chemical stimulus | adenylyltransferase activity |
|  |  |  |
| **oeu_miR395d** |  |  |
| POPTR_0005s23530.1|PACid:18206966 | inorganic anion transport | transporter activity |
| POPTR_0009s03390.1|PACid:18228182 | - | - |
| POPTR_0009s03390.2|PACid:18228183 | - | - |
| POPTR_0009s01240.1|PACid:18228577 | metabolic process | binding |
| POPTR_0001s24390.1|PACid:18237160 | - | - |
| POPTR_0010s09140.1|PACid:18239985 | metabolic process, response to chemical stimulus | adenylyltransferase activity |
| POPTR_0008s13590.1|PACid:18249173 | metabolic process | catalytic activity |
| POPTR_0008s15880.1|PACid:18249729 | metabolic process, response to chemical stimulus | adenylyltransferase activity |
|  |  |  |
| **oeu_miR395e** |  |  |
| POPTR_0005s23530.1|PACid:18206966 | inorganic anion transport | transporter activity |
| POPTR_0009s03390.1|PACid:18228182 | - | - |
| POPTR_0009s03390.2|PACid:18228183 | - | - |
| POPTR_0009s01240.1|PACid:18228577 | metabolic process | binding |
| POPTR_0001s24390.1|PACid:18237160 | - | - |
| POPTR_0010s09140.1|PACid:18239985 | metabolic process, response to chemical stimulus | adenylyltransferase activity |
| POPTR_0008s13590.1|PACid:18249173 | metabolic process | catalytic activity |
| POPTR_0008s15880.1|PACid:18249729 | metabolic process, response to chemical stimulus | adenylyltransferase activity |
|  |  |  |
| **oeu_miR395f** |  |  |
| POPTR_0005s23530.1|PACid:18206966 | inorganic anion transport | transporter activity |
| POPTR_0009s03390.1|PACid:18228182 | - | - |
| POPTR_0009s03390.2|PACid:18228183 | - | - |
| POPTR_0009s01240.1|PACid:18228577 | metabolic process | binding |
| POPTR_0001s24390.1|PACid:18237160 | - | - |
| POPTR_0010s09140.1|PACid:18239985 | metabolic process, response to chemical stimulus | adenylyltransferase activity |
| POPTR_0008s13590.1|PACid:18249173 | metabolic process | catalytic activity |
| POPTR_0008s15880.1|PACid:18249729 | metabolic process, response to chemical stimulus | adenylyltransferase activity |
|  |  |  |
| **oeu_miR395g** |  |  |
| POPTR_0005s23530.1|PACid:18206966 | inorganic anion transport | transporter activity |
| POPTR_0009s03390.1|PACid:18228182 | - | - |
| POPTR_0009s03390.2|PACid:18228183 | - | - |
| POPTR_0009s01240.1|PACid:18228577 | metabolic process | binding |
| POPTR_0001s24390.1|PACid:18237160 | - | - |
| POPTR_0010s09140.1|PACid:18239985 | metabolic process, response to chemical stimulus | adenylyltransferase activity |
| POPTR_0008s13590.1|PACid:18249173 | metabolic process | catalytic activity |
| POPTR_0008s15880.1|PACid:18249729 | metabolic process, response to chemical stimulus | adenylyltransferase activity |
|  |  |  |
| **oeu_miR395h** |  |  |
| POPTR_0005s23530.1|PACid:18206966 | inorganic anion transport | transporter activity |
| POPTR_0009s03390.1|PACid:18228182 | - | - |
| POPTR_0009s03390.2|PACid:18228183 | - | - |
| POPTR_0009s01240.1|PACid:18228577 | metabolic process | binding |
| POPTR_0001s24390.1|PACid:18237160 | - | - |
| POPTR_0010s09140.1|PACid:18239985 | metabolic process, response to chemical stimulus | adenylyltransferase activity |
| POPTR_0008s13590.1|PACid:18249173 | metabolic process | catalytic activity |
| POPTR_0008s15880.1|PACid:18249729 | metabolic process, response to chemical stimulus | adenylyltransferase activity |
|  |  |  |
| **oeu_miR395i** |  |  |
| POPTR_0005s23530.1|PACid:18206966 | inorganic anion transport | transporter activity |
| POPTR_0009s03390.1|PACid:18228182 | - | - |
| POPTR_0009s03390.2|PACid:18228183 | - | - |
| POPTR_0009s01240.1|PACid:18228577 | metabolic process | binding |
| POPTR_0001s24390.1|PACid:18237160 | - | - |
| POPTR_0010s09140.1|PACid:18239985 | metabolic process, response to chemical stimulus | adenylyltransferase activity |
| POPTR_0008s13590.1|PACid:18249173 | metabolic process | catalytic activity |
| POPTR_0008s15880.1|PACid:18249729 | metabolic process, response to chemical stimulus | adenylyltransferase activity |
|  |  |  |
| **oeu_miR395j** |  |  |
| POPTR_0005s23530.1|PACid:18206966 | inorganic anion transport | transporter activity |
| POPTR_0009s03390.1|PACid:18228182 | - | - |
| POPTR_0009s03390.2|PACid:18228183 | - | - |
| POPTR_0009s01240.1|PACid:18228577 | metabolic process | binding |
| POPTR_0001s24390.1|PACid:18237160 | - | - |
| POPTR_0010s09140.1|PACid:18239985 | metabolic process, response to chemical stimulus | adenylyltransferase activity |
| POPTR_0008s13590.1|PACid:18249173 | metabolic process | catalytic activity |
| POPTR_0008s15880.1|PACid:18249729 | metabolic process, response to chemical stimulus | adenylyltransferase activity |
|  |  |  |
| **oeu_miR396a** |  |  |
| POPTR_0017s03410.1|PACid:18209274 | biological regulation, negative regulation of molecular function | catalytic activity, hydrolase activity |
| POPTR_0006s20610.1|PACid:18212766 | - | - |
| POPTR_0006s20610.2|PACid:18212767 | - | - |
| POPTR_0006s06020.1|PACid:18213545 | - | - |
| POPTR_0006s06020.2|PACid:18213546 | - | - |
| POPTR_0018s12710.1|PACid:18214775 | - | - |
| POPTR_0014s07310.1|PACid:18222886 | metabolic process | methyltransferase activity |
| POPTR_0014s11190.1|PACid:18223588 | - | - |
| POPTR_0014s11190.2|PACid:18223589 | - | - |
| POPTR_0001s25460.1|PACid:18238148 | - | hydrolase activity |
| POPTR_0001s25460.2|PACid:18238149 | - | hydrolase activity |
| POPTR_0007s14310.1|PACid:18243280 | organismal development, reproductive developmental process, flower development | binding |
| POPTR_0016s04810.1|PACid:18250686 | - | - |
| POPTR_0025s00350.1|PACid:18251432 | - | - |
|  |  |  |
| **oeu_miR396b** |  |  |
| POPTR_0017s03410.1|PACid:18209274 | biological regulation, negative regulation of molecular function | catalytic activity, hydrolase activity |
| POPTR_0006s20610.1|PACid:18212766 | - | - |
| POPTR_0006s20610.2|PACid:18212767 | - | - |
| POPTR_0006s06020.1|PACid:18213545 | - | - |
| POPTR_0006s06020.2|PACid:18213546 | - | - |
| POPTR_0018s12710.1|PACid:18214775 | - | - |
| POPTR_0014s07310.1|PACid:18222886 | metabolic process | methyltransferase activity |
| POPTR_0014s11190.1|PACid:18223588 | - | - |
| POPTR_0014s11190.2|PACid:18223589 | - | - |
| POPTR_0001s25460.1|PACid:18238148 | - | hydrolase activity |
| POPTR_0001s25460.2|PACid:18238149 | - | hydrolase activity |
| POPTR_0007s14310.1|PACid:18243280 | organismal development, reproductive developmental process, flower development | binding |
| POPTR_0016s04810.1|PACid:18250686 | - | - |
| POPTR_0025s00350.1|PACid:18251432 | - | - |
|  |  |  |
| **oeu_miR396c** |  |  |
| POPTR_0005s18520.1|PACid:18207134 | metabolic process | oxidoreductase activity |
| POPTR_0017s03410.1|PACid:18209274 | biological regulation, negative regulation of molecular function | catalytic activity, hydrolase activity |
| POPTR_0017s12870.1|PACid:18210697 | metabolic process | binding |
| POPTR_0006s26980.1|PACid:18213411 | organismal development, reproductive developmental process, flower development | binding, transferase activity |
| POPTR_0018s12710.1|PACid:18214775 | - | - |
| POPTR_0019s08260.1|PACid:18218705 | - | - |
| POPTR_0019s08260.3|PACid:18218706 | - | - |
| POPTR_0019s08260.2|PACid:18218707 | - | - |
| POPTR_0014s07310.1|PACid:18222886 | metabolic process | methyltransferase activity |
| POPTR_0014s11190.1|PACid:18223588 | - | - |
| POPTR_0014s11190.2|PACid:18223589 | - | - |
| POPTR_0001s00600.1|PACid:18238020 | - | - |
| POPTR_0007s05710.1|PACid:18243084 | metabolic process | binding, oxidoreductase activity |
| POPTR_0007s10650.1|PACid:18243302 | metabolic process | oxidoreductase activity |
| POPTR_0016s04810.1|PACid:18250686 | - | - |
| POPTR_0016s08320.1|PACid:18250724 | cellular process, microtubule-based process | binding |
|  |  |  |
| **oeu_miR396d** |  |  |
| POPTR_0005s18520.1|PACid:18207134 | metabolic process | oxidoreductase activity |
| POPTR_0017s03410.1|PACid:18209274 | biological regulation, negative regulation of molecular function | catalytic activity, hydrolase activity |
| POPTR_0017s12870.1|PACid:18210697 | metabolic process | binding |
| POPTR_0006s26980.1|PACid:18213411 | organismal development, reproductive developmental process, flower development | binding, transferase activity |
| POPTR_0018s12710.1|PACid:18214775 | - | - |
| POPTR_0019s08260.1|PACid:18218705 | - | - |
| POPTR_0019s08260.3|PACid:18218706 | - | - |
| POPTR_0019s08260.2|PACid:18218707 | - | - |
| POPTR_0014s07310.1|PACid:18222886 | metabolic process | methyltransferase activity |
| POPTR_0014s11190.1|PACid:18223588 | - | - |
| POPTR_0014s11190.2|PACid:18223589 | - | - |
| POPTR_0001s00600.1|PACid:18238020 | - | - |
| POPTR_0007s05710.1|PACid:18243084 | metabolic process | binding, oxidoreductase activity |
| POPTR_0007s10650.1|PACid:18243302 | metabolic process | oxidoreductase activity |
| POPTR_0016s04810.1|PACid:18250686 | - | - |
| POPTR_0016s08320.1|PACid:18250724 | cellular process, microtubule-based process | binding |
|  |  |  |
| **oeu_miR396e** |  |  |
| POPTR_0005s18520.1|PACid:18207134 | metabolic process | oxidoreductase activity |
| POPTR_0017s03410.1|PACid:18209274 | biological regulation, negative regulation of molecular function | catalytic activity, hydrolase activity |
| POPTR_0017s12870.1|PACid:18210697 | metabolic process | binding |
| POPTR_0006s26980.1|PACid:18213411 | organismal development, reproductive developmental process, flower development | binding, transferase activity |
| POPTR_0018s12710.1|PACid:18214775 | - | - |
| POPTR_0019s08260.1|PACid:18218705 | - | - |
| POPTR_0019s08260.3|PACid:18218706 | - | - |
| POPTR_0019s08260.2|PACid:18218707 | - | - |
| POPTR_0014s07310.1|PACid:18222886 | metabolic process | methyltransferase activity |
| POPTR_0014s11190.1|PACid:18223588 | - | - |
| POPTR_0014s11190.2|PACid:18223589 | - | - |
| POPTR_0001s00600.1|PACid:18238020 | - | - |
| POPTR_0007s05710.1|PACid:18243084 | metabolic process | binding, oxidoreductase activity |
| POPTR_0007s10650.1|PACid:18243302 | metabolic process | oxidoreductase activity |
| POPTR_0016s04810.1|PACid:18250686 | - | - |
| POPTR_0016s08320.1|PACid:18250724 | cellular process, microtubule-based process | binding |
|  |  |  |
| **oeu_miR396f** |  |  |
| POPTR_0005s26800.1|PACid:18208606 | - | - |
| POPTR_0018s12710.1|PACid:18214775 | - | - |
| POPTR_0013s09210.1|PACid:18221799 | - | - |
| POPTR_0014s19510.1|PACid:18222251 | - | - |
| POPTR_0009s04590.1|PACid:18227434 | - | - |
| POPTR_0001s12700.1|PACid:18235200 | - | - |
| POPTR_0001s00600.1|PACid:18238020 | - | - |
| POPTR_0001s47300.1|PACid:18239340 | - | - |
| POPTR_0002s11150.1|PACid:18244442 | - | - |
|  |  |  |
| **oeu_miR396g** |  |  |
| POPTR_0005s12000.1|PACid:18207004 | - | - |
| POPTR_0017s12870.1|PACid:18210697 | metabolic process | binding |
| POPTR_0018s12710.1|PACid:18214775 | - | - |
| POPTR_0013s13340.1|PACid:18221696 | - | - |
| POPTR_0013s09210.1|PACid:18221799 | - | - |
| POPTR_0014s19510.1|PACid:18222251 | - | - |
| POPTR_0014s07310.1|PACid:18222886 | metabolic process | methyltransferase activity |
| POPTR_0014s11190.1|PACid:18223588 | - | - |
| POPTR_0014s11190.2|PACid:18223589 | - | - |
| POPTR_0009s04590.1|PACid:18227434 | - | - |
| POPTR_0001s12700.1|PACid:18235200 | - | - |
| POPTR_0001s00600.1|PACid:18238020 | - | - |
| POPTR_0001s47300.1|PACid:18239340 | - | - |
|  |  |  |
| **oeu_miR397a** |  |  |
| POPTR_0005s23060.1|PACid:18206301 | organismal development, reproductive developmental process | binding |
| POPTR_0006s09830.1|PACid:18211225 | lignin metabolic process | oxidoreductase activity |
| POPTR_0006s09840.1|PACid:18213015 | lignin metabolic process | oxidoreductase activity |
| POPTR_0006s09520.1|PACid:18213072 | lignin metabolic process | oxidoreductase activity |
| POPTR_0006s08780.1|PACid:18213801 | lignin metabolic process | oxidoreductase activity |
| POPTR_0006s08740.1|PACid:18213880 | lignin metabolic process | oxidoreductase activity |
| POPTR_0958s00200.1|PACid:18220180 | lignin metabolic process | oxidoreductase activity |
| POPTR_0014s10160.1|PACid:18223297 | biological regulation, metabolic process | signal transducer activity |
| POPTR_0004s16370.1|PACid:18226496 | lignin metabolic process | oxidoreductase activity |
| POPTR_0009s15860.1|PACid:18227298 | lignin metabolic process | oxidoreductase activity |
| POPTR_0009s04720.1|PACid:18227330 | lignin metabolic process | oxidoreductase activity |
| POPTR_0009s15840.1|PACid:18227693 | lignin metabolic process | oxidoreductase activity |
| POPTR_0009s03940.1|PACid:18228008 | lignin metabolic process | oxidoreductase activity |
| POPTR_0009s10550.1|PACid:18228529 | lignin metabolic process | oxidoreductase activity |
| POPTR_0011s12090.1|PACid:18231067 | lignin metabolic process | oxidoreductase activity |
| POPTR_0011s04090.1|PACid:18231177 | - | - |
| POPTR_0011s12100.1|PACid:18231397 | lignin metabolic process | oxidoreductase activity |
| POPTR_0001s41155.1|PACid:18235989 | - | oxidoreductase activity |
| POPTR_0001s25580.1|PACid:18236016 | lignin metabolic process | oxidoreductase activity |
| POPTR_0001s41160.1|PACid:18236745 | lignin metabolic process | oxidoreductase activity |
| POPTR_0001s35740.1|PACid:18237113 | lignin metabolic process | oxidoreductase activity |
| POPTR_0001s14010.1|PACid:18237799 | lignin metabolic process | oxidoreductase activity |
| POPTR_0001s41170.1|PACid:18237813 | lignin metabolic process | oxidoreductase activity |
| POPTR_0001s18500.1|PACid:18238627 | lignin metabolic process | oxidoreductase activity |
| POPTR_0010s19090.1|PACid:18240097 | lignin metabolic process | oxidoreductase activity |
| POPTR_0007s13050.1|PACid:18243260 | lignin metabolic process | oxidoreductase activity |
| POPTR_0091s00270.1|PACid:18247201 | lignin metabolic process | oxidoreductase activity |
| POPTR_0008s07370.1|PACid:18248447 | lignin metabolic process | oxidoreductase activity |
| POPTR_0016s11950.1|PACid:18250097 | lignin metabolic process | oxidoreductase activity |
| POPTR_0016s11540.1|PACid:18250517 | lignin metabolic process | oxidoreductase activity |
| POPTR_0016s11500.1|PACid:18250597 | lignin metabolic process | oxidoreductase activity |
| POPTR_0016s11960.1|PACid:18250678 | lignin metabolic process | oxidoreductase activity |
| POPTR_0016s11520.1|PACid:18251291 | lignin metabolic process | oxidoreductase activity |
|  |  |  |
| **oeu_miR397b** |  |  |
| POPTR_0006s09830.1|PACid:18211225 | lignin metabolic process | oxidoreductase activity |
| POPTR_0006s09840.1|PACid:18213015 | lignin metabolic process | oxidoreductase activity |
| POPTR_0006s09520.1|PACid:18213072 | lignin metabolic process | oxidoreductase activity |
| POPTR_0006s08740.1|PACid:18213880 | lignin metabolic process | oxidoreductase activity |
| POPTR_0958s00200.1|PACid:18220180 | lignin metabolic process | oxidoreductase activity |
| POPTR_0014s10160.1|PACid:18223297 | lignin metabolic process | signal transducer activity |
| POPTR_0004s16370.1|PACid:18226496 | lignin metabolic process | oxidoreductase activity |
| POPTR_0004s13470.1|PACid:18226709 | - | - |
| POPTR_0004s13470.2|PACid:18226710 | - | - |
| POPTR_0009s15860.1|PACid:18227298 | lignin metabolic process | oxidoreductase activity |
| POPTR_0009s04720.1|PACid:18227330 | lignin metabolic process | oxidoreductase activity |
| POPTR_0009s15840.1|PACid:18227693 | lignin metabolic process | oxidoreductase activity |
| POPTR_0009s03940.1|PACid:18228008 | lignin metabolic process | oxidoreductase activity |
| POPTR_0009s10550.1|PACid:18228529 | lignin metabolic process | oxidoreductase activity |
| POPTR_0011s12090.1|PACid:18231067 | lignin metabolic process | oxidoreductase activity |
| POPTR_0011s12100.1|PACid:18231397 | lignin metabolic process | oxidoreductase activity |
| POPTR_0001s25580.1|PACid:18236016 | lignin metabolic process | oxidoreductase activity |
| POPTR_0001s41160.1|PACid:18236745 | lignin metabolic process | oxidoreductase activity |
| POPTR_0001s14010.1|PACid:18237799 | lignin metabolic process | oxidoreductase activity |
| POPTR_0001s41170.1|PACid:18237813 | lignin metabolic process | oxidoreductase activity |
| POPTR_0001s18500.1|PACid:18238627 | lignin metabolic process | oxidoreductase activity |
| POPTR_0010s19090.1|PACid:18240097 | lignin metabolic process | oxidoreductase activity |
| POPTR_0008s07370.1|PACid:18248447 | lignin metabolic process | oxidoreductase activity |
| POPTR_0016s11950.1|PACid:18250097 | lignin metabolic process | oxidoreductase activity |
| POPTR_0016s11500.1|PACid:18250597 | lignin metabolic process | oxidoreductase activity |
| POPTR_0016s11960.1|PACid:18250678 | lignin metabolic process | oxidoreductase activity |
|  |  |  |
| **oeu_miR399b** |  |  |
| POPTR_0012s13740.1|PACid:18229240 | - | - |
|  |  |  |
| **oeu_miR399c** |  |  |
| POPTR_0012s13740.1|PACid:18229240 | - | - |
|  |  |  |
| **oeu_miR399f** |  |  |
| POPTR_0019s08990.1|PACid:18218766 | transport, localization | transporter activity |
| POPTR_0014s02900.1|PACid:18223049 | - | - |
| POPTR_0014s02900.2|PACid:18223050 | - | - |
| POPTR_0012s13740.1|PACid:18229240 | - | - |
| POPTR_0012s11760.1|PACid:18229819 | - | - |
| POPTR_0001s44150.1|PACid:18238919 | - | - |
| POPTR_0008s00770.1|PACid:18249606 | - | - |
|  |  |  |
| **oeu_miR399g** |  |  |
| POPTR_0019s08990.1|PACid:18218766 | transport, localization | transporter activity |
| POPTR_0014s02900.1|PACid:18223049 | - | - |
| POPTR_0014s02900.2|PACid:18223050 | - | - |
| POPTR_0012s13740.1|PACid:18229240 | - | - |
| POPTR_0012s11760.1|PACid:18229819 | - | - |
| POPTR_0001s44150.1|PACid:18238919 | - | - |
| POPTR_0008s00770.1|PACid:18249606 | - | - |
|  |  |  |
| **oeu_miR399i** |  |  |
| POPTR_0019s08990.1|PACid:18218766 | ion transport | transporter activity |
| POPTR_0012s13740.1|PACid:18229240 | - | - |
|  |  |  |
| **oeu_miR408** |  |  |
| POPTR_0017s04300.1|PACid:18209262 | - | transferase activity |
| POPTR_0019s14530.1|PACid:18218564 | lignin metabolic process | oxidoreductase activity |
| POPTR_0013s14890.1|PACid:18221306 | lignin metabolic process | oxidoreductase activity |
| POPTR_0013s13650.1|PACid:18221641 | - | - |
| POPTR_0004s00750.1|PACid:18224965 | - | - |
| POPTR_0011s01460.1|PACid:18230556 | - | - |
| POPTR_0001s21660.1|PACid:18234854 | - | binding |
| POPTR_0001s04640.1|PACid:18235761 | - | - |
| POPTR_0001s15240.1|PACid:18235841 | unannotated | - |
| POPTR_0796s00210.1|PACid:18239434 | - | - |
| POPTR_0010s19080.1|PACid:18242197 | lignin metabolic process | oxidoreductase activity |
| POPTR_0002s18910.1|PACid:18246593 | - | - |
| POPTR_0008s07230.1|PACid:18248264 | biological regulation | - |
|  |  |  |
| **oeu_miR530a** |  |  |
| POPTR_0005s05590.1|PACid:18207126 | - | - |
| POPTR_0006s24170.1|PACid:18211457 | - | - |
| POPTR_0006s11260.1|PACid:18211952 | - | - |
| POPTR_0006s19420.1|PACid:18212727 | - | - |
| POPTR_0018s11180.1|PACid:18214897 | - | - |
| POPTR_0003s05100.1|PACid:18217925 | - | - |
| POPTR_0013s00860.1|PACid:18220255 | - | - |
| POPTR_0013s03100.1|PACid:18220657 | - | - |
| POPTR_0013s03810.1|PACid:18221454 | - | - |
| POPTR_0013s12820.1|PACid:18221738 | - | - |
| POPTR_0013s03120.1|PACid:18221827 | - | - |
| POPTR_0014s09520.1|PACid:18223713 | - | - |
| POPTR_0004s06040.1|PACid:18225986 | - | - |
| POPTR_0009s15750.1|PACid:18227610 | - | - |
| POPTR_0009s04140.1|PACid:18227867 | - | - |
| POPTR_0011s06790.1|PACid:18231309 | - | - |
| POPTR_0015s05470.1|PACid:18233077 | - | - |
| POPTR_0015s09780.1|PACid:18233670 | - | - |
| POPTR_0015s09780.2|PACid:18233671 | - | - |
| POPTR_0001s25120.1|PACid:18234368 | - | - |
| POPTR_0001s25120.2|PACid:18234369 | - | - |
| POPTR_0001s25120.3|PACid:18234370 | - | - |
| POPTR_0001s11180.1|PACid:18235469 | - | - |
| POPTR_0001s32500.1|PACid:18236844 | - | - |
| POPTR_0001s19380.1|PACid:18238614 | - | - |
| POPTR_1333s00200.1|PACid:18239450 | - | - |
| POPTR_0010s07230.1|PACid:18239905 | - | - |
| POPTR_0010s08720.1|PACid:18241753 | - | - |
| POPTR_0010s11600.1|PACid:18241916 | - | - |
| POPTR_0007s04910.1|PACid:18243324 | - | - |
| POPTR_0002s15230.1|PACid:18245496 | - | - |
| POPTR_0008s17490.1|PACid:18248253 | - | - |
| POPTR_0008s13490.1|PACid:18248324 | - | - |
| POPTR_0008s16240.1|PACid:18248878 | - | - |
| POPTR_0008s22090.1|PACid:18249166 | - | - |
| POPTR_0016s12770.1|PACid:18250070 | - | - |
| POPTR_0016s14810.1|PACid:18250938 | - | - |
