# Supplementary material for: Genome-wide identification of alternate bearing-associated microRNAs (miRNAs) in olive (Olea europaea L.)
Source: BMC Plant Biol. 2013 Jan 15;13:10. doi: 10.1186/1471-2229-13-10 (PMC3564680; doi:10.1186/1471-2229-13-10)
Supplement: Additional file 5 — The most abundant gene ontology terms from the biological function ontology. Eight ontology terms were found with significant abundance (p <0.05). [file 1471-2229-13-10-S5.doc]

**Additional file 4: The** **most abundant gene ontology terms from biological function ontology.** Eight ontology terms were found with significant abundance (p<0.05).

|  | **HM** | | | **OM** | | | **VK** | | | **VT** | | | **YK** | | | **YT** | | |
| --- | --- | --- | --- | --- | --- | --- | --- | --- | --- | --- | --- | --- | --- | --- | --- | --- | --- | --- |
| **Gene Ontology Term** | **Cluster frequency** | **Genome frequency of use** | **Corrected P-value** | **Cluster frequency** | **Genome frequency of use** | **Corrected P-value** | **Cluster frequency** | **Genome frequency of use** | **Corrected P-value** | **Cluster frequency** | **Genome frequency of use** | **Corrected P-value** | **Cluster frequency** | **Genome frequency of use** | **Corrected P-value** | **Cluster frequency** | **Genome frequency of use** | **Corrected P-value** |
| [**oxidoreductase activity, acting on diphenols and related substances as donors, oxygen as acceptor**](http://amigo.geneontology.org/cgi-bin/amigo/go.cgi?action=query&view=query&query=GO:0016682&search_constraint=terms) | 33 out of 228 genes, 14.5% | 105 out of 25596 genes, 0.4% | 3.02E-40 | 33 out of 216 genes, 15.3% | 105 out of 25596 genes, 0.4% | 4.26E-41 | 33 out of 241 genes, 13.7% | 105 out of 25596 genes, 0.4% | 1.95E-39 | 33 out of 280 genes, 11.8% | 105 out of 25596 genes, 0.4% | 3.65E-37 | 33 out of 278 genes, 11.9% | 105 out of 25596 genes, 0.4% | 2.90E-37 | 33 out of 263 genes, 12.5% | 105 out of 25596 genes, 0.4% | 4.21E-38 |
| [**oxidoreductase activity, acting on diphenols and related substances as donors**](http://amigo.geneontology.org/cgi-bin/amigo/go.cgi?action=query&view=query&query=GO:0016679&search_constraint=terms) | 33 out of 228 genes, 14.5% | 110 out of 25596 genes, 0.4% | 1.80E-39 | 33 out of 216 genes, 15.3% | 110 out of 25596 genes, 0.4% | 2.55E-40 | 33 out of 241 genes, 13.7% | 110 out of 25596 genes, 0.4% | 1.16E-38 | 33 out of 280 genes, 11.8% | 110 out of 25596 genes, 0.4% | 2.16E-36 | 33 out of 278 genes, 11.9% | 110 out of 25596 genes, 0.4% | 1.71E-36 | 33 out of 263 genes, 12.5% | 110 out of 25596 genes, 0.4% | 2.50E-37 |
| [**oxidoreductase activity**](http://amigo.geneontology.org/cgi-bin/amigo/go.cgi?action=query&view=query&query=GO:0016491&search_constraint=terms) | 71 out of 228 genes, 31.1% | 3177 out of 25596 genes, 12.4% | 8.37E-12 | 70 out of 216 genes, 32.4% | 3177 out of 25596 genes, 12.4% | 1.26E-12 | 70 out of 241 genes, 29.0% | 3177 out of 25596 genes, 12.4% | 4.81E-10 | 73 out of 280 genes, 26.1% | 3177 out of 25596 genes, 12.4% | 4.51E-08 | 73 out of 278 genes, 26.3% | 3177 out of 25596 genes, 12.4% | 3.23E-08 | 72 out of 263 genes, 27.4% | 3177 out of 25596 genes, 12.4% | 5.35E-09 |
| [**alcohol binding**](http://amigo.geneontology.org/cgi-bin/amigo/go.cgi?action=query&view=query&query=GO:0043178&search_constraint=terms) | 4 out of 228 genes, 1.8% | 8 out of 25596 genes, 0.0% | 5.38E-05 | 4 out of 216 genes, 1.9% | 8 out of 25596 genes, 0.0% | 4.06E-05 | 4 out of 241 genes, 1.7% | 8 out of 25596 genes, 0.0% | 6.30E-05 | 4 out of 280 genes, 1.4% | 8 out of 25596 genes, 0.0% | 0.00012 | 4 out of 278 genes, 1.4% | 8 out of 25596 genes, 0.0% | 0.00011 | 218 out of 263 genes, 82.9% | 17286 out of 25596 genes, 67.5% | 1.62E-06 |
| [**binding**](http://amigo.geneontology.org/cgi-bin/amigo/go.cgi?action=query&view=query&query=GO:0005488&search_constraint=terms) | 187 out of 228 genes, 82.0% | 17286 out of 25596 genes, 67.5% | 7.91E-05 | 177 out of 216 genes, 81.9% | 17286 out of 25596 genes, 67.5% | 0.00016 | 200 out of 241 genes, 83.0% | 17286 out of 25596 genes, 67.5% | 5.06E-06 | 226 out of 280 genes, 80.7% | 17286 out of 25596 genes, 67.5% | 6.87E-05 | 225 out of 278 genes, 80.9% | 17286 out of 25596 genes, 67.5% | 4.89E-05 | 4 out of 263 genes, 1.5% | 8 out of 25596 genes, 0.0% | 9.37E-05 |
| [**hormone binding**](http://amigo.geneontology.org/cgi-bin/amigo/go.cgi?action=query&view=query&query=GO:0042562&search_constraint=terms) | 4 out of 228 genes, 1.8% | 9 out of 25596 genes, 0.0% | 9.62E-05 | 4 out of 216 genes, 1.9% | 9 out of 25596 genes, 0.0% | 7.27E-05 | 4 out of 241 genes, 1.7% | 9 out of 25596 genes, 0.0% | 0.00011 | 4 out of 280 genes, 1.4% | 9 out of 25596 genes, 0.0% | 0.00021 | 4 out of 278 genes, 1.4% | 9 out of 25596 genes, 0.0% | 0.00021 | 4 out of 263 genes, 1.5% | 9 out of 25596 genes, 0.0% | 0.00016 |
| [**nucleic acid binding**](http://amigo.geneontology.org/cgi-bin/amigo/go.cgi?action=query&view=query&query=GO:0003676&search_constraint=terms) | 59 out of 228 genes, 25.9% | 3926 out of 25596 genes, 15.3% | 0.00322 | 59 out of 216 genes, 27.3% | 3926 out of 25596 genes, 15.3% | 0.00051 | 68 out of 241 genes, 28.2% | 3926 out of 25596 genes, 15.3% | 2.68E-05 | 73 out of 280 genes, 26.1% | 3926 out of 25596 genes, 15.3% | 0.00028 | 75 out of 278 genes, 27.0% | 3926 out of 25596 genes, 15.3% | 5.05E-05 | 70 out of 263 genes, 26.6% | 3926 out of 25596 genes, 15.3% | 0.0002 |
| [**transition metal ion binding**](http://amigo.geneontology.org/cgi-bin/amigo/go.cgi?action=query&view=query&query=GO:0046914&search_constraint=terms) | 56 out of 228 genes, 24.6% | 3709 out of 25596 genes, 14.5% | 0.00489 | 52 out of 216 genes, 24.1% | 3709 out of 25596 genes, 14.5% | 0.01474 |  |  |  | 62 out of 280 genes, 22.1% | 3709 out of 25596 genes, 14.5% | 0.04576 |  |  |  |  |  |  |
