# Supplementary material for: Genome-wide identification of alternate bearing-associated microRNAs (miRNAs) in olive (Olea europaea L.)
Source: BMC Plant Biol. 2013 Jan 15;13:10. doi: 10.1186/1471-2229-13-10 (PMC3564680; doi:10.1186/1471-2229-13-10)
Supplement: Additional file 6 — The most abundant gene ontology terms from the molecular process ontology. A total of 108 ontology terms were found with significant abundance (p <0.05). [file 1471-2229-13-10-S6.doc]

**Additional file 5: The most abundant gene ontology terms from molecular process ontology. 108 ontology terms were found with significant abundance (p<0.05)**

|  | **HM** | | | **OM** | | | **VK** | | | **VT** | | | **YK** | | | **YT** | | |
| --- | --- | --- | --- | --- | --- | --- | --- | --- | --- | --- | --- | --- | --- | --- | --- | --- | --- | --- |
| **Gene Ontology term** | **Cluster frequency** | **Genome frequency of use** | **Corrected P-value** | **Cluster frequency** | **Genome frequency of use** | **Corrected P-value** | **Cluster frequency** | **Genome frequency of use** | **Corrected P-value** | **Cluster frequency** | **Genome frequency of use** | **Corrected P-value** | **Cluster frequency** | **Genome frequency of use** | **Corrected P-value** | **Cluster frequency** | **Genome frequency of use** | **Corrected P-value** |
| **cellular metabolic process** | 151 out of 206 genes, 73.3% | 11088 out of 22088 genes, 50.2% | 2.99E-09 | 148 out of 197 genes, 75.1% | 11088 out of 22088 genes, 50.2% | 1.78E-10 | 165 out of 220 genes, 75.0% | 11088 out of 22088 genes, 50.2% | 1.10E-11 | 191 out of 255 genes, 74.9% | 11088 out of 22088 genes, 50.2% | 1.43E-13 | 191 out of 257 genes, 74.3% | 11088 out of 22088 genes, 50.2% | 5.66E-13 | 174 out of 242 genes, 71.9% | 11088 out of 22088 genes, 50.2% | 1.36E-09 |
| **primary metabolic process** | 152 out of 206 genes, 73.8% | 11478 out of 22088 genes, 52.0% | 3.12E-08 | 146 out of 197 genes, 74.1% | 11478 out of 22088 genes, 52.0% | 4.14E-08 | 167 out of 220 genes, 75.9% | 11478 out of 22088 genes, 52.0% | 5.85E-11 | 194 out of 255 genes, 76.1% | 11478 out of 22088 genes, 52.0% | 4.70E-13 | 196 out of 257 genes, 76.3% | 11478 out of 22088 genes, 52.0% | 2.17E-13 | 178 out of 242 genes, 73.6% | 11478 out of 22088 genes, 52.0% | 1.31E-09 |
| **cellular process** | 162 out of 206 genes, 78.6% | 14561 out of 22088 genes, 65.9% | 0.01358 | 159 out of 197 genes, 80.7% | 14561 out of 22088 genes, 65.9% | 0.00098 | 180 out of 220 genes, 81.8% | 14561 out of 22088 genes, 65.9% | 3.56E-05 | 207 out of 255 genes, 81.2% | 14561 out of 22088 genes, 65.9% | 1.49E-05 | 208 out of 257 genes, 80.9% | 14561 out of 22088 genes, 65.9% | 2.23E-05 | 194 out of 242 genes, 80.2% | 14561 out of 22088 genes, 65.9% | 0.00022 |
| **biopolymer metabolic process** | 102 out of 206 genes, 49.5% | 7973 out of 22088 genes, 36.1% | 0.01658 | 97 out of 197 genes, 49.2% | 7973 out of 22088 genes, 36.1% | 0.03185 | 118 out of 220 genes, 53.6% | 7973 out of 22088 genes, 36.1% | 2.33E-05 | 140 out of 255 genes, 54.9% | 7973 out of 22088 genes, 36.1% | 2.00E-07 | 140 out of 257 genes, 54.5% | 7973 out of 22088 genes, 36.1% | 4.10E-07 | 124 out of 242 genes, 51.2% | 7973 out of 22088 genes, 36.1% | 0.00031 |
| **cellular biopolymer metabolic process** | 95 out of 206 genes, 46.1% | 7128 out of 22088 genes, 32.3% | 0.00708 | 94 out of 197 genes, 47.7% | 7128 out of 22088 genes, 32.3% | 0.00134 | 111 out of 220 genes, 50.5% | 7128 out of 22088 genes, 32.3% | 4.78E-06 | 132 out of 255 genes, 51.8% | 7128 out of 22088 genes, 32.3% | 2.53E-08 | 132 out of 257 genes, 51.4% | 7128 out of 22088 genes, 32.3% | 5.08E-08 | 117 out of 242 genes, 48.3% | 7128 out of 22088 genes, 32.3% | 4.31E-05 |
| **cellular macromolecule metabolic process** | 95 out of 206 genes, 46.1% | 7213 out of 22088 genes, 32.7% | 0.0119 | 94 out of 197 genes, 47.7% | 7213 out of 22088 genes, 32.7% | 0.00234 | 111 out of 220 genes, 50.5% | 7213 out of 22088 genes, 32.7% | 9.80E-06 | 133 out of 255 genes, 52.2% | 7213 out of 22088 genes, 32.7% | 2.70E-08 | 133 out of 257 genes, 51.8% | 7213 out of 22088 genes, 32.7% | 5.45E-08 | 117 out of 242 genes, 48.3% | 7213 out of 22088 genes, 32.7% | 8.68E-05 |
| **biological regulation** | 64 out of 206 genes, 31.1% | 2546 out of 22088 genes, 11.5% | 1.28E-11 | 61 out of 197 genes, 31.0% | 2546 out of 22088 genes, 11.5% | 5.81E-11 | 72 out of 220 genes, 32.7% | 2546 out of 22088 genes, 11.5% | 1.44E-14 | 73 out of 255 genes, 28.6% | 2546 out of 22088 genes, 11.5% | 2.44E-11 | 77 out of 257 genes, 30.0% | 2546 out of 22088 genes, 11.5% | 3.34E-13 | 73 out of 242 genes, 30.2% | 2546 out of 22088 genes, 11.5% | 1.23E-12 |
| **developmental process** | 66 out of 206 genes, 32.0% | 2689 out of 22088 genes, 12.2% | 1.35E-11 | 66 out of 197 genes, 33.5% | 2689 out of 22088 genes, 12.2% | 1.15E-12 | 75 out of 220 genes, 34.1% | 2689 out of 22088 genes, 12.2% | 5.14E-15 | 82 out of 255 genes, 32.2% | 2689 out of 22088 genes, 12.2% | 9.27E-15 | 82 out of 257 genes, 31.9% | 2689 out of 22088 genes, 12.2% | 1.57E-14 | 84 out of 242 genes, 34.7% | 2689 out of 22088 genes, 12.2% | 1.71E-17 |
| **multicellular organismal development** | 55 out of 206 genes, 26.7% | 1714 out of 22088 genes, 7.8% | 5.83E-14 | 55 out of 197 genes, 27.9% | 1714 out of 22088 genes, 7.8% | 6.50E-15 | 63 out of 220 genes, 28.6% | 1714 out of 22088 genes, 7.8% | 7.19E-18 | 65 out of 255 genes, 25.5% | 1714 out of 22088 genes, 7.8% | 1.45E-15 | 69 out of 257 genes, 26.8% | 1714 out of 22088 genes, 7.8% | 6.23E-18 | 68 out of 242 genes, 28.1% | 1714 out of 22088 genes, 7.8% | 7.47E-19 |
| **multicellular organismal process** | 57 out of 206 genes, 27.7% | 1922 out of 22088 genes, 8.7% | 4.65E-13 | 57 out of 197 genes, 28.9% | 1922 out of 22088 genes, 8.7% | 5.02E-14 | 65 out of 220 genes, 29.5% | 1922 out of 22088 genes, 8.7% | 1.14E-16 | 67 out of 255 genes, 26.3% | 1922 out of 22088 genes, 8.7% | 2.89E-14 | 71 out of 257 genes, 27.6% | 1922 out of 22088 genes, 8.7% | 1.76E-16 | 70 out of 242 genes, 28.9% | 1922 out of 22088 genes, 8.7% | 1.90E-17 |
| **regulation of cellular process** | 41 out of 206 genes, 19.9% | 1537 out of 22088 genes, 7.0% | 2.60E-07 | 41 out of 197 genes, 20.8% | 1537 out of 22088 genes, 7.0% | 6.11E-08 | 42 out of 220 genes, 19.1% | 1537 out of 22088 genes, 7.0% | 6.01E-07 | 45 out of 255 genes, 17.6% | 1537 out of 22088 genes, 7.0% | 2.24E-06 | 45 out of 257 genes, 17.5% | 1537 out of 22088 genes, 7.0% | 2.91E-06 | 42 out of 242 genes, 17.4% | 1537 out of 22088 genes, 7.0% | 1.16E-05 |
| **reproduction** | 41 out of 206 genes, 19.9% | 1237 out of 22088 genes, 5.6% | 3.16E-10 | 41 out of 197 genes, 20.8% | 1237 out of 22088 genes, 5.6% | 6.53E-11 | 43 out of 220 genes, 19.5% | 1237 out of 22088 genes, 5.6% | 1.66E-10 | 41 out of 255 genes, 16.1% | 1237 out of 22088 genes, 5.6% | 3.43E-07 | 43 out of 257 genes, 16.7% | 1237 out of 22088 genes, 5.6% | 3.73E-08 | 43 out of 242 genes, 17.8% | 1237 out of 22088 genes, 5.6% | 4.89E-09 |
| **reproductive developmental process** | 41 out of 206 genes, 19.9% | 1054 out of 22088 genes, 4.8% | 1.61E-12 | 41 out of 197 genes, 20.8% | 1054 out of 22088 genes, 4.8% | 3.08E-13 | 43 out of 220 genes, 19.5% | 1054 out of 22088 genes, 4.8% | 6.75E-13 | 41 out of 255 genes, 16.1% | 1054 out of 22088 genes, 4.8% | 2.65E-09 | 43 out of 257 genes, 16.7% | 1054 out of 22088 genes, 4.8% | 2.07E-10 | 43 out of 242 genes, 17.8% | 1054 out of 22088 genes, 4.8% | 2.39E-11 |
| **reproductive process** | 41 out of 206 genes, 19.9% | 1214 out of 22088 genes, 5.5% | 1.72E-10 | 41 out of 197 genes, 20.8% | 1214 out of 22088 genes, 5.5% | 3.53E-11 | 43 out of 220 genes, 19.5% | 1214 out of 22088 genes, 5.5% | 8.85E-11 | 41 out of 255 genes, 16.1% | 1214 out of 22088 genes, 5.5% | 1.97E-07 | 43 out of 257 genes, 16.7% | 1214 out of 22088 genes, 5.5% | 2.06E-08 | 43 out of 242 genes, 17.8% | 1214 out of 22088 genes, 5.5% | 2.66E-09 |
| **response to chemical stimulus** | 43 out of 206 genes, 20.9% | 2432 out of 22088 genes, 11.0% | 0.00855 | 42 out of 197 genes, 21.3% | 2432 out of 22088 genes, 11.0% | 0.00607 |  |  |  | 32 out of 255 genes, 12.5% | 1359 out of 22088 genes, 6.2% | 0.03433 |  |  |  |  |  |  |
| **Anatomical structure development** | 44 out of 206 genes, 21.4% | 1523 out of 22088 genes, 6.9% | 4.08E-09 | 44 out of 197 genes, 22.3% | 1523 out of 22088 genes, 6.9% | 8.04E-10 | 53 out of 220 genes, 24.1% | 1523 out of 22088 genes, 6.9% | 1.61E-13 | 59 out of 255 genes, 23.1% | 1523 out of 22088 genes, 6.9% | 2.83E-14 | 59 out of 257 genes, 23.0% | 1523 out of 22088 genes, 6.9% | 4.22E-14 | 62 out of 242 genes, 25.6% | 1523 out of 22088 genes, 6.9% | 1.95E-17 |
| **RNA biosynthetic process** | 45 out of 206 genes, 21.8% | 1924 out of 22088 genes, 8.7% | 2.06E-06 | 45 out of 197 genes, 22.8% | 1924 out of 22088 genes, 8.7% | 4.54E-07 | 51 out of 220 genes, 23.2% | 1924 out of 22088 genes, 8.7% | 2.12E-08 | 65 out of 255 genes, 25.5% | 1924 out of 22088 genes, 8.7% | 4.40E-13 | 65 out of 257 genes, 25.3% | 1924 out of 22088 genes, 8.7% | 6.72E-13 | 55 out of 242 genes, 22.7% | 1924 out of 22088 genes, 8.7% | 9.21E-09 |
| **transcription** | 45 out of 206 genes, 21.8% | 1924 out of 22088 genes, 8.7% | 2.06E-06 | 45 out of 197 genes, 22.8% | 1924 out of 22088 genes, 8.7% | 4.54E-07 | 51 out of 220 genes, 23.2% | 1924 out of 22088 genes, 8.7% | 2.12E-08 | 65 out of 255 genes, 25.5% | 1924 out of 22088 genes, 8.7% | 4.40E-13 | 65 out of 257 genes, 25.3% | 1924 out of 22088 genes, 8.7% | 6.72E-13 | 55 out of 242 genes, 22.7% | 1924 out of 22088 genes, 8.7% | 9.21E-09 |
| **transcription, DNA-dependent** | 45 out of 206 genes, 21.8% | 1924 out of 22088 genes, 8.7% | 2.06E-06 | 45 out of 197 genes, 22.8% | 1924 out of 22088 genes, 8.7% | 4.54E-07 | 51 out of 220 genes, 23.2% | 1924 out of 22088 genes, 8.7% | 2.12E-08 | 65 out of 255 genes, 25.5% | 1924 out of 22088 genes, 8.7% | 4.40E-13 | 65 out of 257 genes, 25.3% | 1924 out of 22088 genes, 8.7% | 6.72E-13 | 55 out of 242 genes, 22.7% | 1924 out of 22088 genes, 8.7% | 9.21E-09 |
| **biopolymer biosynthetic process** | 47 out of 206 genes, 22.8% | 2500 out of 22088 genes, 11.3% | 0.00061 | 47 out of 197 genes, 23.9% | 2500 out of 22088 genes, 11.3% | 0.00015 | 55 out of 220 genes, 25.0% | 2500 out of 22088 genes, 11.3% | 3.06E-06 | 71 out of 255 genes, 27.8% | 2500 out of 22088 genes, 11.3% | 9.76E-11 | 71 out of 257 genes, 27.6% | 2500 out of 22088 genes, 11.3% | 1.50E-10 | 60 out of 242 genes, 24.8% | 2500 out of 22088 genes, 11.3% | 9.85E-07 |
| **cellular biopolymer biosynthetic process** | 47 out of 206 genes, 22.8% | 2500 out of 22088 genes, 11.3% | 0.00061 | 47 out of 197 genes, 23.9% | 2500 out of 22088 genes, 11.3% | 0.00015 | 55 out of 220 genes, 25.0% | 2500 out of 22088 genes, 11.3% | 3.06E-06 | 71 out of 255 genes, 27.8% | 2500 out of 22088 genes, 11.3% | 9.76E-11 | 71 out of 257 genes, 27.6% | 2500 out of 22088 genes, 11.3% | 1.50E-10 | 60 out of 242 genes, 24.8% | 2500 out of 22088 genes, 11.3% | 9.85E-07 |
| **cellular macromolecule biosynthetic process** | 47 out of 206 genes, 22.8% | 2574 out of 22088 genes, 11.7% | 0.00136 | 47 out of 197 genes, 23.9% | 2574 out of 22088 genes, 11.7% | 0.00035 | 55 out of 220 genes, 25.0% | 2574 out of 22088 genes, 11.7% | 8.44E-06 | 71 out of 255 genes, 27.8% | 2574 out of 22088 genes, 11.7% | 4.02E-10 | 71 out of 257 genes, 27.6% | 2574 out of 22088 genes, 11.7% | 6.13E-10 | 60 out of 242 genes, 24.8% | 2574 out of 22088 genes, 11.7% | 2.95E-06 |
| **macromolecule biosynthetic process** | 47 out of 206 genes, 22.8% | 2574 out of 22088 genes, 11.7% | 0.00136 | 47 out of 197 genes, 23.9% | 2574 out of 22088 genes, 11.7% | 0.00035 | 55 out of 220 genes, 25.0% | 2574 out of 22088 genes, 11.7% | 8.44E-06 | 71 out of 255 genes, 27.8% | 2574 out of 22088 genes, 11.7% | 4.02E-10 | 71 out of 257 genes, 27.6% | 2574 out of 22088 genes, 11.7% | 6.13E-10 | 60 out of 242 genes, 24.8% | 2574 out of 22088 genes, 11.7% | 2.95E-06 |
| **RNA metabolic process** | 47 out of 206 genes, 22.8% | 2452 out of 22088 genes, 11.1% | 0.00035 | 47 out of 197 genes, 23.9% | 2452 out of 22088 genes, 11.1% | 8.80E-05 | 53 out of 220 genes, 24.1% | 2452 out of 22088 genes, 11.1% | 1.12E-05 | 69 out of 255 genes, 27.1% | 2452 out of 22088 genes, 11.1% | 3.68E-10 | 69 out of 257 genes, 26.8% | 2452 out of 22088 genes, 11.1% | 5.57E-10 | 58 out of 242 genes, 24.0% | 2452 out of 22088 genes, 11.1% | 3.34E-06 |
| **regulation of biological process** | 48 out of 206 genes, 23.3% | 1766 out of 22088 genes, 8.0% | 3.33E-09 | 48 out of 197 genes, 24.4% | 1766 out of 22088 genes, 8.0% | 5.78E-10 | 55 out of 220 genes, 25.0% | 1766 out of 22088 genes, 8.0% | 4.44E-12 | 54 out of 255 genes, 21.2% | 1766 out of 22088 genes, 8.0% | 9.56E-09 | 58 out of 257 genes, 22.6% | 1766 out of 22088 genes, 8.0% | 1.07E-10 | 55 out of 242 genes, 22.7% | 1766 out of 22088 genes, 8.0% | 3.21E-10 |
| **cell communication** | 30 out of 206 genes, 14.6% | 1329 out of 22088 genes, 6.0% | 0.00206 | 30 out of 197 genes, 15.2% | 1329 out of 22088 genes, 6.0% | 0.00079 | 31 out of 220 genes, 14.1% | 1329 out of 22088 genes, 6.0% | 0.00283 | 34 out of 255 genes, 13.3% | 1329 out of 22088 genes, 6.0% | 0.0038 | 34 out of 257 genes, 13.2% | 1329 out of 22088 genes, 6.0% | 0.00455 | 31 out of 242 genes, 12.8% | 1329 out of 22088 genes, 6.0% | 0.01976 |
| **response to hormone stimulus** | 30 out of 206 genes, 14.6% | 1274 out of 22088 genes, 5.8% | 0.00089 | 30 out of 197 genes, 15.2% | 1274 out of 22088 genes, 5.8% | 0.00033 | 30 out of 220 genes, 13.6% | 1274 out of 22088 genes, 5.8% | 0.00335 | 31 out of 255 genes, 12.2% | 1274 out of 22088 genes, 5.8% | 0.02451 |  |  |  | 31 out of 242 genes, 12.8% | 1274 out of 22088 genes, 5.8% | 0.00893 |
| **signal transduction** | 30 out of 206 genes, 14.6% | 1162 out of 22088 genes, 5.3% | 0.00013 | 30 out of 197 genes, 15.2% | 1162 out of 22088 genes, 5.3% | 4.87E-05 | 31 out of 220 genes, 14.1% | 1162 out of 22088 genes, 5.3% | 0.00017 |  |  |  | 33 out of 257 genes, 12.8% | 1162 out of 22088 genes, 5.3% | 0.0007 | 31 out of 242 genes, 12.8% | 1162 out of 22088 genes, 5.3% | 0.00146 |
| **response to endogenous stimulus** | 31 out of 206 genes, 15.0% | 1359 out of 22088 genes, 6.2% | 0.00114 | 31 out of 197 genes, 15.7% | 1359 out of 22088 genes, 6.2% | 0.00042 | 31 out of 220 genes, 14.1% | 1359 out of 22088 genes, 6.2% | 0.00439 |  |  |  |  |  |  | 32 out of 242 genes, 13.2% | 1359 out of 22088 genes, 6.2% | 0.0124 |
| **lignin metabolic process** | 32 out of 206 genes, 15.5% | 55 out of 22088 genes, 0.2% | 4.38E-49 | 32 out of 197 genes, 16.2% | 55 out of 22088 genes, 0.2% | 9.13E-50 | 32 out of 220 genes, 14.5% | 55 out of 22088 genes, 0.2% | 4.12E-48 | 32 out of 255 genes, 12.5% | 55 out of 22088 genes, 0.2% | 6.67E-46 | 32 out of 257 genes, 12.5% | 55 out of 22088 genes, 0.2% | 8.79E-46 | 32 out of 242 genes, 13.2% | 55 out of 22088 genes, 0.2% | 1.12E-46 |
| **cellular amino acid derivative metabolic process** | 34 out of 206 genes, 16.5% | 430 out of 22088 genes, 1.9% | 2.55E-19 | 34 out of 197 genes, 17.3% | 430 out of 22088 genes, 1.9% | 5.55E-20 | 34 out of 220 genes, 15.5% | 430 out of 22088 genes, 1.9% | 2.22E-18 | 34 out of 255 genes, 13.3% | 430 out of 22088 genes, 1.9% | 2.82E-16 | 34 out of 257 genes, 13.2% | 430 out of 22088 genes, 1.9% | 3.66E-16 | 34 out of 242 genes, 14.0% | 430 out of 22088 genes, 1.9% | 5.25E-17 |
| **phenylpropanoid metabolic process** | 34 out of 206 genes, 16.5% | 288 out of 22088 genes, 1.3% | 4.61E-25 | 34 out of 197 genes, 17.3% | 288 out of 22088 genes, 1.3% | 9.49E-26 | 34 out of 220 genes, 15.5% | 288 out of 22088 genes, 1.3% | 4.39E-24 | 34 out of 255 genes, 13.3% | 288 out of 22088 genes, 1.3% | 6.97E-22 | 34 out of 257 genes, 13.2% | 288 out of 22088 genes, 1.3% | 9.17E-22 | 34 out of 242 genes, 14.0% | 288 out of 22088 genes, 1.3% | 1.19E-22 |
| **cellular amino acid and derivative metabolic process** | 35 out of 206 genes, 17.0% | 921 out of 22088 genes, 4.2% | 4.17E-10 | 35 out of 197 genes, 17.8% | 921 out of 22088 genes, 4.2% | 1.05E-10 | 35 out of 220 genes, 15.9% | 921 out of 22088 genes, 4.2% | 2.87E-09 | 37 out of 255 genes, 14.5% | 921 out of 22088 genes, 4.2% | 1.26E-08 | 37 out of 257 genes, 14.4% | 921 out of 22088 genes, 4.2% | 1.61E-08 | 36 out of 242 genes, 14.9% | 921 out of 22088 genes, 4.2% | 1.12E-08 |
| **secondary metabolic process** | 35 out of 206 genes, 17.0% | 515 out of 22088 genes, 2.3% | 8.41E-18 | 35 out of 197 genes, 17.8% | 515 out of 22088 genes, 2.3% | 1.79E-18 | 35 out of 220 genes, 15.9% | 515 out of 22088 genes, 2.3% | 7.50E-17 | 35 out of 255 genes, 13.7% | 515 out of 22088 genes, 2.3% | 9.92E-15 | 35 out of 257 genes, 13.6% | 515 out of 22088 genes, 2.3% | 1.28E-14 | 35 out of 242 genes, 14.5% | 515 out of 22088 genes, 2.3% | 1.82E-15 |
| **cellular aromatic compound metabolic process** | 37 out of 206 genes, 18.0% | 512 out of 22088 genes, 2.3% | 7.47E-20 | 37 out of 197 genes, 18.8% | 512 out of 22088 genes, 2.3% | 1.42E-20 | 37 out of 220 genes, 16.8% | 512 out of 22088 genes, 2.3% | 7.82E-19 | 39 out of 255 genes, 15.3% | 512 out of 22088 genes, 2.3% | 2.30E-18 | 39 out of 257 genes, 15.2% | 512 out of 22088 genes, 2.3% | 3.10E-18 | 37 out of 242 genes, 15.3% | 512 out of 22088 genes, 2.3% | 2.39E-17 |
| **organ development** | 37 out of 206 genes, 18.0% | 869 out of 22088 genes, 3.9% | 2.65E-12 | 37 out of 197 genes, 18.8% | 869 out of 22088 genes, 3.9% | 5.87E-13 | 45 out of 220 genes, 20.5% | 869 out of 22088 genes, 3.9% | 1.51E-17 | 45 out of 255 genes, 17.6% | 869 out of 22088 genes, 3.9% | 6.74E-15 | 49 out of 257 genes, 19.1% | 869 out of 22088 genes, 3.9% | 8.26E-18 | 50 out of 242 genes, 20.7% | 869 out of 22088 genes, 3.9% | 8.09E-20 |
| **system development** | 37 out of 206 genes, 18.0% | 869 out of 22088 genes, 3.9% | 2.65E-12 | 37 out of 197 genes, 18.8% | 869 out of 22088 genes, 3.9% | 5.87E-13 | 45 out of 220 genes, 20.5% | 869 out of 22088 genes, 3.9% | 1.51E-17 | 45 out of 255 genes, 17.6% | 869 out of 22088 genes, 3.9% | 6.74E-15 | 49 out of 257 genes, 19.1% | 869 out of 22088 genes, 3.9% | 8.26E-18 | 50 out of 242 genes, 20.7% | 869 out of 22088 genes, 3.9% | 8.09E-20 |
| **regulation of biological quality** | 21 out of 206 genes, 10.2% | 777 out of 22088 genes, 3.5% | 0.00428 | 21 out of 197 genes, 10.7% | 777 out of 22088 genes, 3.5% | 0.00209 | 21 out of 220 genes, 9.5% | 777 out of 22088 genes, 3.5% | 0.0113 | 23 out of 255 genes, 9.0% | 777 out of 22088 genes, 3.5% | 0.01273 | 23 out of 257 genes, 8.9% | 777 out of 22088 genes, 3.5% | 0.01455 | 22 out of 242 genes, 9.1% | 777 out of 22088 genes, 3.5% | 0.01651 |
| **cellular developmental process** | 22 out of 206 genes, 10.7% | 646 out of 22088 genes, 2.9% | 5.93E-05 | 22 out of 197 genes, 11.2% | 646 out of 22088 genes, 2.9% | 2.62E-05 | 27 out of 220 genes, 12.3% | 646 out of 22088 genes, 2.9% | 1.09E-07 | 31 out of 255 genes, 12.2% | 646 out of 22088 genes, 2.9% | 7.27E-09 | 29 out of 257 genes, 11.3% | 646 out of 22088 genes, 2.9% | 1.95E-07 | 33 out of 242 genes, 13.6% | 646 out of 22088 genes, 2.9% | 6.36E-11 |
| **post-embryonic development** | 23 out of 206 genes, 11.2% | 669 out of 22088 genes, 3.0% | 2.58E-05 | 23 out of 197 genes, 11.7% | 669 out of 22088 genes, 3.0% | 1.09E-05 | 23 out of 220 genes, 10.5% | 669 out of 22088 genes, 3.0% | 8.42E-05 | 25 out of 255 genes, 9.8% | 669 out of 22088 genes, 3.0% | 9.36E-05 | 25 out of 257 genes, 9.7% | 669 out of 22088 genes, 3.0% | 0.0001 | 23 out of 242 genes, 9.5% | 669 out of 22088 genes, 3.0% | 0.00047 |
| **tissue development** | 23 out of 206 genes, 11.2% | 348 out of 22088 genes, 1.6% | 7.15E-11 | 23 out of 197 genes, 11.7% | 348 out of 22088 genes, 1.6% | 2.67E-11 | 23 out of 220 genes, 10.5% | 348 out of 22088 genes, 1.6% | 2.83E-10 | 27 out of 255 genes, 10.6% | 348 out of 22088 genes, 1.6% | 2.14E-12 | 27 out of 257 genes, 10.5% | 348 out of 22088 genes, 1.6% | 2.64E-12 | 28 out of 242 genes, 11.6% | 348 out of 22088 genes, 1.6% | 6.59E-14 |
| **cellular response to hormone stimulus** | 24 out of 206 genes, 11.7% | 473 out of 22088 genes, 2.1% | 5.87E-09 | 24 out of 197 genes, 12.2% | 473 out of 22088 genes, 2.1% | 2.19E-09 | 24 out of 220 genes, 10.9% | 473 out of 22088 genes, 2.1% | 2.32E-08 | 24 out of 255 genes, 9.4% | 473 out of 22088 genes, 2.1% | 5.13E-07 | 24 out of 257 genes, 9.3% | 473 out of 22088 genes, 2.1% | 6.07E-07 | 24 out of 242 genes, 9.9% | 473 out of 22088 genes, 2.1% | 1.77E-07 |
| **hormone-mediated signaling** | 24 out of 206 genes, 11.7% | 473 out of 22088 genes, 2.1% | 5.87E-09 | 24 out of 197 genes, 12.2% | 473 out of 22088 genes, 2.1% | 2.19E-09 | 24 out of 220 genes, 10.9% | 473 out of 22088 genes, 2.1% | 2.32E-08 | 24 out of 255 genes, 9.4% | 473 out of 22088 genes, 2.1% | 5.13E-07 | 24 out of 257 genes, 9.3% | 473 out of 22088 genes, 2.1% | 6.07E-07 | 24 out of 242 genes, 9.9% | 473 out of 22088 genes, 2.1% | 1.77E-07 |
| **intracellular signaling cascade** | 24 out of 206 genes, 11.7% | 676 out of 22088 genes, 3.1% | 7.20E-06 | 24 out of 197 genes, 12.2% | 676 out of 22088 genes, 3.1% | 2.91E-06 | 24 out of 220 genes, 10.9% | 676 out of 22088 genes, 3.1% | 2.51E-05 | 24 out of 255 genes, 9.4% | 676 out of 22088 genes, 3.1% | 0.0004 | 24 out of 257 genes, 9.3% | 676 out of 22088 genes, 3.1% | 0.00047 | 24 out of 242 genes, 9.9% | 676 out of 22088 genes, 3.1% | 0.00015 |
| **cellular response to stimulus** | 28 out of 206 genes, 13.6% | 1009 out of 22088 genes, 4.6% | 8.11E-05 | 28 out of 197 genes, 14.2% | 1009 out of 22088 genes, 4.6% | 3.06E-05 | 30 out of 220 genes, 13.6% | 1009 out of 22088 genes, 4.6% | 2.78E-05 | 31 out of 255 genes, 12.2% | 1009 out of 22088 genes, 4.6% | 0.00024 | 31 out of 257 genes, 12.1% | 1009 out of 22088 genes, 4.6% | 0.00028 | 30 out of 242 genes, 12.4% | 1009 out of 22088 genes, 4.6% | 0.00023 |
| **Anatomical structure morphogenesis** | 29 out of 206 genes, 14.1% | 780 out of 22088 genes, 3.5% | 7.32E-08 | 29 out of 197 genes, 14.7% | 780 out of 22088 genes, 3.5% | 2.39E-08 | 36 out of 220 genes, 16.4% | 780 out of 22088 genes, 3.5% | 4.50E-12 | 42 out of 255 genes, 16.5% | 780 out of 22088 genes, 3.5% | 2.29E-14 |  |  |  | 45 out of 242 genes, 18.6% | 780 out of 22088 genes, 3.5% | 1.23E-17 |
| **meristem maintenance** | 11 out of 206 genes, 5.3% | 58 out of 22088 genes, 0.3% | 1.78E-09 | 11 out of 197 genes, 5.6% | 58 out of 22088 genes, 0.3% | 1.07E-09 | 11 out of 220 genes, 5.0% | 58 out of 22088 genes, 0.3% | 3.57E-09 | 14 out of 255 genes, 5.5% | 58 out of 22088 genes, 0.3% | 1.13E-12 | 14 out of 257 genes, 5.4% | 58 out of 22088 genes, 0.3% | 1.28E-12 | 14 out of 242 genes, 5.8% | 58 out of 22088 genes, 0.3% | 5.46E-13 |
| **regionalization** | 11 out of 206 genes, 5.3% | 161 out of 22088 genes, 0.7% | 0.00011 | 11 out of 197 genes, 5.6% | 161 out of 22088 genes, 0.7% | 7.05E-05 | 11 out of 220 genes, 5.0% | 161 out of 22088 genes, 0.7% | 0.00021 | 12 out of 255 genes, 4.7% | 161 out of 22088 genes, 0.7% | 0.00013 | 12 out of 257 genes, 4.7% | 161 out of 22088 genes, 0.7% | 0.00014 | 12 out of 242 genes, 5.0% | 161 out of 22088 genes, 0.7% | 7.54E-05 |
| **Regulation of Cell Differentiation** | 10 out of 206 genes, 4.9% | 44 out of 22088 genes, 0.2% | 2.43E-09 | 10 out of 197 genes, 5.1% | 44 out of 22088 genes, 0.2% | 1.52E-09 | 10 out of 220 genes, 4.5% | 44 out of 22088 genes, 0.2% | 4.59E-09 | 11 out of 255 genes, 4.3% | 44 out of 22088 genes, 0.2% | 7.22E-10 | 11 out of 257 genes, 4.3% | 44 out of 22088 genes, 0.2% | 7.95E-10 | 10 out of 242 genes, 4.1% | 44 out of 22088 genes, 0.2% | 1.24E-08 |
| **negative regulation of cellular process** | 10 out of 206 genes, 4.9% | 66 out of 22088 genes, 0.3% | 1.73E-07 | 10 out of 197 genes, 5.1% | 66 out of 22088 genes, 0.3% | 1.09E-07 | 10 out of 220 genes, 4.5% | 66 out of 22088 genes, 0.3% | 3.22E-07 | 11 out of 255 genes, 4.3% | 66 out of 22088 genes, 0.3% | 8.09E-08 | 11 out of 257 genes, 4.3% | 66 out of 22088 genes, 0.3% | 8.89E-08 | 10 out of 242 genes, 4.1% | 66 out of 22088 genes, 0.3% | 8.57E-07 |
| **cell morphogenesis** | 12 out of 206 genes, 5.8% | 363 out of 22088 genes, 1.6% | 0.0533 | 12 out of 197 genes, 6.1% | 363 out of 22088 genes, 1.6% | 0.03416 | 13 out of 220 genes, 5.9% | 363 out of 22088 genes, 1.6% | 0.0244 | 18 out of 255 genes, 7.1% | 363 out of 22088 genes, 1.6% | 8.66E-05 | 14 out of 257 genes, 5.4% | 363 out of 22088 genes, 1.6% | 0.03348 | 18 out of 242 genes, 7.4% | 363 out of 22088 genes, 1.6% | 3.95E-05 |
| **pattern specification process** | 12 out of 206 genes, 5.8% | 176 out of 22088 genes, 0.8% | 3.35E-05 | 12 out of 197 genes, 6.1% | 176 out of 22088 genes, 0.8% | 2.00E-05 | 12 out of 220 genes, 5.5% | 176 out of 22088 genes, 0.8% | 6.73E-05 | 13 out of 255 genes, 5.1% | 176 out of 22088 genes, 0.8% | 4.80E-05 | 13 out of 257 genes, 5.1% | 176 out of 22088 genes, 0.8% | 5.32E-05 | 13 out of 242 genes, 5.4% | 176 out of 22088 genes, 0.8% | 2.60E-05 |
| **flower development** | 13 out of 206 genes, 6.3% | 261 out of 22088 genes, 1.2% | 0.00036 | 13 out of 197 genes, 6.6% | 261 out of 22088 genes, 1.2% | 0.00021 | 13 out of 220 genes, 5.9% | 261 out of 22088 genes, 1.2% | 0.00074 | 13 out of 255 genes, 5.1% | 261 out of 22088 genes, 1.2% | 0.00394 | 13 out of 257 genes, 5.1% | 261 out of 22088 genes, 1.2% | 0.00433 | 13 out of 242 genes, 5.4% | 261 out of 22088 genes, 1.2% | 0.00224 |
| **negative regulation of biological process** | 13 out of 206 genes, 6.3% | 161 out of 22088 genes, 0.7% | 1.27E-06 | 13 out of 197 genes, 6.6% | 161 out of 22088 genes, 0.7% | 7.21E-07 | 13 out of 220 genes, 5.9% | 161 out of 22088 genes, 0.7% | 2.76E-06 | 14 out of 255 genes, 5.5% | 161 out of 22088 genes, 0.7% | 1.96E-06 | 14 out of 257 genes, 5.4% | 161 out of 22088 genes, 0.7% | 2.19E-06 | 13 out of 242 genes, 5.4% | 161 out of 22088 genes, 0.7% | 9.07E-06 |
| **homeostatic process** | 15 out of 206 genes, 7.3% | 402 out of 22088 genes, 1.8% | 0.00196 | 15 out of 197 genes, 7.6% | 402 out of 22088 genes, 1.8% | 0.0011 | 15 out of 220 genes, 6.8% | 402 out of 22088 genes, 1.8% | 0.00424 | 15 out of 255 genes, 5.9% | 402 out of 22088 genes, 1.8% | 0.02482 | 15 out of 257 genes, 5.8% | 402 out of 22088 genes, 1.8% | 0.02742 | 15 out of 242 genes, 6.2% | 402 out of 22088 genes, 1.8% | 0.01365 |
| **reproductive structure development** | 15 out of 206 genes, 7.3% | 361 out of 22088 genes, 1.6% | 0.00052 | 15 out of 197 genes, 7.6% | 361 out of 22088 genes, 1.6% | 0.00029 | 15 out of 220 genes, 6.8% | 361 out of 22088 genes, 1.6% | 0.00117 | 15 out of 255 genes, 5.9% | 361 out of 22088 genes, 1.6% | 0.00729 | 15 out of 257 genes, 5.8% | 361 out of 22088 genes, 1.6% | 0.00808 | 15 out of 242 genes, 6.2% | 361 out of 22088 genes, 1.6% | 0.00392 |
| **meristem development** | 19 out of 206 genes, 9.2% | 165 out of 22088 genes, 0.7% | 4.29E-13 | 19 out of 197 genes, 9.6% | 165 out of 22088 genes, 0.7% | 1.82E-13 | 19 out of 220 genes, 8.6% | 165 out of 22088 genes, 0.7% | 1.42E-12 | 22 out of 255 genes, 8.6% | 165 out of 22088 genes, 0.7% | 9.24E-15 | 22 out of 257 genes, 8.6% | 165 out of 22088 genes, 0.7% | 1.10E-14 | 22 out of 242 genes, 9.1% | 165 out of 22088 genes, 0.7% | 3.00E-15 |
| **determination of symmetry** | 6 out of 206 genes, 2.9% | 7 out of 22088 genes, 0.0% | 1.37E-09 | 6 out of 197 genes, 3.0% | 7 out of 22088 genes, 0.0% | 1.02E-09 | 6 out of 220 genes, 2.7% | 7 out of 22088 genes, 0.0% | 2.01E-09 | 6 out of 255 genes, 2.4% | 7 out of 22088 genes, 0.0% | 5.21E-09 | 6 out of 257 genes, 2.3% | 7 out of 22088 genes, 0.0% | 5.53E-09 | 6 out of 242 genes, 2.5% | 7 out of 22088 genes, 0.0% | 3.78E-09 |
| **Axis specification** | 5 out of 206 genes, 2.4% | 34 out of 22088 genes, 0.2% | 0.00486 | 5 out of 197 genes, 2.5% | 34 out of 22088 genes, 0.2% | 0.00382 | 5 out of 220 genes, 2.3% | 34 out of 22088 genes, 0.2% | 0.00654 | 5 out of 255 genes, 2.0% | 34 out of 22088 genes, 0.2% | 0.01406 | 5 out of 257 genes, 1.9% | 34 out of 22088 genes, 0.2% | 0.01476 | 5 out of 242 genes, 2.1% | 34 out of 22088 genes, 0.2% | 0.01092 |
| **cellular component assembly involved in morphogenesis** | 5 out of 206 genes, 2.4% | 37 out of 22088 genes, 0.2% | 0.00744 | 5 out of 197 genes, 2.5% | 37 out of 22088 genes, 0.2% | 0.00585 | 5 out of 220 genes, 2.3% | 37 out of 22088 genes, 0.2% | 0.01001 | 5 out of 255 genes, 2.0% | 37 out of 22088 genes, 0.2% | 0.02141 | 5 out of 257 genes, 1.9% | 37 out of 22088 genes, 0.2% | 0.02248 | 5 out of 242 genes, 2.1% | 37 out of 22088 genes, 0.2% | 0.01666 |
| **phytosteroid biosynthetic process** | 5 out of 206 genes, 2.4% | 25 out of 22088 genes, 0.1% | 0.00099 | 5 out of 197 genes, 2.5% | 25 out of 22088 genes, 0.1% | 0.00077 | 5 out of 220 genes, 2.3% | 25 out of 22088 genes, 0.1% | 0.00134 |  |  |  | 5 out of 257 genes, 1.9% | 25 out of 22088 genes, 0.1% | 0.00307 | 5 out of 242 genes, 2.1% | 25 out of 22088 genes, 0.1% | 0.00226 |
| **phytosteroid metabolic process** | 5 out of 206 genes, 2.4% | 39 out of 22088 genes, 0.2% | 0.00968 | 5 out of 197 genes, 2.5% | 39 out of 22088 genes, 0.2% | 0.00762 | 5 out of 220 genes, 2.3% | 39 out of 22088 genes, 0.2% | 0.01301 | 5 out of 255 genes, 2.0% | 39 out of 22088 genes, 0.2% | 0.02776 | 5 out of 257 genes, 1.9% | 39 out of 22088 genes, 0.2% | 0.02914 | 5 out of 242 genes, 2.1% | 39 out of 22088 genes, 0.2% | 0.02161 |
| **pollen wall assembly** | 5 out of 206 genes, 2.4% | 37 out of 22088 genes, 0.2% | 0.00744 | 5 out of 197 genes, 2.5% | 37 out of 22088 genes, 0.2% | 0.00585 | 5 out of 220 genes, 2.3% | 37 out of 22088 genes, 0.2% | 0.01001 | 5 out of 255 genes, 2.0% | 37 out of 22088 genes, 0.2% | 0.02141 | 5 out of 257 genes, 1.9% | 37 out of 22088 genes, 0.2% | 0.02248 | 5 out of 242 genes, 2.1% | 37 out of 22088 genes, 0.2% | 0.01666 |
| **steroid biosynthetic process** | 5 out of 206 genes, 2.4% | 45 out of 22088 genes, 0.2% | 0.01964 | 5 out of 197 genes, 2.5% | 45 out of 22088 genes, 0.2% | 0.01549 | 5 out of 220 genes, 2.3% | 45 out of 22088 genes, 0.2% | 0.0263 | 5 out of 255 genes, 2.0% | 45 out of 22088 genes, 0.2% | 0.05568 | 5 out of 257 genes, 1.9% | 45 out of 22088 genes, 0.2% | 0.05842 | 5 out of 242 genes, 2.1% | 45 out of 22088 genes, 0.2% | 0.04348 |
| **Adaxial/abaxial axis specification** | 4 out of 206 genes, 1.9% | 25 out of 22088 genes, 0.1% | 0.02582 | 4 out of 197 genes, 2.0% | 25 out of 22088 genes, 0.1% | 0.02118 | 4 out of 220 genes, 1.8% | 25 out of 22088 genes, 0.1% | 0.03268 |  |  |  |  |  |  | 4 out of 242 genes, 1.7% | 25 out of 22088 genes, 0.1% | 0.04984 |
| **Adaxial/abaxial pattern formation** | 4 out of 206 genes, 1.9% | 27 out of 22088 genes, 0.1% | 0.03531 | 4 out of 197 genes, 2.0% | 27 out of 22088 genes, 0.1% | 0.02898 | 4 out of 220 genes, 1.8% | 27 out of 22088 genes, 0.1% | 0.04464 |  |  |  |  |  |  |  |  |  |
| **unannotated** | 4 out of 206 genes, 1.9% | 254 out of 22088 genes, 1.1% | 1 | 2 out of 197 genes, 1.0% | 254 out of 22088 genes, 1.1% | 1 | 2 out of 220 genes, 0.9% | 254 out of 22088 genes, 1.1% | 1 | 3 out of 255 genes, 1.2% | 254 out of 22088 genes, 1.1% | 1 | 3 out of 257 genes, 1.2% | 254 out of 22088 genes, 1.1% | 1 | 2 out of 242 genes, 0.8% | 254 out of 22088 genes, 1.1% | 1 |
| **Anatomical structure formation involved in morphogenesis** | 7 out of 206 genes, 3.4% | 106 out of 22088 genes, 0.5% | 0.02005 | 7 out of 197 genes, 3.6% | 106 out of 22088 genes, 0.5% | 0.01474 | 7 out of 220 genes, 3.2% | 106 out of 22088 genes, 0.5% | 0.02972 |  |  |  | 8 out of 257 genes, 3.1% | 25 out of 22088 genes, 0.1% | 9.36E-08 | 7 out of 242 genes, 2.9% | 106 out of 22088 genes, 0.5% | 0.05665 |
| **cell wall biogenesis** | 7 out of 206 genes, 3.4% | 118 out of 22088 genes, 0.5% | 0.03949 | 7 out of 197 genes, 3.6% | 118 out of 22088 genes, 0.5% | 0.02916 | 7 out of 220 genes, 3.2% | 118 out of 22088 genes, 0.5% | 0.05814 |  |  |  |  |  |  | 8 out of 242 genes, 3.3% | 118 out of 22088 genes, 0.5% | 0.01612 |
| **floral organ development** | 7 out of 206 genes, 3.4% | 75 out of 22088 genes, 0.3% | 0.00208 | 7 out of 197 genes, 3.6% | 75 out of 22088 genes, 0.3% | 0.00151 | 7 out of 220 genes, 3.2% | 75 out of 22088 genes, 0.3% | 0.00314 | 7 out of 255 genes, 2.7% | 75 out of 22088 genes, 0.3% | 0.00862 | 7 out of 257 genes, 2.7% | 75 out of 22088 genes, 0.3% | 0.00917 | 7 out of 242 genes, 2.9% | 75 out of 22088 genes, 0.3% | 0.00614 |
| **plant-type cell wall biogenesis** | 7 out of 206 genes, 3.4% | 67 out of 22088 genes, 0.3% | 0.00097 | 7 out of 197 genes, 3.6% | 67 out of 22088 genes, 0.3% | 0.0007 | 7 out of 220 genes, 3.2% | 67 out of 22088 genes, 0.3% | 0.00147 | 7 out of 255 genes, 2.7% | 67 out of 22088 genes, 0.3% | 0.00408 | 7 out of 257 genes, 2.7% | 67 out of 22088 genes, 0.3% | 0.00435 | 7 out of 242 genes, 2.9% | 67 out of 22088 genes, 0.3% | 0.0029 |
| **post-embryonic organ development** | 7 out of 206 genes, 3.4% | 75 out of 22088 genes, 0.3% | 0.00208 | 7 out of 197 genes, 3.6% | 75 out of 22088 genes, 0.3% | 0.00151 | 7 out of 220 genes, 3.2% | 75 out of 22088 genes, 0.3% | 0.00314 | 7 out of 255 genes, 2.7% | 75 out of 22088 genes, 0.3% | 0.00862 | 7 out of 257 genes, 2.7% | 75 out of 22088 genes, 0.3% | 0.00917 | 7 out of 242 genes, 2.9% | 75 out of 22088 genes, 0.3% | 0.00614 |
| **Anatomical structure homeostasis** | 8 out of 206 genes, 3.9% | 25 out of 22088 genes, 0.1% | 1.52E-08 | 8 out of 197 genes, 4.1% | 25 out of 22088 genes, 0.1% | 1.04E-08 | 8 out of 220 genes, 3.6% | 25 out of 22088 genes, 0.1% | 2.53E-08 | 8 out of 255 genes, 3.1% | 25 out of 22088 genes, 0.1% | 8.69E-08 | 40 out of 257 genes, 15.6% | 780 out of 22088 genes, 3.5% | 9.40E-13 | 8 out of 242 genes, 3.3% | 25 out of 22088 genes, 0.1% | 5.72E-08 |
| **maintenance of meristem identity** | 8 out of 206 genes, 3.9% | 20 out of 22088 genes, 0.1% | 1.85E-09 | 8 out of 197 genes, 4.1% | 20 out of 22088 genes, 0.1% | 1.26E-09 | 8 out of 220 genes, 3.6% | 20 out of 22088 genes, 0.1% | 3.08E-09 | 8 out of 255 genes, 3.1% | 20 out of 22088 genes, 0.1% | 1.06E-08 | 8 out of 257 genes, 3.1% | 20 out of 22088 genes, 0.1% | 1.14E-08 | 8 out of 242 genes, 3.3% | 20 out of 22088 genes, 0.1% | 6.98E-09 |
| **negative regulation of cell differentiation** | 8 out of 206 genes, 3.9% | 25 out of 22088 genes, 0.1% | 1.52E-08 | 8 out of 197 genes, 4.1% | 25 out of 22088 genes, 0.1% | 1.04E-08 | 8 out of 220 genes, 3.6% | 25 out of 22088 genes, 0.1% | 2.53E-08 | 9 out of 255 genes, 3.5% | 25 out of 22088 genes, 0.1% | 1.85E-09 | 9 out of 257 genes, 3.5% | 25 out of 22088 genes, 0.1% | 2.01E-09 | 8 out of 242 genes, 3.3% | 25 out of 22088 genes, 0.1% | 5.72E-08 |
| **negative regulation of developmental process** | 8 out of 206 genes, 3.9% | 40 out of 22088 genes, 0.2% | 9.63E-07 | 8 out of 197 genes, 4.1% | 40 out of 22088 genes, 0.2% | 6.60E-07 | 8 out of 220 genes, 3.6% | 40 out of 22088 genes, 0.2% | 1.58E-06 | 9 out of 255 genes, 3.5% | 40 out of 22088 genes, 0.2% | 2.13E-07 | 9 out of 257 genes, 3.5% | 40 out of 22088 genes, 0.2% | 2.31E-07 | 8 out of 242 genes, 3.3% | 40 out of 22088 genes, 0.2% | 3.53E-06 |
| **regulation of cell differentiation** | 8 out of 206 genes, 3.9% | 25 out of 22088 genes, 0.1% | 1.52E-08 | 8 out of 197 genes, 4.1% | 25 out of 22088 genes, 0.1% | 1.04E-08 | 8 out of 220 genes, 3.6% | 25 out of 22088 genes, 0.1% | 2.53E-08 | 9 out of 255 genes, 3.5% | 25 out of 22088 genes, 0.1% | 1.85E-09 | 9 out of 257 genes, 3.5% | 25 out of 22088 genes, 0.1% | 2.01E-09 | 8 out of 242 genes, 3.3% | 25 out of 22088 genes, 0.1% | 5.72E-08 |
| **root development** | 8 out of 206 genes, 3.9% | 156 out of 22088 genes, 0.7% | 0.03609 | 8 out of 197 genes, 4.1% | 156 out of 22088 genes, 0.7% | 0.0258 | 10 out of 220 genes, 4.5% | 156 out of 22088 genes, 0.7% | 0.00123 | 10 out of 255 genes, 3.9% | 156 out of 22088 genes, 0.7% | 0.00476 | 10 out of 257 genes, 3.9% | 156 out of 22088 genes, 0.7% | 0.00515 | 10 out of 242 genes, 4.1% | 156 out of 22088 genes, 0.7% | 0.00301 |
| **root system development** | 8 out of 206 genes, 3.9% | 156 out of 22088 genes, 0.7% | 0.03609 | 8 out of 197 genes, 4.1% | 156 out of 22088 genes, 0.7% | 0.0258 | 10 out of 220 genes, 4.5% | 156 out of 22088 genes, 0.7% | 0.00123 | 10 out of 255 genes, 3.9% | 156 out of 22088 genes, 0.7% | 0.00476 | 10 out of 257 genes, 3.9% | 156 out of 22088 genes, 0.7% | 0.00515 | 10 out of 242 genes, 4.1% | 156 out of 22088 genes, 0.7% | 0.00301 |
| **stem cell development** | 8 out of 206 genes, 3.9% | 23 out of 22088 genes, 0.1% | 7.03E-09 | 8 out of 197 genes, 4.1% | 23 out of 22088 genes, 0.1% | 4.79E-09 | 8 out of 220 genes, 3.6% | 23 out of 22088 genes, 0.1% | 1.16E-08 | 8 out of 255 genes, 3.1% | 23 out of 22088 genes, 0.1% | 4.02E-08 | 8 out of 257 genes, 3.1% | 23 out of 22088 genes, 0.1% | 4.33E-08 | 8 out of 242 genes, 3.3% | 23 out of 22088 genes, 0.1% | 2.64E-08 |
| **stem cell differentiation** | 8 out of 206 genes, 3.9% | 23 out of 22088 genes, 0.1% | 7.03E-09 | 8 out of 197 genes, 4.1% | 23 out of 22088 genes, 0.1% | 4.79E-09 | 8 out of 220 genes, 3.6% | 23 out of 22088 genes, 0.1% | 1.16E-08 | 8 out of 255 genes, 3.1% | 23 out of 22088 genes, 0.1% | 4.02E-08 | 8 out of 257 genes, 3.1% | 23 out of 22088 genes, 0.1% | 4.33E-08 | 8 out of 242 genes, 3.3% | 23 out of 22088 genes, 0.1% | 2.64E-08 |
| **stem cell maintenance** | 8 out of 206 genes, 3.9% | 23 out of 22088 genes, 0.1% | 7.03E-09 | 8 out of 197 genes, 4.1% | 23 out of 22088 genes, 0.1% | 4.79E-09 | 8 out of 220 genes, 3.6% | 23 out of 22088 genes, 0.1% | 1.16E-08 | 8 out of 255 genes, 3.1% | 23 out of 22088 genes, 0.1% | 4.02E-08 | 8 out of 257 genes, 3.1% | 23 out of 22088 genes, 0.1% | 4.33E-08 | 8 out of 242 genes, 3.3% | 23 out of 22088 genes, 0.1% | 2.64E-08 |
| **Regulation of developmental process** | 9 out of 206 genes, 4.4% | 89 out of 22088 genes, 0.4% | 4.84E-05 | 9 out of 197 genes, 4.6% | 89 out of 22088 genes, 0.4% | 3.22E-05 | 13 out of 220 genes, 5.9% | 89 out of 22088 genes, 0.4% | 1.52E-09 | 12 out of 255 genes, 4.7% | 89 out of 22088 genes, 0.4% | 1.57E-07 | 14 out of 257 genes, 5.4% | 89 out of 22088 genes, 0.4% | 7.00E-10 | 13 out of 242 genes, 5.4% | 89 out of 22088 genes, 0.4% | 5.36E-09 |
| **Anatomical structure arrangement** | 9 out of 206 genes, 4.4% | 49 out of 22088 genes, 0.2% | 2.15E-07 | 9 out of 197 genes, 4.6% | 49 out of 22088 genes, 0.2% | 1.41E-07 | 9 out of 220 genes, 4.1% | 49 out of 22088 genes, 0.2% | 3.78E-07 | 12 out of 255 genes, 4.7% | 49 out of 22088 genes, 0.2% | 9.21E-11 | 12 out of 257 genes, 4.7% | 49 out of 22088 genes, 0.2% | 1.02E-10 | 12 out of 242 genes, 5.0% | 49 out of 22088 genes, 0.2% | 4.93E-11 |
| **meristem structural organization** | 9 out of 206 genes, 4.4% | 49 out of 22088 genes, 0.2% | 2.15E-07 | 9 out of 197 genes, 4.6% | 49 out of 22088 genes, 0.2% | 1.41E-07 | 9 out of 220 genes, 4.1% | 49 out of 22088 genes, 0.2% | 3.78E-07 | 12 out of 255 genes, 4.7% | 49 out of 22088 genes, 0.2% | 9.21E-11 | 12 out of 257 genes, 4.7% | 49 out of 22088 genes, 0.2% | 1.02E-10 | 12 out of 242 genes, 5.0% | 49 out of 22088 genes, 0.2% | 4.93E-11 |
| **pollen development** | 9 out of 206 genes, 4.4% | 85 out of 22088 genes, 0.4% | 3.24E-05 | 9 out of 197 genes, 4.6% | 85 out of 22088 genes, 0.4% | 2.15E-05 | 9 out of 220 genes, 4.1% | 85 out of 22088 genes, 0.4% | 5.56E-05 | 9 out of 255 genes, 3.5% | 85 out of 22088 genes, 0.4% | 0.0002 | 9 out of 257 genes, 3.5% | 85 out of 22088 genes, 0.4% | 0.00022 | 9 out of 242 genes, 3.7% | 85 out of 22088 genes, 0.4% | 0.00013 |
| **regulation of developmental process** | 9 out of 206 genes, 4.4% | 88 out of 22088 genes, 0.4% | 4.38E-05 | 9 out of 197 genes, 4.6% | 88 out of 22088 genes, 0.4% | 2.92E-05 | 13 out of 220 genes, 5.9% | 88 out of 22088 genes, 0.4% | 1.31E-09 |  |  |  | 14 out of 257 genes, 5.4% | 88 out of 22088 genes, 0.4% | 5.96E-10 | 13 out of 242 genes, 5.4% | 88 out of 22088 genes, 0.4% | 4.62E-09 |
| **cellular response to nutrient** | 2 out of 206 genes, 1.0% | 2 out of 22088 genes, 0.0% | 0.02804 | 2 out of 197 genes, 1.0% | 2 out of 22088 genes, 0.0% | 0.02501 | 2 out of 220 genes, 0.9% | 2 out of 22088 genes, 0.0% | 0.0314 | 2 out of 255 genes, 0.8% | 2 out of 22088 genes, 0.0% | 0.04474 | 2 out of 257 genes, 0.8% | 2 out of 22088 genes, 0.0% | 0.04598 | 2 out of 242 genes, 0.8% | 2 out of 22088 genes, 0.0% | 0.04016 |
| **dorsal/ventral pattern formation** | 2 out of 206 genes, 1.0% | 2 out of 22088 genes, 0.0% | 0.02804 | 2 out of 197 genes, 1.0% | 2 out of 22088 genes, 0.0% | 0.02501 | 2 out of 220 genes, 0.9% | 2 out of 22088 genes, 0.0% | 0.0314 | 2 out of 255 genes, 0.8% | 2 out of 22088 genes, 0.0% | 0.04474 | 2 out of 257 genes, 0.8% | 2 out of 22088 genes, 0.0% | 0.04598 | 2 out of 242 genes, 0.8% | 2 out of 22088 genes, 0.0% | 0.04016 |
| **negative regulation of transcription by carbon catabolites** | 2 out of 206 genes, 1.0% | 2 out of 22088 genes, 0.0% | 0.02804 | 2 out of 197 genes, 1.0% | 2 out of 22088 genes, 0.0% | 0.02501 | 2 out of 220 genes, 0.9% | 2 out of 22088 genes, 0.0% | 0.0314 | 2 out of 255 genes, 0.8% | 2 out of 22088 genes, 0.0% | 0.04474 | 2 out of 257 genes, 0.8% | 2 out of 22088 genes, 0.0% | 0.04598 | 2 out of 242 genes, 0.8% | 2 out of 22088 genes, 0.0% | 0.04016 |
| **regulation of transcription by carbon catabolites** | 2 out of 206 genes, 1.0% | 2 out of 22088 genes, 0.0% | 0.02804 | 2 out of 197 genes, 1.0% | 2 out of 22088 genes, 0.0% | 0.02501 | 2 out of 220 genes, 0.9% | 2 out of 22088 genes, 0.0% | 0.0314 | 2 out of 255 genes, 0.8% | 2 out of 22088 genes, 0.0% | 0.04474 | 2 out of 257 genes, 0.8% | 2 out of 22088 genes, 0.0% | 0.04598 | 2 out of 242 genes, 0.8% | 2 out of 22088 genes, 0.0% | 0.04016 |
| **Regulation of macromolecule metabolic process** |  |  |  | 12 out of 197 genes, 6.1% | 381 out of 22088 genes, 1.7% | 0.0536 |  |  |  |  |  |  |  |  |  |  |  |  |
| **biosynthetic process** |  |  |  |  |  |  | 61 out of 220 genes, 27.7% | 3786 out of 22088 genes, 17.1% | 0.01809 | 77 out of 255 genes, 30.2% | 3786 out of 22088 genes, 17.1% | 5.95E-05 | 77 out of 257 genes, 30.0% | 3786 out of 22088 genes, 17.1% | 8.55E-05 | 66 out of 242 genes, 27.3% | 3786 out of 22088 genes, 17.1% | 0.01698 |
| **cell development** |  |  |  | 11 out of 197 genes, 5.6% | 326 out of 22088 genes, 1.5% | 0.05541 |  |  |  | 16 out of 255 genes, 6.3% | 326 out of 22088 genes, 1.5% | 0.00046 |  |  |  | 16 out of 242 genes, 6.6% | 326 out of 22088 genes, 1.5% | 0.00023 |
| **cell differentiation** |  |  |  | 13 out of 197 genes, 6.6% | 444 out of 22088 genes, 2.0% | 0.05906 |  |  |  | 19 out of 255 genes, 7.5% | 444 out of 22088 genes, 2.0% | 0.00037 |  |  |  | 19 out of 242 genes, 7.9% | 444 out of 22088 genes, 2.0% | 0.00016 |
| [**cell morphogenesis involved in differentiation**](http://amigo.geneontology.org/cgi-bin/amigo/go.cgi?action=query&view=query&query=GO:0000904&search_constraint=terms) |  |  |  |  |  |  |  |  |  | 12 out of 255 genes, 4.7% | 222 out of 22088 genes, 1.0% | 0.00379 |  |  |  | 12 out of 242 genes, 5.0% | 222 out of 22088 genes, 1.0% | 0.00223 |
| **cell proliferation** |  |  |  |  |  |  | 6 out of 220 genes, 2.7% | 53 out of 22088 genes, 0.2% | 0.0045 | 6 out of 255 genes, 2.4% | 53 out of 22088 genes, 0.2% | 0.01096 | 6 out of 257 genes, 2.3% | 53 out of 22088 genes, 0.2% | 0.01158 | 6 out of 242 genes, 2.5% | 53 out of 22088 genes, 0.2% | 0.00815 |
| **cellular biosynthetic process** |  |  |  |  |  |  | 61 out of 220 genes, 27.7% | 3746 out of 22088 genes, 17.0% | 0.01317 | 77 out of 255 genes, 30.2% | 3746 out of 22088 genes, 17.0% | 3.80E-05 | 77 out of 257 genes, 30.0% | 3746 out of 22088 genes, 17.0% | 5.49E-05 | 66 out of 242 genes, 27.3% | 3746 out of 22088 genes, 17.0% | 0.01215 |
| **cellular component morphogenesis** |  |  |  |  |  |  | 13 out of 220 genes, 5.9% | 396 out of 22088 genes, 1.8% | 0.05781 | 18 out of 255 genes, 7.1% | 396 out of 22088 genes, 1.8% | 0.0003 |  |  |  | 18 out of 242 genes, 7.4% | 396 out of 22088 genes, 1.8% | 0.00014 |
| [**gene expression**](http://amigo.geneontology.org/cgi-bin/amigo/go.cgi?action=query&view=query&query=GO:0010467&search_constraint=terms) |  |  |  |  |  |  |  |  |  | 73 out of 255 genes, 28.6% | 3617 out of 22088 genes, 16.4% | 0.00019 | 73 out of 257 genes, 28.4% | 3617 out of 22088 genes, 16.4% | 0.00027 | 62 out of 242 genes, 25.6% | 3617 out of 22088 genes, 16.4% | 0.05095 |
| **macromolecule metabolic process** |  |  |  |  |  |  | 119 out of 220 genes, 54.1% | 9008 out of 22088 genes, 40.8% | 0.01353 | 142 out of 255 genes, 55.7% | 9008 out of 22088 genes, 40.8% | 0.00033 | 142 out of 257 genes, 55.3% | 9008 out of 22088 genes, 40.8% | 0.0006 |  |  |  |
| **metabolic process** |  |  |  |  |  |  | 183 out of 220 genes, 83.2% | 15829 out of 22088 genes, 71.7% | 0.01441 | 211 out of 255 genes, 82.7% | 15829 out of 22088 genes, 71.7% | 0.00833 | 213 out of 257 genes, 82.9% | 15829 out of 22088 genes, 71.7% | 0.00622 |  |  |  |
| [**nitrogen compound metabolic process**](http://amigo.geneontology.org/cgi-bin/amigo/go.cgi?action=query&view=query&query=GO:0006807&search_constraint=terms) |  |  |  |  |  |  |  |  |  | 84 out of 255 genes, 32.9% | 4402 out of 22088 genes, 19.9% | 0.0002 | 84 out of 257 genes, 32.7% | 4402 out of 22088 genes, 19.9% | 0.0003 | 73 out of 242 genes, 30.2% | 4402 out of 22088 genes, 19.9% | 0.03051 |
| **nucleobase, nucleoside, nucleotide and nucleic acid metabolic process** |  |  |  |  |  |  | 61 out of 220 genes, 27.7% | 3768 out of 22088 genes, 17.1% | 0.01569 | 80 out of 255 genes, 31.4% | 3768 out of 22088 genes, 17.1% | 4.41E-06 | 80 out of 257 genes, 31.1% | 3768 out of 22088 genes, 17.1% | 6.57E-06 | 69 out of 242 genes, 28.5% | 3768 out of 22088 genes, 17.1% | 0.00198 |
| **phyllome development** |  |  |  |  |  |  | 12 out of 220 genes, 5.5% | 164 out of 22088 genes, 0.7% | 3.11E-05 | 5 out of 255 genes, 2.0% | 25 out of 22088 genes, 0.1% | 0.00292 | 12 out of 257 genes, 4.7% | 164 out of 22088 genes, 0.7% | 0.00017 | 12 out of 242 genes, 5.0% | 164 out of 22088 genes, 0.7% | 9.22E-05 |
| **regulation of development, heterochronic** |  |  |  |  |  |  | 6 out of 220 genes, 2.7% | 33 out of 22088 genes, 0.1% | 0.00025 | 12 out of 255 genes, 4.7% | 88 out of 22088 genes, 0.4% | 1.37E-07 | 6 out of 257 genes, 2.3% | 33 out of 22088 genes, 0.1% | 0.00067 | 6 out of 242 genes, 2.5% | 33 out of 22088 genes, 0.1% | 0.00047 |
| **shoot development** |  |  |  |  |  |  | 12 out of 220 genes, 5.5% | 189 out of 22088 genes, 0.9% | 0.00014 | 33 out of 255 genes, 12.9% | 1162 out of 22088 genes, 5.3% | 0.00058 | 12 out of 257 genes, 4.7% | 189 out of 22088 genes, 0.9% | 0.00079 | 12 out of 242 genes, 5.0% | 189 out of 22088 genes, 0.9% | 0.00042 |
| **shoot system development** |  |  |  |  |  |  | 12 out of 220 genes, 5.5% | 189 out of 22088 genes, 0.9% | 0.00014 |  |  |  | 12 out of 257 genes, 4.7% | 189 out of 22088 genes, 0.9% | 0.00079 | 12 out of 242 genes, 5.0% | 189 out of 22088 genes, 0.9% | 0.00042 |
