# Supplementary material for: Genome-wide identification of alternate bearing-associated microRNAs (miRNAs) in olive (Olea europaea L.)
Source: BMC Plant Biol. 2013 Jan 15;13:10. doi: 10.1186/1471-2229-13-10 (PMC3564680; doi:10.1186/1471-2229-13-10)
Supplement: Additional file 7 — Pathway annotation of the miRNA targets. The miRNA targets were included in 130 KEGG pathways. [file 1471-2229-13-10-S7.doc]

**Additional file 6: Pathway annotation of miRNA targets.** miRNA targets were included in 130 KEGG pathways.

|  |  | **HM** | | | | **OM** | | | | **VK** | | | | **VT** | | | | **YK** | | | | **YT** | | | |
| --- | --- | --- | --- | --- | --- | --- | --- | --- | --- | --- | --- | --- | --- | --- | --- | --- | --- | --- | --- | --- | --- | --- | --- | --- | --- |
| **Pathway** | **Pathway ID** | **Sample** | | **Pvalue** | **Qvalue** | **Sample** | | **Pvalue** | **Qvalue** | **Sample** | | **Pvalue** | **Qvalue** | **Sample** | | **Pvalue** | **Qvalue** | **Sample** | | **Pvalue** | **Qvalue** | **Sample** | | **Pvalue** | **Qvalue** |
|  |  | **1 -205** | **2 -22821** |  |  | **1 -205** | **2 -22821** |  |  | **1 -205** | **2 -22821** |  |  | **1 -205** | **2 -22821** |  |  | **1 -205** | **2 -22821** |  |  | **1 -**  **205** | **2 -22821** |  |  |
| [Ascorbate and aldarate metabolism](../../olive%20tables/Additional%20file%206.xls#RANGE!gene1) | ko00053 | 33 | 171 | 1.46E-34 | 1.75E-32 | 33 | 171 | 8.73E-35 | 9.87E-33 | 33 | 171 | 4.65E-34 | 5.35E-32 | 33 | 171 | 9.92E-32 | 1.24E-29 | 33 | 171 | 5.72E-32 | 7.20E-30 | 33 | 171 | 1.38E-32 | 1.69E-30 |
| Plant hormone signal transduction | ko04075 | 39 | 1919 | 1.12E-06 | 6.71E-05 | 39 | 1919 | 7.59E-07 | 4.29E-05 | 41 | 1919 | 3.81E-07 | 2.19E-05 | 46 | 1919 | 2.74E-07 | 1.71E-05 | 46 | 1919 | 1.67E-07 | 1.05E-05 | 42 | 1919 | 2.16E-06 | 1.33E-04 |
| Brassinosteroid biosynthesis | ko00905 | 6 | 52 | 7.05E-06 | 2.82E-04 | 6 | 52 | 6.48E-06 | 2.44E-04 | 6 | 52 | 8.54E-06 | 3.27E-04 | 6 | 52 | 2.08E-05 | 8.65E-04 | 6 | 52 | 1.90E-05 | 7.96E-04 | 6 | 52 | 1.50E-05 | 6.13E-04 |
| Vibrio cholerae infection | ko05110 | 3 | 105 | 0.0689052 | 9.85E-01 | 2 | 105 | 0.238108 | 9.77E-01 | 2 | 105 | 0.255124 | 9.55E-01 | 2 | 105 | 0.31636 | 9.66E-01 | 2 | 105 | 0.309588 | 9.67E-01 | 2 | 105 | 0.292607 | 9.80E-01 |
| Metabolic pathways | ko01100 | 52 | 4805 | 0.0778905 | 9.85E-01 | 50 | 4805 | 0.114787 | 9.77E-01 | 49 | 4805 | 0.253701 | 9.55E-01 | 53 | 4805 | 0.47643 | 9.66E-01 | 53 | 4805 | 0.423853 | 9.67E-01 | 52 | 4805 | 0.354687 | 9.80E-01 |
| Selenocompound metabolism | ko00450 | 2 | 52 | 0.0795101 | 9.85E-01 | 2 | 52 | 0.077523 | 9.77E-01 | 2 | 52 | 0.084209 | 9.55E-01 | 2 | 52 | 0.109612 | 9.66E-01 | 2 | 52 | 0.106696 | 9.67E-01 | 2 | 52 | 0.099502 | 9.80E-01 |
| Carbohydrate digestion and absorption | ko04973 | 1 | 15 | 0.126629 | 9.85E-01 | 1 | 15 | 0.124889 | 9.77E-01 | 1 | 15 | 0.130676 | 9.55E-01 | 1 | 15 | 0.151216 | 9.66E-01 | 1 | 15 | 0.148956 | 9.67E-01 | 1 | 15 | 0.143283 | 9.80E-01 |
| Sulfur metabolism | ko00920 | 2 | 69 | 0.1276972 | 9.85E-01 | 2 | 69 | 0.124676 | 9.77E-01 | 2 | 69 | 0.134814 | 9.55E-01 | 2 | 69 | 0.172669 | 9.66E-01 | 2 | 69 | 0.168375 | 9.67E-01 | 2 | 69 | 0.157726 | 9.80E-01 |
| Tight junction | ko04530 | 3 | 147 | 0.1463954 | 9.85E-01 | 3 | 147 | 0.141909 | 9.77E-01 | 3 | 147 | 0.157042 | 9.55E-01 | 3 | 147 | 0.215069 | 9.66E-01 | 3 | 147 | 0.208395 | 9.67E-01 | 3 | 147 | 0.191932 | 9.80E-01 |
| Lysine degradation | ko00310 | 2 | 81 | 0.1648472 | 9.85E-01 | 2 | 81 | 0.161098 | 9.77E-01 | 2 | 81 | 0.173655 | 9.55E-01 | 2 | 81 | 0.219967 | 9.66E-01 | 2 | 81 | 0.214758 | 9.67E-01 | 2 | 81 | 0.201792 | 9.80E-01 |
| Sphingolipid metabolism | ko00600 | 2 | 85 | 0.1776222 | 9.85E-01 | 2 | 85 | 0.173636 | 9.77E-01 | 2 | 85 | 0.186979 | 9.55E-01 | 2 | 85 | 0.235986 | 9.66E-01 | 2 | 85 | 0.230489 | 9.67E-01 | 2 | 85 | 0.21679 | 9.80E-01 |
| Chagas disease (American trypanosomiasis) | ko05142 | 7 | 520 | 0.1885664 | 9.85E-01 | 7 | 520 | 0.179351 | 9.77E-01 | 8 | 520 | 0.112862 | 9.55E-01 | 8 | 520 | 0.206539 | 9.66E-01 | 8 | 520 | 0.194864 | 9.67E-01 | 8 | 520 | 0.166955 | 9.80E-01 |
| Protein export | ko03060 | 2 | 103 | 0.2365694 | 9.85E-01 | 2 | 103 | 0.231575 | 9.77E-01 | 2 | 103 | 0.248246 | 9.55E-01 | 2 | 103 | 0.308364 | 9.66E-01 | 2 | 103 | 0.301706 | 9.67E-01 | 2 | 103 | 0.285022 | 9.80E-01 |
| Nitrogen metabolism | ko00910 | 2 | 112 | 0.2664861 | 9.85E-01 | 2 | 112 | 0.261032 | 9.77E-01 | 2 | 112 | 0.279211 | 9.55E-01 | 2 | 112 | 0.344162 | 9.66E-01 | 2 | 112 | 0.337015 | 9.67E-01 | 2 | 112 | 0.319055 | 9.80E-01 |
| Plant-pathogen interaction | ko04626 | 27 | 2649 | 0.2709061 | 9.85E-01 | 27 | 2649 | 0.24552 | 9.77E-01 | 30 | 2649 | 0.146375 | 9.55E-01 | 33 | 2649 | 0.226179 | 9.66E-01 | 33 | 2649 | 0.198456 | 9.67E-01 | 31 | 2649 | 0.242452 | 9.80E-01 |
| Collecting duct acid secretion | ko04966 | 1 | 35 | 0.2709847 | 9.85E-01 |  |  |  |  |  |  |  |  |  |  |  |  |  |  |  |  |  |  |  |  |
| Starch and sucrose metabolism | ko00500 | 7 | 583 | 0.2711647 | 9.85E-01 | 7 | 583 | 0.259389 | 9.77E-01 | 7 | 583 | 0.299157 | 9.55E-01 | 8 | 583 | 0.301559 | 9.66E-01 | 8 | 583 | 0.286682 | 9.67E-01 | 7 | 583 | 0.390053 | 9.80E-01 |
| Base excision repair | ko03410 | 2 | 115 | 0.2764678 | 9.85E-01 | 2 | 115 | 0.270869 | 9.77E-01 | 3 | 115 | 0.091965 | 9.55E-01 | 4 | 115 | 0.036944 | 9.24E-01 | 4 | 115 | 0.035128 | 8.85E-01 | 4 | 115 | 0.030821 | 7.58E-01 |
| Purine metabolism | ko00230 | 4 | 305 | 0.2940316 | 9.85E-01 | 3 | 305 | 0.50821 | 9.77E-01 | 3 | 305 | 0.540693 | 9.55E-01 | 4 | 305 | 0.423691 | 9.66E-01 | 4 | 305 | 0.411722 | 9.67E-01 | 4 | 305 | 0.381602 | 9.80E-01 |
| Indole alkaloid biosynthesis | ko00901 | 1 | 41 | 0.3094669 | 9.85E-01 | 1 | 41 | 0.305698 | 9.77E-01 | 1 | 41 | 0.318184 | 9.55E-01 | 1 | 112 | 0.706767 | 9.66E-01 | 1 | 112 | 0.700875 | 9.67E-01 | 1 | 41 | 0.344885 | 9.80E-01 |
| One carbon pool by folate | ko00670 | 1 | 46 | 0.3399863 | 9.85E-01 | 1 | 46 | 0.335943 | 9.77E-01 | 1 | 46 | 0.349328 | 9.55E-01 | 1 | 190 | 0.875663 | 9.66E-01 | 1 | 46 | 0.390409 | 9.67E-01 | 1 | 46 | 0.377851 | 9.80E-01 |
| Zeatin biosynthesis | ko00908 | 2 | 137 | 0.34904 | 9.85E-01 | 2 | 137 | 0.342503 | 9.77E-01 | 2 | 137 | 0.364207 | 9.55E-01 | 2 | 137 | 0.439804 | 9.66E-01 | 2 | 137 | 0.431633 | 9.67E-01 | 2 | 137 | 0.410939 | 9.80E-01 |
| Aldosterone-regulated sodium reabsorption | ko04960 | 1 | 51 | 0.3691631 | 9.85E-01 | 1 | 51 | 0.364877 | 9.77E-01 | 1 | 51 | 0.379055 | 9.55E-01 | 1 | 51 | 0.427576 | 9.66E-01 | 1 | 51 | 0.422374 | 9.67E-01 | 1 | 51 | 0.409165 | 9.80E-01 |
| Other glycan degradation | ko00511 | 1 | 55 | 0.3915775 | 9.85E-01 | 1 | 55 | 0.387117 | 9.77E-01 | 1 | 55 | 0.401861 | 9.55E-01 | 1 | 100 | 0.665479 | 9.66E-01 | 1 | 55 | 0.446739 | 9.67E-01 | 1 | 55 | 0.433082 | 9.80E-01 |
| mRNA surveillance pathway | ko03015 | 4 | 358 | 0.4015959 | 9.85E-01 | 4 | 358 | 0.390983 | 9.77E-01 | 3 | 358 | 0.649018 | 9.55E-01 | 3 | 358 | 0.749301 | 9.66E-01 | 3 | 358 | 0.739409 | 9.67E-01 | 4 | 358 | 0.501569 | 9.80E-01 |
| Epithelial cell signaling in Helicobacter pylori infection | ko05120 | 1 | 57 | 0.4024857 | 9.85E-01 |  |  |  |  |  |  |  |  |  |  |  |  |  |  |  |  |  |  |  |  |
| Ether lipid metabolism | ko00565 | 1 | 63 | 0.4340562 | 9.85E-01 | 1 | 63 | 0.429301 | 9.77E-01 | 1 | 63 | 0.445001 | 9.55E-01 | 1 | 63 | 0.498084 | 9.66E-01 | 1 | 63 | 0.492442 | 9.67E-01 | 1 | 63 | 0.478062 | 9.80E-01 |
| NOD-like receptor signaling pathway | ko04621 | 1 | 65 | 0.4442063 | 9.85E-01 | 1 | 65 | 0.439387 | 9.77E-01 | 1 | 65 | 0.455294 | 9.55E-01 | 1 | 65 | 0.508963 | 9.66E-01 | 1 | 65 | 0.503267 | 9.67E-01 | 1 | 65 | 0.488741 | 9.80E-01 |
| Dorso-ventral axis formation | ko04320 | 1 | 67 | 0.4541753 | 9.85E-01 | 1 | 67 | 0.449296 | 9.77E-01 | 1 | 67 | 0.465396 | 9.55E-01 | 1 | 67 | 0.519608 | 9.66E-01 | 1 | 67 | 0.513863 | 9.67E-01 | 1 | 67 | 0.499201 | 9.80E-01 |
| MAPK signaling pathway - fly | ko04013 | 1 | 67 | 0.4541753 | 9.85E-01 | 1 | 67 | 0.449296 | 9.77E-01 | 1 | 67 | 0.465396 | 9.55E-01 | 1 | 67 | 0.519608 | 9.66E-01 | 1 | 67 | 0.513863 | 9.67E-01 | 1 | 67 | 0.499201 | 9.80E-01 |
| Lysosome | ko04142 | 3 | 282 | 0.4661194 | 9.85E-01 | 2 | 282 | 0.715179 | 9.77E-01 | 2 | 282 | 0.739749 | 9.55E-01 | 2 | 282 | 0.813722 | 9.66E-01 | 2 | 282 | 0.806539 | 9.67E-01 | 2 | 282 | 0.787496 | 9.80E-01 |
| Antigen processing and presentation | ko04612 | 2 | 176 | 0.4704863 | 9.85E-01 | 2 | 176 | 0.462865 | 9.77E-01 | 3 | 176 | 0.224893 | 9.55E-01 | 3 | 176 | 0.299452 | 9.66E-01 | 3 | 176 | 0.291051 | 9.67E-01 | 3 | 176 | 0.270136 | 9.80E-01 |
| Circadian rhythm - plant | ko04712 | 3 | 286 | 0.4753381 | 9.85E-01 | 3 | 286 | 0.465694 | 9.77E-01 | 4 | 286 | 0.275571 | 9.55E-01 | 4 | 286 | 0.377218 | 9.66E-01 | 4 | 286 | 0.365867 | 9.67E-01 | 4 | 286 | 0.33747 | 9.80E-01 |
| Bladder cancer | ko05219 | 1 | 73 | 0.4830271 | 9.85E-01 | 1 | 73 | 0.477989 | 9.77E-01 | 1 | 73 | 0.494597 | 9.55E-01 | 1 | 73 | 0.550183 | 9.66E-01 | 1 | 73 | 0.544318 | 9.67E-01 | 1 | 73 | 0.529322 | 9.80E-01 |
| Acute myeloid leukemia | ko05221 | 1 | 77 | 0.5014133 | 9.85E-01 | 1 | 77 | 0.496286 | 9.77E-01 | 1 | 77 | 0.513177 | 9.55E-01 | 1 | 77 | 0.569481 | 9.66E-01 | 1 | 77 | 0.563557 | 9.67E-01 | 1 | 77 | 0.548393 | 9.80E-01 |
| Melanoma | ko05218 | 1 | 80 | 0.5147745 | 9.85E-01 | 1 | 80 | 0.509589 | 9.77E-01 | 1 | 80 | 0.526664 | 9.55E-01 | 1 | 80 | 0.583412 | 9.66E-01 | 1 | 80 | 0.577455 | 9.67E-01 | 1 | 80 | 0.56219 | 9.80E-01 |
| Non-small cell lung cancer | ko05223 | 1 | 81 | 0.5191486 | 9.85E-01 | 1 | 81 | 0.513945 | 9.77E-01 | 1 | 81 | 0.531077 | 9.55E-01 | 1 | 81 | 0.587955 | 9.66E-01 | 1 | 81 | 0.581988 | 9.67E-01 | 1 | 81 | 0.566695 | 9.80E-01 |
| Glyoxylate and dicarboxylate metabolism | ko00630 | 1 | 82 | 0.5234835 | 9.85E-01 | 1 | 82 | 0.518263 | 9.77E-01 | 1 | 82 | 0.535448 | 9.55E-01 | 1 | 82 | 0.592449 | 9.66E-01 | 1 | 82 | 0.586473 | 9.67E-01 | 1 | 82 | 0.571154 | 9.80E-01 |
| Chronic myeloid leukemia | ko05220 | 1 | 83 | 0.5277794 | 9.85E-01 | 1 | 83 | 0.522542 | 9.77E-01 | 1 | 83 | 0.53978 | 9.55E-01 | 1 | 83 | 0.596894 | 9.66E-01 | 1 | 83 | 0.590911 | 9.67E-01 | 1 | 83 | 0.575567 | 9.80E-01 |
| Protein processing in endoplasmic reticulum | ko04141 | 6 | 653 | 0.535236 | 9.85E-01 | 6 | 653 | 0.520818 | 9.77E-01 | 6 | 653 | 0.568132 | 9.55E-01 | 7 | 653 | 0.567744 | 9.66E-01 | 7 | 653 | 0.550486 | 9.67E-01 | 6 | 653 | 0.663375 | 9.80E-01 |
| Type II diabetes mellitus | ko04930 | 1 | 85 | 0.5362561 | 9.85E-01 | 1 | 85 | 0.530988 | 9.77E-01 | 1 | 85 | 0.548322 | 9.55E-01 | 1 | 85 | 0.605639 | 9.66E-01 | 1 | 85 | 0.599644 | 9.67E-01 | 1 | 85 | 0.584258 | 9.80E-01 |
| Fc epsilon RI signaling pathway | ko04664 | 1 | 89 | 0.5527578 | 9.85E-01 | 1 | 89 | 0.547436 | 9.77E-01 | 1 | 89 | 0.564935 | 9.55E-01 | 1 | 89 | 0.622567 | 9.66E-01 | 1 | 89 | 0.616556 | 9.67E-01 | 1 | 89 | 0.601112 | 9.80E-01 |
| Cell cycle - yeast | ko04111 | 4 | 437 | 0.5544546 | 9.85E-01 | 4 | 437 | 0.542808 | 9.77E-01 | 6 | 437 | 0.222082 | 9.55E-01 | 6 | 437 | 0.339392 | 9.66E-01 | 6 | 437 | 0.325875 | 9.67E-01 | 6 | 437 | 0.292461 | 9.80E-01 |
| ErbB signaling pathway | ko04012 | 1 | 91 | 0.5607881 | 9.85E-01 | 1 | 91 | 0.555444 | 9.77E-01 | 1 | 91 | 0.573012 | 9.55E-01 | 1 | 91 | 0.630758 | 9.66E-01 | 1 | 91 | 0.624744 | 9.67E-01 | 1 | 91 | 0.609282 | 9.80E-01 |
| Long-term depression | ko04730 | 1 | 92 | 0.5647494 | 9.85E-01 | 1 | 92 | 0.559395 | 9.77E-01 | 1 | 92 | 0.576995 | 9.55E-01 | 1 | 92 | 0.634787 | 9.66E-01 | 1 | 92 | 0.628772 | 9.67E-01 | 1 | 92 | 0.613304 | 9.80E-01 |
| Citrate cycle (TCA cycle) | ko00020 | 1 | 94 | 0.5725654 | 9.85E-01 | 1 | 94 | 0.567192 | 9.77E-01 | 1 | 94 | 0.584849 | 9.55E-01 | 1 | 94 | 0.642713 | 9.66E-01 | 1 | 94 | 0.6367 | 9.67E-01 | 1 | 94 | 0.621226 | 9.80E-01 |
| Endometrial cancer | ko05213 | 1 | 95 | 0.576421 | 9.85E-01 | 1 | 95 | 0.571039 | 9.77E-01 | 1 | 95 | 0.588722 | 9.55E-01 | 1 | 95 | 0.646612 | 9.66E-01 | 1 | 95 | 0.640601 | 9.67E-01 | 1 | 95 | 0.625126 | 9.80E-01 |
| Osteoclast differentiation | ko04380 | 1 | 100 | 0.5951855 | 9.85E-01 | 1 | 100 | 0.589768 | 9.77E-01 | 1 | 100 | 0.607552 | 9.55E-01 | 1 | 343 | 0.977094 | 1.00E+00 | 1 | 100 | 0.659486 | 9.67E-01 | 1 | 100 | 0.644033 | 9.80E-01 |
| Pancreatic cancer | ko05212 | 1 | 104 | 0.6095996 | 9.85E-01 | 1 | 104 | 0.604164 | 9.77E-01 | 1 | 104 | 0.621996 | 9.55E-01 | 1 | 104 | 0.679848 | 9.66E-01 | 1 | 104 | 0.67388 | 9.67E-01 | 1 | 104 | 0.658474 | 9.80E-01 |
| Renal cell carcinoma | ko05211 | 1 | 104 | 0.6095996 | 9.85E-01 | 1 | 104 | 0.604164 | 9.77E-01 | 1 | 104 | 0.621996 | 9.55E-01 | 1 | 104 | 0.679848 | 9.66E-01 | 1 | 104 | 0.67388 | 9.67E-01 | 1 | 104 | 0.658474 | 9.80E-01 |
| Natural killer cell mediated cytotoxicity | ko04650 | 1 | 105 | 0.6131226 | 9.85E-01 | 1 | 105 | 0.607684 | 9.77E-01 | 1 | 105 | 0.625524 | 9.55E-01 | 1 | 105 | 0.683343 | 9.66E-01 | 1 | 105 | 0.677383 | 9.67E-01 | 1 | 105 | 0.661991 | 9.80E-01 |
| Regulation of actin cytoskeleton | ko04810 | 2 | 230 | 0.6142508 | 9.85E-01 | 1 | 230 | 0.871944 | 9.77E-01 | 1 | 230 | 0.884387 | 9.67E-01 | 2 | 230 | 0.715823 | 9.66E-01 | 2 | 230 | 0.70741 | 9.67E-01 | 1 | 230 | 0.907687 | 9.91E-01 |
| T cell receptor signaling pathway | ko04660 | 1 | 106 | 0.616614 | 9.85E-01 | 1 | 106 | 0.611173 | 9.77E-01 | 1 | 106 | 0.629019 | 9.55E-01 | 1 | 106 | 0.6868 | 9.66E-01 | 1 | 106 | 0.680848 | 9.67E-01 | 1 | 106 | 0.665473 | 9.80E-01 |
| Leishmaniasis | ko05140 | 4 | 489 | 0.6430006 | 9.85E-01 | 4 | 489 | 0.63154 | 9.77E-01 | 5 | 489 | 0.477547 | 9.55E-01 | 5 | 489 | 0.616281 | 9.66E-01 | 5 | 489 | 0.601938 | 9.67E-01 | 5 | 489 | 0.5648 | 9.80E-01 |
| Adherens junction | ko04520 | 1 | 114 | 0.6434394 | 9.85E-01 | 1 | 114 | 0.637993 | 9.77E-01 | 1 | 114 | 0.655834 | 9.55E-01 | 1 | 114 | 0.713137 | 9.66E-01 | 1 | 114 | 0.707269 | 9.67E-01 | 1 | 114 | 0.692073 | 9.80E-01 |
| Fatty acid metabolism | ko00071 | 1 | 114 | 0.6434394 | 9.85E-01 | 1 | 114 | 0.637993 | 9.77E-01 | 1 | 114 | 0.655834 | 9.55E-01 | 1 | 114 | 0.713137 | 9.66E-01 | 1 | 114 | 0.707269 | 9.67E-01 | 1 | 114 | 0.692073 | 9.80E-01 |
| VEGF signaling pathway | ko04370 | 1 | 114 | 0.6434394 | 9.85E-01 | 1 | 114 | 0.637993 | 9.77E-01 | 1 | 114 | 0.655834 | 9.55E-01 | 1 | 114 | 0.713137 | 9.66E-01 | 1 | 114 | 0.707269 | 9.67E-01 | 1 | 114 | 0.692073 | 9.80E-01 |
| Axon guidance | ko04360 | 1 | 115 | 0.6466584 | 9.85E-01 | 1 | 115 | 0.641214 | 9.77E-01 | 1 | 115 | 0.659047 | 9.55E-01 | 1 | 115 | 0.71627 | 9.66E-01 | 1 | 115 | 0.710414 | 9.67E-01 | 1 | 115 | 0.695246 | 9.80E-01 |
| Colorectal cancer | ko05210 | 1 | 115 | 0.6466584 | 9.85E-01 | 1 | 115 | 0.641214 | 9.77E-01 | 2 | 115 | 0.289523 | 9.55E-01 | 2 | 115 | 0.35597 | 9.66E-01 | 2 | 115 | 0.348674 | 9.67E-01 | 2 | 115 | 0.330322 | 9.80E-01 |
| Glioma | ko05214 | 1 | 116 | 0.6498485 | 9.85E-01 | 1 | 116 | 0.644405 | 9.77E-01 | 1 | 116 | 0.66223 | 9.55E-01 | 1 | 116 | 0.719369 | 9.66E-01 | 1 | 116 | 0.713526 | 9.67E-01 | 1 | 116 | 0.698387 | 9.80E-01 |
| Shigellosis | ko05131 | 1 | 116 | 0.6498485 | 9.85E-01 | 1 | 116 | 0.644405 | 9.77E-01 | 1 | 116 | 0.66223 | 9.55E-01 | 1 | 116 | 0.719369 | 9.66E-01 | 1 | 116 | 0.713526 | 9.67E-01 | 1 | 116 | 0.698387 | 9.80E-01 |
| Aminoacyl-tRNA biosynthesis | ko00970 | 1 | 119 | 0.659248 | 9.85E-01 | 1 | 119 | 0.653813 | 9.77E-01 | 1 | 119 | 0.671604 | 9.55E-01 | 3 | 119 | 0.14 | 9.66E-01 | 3 | 119 | 0.135234 | 9.67E-01 | 2 | 119 | 0.345264 | 9.80E-01 |
| B cell receptor signaling pathway | ko04662 | 1 | 119 | 0.659248 | 9.85E-01 | 1 | 119 | 0.653813 | 9.77E-01 | 1 | 119 | 0.671604 | 9.55E-01 | 1 | 119 | 0.728465 | 9.66E-01 | 1 | 119 | 0.722664 | 9.67E-01 | 1 | 119 | 0.707617 | 9.80E-01 |
| TGF-beta signaling pathway | ko04350 | 1 | 119 | 0.659248 | 9.85E-01 | 1 | 119 | 0.653813 | 9.77E-01 | 1 | 119 | 0.671604 | 9.55E-01 | 1 | 119 | 0.728465 | 9.66E-01 | 1 | 119 | 0.722664 | 9.67E-01 | 1 | 119 | 0.707617 | 9.80E-01 |
| Biosynthesis of unsaturated fatty acids | ko01040 | 1 | 121 | 0.6653743 | 9.85E-01 | 1 | 121 | 0.659946 | 9.77E-01 | 1 | 121 | 0.677709 | 9.55E-01 | 1 | 121 | 0.734365 | 9.66E-01 | 1 | 121 | 0.728593 | 9.67E-01 | 1 | 121 | 0.713613 | 9.80E-01 |
| Chemokine signaling pathway | ko04062 | 1 | 123 | 0.671391 | 9.85E-01 | 1 | 123 | 0.665971 | 9.77E-01 | 1 | 123 | 0.683701 | 9.55E-01 | 1 | 123 | 0.740138 | 9.66E-01 | 1 | 123 | 0.734397 | 9.67E-01 | 1 | 123 | 0.719487 | 9.80E-01 |
| Amino sugar and nucleotide sugar metabolism | ko00520 | 2 | 256 | 0.6720986 | 9.85E-01 | 2 | 256 | 0.664221 | 9.77E-01 | 2 | 256 | 0.689899 | 9.55E-01 | 2 | 256 | 0.769237 | 9.66E-01 | 2 | 256 | 0.76138 | 9.67E-01 | 2 | 256 | 0.740723 | 9.80E-01 |
| Alzheimer's disease | ko05010 | 2 | 257 | 0.6741773 | 9.85E-01 | 2 | 257 | 0.666309 | 9.77E-01 | 2 | 257 | 0.691953 | 9.55E-01 | 2 | 257 | 0.771106 | 9.66E-01 | 2 | 257 | 0.763273 | 9.67E-01 | 2 | 257 | 0.742673 | 9.80E-01 |
| Prostate cancer | ko05215 | 1 | 125 | 0.6773001 | 9.85E-01 | 1 | 125 | 0.67189 | 9.77E-01 | 1 | 125 | 0.689582 | 9.55E-01 | 1 | 125 | 0.745786 | 9.66E-01 | 1 | 125 | 0.740077 | 9.67E-01 | 1 | 125 | 0.725241 | 9.80E-01 |
| Thyroid cancer | ko05216 | 1 | 128 | 0.685966 | 9.85E-01 | 1 | 128 | 0.680574 | 9.77E-01 | 1 | 128 | 0.6982 | 9.55E-01 | 1 | 128 | 0.754029 | 9.66E-01 | 1 | 128 | 0.74837 | 9.67E-01 | 2 | 128 | 0.37846 | 9.80E-01 |
| Gap junction | ko04540 | 1 | 130 | 0.6916143 | 9.85E-01 | 1 | 130 | 0.686235 | 9.77E-01 | 1 | 130 | 0.703812 | 9.55E-01 | 1 | 130 | 0.759376 | 9.66E-01 | 1 | 130 | 0.753752 | 9.67E-01 | 1 | 130 | 0.739117 | 9.80E-01 |
| PPAR signaling pathway | ko03320 | 1 | 130 | 0.6916143 | 9.85E-01 | 1 | 130 | 0.686235 | 9.77E-01 | 1 | 130 | 0.703812 | 9.55E-01 | 2 | 130 | 0.413727 | 9.66E-01 | 2 | 130 | 0.405794 | 9.67E-01 | 2 | 130 | 0.385745 | 9.80E-01 |
| Glycerolipid metabolism | ko00561 | 1 | 131 | 0.6944004 | 9.85E-01 | 1 | 131 | 0.689028 | 9.77E-01 |  |  |  |  | 1 | 131 | 0.762006 | 9.66E-01 | 1 | 131 | 0.7564 | 9.67E-01 | 2 | 131 | 0.389373 | 9.80E-01 |
| Prion diseases | ko05020 | 1 | 131 | 0.6944004 | 9.85E-01 | 1 | 131 | 0.689028 | 9.77E-01 | 1 | 131 | 0.70658 | 9.55E-01 | 1 | 131 | 0.762006 | 9.66E-01 | 1 | 131 | 0.7564 | 9.67E-01 | 1 | 131 | 0.741808 | 9.80E-01 |
| Toll-like receptor signaling pathway | ko04620 | 4 | 535 | 0.7111296 | 9.85E-01 | 4 | 535 | 0.700266 | 9.77E-01 | 5 | 535 | 0.557047 | 9.55E-01 | 5 | 535 | 0.69373 | 9.66E-01 | 5 | 535 | 0.680078 | 9.67E-01 | 5 | 535 | 0.644206 | 9.80E-01 |
| Vascular smooth muscle contraction | ko04270 | 1 | 142 | 0.7234424 | 9.85E-01 | 1 | 142 | 0.718168 | 9.77E-01 | 1 | 142 | 0.735372 | 9.55E-01 | 1 | 142 | 0.789112 | 9.66E-01 | 1 | 142 | 0.783721 | 9.67E-01 | 1 | 142 | 0.769639 | 9.80E-01 |
| Inositol phosphate metabolism | ko00562 | 1 | 143 | 0.7259423 | 9.85E-01 |  |  |  |  |  |  |  |  | 1 | 143 | 0.791418 | 9.66E-01 | 1 | 143 | 0.786048 | 9.67E-01 |  |  |  |  |
| Melanogenesis | ko04916 | 1 | 144 | 0.7284196 | 9.85E-01 | 1 | 144 | 0.723166 | 9.77E-01 | 1 | 144 | 0.740297 | 9.55E-01 | 1 | 144 | 0.793699 | 9.66E-01 | 1 | 144 | 0.78835 | 9.67E-01 | 1 | 144 | 0.774368 | 9.80E-01 |
| GnRH signaling pathway | ko04912 | 1 | 147 | 0.7357188 | 9.85E-01 | 1 | 147 | 0.730498 | 9.77E-01 | 1 | 147 | 0.747513 | 9.55E-01 | 1 | 147 | 0.800394 | 9.66E-01 | 1 | 147 | 0.795109 | 9.67E-01 | 1 | 147 | 0.781281 | 9.80E-01 |
| Carbon fixation in photosynthetic organisms | ko00710 | 1 | 151 | 0.7451483 | 9.85E-01 | 1 | 151 | 0.739975 | 9.77E-01 | 1 | 151 | 0.756825 | 9.55E-01 | 1 | 151 | 0.808985 | 9.66E-01 | 1 | 151 | 0.803788 | 9.67E-01 | 1 | 151 | 0.790172 | 9.80E-01 |
| Phosphatidylinositol signaling system | ko04070 | 1 | 151 | 0.7451483 | 9.85E-01 |  |  |  |  |  |  |  |  | 1 | 151 | 0.808985 | 9.66E-01 | 1 | 151 | 0.803788 | 9.67E-01 |  |  |  |  |
| mTOR signaling pathway | ko04150 | 1 | 153 | 0.7497367 | 9.85E-01 | 1 | 153 | 0.744588 | 9.77E-01 | 1 | 153 | 0.761352 | 9.55E-01 | 1 | 153 | 0.813141 | 9.66E-01 | 1 | 153 | 0.807989 | 9.67E-01 | 1 | 153 | 0.794482 | 9.80E-01 |
| Limonene and pinene degradation | ko00903 | 2 | 301 | 0.75547 | 9.85E-01 | 2 | 301 | 0.748202 | 9.77E-01 | 2 | 301 | 0.771728 | 9.55E-01 | 2 | 301 | 0.841236 | 9.66E-01 | 2 | 301 | 0.834582 | 9.67E-01 | 2 | 301 | 0.816839 | 9.80E-01 |
| Bisphenol degradation | ko00363 | 2 | 302 | 0.7570967 | 9.85E-01 | 2 | 302 | 0.749846 | 9.77E-01 | 2 | 302 | 0.773313 | 9.55E-01 | 2 | 302 | 0.842577 | 9.66E-01 | 2 | 302 | 0.835952 | 9.67E-01 | 2 | 302 | 0.818278 | 9.80E-01 |
| Toxoplasmosis | ko05145 | 4 | 581 | 0.7693327 | 9.85E-01 | 4 | 581 | 0.759347 | 9.77E-01 | 5 | 581 | 0.630185 | 9.55E-01 | 5 | 581 | 0.759842 | 9.66E-01 | 5 | 581 | 0.747337 | 9.67E-01 | 5 | 581 | 0.713992 | 9.80E-01 |
| Porphyrin and chlorophyll metabolism | ko00860 | 1 | 163 | 0.7714744 | 9.85E-01 | 1 | 163 | 0.766462 | 9.77E-01 | 1 | 163 | 0.782759 | 9.55E-01 | 1 | 163 | 0.832611 | 9.66E-01 | 1 | 163 | 0.827688 | 9.67E-01 | 1 | 163 | 0.814742 | 9.80E-01 |
| Long-term potentiation | ko04720 | 1 | 169 | 0.7836041 | 9.85E-01 | 1 | 169 | 0.77868 | 9.77E-01 | 1 | 169 | 0.794674 | 9.55E-01 | 1 | 169 | 0.843308 | 9.66E-01 | 1 | 169 | 0.838527 | 9.67E-01 | 1 | 169 | 0.82593 | 9.80E-01 |
| Polycyclic aromatic hydrocarbon degradation | ko00624 | 2 | 321 | 0.7862771 | 9.85E-01 | 2 | 321 | 0.779374 | 9.77E-01 | 2 | 321 | 0.801652 | 9.55E-01 | 2 | 321 | 0.866184 | 9.66E-01 | 2 | 321 | 0.8601 | 9.67E-01 | 2 | 321 | 0.843773 | 9.80E-01 |
| Neurotrophin signaling pathway | ko04722 | 4 | 597 | 0.7873068 | 9.85E-01 | 4 | 597 | 0.777671 | 9.77E-01 | 5 | 597 | 0.653856 | 9.55E-01 | 5 | 597 | 0.780167 | 9.66E-01 | 5 | 597 | 0.768134 | 9.67E-01 | 5 | 597 | 0.735881 | 9.80E-01 |
| Alpha-Linolenic acid metabolism | ko00592 | 1 | 175 | 0.795093 | 9.85E-01 | 1 | 175 | 0.790262 | 9.77E-01 | 1 | 175 | 0.805939 | 9.55E-01 | 1 | 175 | 0.853325 | 9.66E-01 | 1 | 175 | 0.848687 | 9.67E-01 | 1 | 175 | 0.836446 | 9.80E-01 |
| Focal adhesion | ko04510 | 1 | 181 | 0.8059746 | 9.85E-01 | 1 | 181 | 0.801241 | 9.77E-01 | 1 | 181 | 0.816588 | 9.55E-01 | 1 | 181 | 0.862703 | 9.66E-01 | 1 | 181 | 0.85821 | 9.67E-01 | 1 | 181 | 0.846328 | 9.80E-01 |
| Pyruvate metabolism | ko00620 | 1 | 183 | 0.8094725 | 9.85E-01 | 1 | 183 | 0.804772 | 9.77E-01 | 1 | 183 | 0.820007 | 9.55E-01 | 1 | 183 | 0.865695 | 9.66E-01 | 1 | 183 | 0.86125 | 9.67E-01 | 1 | 183 | 0.849489 | 9.80E-01 |
| Aminobenzoate degradation | ko00627 | 2 | 342 | 0.8149165 | 9.85E-01 | 2 | 342 | 0.808431 | 9.77E-01 | 2 | 342 | 0.829292 | 9.55E-01 | 2 | 342 | 0.888478 | 9.66E-01 | 2 | 342 | 0.882988 | 9.67E-01 | 2 | 342 | 0.868154 | 9.80E-01 |
| Progesterone-mediated oocyte maturation | ko04914 | 1 | 192 | 0.8244523 | 9.85E-01 | 1 | 192 | 0.819905 | 9.77E-01 | 1 | 192 | 0.834624 | 9.55E-01 | 1 | 192 | 0.878373 | 9.66E-01 | 1 | 192 | 0.874146 | 9.67E-01 | 1 | 192 | 0.862928 | 9.80E-01 |
| Glycerophospholipid metabolism | ko00564 | 1 | 194 | 0.8276186 | 9.85E-01 | 1 | 194 | 0.823106 | 9.77E-01 | 1 | 194 | 0.837708 | 9.55E-01 | 1 | 194 | 0.881024 | 9.66E-01 | 1 | 194 | 0.876845 | 9.67E-01 | 1 | 194 | 0.865748 | 9.80E-01 |
| ABC transporters | ko02010 | 1 | 195 | 0.8291804 | 9.85E-01 | 1 | 195 | 0.824685 | 9.77E-01 | 1 | 195 | 0.839229 | 9.55E-01 | 3 | 195 | 0.355822 | 9.66E-01 | 3 | 195 | 0.346502 | 9.67E-01 | 3 | 195 | 0.323164 | 9.80E-01 |
| Bile secretion | ko04976 | 1 | 200 | 0.8367805 | 9.85E-01 | 1 | 200 | 0.832373 | 9.77E-01 | 1 | 200 | 0.846622 | 9.55E-01 | 3 | 200 | 0.3706 | 9.66E-01 | 3 | 200 | 0.361071 | 9.67E-01 | 3 | 200 | 0.337171 | 9.80E-01 |
| Apoptosis | ko04210 | 3 | 507 | 0.8368086 | 9.85E-01 | 3 | 507 | 0.829429 | 9.77E-01 | 4 | 507 | 0.696125 | 9.55E-01 | 4 | 507 | 0.804103 | 9.66E-01 | 4 | 507 | 0.793868 | 9.67E-01 | 4 | 507 | 0.766406 | 9.80E-01 |
| Glutathione metabolism | ko00480 | 1 | 204 | 0.8426176 | 9.85E-01 |  |  |  |  |  |  |  |  |  |  |  |  |  |  |  |  |  |  |  |  |
| Measles | ko05162 | 3 | 533 | 0.8606268 | 9.85E-01 | 3 | 533 | 0.853849 | 9.77E-01 | 4 | 533 | 0.732681 | 9.55E-01 | 4 | 533 | 0.834012 | 9.66E-01 | 4 | 533 | 0.824598 | 9.67E-01 | 4 | 533 | 0.799127 | 9.80E-01 |
| Fc gamma R-mediated phagocytosis | ko04666 | 1 | 221 | 0.8651945 | 9.85E-01 | 1 | 221 | 0.861164 | 9.77E-01 | 1 | 221 | 0.874151 | 9.67E-01 | 2 | 221 | 0.69506 | 9.66E-01 | 2 | 221 | 0.686494 | 9.67E-01 | 1 | 221 | 0.89862 | 9.91E-01 |
| Ribosome biogenesis in eukaryotes | ko03008 | 2 | 397 | 0.8743612 | 9.85E-01 | 2 | 397 | 0.869043 | 9.77E-01 | 2 | 397 | 0.886006 | 9.67E-01 | 2 | 397 | 0.931607 | 9.95E-01 | 2 | 397 | 0.927554 | 9.88E-01 | 2 | 397 | 0.916409 | 9.91E-01 |
| Oxidative phosphorylation | ko00190 | 1 | 230 | 0.8758105 | 9.85E-01 |  |  |  |  |  |  |  |  | 1 | 55 | 0.452111 | 9.66E-01 |  |  |  |  |  |  |  |  |
| MAPK signaling pathway | ko04010 | 1 | 237 | 0.8834885 | 9.85E-01 | 1 | 237 | 0.879748 | 9.77E-01 | 1 | 237 | 0.891772 | 9.67E-01 | 1 | 237 | 0.925962 | 9.95E-01 | 1 | 237 | 0.92277 | 9.88E-01 | 1 | 237 | 0.914176 | 9.91E-01 |
| Ubiquitin mediated proteolysis | ko04120 | 2 | 413 | 0.8880384 | 9.85E-01 | 2 | 413 | 0.883059 | 9.77E-01 | 3 | 413 | 0.740983 | 9.55E-01 | 4 | 413 | 0.659374 | 9.66E-01 | 4 | 413 | 0.646849 | 9.67E-01 | 4 | 413 | 0.614233 | 9.80E-01 |
| Stilbenoid, diarylheptanoid and gingerol biosynthesis | ko00945 | 2 | 433 | 0.9032014 | 9.85E-01 | 2 | 433 | 0.898634 | 9.77E-01 | 2 | 433 | 0.913122 | 9.77E-01 | 2 | 433 | 0.950729 | 9.99E-01 | 2 | 433 | 0.94748 | 9.88E-01 | 2 | 433 | 0.938443 | 9.96E-01 |
| Pathways in cancer | ko05200 | 1 | 258 | 0.9038016 | 9.85E-01 | 1 | 258 | 0.900433 | 9.77E-01 | 2 | 258 | 0.693995 | 9.55E-01 | 2 | 258 | 0.772962 | 9.66E-01 | 2 | 258 | 0.765153 | 9.67E-01 | 3 | 258 | 0.49465 | 9.80E-01 |
| Peroxisome | ko04146 | 1 | 275 | 0.9176322 | 9.85E-01 | 1 | 275 | 0.914553 | 9.77E-01 | 1 | 275 | 0.924393 | 9.77E-01 | 1 | 275 | 0.951351 | 9.99E-01 | 1 | 275 | 0.948906 | 9.88E-01 | 1 | 275 | 0.942246 | 9.96E-01 |
| Pentose and glucuronate interconversions | ko00040 | 1 | 277 | 0.9191233 | 9.85E-01 | 1 | 277 | 0.916078 | 9.77E-01 | 1 | 277 | 0.925809 | 9.77E-01 | 2 | 277 | 0.805808 | 9.66E-01 | 2 | 277 | 0.798489 | 9.67E-01 | 2 | 277 | 0.779118 | 9.80E-01 |
| Pyrimidine metabolism | ko00240 | 1 | 302 | 0.935644 | 9.87E-01 |  |  |  |  |  |  |  |  | 1 | 302 | 0.963917 | 1.00E+00 | 1 | 302 | 0.96192 | 9.89E-01 | 1 | 302 | 0.956432 | 9.96E-01 |
| Insulin signaling pathway | ko04910 | 1 | 312 | 0.9412694 | 9.87E-01 | 1 | 312 | 0.93877 | 9.85E-01 | 1 | 312 | 0.946712 | 9.86E-01 | 1 | 312 | 0.9677 | 1.00E+00 | 1 | 312 | 0.965851 | 9.89E-01 | 1 | 312 | 0.960754 | 9.96E-01 |
| Cell cycle | ko04110 | 1 | 343 | 0.9557814 | 9.87E-01 | 1 | 343 | 0.953707 | 9.85E-01 | 3 | 343 | 0.620283 | 9.55E-01 | 3 | 343 | 0.722937 | 9.66E-01 | 3 | 343 | 0.712696 | 9.67E-01 | 3 | 343 | 0.685828 | 9.80E-01 |
| Oocyte meiosis | ko04114 | 1 | 343 | 0.9557814 | 9.87E-01 | 1 | 343 | 0.953707 | 9.85E-01 | 1 | 343 | 0.960268 | 9.86E-01 | 1 | 46 | 0.395362 | 9.66E-01 | 1 | 343 | 0.975648 | 9.91E-01 | 1 | 343 | 0.97162 | 9.96E-01 |
| Microbial metabolism in diverse environments | ko01120 | 5 | 1052 | 0.9623292 | 9.87E-01 | 5 | 1052 | 0.958776 | 9.85E-01 | 5 | 1052 | 0.969552 | 9.87E-01 | 5 | 1052 | 0.990317 | 1.00E+00 | 7 | 1052 | 0.936461 | 9.88E-01 | 7 | 1052 | 0.918303 | 9.91E-01 |
| Spliceosome | ko03040 | 2 | 687 | 0.9862985 | 1.00E+00 | 2 | 687 | 0.985162 | 1.00E+00 | 2 | 687 | 0.98863 | 9.97E-01 | 2 | 687 | 0.995701 | 1.00E+00 | 2 | 687 | 0.995206 | 1.00E+00 | 2 | 687 | 0.99371 | 1.00E+00 |
| RNA transport | ko03013 | 1 | 619 | 0.9965278 | 1.00E+00 | 1 | 619 | 0.996226 | 1.00E+00 | 1 | 333 | 0.95632 | 9.86E-01 |  |  |  |  |  |  |  |  | 2 | 619 | 0.988216 | 1.00E+00 |
| Biosynthesis of secondary metabolites | ko01110 | 11 | 2877 | 0.9998375 | 1.00E+00 | 11 | 2877 | 0.999789 | 1.00E+00 | 11 | 2877 | 0.999913 | 1.00E+00 | 11 | 2877 | 0.999997 | 1.00E+00 | 13 | 2877 | 0.999957 | 1.00E+00 | 13 | 2877 | 0.999899 | 1.00E+00 |
| Basal transcription factors | ko03022 |  |  |  |  |  |  |  |  |  |  |  |  | 4 | 139 | 0.065361 | 9.66E-01 | 4 | 139 | 0.062338 | 9.67E-01 |  |  |  |  |
| DNA replication | ko03030 |  |  |  |  |  |  |  |  | 2 | 124 | 0.320329 | 9.55E-01 | 3 | 124 | 0.152695 | 9.66E-01 | 3 | 124 | 0.147581 | 9.67E-01 | 3 | 124 | 0.135053 | 9.80E-01 |
| Endocytosis | ko04144 |  |  |  |  |  |  |  |  |  |  |  |  | 5 | 258 | 0.150807 | 9.66E-01 | 1 | 258 | 0.938529 | 9.88E-01 | 4 | 258 | 0.273059 | 9.80E-01 |
| Glycolysis / Gluconeogenesis | ko00010 |  |  |  |  |  |  |  |  |  |  |  |  |  |  |  |  | 2 | 231 | 0.709661 | 9.67E-01 | 2 | 231 | 0.687762 | 9.80E-01 |
| Homologous recombination | ko03440 |  |  |  |  |  |  |  |  |  |  |  |  |  |  |  |  |  |  |  |  | 1 | 112 | 0.685627 | 9.80E-01 |
| Inositol alkaloid biosynthesis | ko00562 |  |  |  |  |  |  |  |  |  |  |  |  | 1 | 41 | 0.361343 | 9.66E-01 | 1 | 41 | 0.356683 | 9.67E-01 |  |  |  |  |
| MAPK signaling pathway - yeast | ko04011 |  |  |  |  |  |  |  |  |  |  |  |  |  |  |  |  |  |  |  |  | 1 | 112 | 0.685627 | 9.80E-01 |
| Meiosis - yeast | ko04113 |  |  |  |  |  |  |  |  | 2 | 225 | 0.620558 | 9.55E-01 | 2 | 225 | 0.704438 | 9.66E-01 | 2 | 225 | 0.695937 | 9.67E-01 | 2 | 225 | 0.673804 | 9.80E-01 |
| Mismatch repair | ko03430 |  |  |  |  |  |  |  |  | 1 | 87 | 0.556706 | 9.55E-01 | 2 | 87 | 0.244021 | 9.66E-01 | 2 | 87 | 0.238384 | 9.67E-01 | 2 | 87 | 0.224328 | 9.80E-01 |
| Nucleotide excision repair | ko03420 |  |  |  |  |  |  |  |  |  |  |  |  |  |  |  |  | 1 | 190 | 0.871388 | 9.67E-01 | 1 | 190 | 0.860049 | 9.80E-01 |
| Regulation of autophagy | ko04140 |  |  |  |  |  |  |  |  |  |  |  |  | 1 | 152 | 0.811075 | 9.66E-01 | 1 | 152 | 0.8059 | 9.67E-01 |  |  |  |  |
| RNA degradation | ko03018 |  |  |  |  |  |  |  |  |  |  |  |  | 2 | 333 | 0.879379 | 9.66E-01 | 2 | 333 | 0.873636 | 9.67E-01 | 1 | 333 | 0.96849 | 9.96E-01 |
| Valine, leucine and isoleucine biosynthesis | ko00290 |  |  |  |  |  |  |  |  |  |  |  |  | 1 | 68 | 0.524844 | 9.66E-01 | 1 | 68 | 0.519076 | 9.67E-01 |  |  |  |  |
