# Supplementary material for: Genome-wide identification of alternate bearing-associated microRNAs (miRNAs) in olive (Olea europaea L.)
Source: BMC Plant Biol. 2013 Jan 15;13:10. doi: 10.1186/1471-2229-13-10 (PMC3564680; doi:10.1186/1471-2229-13-10)
Supplement: Additional file 8 — Primer sequences of selected miRNA and target genes for qRT–PCR validation experiments. [file 1471-2229-13-10-S8.doc]

**Additional file 7. Primer sequences of selected miRNA and target genes for qRT–PCR validation experiments.**

| Oeu-miRNAs primers | RT Primers  (5'->3') | Forward Primers(F)  (5'->3') | Universal Reverse Primer(R)  (5'->3') |
| --- | --- | --- | --- |
| Oeu-miR159 | 5’-GTCGTATCCAGTGCAGGGTCCGAGGTATTCGCACTGG-3’ | 5’-GCGGCGGTATTGGAGTGAAGGGA-3’ | 5'-GTGCAGGGTCCGAGGT-3' |
| Oeu-miR164 | 5’-GTCGTATCCAGTGCAGGGTCCGAGGTATTCGCACTGG-3’ | 5’-TCGCTTGGAGAAGCAGGGCA-3’ |
| Oeu-miR166 | 5’-GTCGTATCCAGTGCAGGGTCCGAGGTATTCGCACTGG-3’ | 5'-GCGGCGGTCGGACCAGGCTTCAT-3’ |
| Oeu-miR168 | 5'-GTCGTATCCAGTGCAGGGTCCGAGGTATTCGCACTGGATACGACATTCAC-3' | 5'-TTCCTTGATCCCGCCTTGCACCAA-3' |
| Oeu-miR171 | 5’-GTCGTATCCAGTGCAGGGTCCGAGGTATTCGCACTGG-3’ | 5’-TTCCTTATTGAGCCGTGCC-3’ |
| Oeu-miR395 | 5'-GTCGTATCCAGTGCAGGGTCCGAGGTATTCGCACTGG-3' | 5'-CGGCGGCTGAAGTGTTTGGGGG-3' |
| Oeu-miR396 | 5’-GTCGTATCCAGTGCAGGGTCCGAGGTATTCGCACTGGATACGACAGTTCA-3’ | 5’-TCGCGTTCCACAGCTTTCT-3’ |
| Oeu-miR156 | 5’-GTCGTATCCAGTGCAGGGTCCGAGGTATTCGCACTGGATACGACGTGCTC-3’ | 5’-TCGCGTGACAGAAGAGAGA-3’ |
| Oeu-miR172 | 5’-GTCGTATCCAGTGCAGGGTCCGAGGTATTCGCACTGGATACGACCAGCAG-3’ | 5’-CGGCGGAGGATCTTGATGATG-3’ |

| Oeu-Tar-miRNAs primers | Forward Primers(F)  (5'->3') | Reverse Primers(R)  (5'->3') |
| --- | --- | --- |
| Oeu-Tar-miR159 | 5’-AGGTAACGGCAGGGTGGAA-3’ | 5’-CAATAGCATGACCCCAACGAG-3’ |
| Oeu-Tar-miR164 | 5’-TCAGCCACGCTCACAGGCCT-3’ | 5’-GTCGGTCAGGAGGCTCTCCG-3’ |
| Oeu-Tar-miR166 | 5’-GCTCAATGAAGGCCTTGCCCG-3’ | 5’-GCACGCGAAGCCCTGTTGCA-3’ |
| Oeu-Tar-miR168-2 | 5’-CGCCCGATAGATCGAGAAA -3’ | 5’-AGCCTCAACAGAAGCCAGAG-3’ |
| Oeu-Tar-miR171 | 5’-GCCGTCAACGCCAACCTCGA-3’ | 5’-AGCCTCTCACTGGCGTCCGT-3’ |
| Oeu-Tar-miR395 | 5’-TGTTGGTCGCGATCCTGCCG-3’ | 5’-CGCCAACCCACGCATCTTGGT-3’ |
| Oeu-Tar-miR396 | 5'-CTGCAGTGGATCATGGTGT-3 | 5’-GCCCTTCTCTCCCCATTTAG-3’ |
